# Limited evidence of physical therapy on balance after stroke: A systematic review and meta-analysis

**DOI:** 10.1371/journal.pone.0221700

**Published:** 2019-08-29

**Authors:** Aurélien Hugues, Julie Di Marco, Shams Ribault, Hugo Ardaillon, Perrine Janiaud, Yufeng Xue, Jin Zhu, Jennifer Pires, Hooman Khademi, Laura Rubio, Paloma Hernandez Bernal, Yeliz Bahar, Hadrien Charvat, Pawel Szulc, Carolina Ciumas, Heiwon Won, Michel Cucherat, Isabelle Bonan, François Gueyffier, Gilles Rode

**Affiliations:** 1 Service de médecine physique et réadaptation, hôpital Henry Gabrielle, Hospices Civils de Lyon, Saint-Genis-Laval, France; 2 Plate-forme “Mouvement et Handicap”, hôpital Henry Gabrielle, Hospices Civils de Lyon, Saint-Genis-Laval, France; 3 Equipe “ImpAct”, Centre de Recherche en Neurosciences de Lyon, Inserm UMR-S 1028, CNRS UMR 5292, Université de Lyon, Université Lyon 1, Bron, France; 4 Assistance Publique des Hôpitaux de Paris, Université Paris Descartes, Paris, France; 5 UMR 5558 CNRS Lyon, Université de Lyon, Université Lyon 1, Lyon, France; 6 Université de Lyon, Université Claude Bernard Lyon 1, Université Saint-Étienne, HESPER EA 7425, Lyon, Saint-Etienne, France; 7 Département de pharmacologie, Université Jiaotong de Shanghai, Shanghai, Chine; 8 Rovisco Pais Rehabilitation Centre, Tocha, Portugal; 9 Medicine Faculty of Oporto University, Oporto, Portugal; 10 International Agency for Research on Cancer, World Health Organization, Lyon, France; 11 Centro Lescer, Madrid, Spain; 12 Rehaklinik Zihlschlach, Neurologisches Rehabilitationszentrum, Zihlschlacht, Switzerland; 13 Hitit University Erol Olcok Training and Research Hospital, Corum, Turkey; 14 Division of Prevention, Center for Public Health Sciences, National Cancer, Tokyo, Japan; 15 INSERM UMR 1033, Université de Lyon, Université Lyon 1, Hôpital Edouard Herriot, Lyon, France; 16 Translational and Integrative Group in Epilepsy Research, INSERM U1028, CNRS UMR5292, Centre de Recherche en Neuroscience de Lyon, Université de Lyon, Université Lyon1, Lyon, France; 17 Institut des Epilepsies, Université de Lyon, Université Lyon 1, Lyon, France; 18 Department of Clinical Neurosciences, CHUV, Lausanne, Switzerland; 19 UMR 5316 Litt&Arts, Université Grenoble Alpes, Grenoble, France; 20 KyungHee University, Seoul, South Korea; 21 Service Hospitalo-Universitaire de Pharmaco-Toxicologie, Groupement Hospitalier Est, Hospices Civils de Lyon, Bron, France; 22 Service de médecine physique et de réadaptation, CHU Rennes, Rennes, France; 23 Equipe “VisAGeS”, Inserm Unité U746, Université Rennes 1, Rennes, France; University of Mississippi Medical Center, UNITED STATES

## Abstract

**Background:**

Stroke results in balance disorders and these directly affect autonomy and quality of life. The purpose of this systematic review and meta-analysis was to determine the efficacy of physical therapy (PT) on balance and postural control after stroke.

**Methods:**

We included all randomized controlled trials assessing the efficacy of PT on balance and postural control in adult patients after stroke without language restriction. Medline, Embase/Scopus, Cochrane Central Register of Controlled Trials, PEDro, Pascal, and Francis databases were searched until January 2019. Primary outcomes were balance (Berg Balance scale and Postural Assessment Scale for Stroke) and postural control with postural deviation or stability measurement in sitting or standing static evaluation. A pair of independent reviewers selected studies, extracted data, and assessed risk of bias. Meta-analyses with subgroups (categories of PT, time post-stroke, and lesion location) and meta-regression (duration of PT) were conducted.

**Results:**

A total of 145 studies (n = 5912) were selected from the 13,123 records identified. For balance, evidence was found in favor of the efficacy of functional task-training alone (standardized mean difference 0.39, 95% confidence interval [0.09; 0.68], heterogeneity I^2^ = 63%) or associated with musculoskeletal intervention and/or cardiopulmonary intervention (0.37, [0.19; 0.55], I^2^ = 48%), electrostimulation (0.91, [0.49; 1.34], I^2^ = 52%) immediately after intervention, compared to sham treatment or usual care (ST/UC). For postural deviation eyes open, assistive devices were more effective than no treatment (-0.21, [-0.37; -0.05], I^2^ = 0%) immediately after intervention; for postural stability eyes open, functional task-training and sensory interventions were more effective than ST/UC (0.97, [0.35; 1.59], I^2^ = 65% and 0.80, [0.46; 1.13], I^2^ = 37% respectively) immediately after intervention.

**Conclusions:**

Functional task-training associated with musculoskeletal intervention and/or cardiopulmonary intervention and sensory interventions seem to be immediately effective in improving balance and postural stability, respectively. The heterogeneity of PT and the weak methodological quality of studies limited the interpretation and the confidence in findings.

## Introduction

World-wide, approximately 25.7 million people suffered from stroke in 2013 [1], and this was the third most common cause of disability in 2015 [2]. Stroke frequently results in postural disorders characterized by a mediolateral deviation towards the unaffected lower limb and a greater instability of the center of pressure [3–11]. These dysfunctions lead to balance disorders [12] that are responsible for an increased risk of falls [13] and a lower level of activity and participation in stroke patients [14,15]. Balance is associated with ambulation abilities [16–18] and quality of life [19]. Moreover, balance is a predictor for achieving the ability to walk [16,20,21] and is also found among the factors potentially modifiable by physical activity [22]. Therefore, developing physical therapy (PT) interventions for the improvement of balance is relevant for patients with stroke.

PT includes interventions aiming to develop, maintain, and restore movement and functional ability [23]. Current recommendations regarding PT for the improvement of balance after stroke are based on a poor level of evidence [24–26]. Furthermore, most meta-analyses selected only studies published in English language despite it having been established that significant results are more often published in English-language journals [27,28], introducing language bias into article selection. In addition, among the meta-analyses that have investigated the effects of PT in patients with stroke these considered multiple outcomes or some specific approaches of PT [29–42]. Although these did include balance, to the best of our knowledge no meta-analysis has investigated the effects of all PTs specifically on balance and postural control after stroke without language restriction. Therefore, the objective of this systematic review and meta-analysis was to determine the efficacy of PT (overall and by category of PT) on these parameters in adult patients with stroke.

## Methods

The protocol was developed using the PRISMA guidelines [43] and Cochrane recommendations [27], registered in PROSPERO (CRD42016037966), and published in BMJOpen [44] (S1 Checklist and S1 Protocol). Therefore, methods are described only briefly.

### Definitions

According to the World Health Organization, stroke is defined as “rapidly developing clinical signs of focal (at times global) disturbance of cerebral function, lasting more than 24 h or leading to death with no apparent cause other than that of vascular origin” [45]. PT is defined by the World Confederation for Physical Therapy as “services to individuals and populations to develop, maintain and restore maximum movement and functional ability throughout the life-span” and “physical therapy is concerned with identifying and maximizing quality of life and movement potential within the spheres of promotion, prevention, treatment/intervention, habilitation and rehabilitation” (http://www.wcpt.org/policy/ps-descriptionPT) [23]. Human posture is the position of body parts relative to each other [46]. We defined postural control as the function of body stabilization based on a sensorimotor complex skill, and of body orientation based on internal representation of body scheme [47]. We further defined balance as a posture in which an ideal body mass distribution is achieved and which provides the body carriage stability and conditions for normal functions in stationary position or in movement (Medline Subject Heading; MeSH).

### Eligibility criteria

All types of randomized controlled trials assessing the efficacy of PT on balance or postural control in adult patients (18 years or above) with stroke were included without language restriction. Inspired by the meta-analysis conducted by Pollock *et al*. [40], we included all PTs that may be used by physiotherapists during rehabilitation without restriction to only PTs that had a stated objective of promoting recovery of balance or postural control. We included PTs using electric devices (such as functional electric stimulation), treadmills, and assistive devices (such as a cane or orthosis). The classification of PT categories, based on that used by Pollock *et al*. [40], included assistive devices, constraint-induced therapy, cardiopulmonary intervention, functional task-training, musculoskeletal intervention, sensory interventions, and other intervention (Table 1). Only the outcomes defined as primary in the following paragraph were considered for selection of trials.

**Table 1:** Categories of physical therapy.

Categories	Component of categories		Definition
Assistive devices	Cane and aid to stand or walk		Described in additional Table 2 in Pollock *et al*., 2014, p. 361 [40]: “Devices to assist walking, including sticks and frames”
	Orthosis		Described in additional Table 2 in Pollock *et al*., 2014, p. 361 [40]: “Externally applied orthoses to assist walking, including AFO, knee braces”
Constraint-induced therapy	Weight, resistance		Passive and external constraint imposed on movements or mobility of patients
	Body or limb positioning		
	Wedge, lift		
Cardiopulmonary intervention	Fitness, endurance, aerobic training		Described in additional Table 2 in Pollock *et al*., 2014, p. 361 [40]: “Activities to improve cardiopulmonary fitness”
Functional task-training	Balance training		Task-oriented training specifically focus on balance in various modalities.
	Gait training		Task-oriented training of specifically focus on gait in various modalities.
	Sit-to-stand training		Task-oriented training of specifically focus on sit-to-stand in various modalities.
	Transfer training		Task-oriented training of specifically focus on transfers in various modalities.
	Reach or upper limb training		Task-oriented training of specifically focus on reach or function of upper limb in various modalities.
	Daily activity training		Task-oriented training of specifically focus on activities of daily living in various modalities.
	Other task-oriented training		Other task-oriented training in various modalities such as coordination tasks
Musculoskeletal intervention	Active	Strengthening	Described in additional Table 2 in Pollock *et al*., 2014, p. 362 [40]: “Practice of activities to progressively increase the ability to generate muscle force, including using body weight and external resistance”
		Mobilization	Described in additional Table 2 in Pollock *et al*., 2014, p. 362 [40]: “Moving a limb through its range of movement, under the patient’s active control without assistance”
	Active assisted	Mobilization	Described in additional Table 2 in Pollock *et al*., 2014, p. 362 [40]: “Moving a limb through its range of movement, under the patient’s active control with assistance”
		Electrostimulation	Electrical current used to produce a muscle contraction
	Passive	Mobilization	Described in additional Table 2 in Pollock *et al*., 2014, p. 362 [40]: “Moving a limb through its range of movement, whilst the patient is passive”
		Stretching	Lengthening of muscle to improve elasticity and control muscle tone.
		Immobilization	Described in additional Table 2 in Pollock *et al*., 2014, p. 362 [40]: “placing a limb or body part in a supported position, to maintain optimal alignment “
		Verticalization	Described in additional Table 2 in Pollock *et al*., 2014, p. 362 [40]: “To promote early lower limb loading”
		Massage	Described in additional Table 2 in Pollock *et al*., 2014, p. 362 [40]: “Manipulation of soft tissue, using the hands or a tool designed for the purpose
Neurophysiological intervention	Bobath, Proprioceptive neuromuscular facilitation and other neurodevelopmental interventions		Described in additional Table 1 in Pollock *et al*., 2014, p. 356–362 [40]: “Intervention which is described as facilitation of movement”
Sensory interventions	Tactile, vibration, thermal, proprioception		Practice of stimulation, perturbation or modification of sensorial input (*e*.*g*. tactile, thermal, proprioception, visual, vestibular) by different methods.
	Visual		
	Vestibular		
Other intervention	Acupuncture		Described in additional Table 2 in Pollock *et al*., 2014, p. 362 [40]: “Devices to assist walking, including sticks and frames”
	Aquatic therapy		Use of aquatic environment to assist or stimulate function or mobility of body
	Body awareness therapy		Practice aimed at being aware of one owns body and reflect upon how the body feels when performing the movements
	Other		

This classification was based on the classification reported in Pollock *et al*., 2014, a Cochrane meta-analysis (additional Table 1 p. 356–361 and additional Table 2 p. 361–363).

### Outcomes

For this meta-analysis, we studied both balance and postural control. Based on the International Classification of Functioning, Disability and Health (ICF), we considered balance as a level of activity reflecting functional abilities, and postural control as a body structure function reflecting both orientation and stabilization body [47]. Therefore, the primary outcomes were: balance measured by the Berg Balance Scale (BBS) or the Postural Assessment Scale for Stroke (PASS); postural deviation measured by the weight bearing asymmetry (WBA) on lower limbs or the mediolateral and anteroposterior position of the center of pressure (COP); and postural stability measured by all COP sway or limit of stability (LOS) parameters. BBS and PASS are two clinical scales measuring the functional abilities of patients for various balance skills [44] (**S1 Protocol)**. BBS is very widely used in studies and has metrological properties that make it a gold standard to assess balance in patients with stroke. We included studies that assessed postural control with postural deviation or stability measurement in sitting or standing static evaluation on a force plate with eyes open (EO) or closed (EC). Postural deviation included mediolateral postural deviation (measured by WBA and mediolateral position of COP) and anteroposterior postural deviation (measured by anteroposterior position of COP). Additionally, we included studies that measured WBA by means of another device than force plate, such as weight scale, if the measure was done in static position. The secondary outcome was autonomy measured by the Barthel Index, the Functional Independence Measure, the Activities of Daily Living or the Instrumental Activities of Daily Living scales.

### Data sources

Medline, Elsevier databases (*i*.*e*. EMBASE until October 2015, SCOPUS thereafter), Cochrane Central Register of Controlled Trials, PEDro, Pascal, and Francis databases were searched from inception until January 14, 2019 (S1 Table and **S1 Protocol** for search strategy [44]). Scopus was replaced by Embase because we had no longer access to it. These two databases are both produced by Elsevier and the recall by Scopus of references found by Embase was optimal or suboptimal that is considered as acceptable [48]. Unpublished studies, conferences, and presentations were searched without language restriction.

### Study selection

Based on eligibility criteria, two authors (AH, JDM) independently selected the studies. The judgment of three other authors (IB, FG, GR) was used to resolve potential conflicts [44] (**S1 Protocol)**. No language restriction was applied.

### Data extraction

Two authors (AH and JDM) independently extracted data; potential conflicts were resolved with the help of three other authors (IB, FG, GR). In case of unclear or missing data, we contacted the authors of the respective studies. Extracted data included: study design, participant characteristics, risk of bias, PT characteristics, and outcomes (**S1 Protocol** [44]). All outcomes were statistically treated as continuous measures. We extracted the mean value, the standard deviation (SD), and the number of participants to the outcome measurements in each intervention group. The change-from-baseline was used to determine the outcome. Due to poor, variable or incomplete reporting of change score, different methods were used to obtain the mean and SD of changes when necessary. The most parsimonious statistical treatment was preferred. Finally, when only mean and SD values for before and after intervention assessments were given, SD was imputed by using a correlation coefficient with respect to the most conservative approach.

### Risk of bias assessment

Two authors (AH and JDM) independently assessed the seven items of the risk of bias tool from the Cochrane Collaboration [27] for each study, and used the Grades of Recommendation, Assessment, Development, and Evaluations (GRADE) as reported in Cochrane Handbook [27] to assess the overall quality of evidence of this meta-analysis. The judgment of two other authors (MC, FG) was used to resolve potential conflicts.

### Data synthesis and analysis

Statistical analyses were performed using R (R Foundation for Statistical Computing, Vienna, Austria; available in http://www.R-project.org/; version 3.5.2). Concordance between authors for the selection of studies was estimated using the Cohen’s Kappa coefficient and the recommendations of Landis and Kock [49]. Post-intervention effects were investigated by calculating the change from baseline to the immediate post-intervention assessment, and persisting effects by computing the change from baseline to the last follow-up assessment. These changes were compared between groups. The inverse-variance method was applied to summarize effects across studies. The summary effect estimate for all scales was calculated as the mean difference and its 95% confidence interval (95%CI). The estimate for outcomes was calculated as the standardized mean difference (SMD) and its 95%CI [44] because each outcome pooled several scales. We used Hedges’g to calculate SMD. The fixed-effect model was applied by default and the random-effect model was used in case of substantial heterogeneity (I^2^≥50%) [44] (**S1 Protocol)**. We summarize effects of crossover trials by following the recommendations of Cochrane Handbook (chapter 16.4) [27]. When several scales were available for the same outcome and to prevent any overweight of a study in a same SMD analysis, we ranked the scales based on the frequency of use in all trials. We selected the most frequent scales.

We performed subgroup analyses according to categories of PT, time post-stroke, and location of stroke lesion. We also performed sensitivity analyses to explore the effects of methodological quality according to appraisal of risk of bias. We investigated publication bias by funnel plots, contour-enhanced funnel plot, and Egger tests [27,50,51]. If publication bias was suspected, we performed the trim and fill method as a form of sensitivity analysis of the pooled estimate [50,52,53]. To determine the impact of the dose of PT, effect estimates were correlated with parameters of duration of PT using meta-regression. We compared PT versus no treatment (NT) and PT versus sham treatment (ST) or usual care (UC), irrespective of the design of study used (direct design, *e*.*g*. A versus B; or “add-on” design, *e*.*g*. A+C versus B+C). ST was a placebo treatment or a control treatment different from a PT, such as music or relaxation, delivered using the same protocol as that used in the experimental group. UC was various and non-protocoled standard care freely defined by therapists according to practices at that time.

## Results

### Study selection

**Fig 1:**
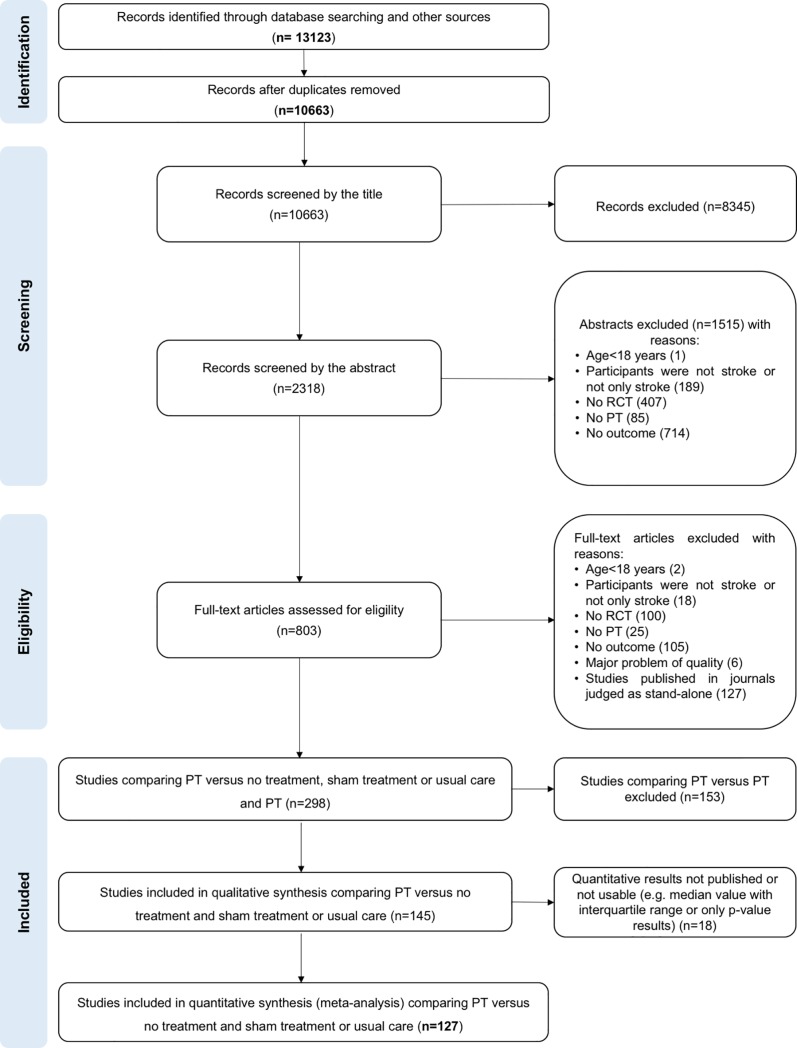
Flow-chart.

Among the 13,123 records identified, 10,663 single records were screened. For title screening, 8345 studies were excluded because they clearly did not address the topic of stroke or that did not include human subjects, or that the design mentioned in the title was explicitly different from a randomized controlled trial. The reasons for exclusion of records during the abstract screening then the full-text assessment are reported in the flow chart (Fig 1). For assessment of full-text eligibility, 56 studies were translated by co-authors (Chinese: n = 27, German: n = 6, Korean: n = 5, Spanish: n = 4, Russian: n = 3, Italian: n = 2, Persian: n = 2, Portuguese: n = 2, Turkish: n = 2, Japanese: n = 1, Norwegian: n = 1, Polish: n = 1). A total of 145 studies were selected (Fig 1 and S2 Table). The mean concordance between the two independent authors for the three steps of selection process, was substantial (kappa = 0.64). The authors of 130 of the 145 studies regarding unclear or missing data were contacted; answers were received for 20 studies.

### Study and participant characteristics

A total of 91 comparisons of PT versus NT in 76 studies and 81 comparisons of PT versus ST/UC in 70 studies were analyzed; 1 study was included in both comparisons. Among these 145 studies, 18 were of crossover design and 127 parallel group design; they included a total of 5912 participants (mean: 40.8, SD: 42.9, range: 7–408). Weighted participant age was 60.8 years (SD: 44.3, range: 46.9–78.5; S3 Table).

### Risk of bias

Risk of bias was low for random sequence generation in 55% of studies, for allocation concealment in 13% of studies, for blinding outcome assessment in 44% of studies, for incomplete outcome data in 17% of studies, and for selective reporting in 16% of studies. Most studies had a high or unclear risk of bias for blinding of patients and therapists (99%) but a low risk for other bias (92%; S1 Fig and S4 Table). Funnel plots and Egger tests found no evidence of publication bias for PT versus NT on balance, mediolateral postural deviation EO, postural stability EO, or autonomy; whereas for comparison PT versus ST/UC, there was a potential publication bias on balance (post-intervention effects and persisting effects), postural stability EO (post-intervention effects), and autonomy (post-intervention effects and persisting effects). The number of unpublished studies estimated by the trim and fill method was 0 for post-intervention effects on postural stability EO and post-intervention effects on autonomy, 1 for post-intervention effects on balance, 4 for persisting effects on autonomy, and 9 for persisting effects on balance (S2 Fig and S5 Table).

### Physical therapy

Functional task-training (including balance training) and assistive devices were the most common categories of PT that were compared to NT. Functional task-training, musculoskeletal interventions, and sensory interventions were the most common categories of PT that were compared to ST/UC (S6 Table).

Expressed as median values, participants received an additional 300 minutes dispensed in 12 sessions of 20 minutes for 3 weeks (PT versus NT). When PT was compared to ST/UC, treatment was delivered over 570 minutes, and dispensed in 16 sessions of 30 minutes for 5 weeks (S7 Table).

### Outcomes/Measures

BBS was the most common scale of balance used in studies for both post-intervention and persisting effects. For autonomy, the Barthel Index was the most frequent scale used. Sixty-four different parameters for WBA, LOS, and COP were identified. Fifty-one of these were assessed in ≤5 studies and the most common parameter was assessed in 23 studies (S8 Table).

### Effects

#### Balance

**Fig 2:**
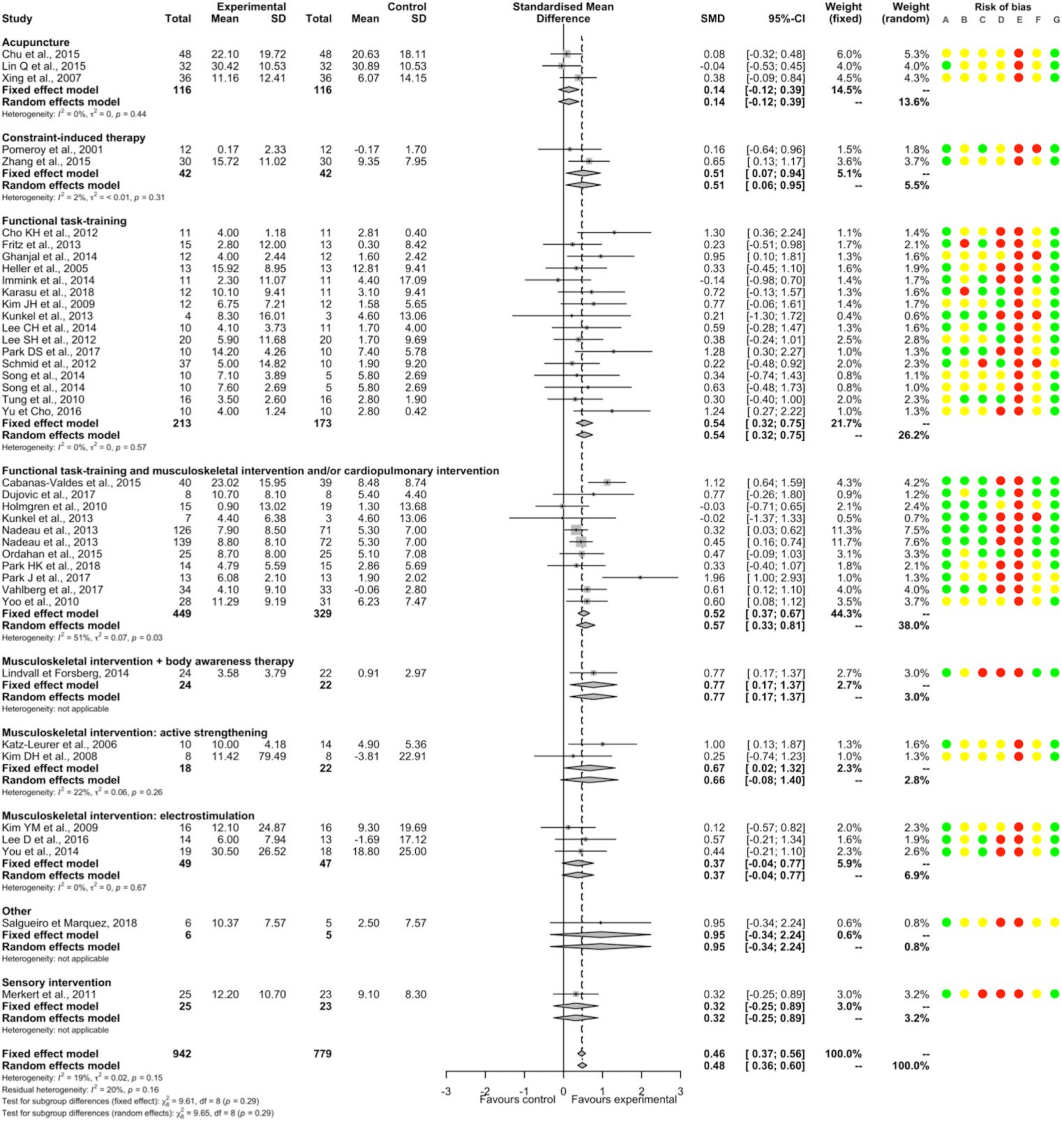
Forest plot of PT versus NT. **Outcome: Balance, post-intervention effects.** Risk of bias: A, Random sequence generation; B, Allocation concealment; C, Blinding of outcome assessment; D, Incomplete outcome data; E, Blinding of participants and therapists; F, Selective reporting; G, Other bias. Risk of bias: green color corresponds to low risk, yellow color unclear risk, and red color high risk. Abbreviations: CI, Confidence interval; SD, Standard deviation; SMD, Standardized mean difference.

PT had a significantly beneficial post-intervention effect compared to NT (37 studies, 1721 participants, SMD 0.46, 95%CI [0.37; 0.56]) with low heterogeneity (I^2^ = 19%). Significant positive SMDs were found for constraint-induced therapy, functional task-training, functional task-training associated with musculoskeletal intervention and/or cardiopulmonary intervention, musculoskeletal intervention with body awareness therapy, and musculoskeletal intervention by active strengthening; and non-significant SMDs for acupuncture, musculoskeletal intervention by electrostimulation, sensory interventions and other intervention (no significant between-subgroup difference, p = 0.29; Fig 2). There were significant positive SMDs for acute-subacute stroke patient and chronic stroke patient subgroups without significant between-subgroup difference (p = 0.50; S9 Table). A significant positive SMD was found for a subgroup of studies that included only supratentorial stroke patients (S10 Table). There was no significant meta-regression with duration of PT. For each item of bias, removing the studies judged as having high or unclear risk found a similar direction of SMDs favoring PT (except for blinding of patients and therapists because all studies showed a high or unclear risk; S3 Fig).

**Fig 3:**
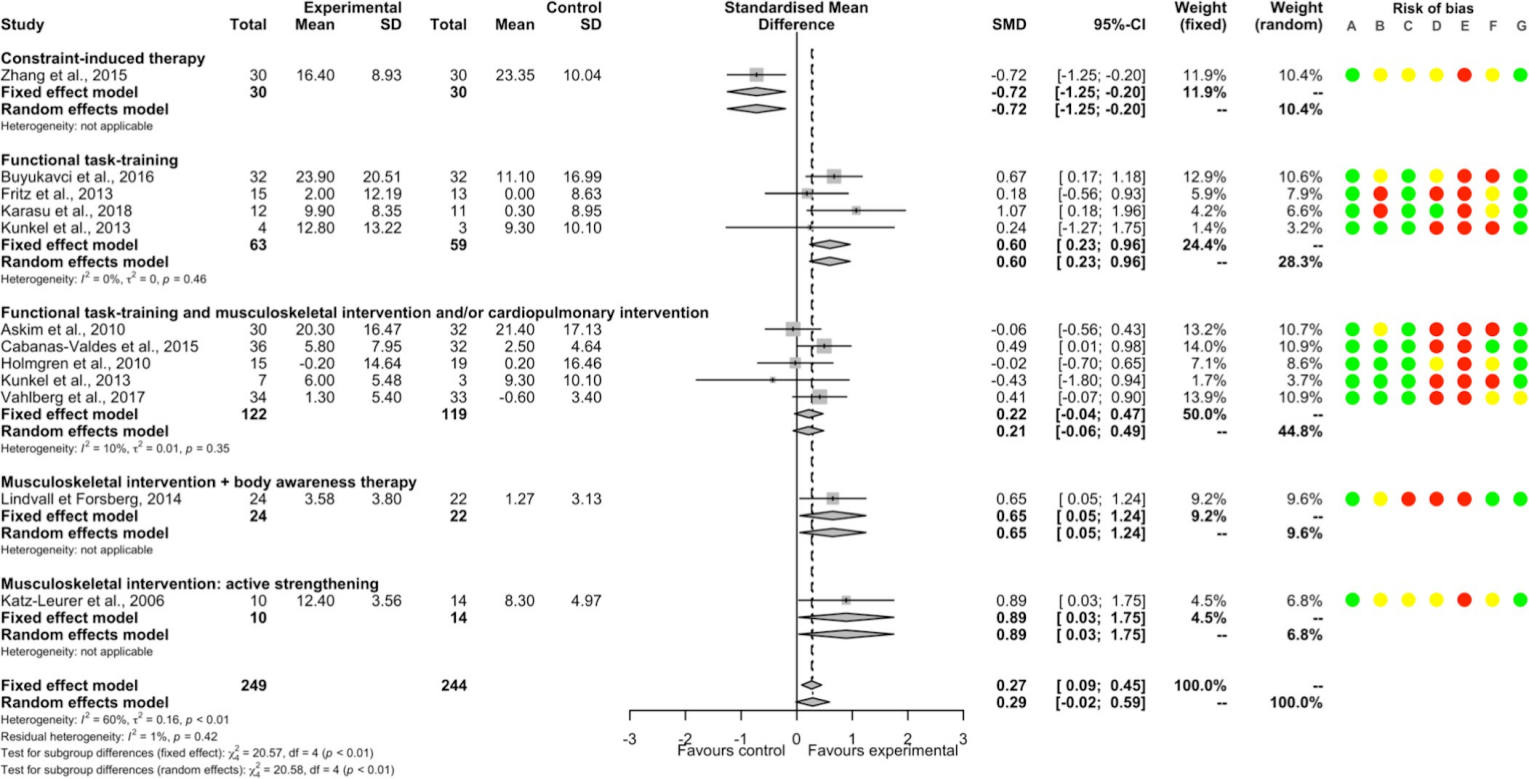
Forest plot of PT versus NT. **Outcome: Balance, persisting effects.** Risk of bias: A, Random sequence generation; B, Allocation concealment; C, Blinding of outcome assessment; D, Incomplete outcome data; E, Blinding of participants and therapists; F, Selective reporting; G, Other bias. Risk of bias: green color corresponds to low risk, yellow color unclear risk, and red color high risk. Abbreviations: CI, Confidence interval; SD, Standard deviation; SMD, Standardized mean difference.

There was a non-significant SMD between PT and NT for persisting effects (11 studies, 493 participants, SMD 0.29, 95%CI [-0.02; 0.59]) with substantial heterogeneity (I^2^ = 60%). A significant between-subgroup difference was found (p<0.01); there were significant positive SMDs for subgroups of functional task-training, of musculoskeletal intervention with body awareness therapy and of musculoskeletal intervention by active strengthening; a significant negative SMD for the subgroup of constraint-induced therapy; and non-significant SMDs for the subgroup of functional task-training associated with musculoskeletal intervention and/or cardiopulmonary intervention (Fig 3). There was a significant positive SMD for the subgroup of chronic stroke patients and a non-significant SMD for the subgroup of acute-subacute stroke patients, without significant difference between subgroups (p = 0.64; S9 Table).

**Fig 4:**
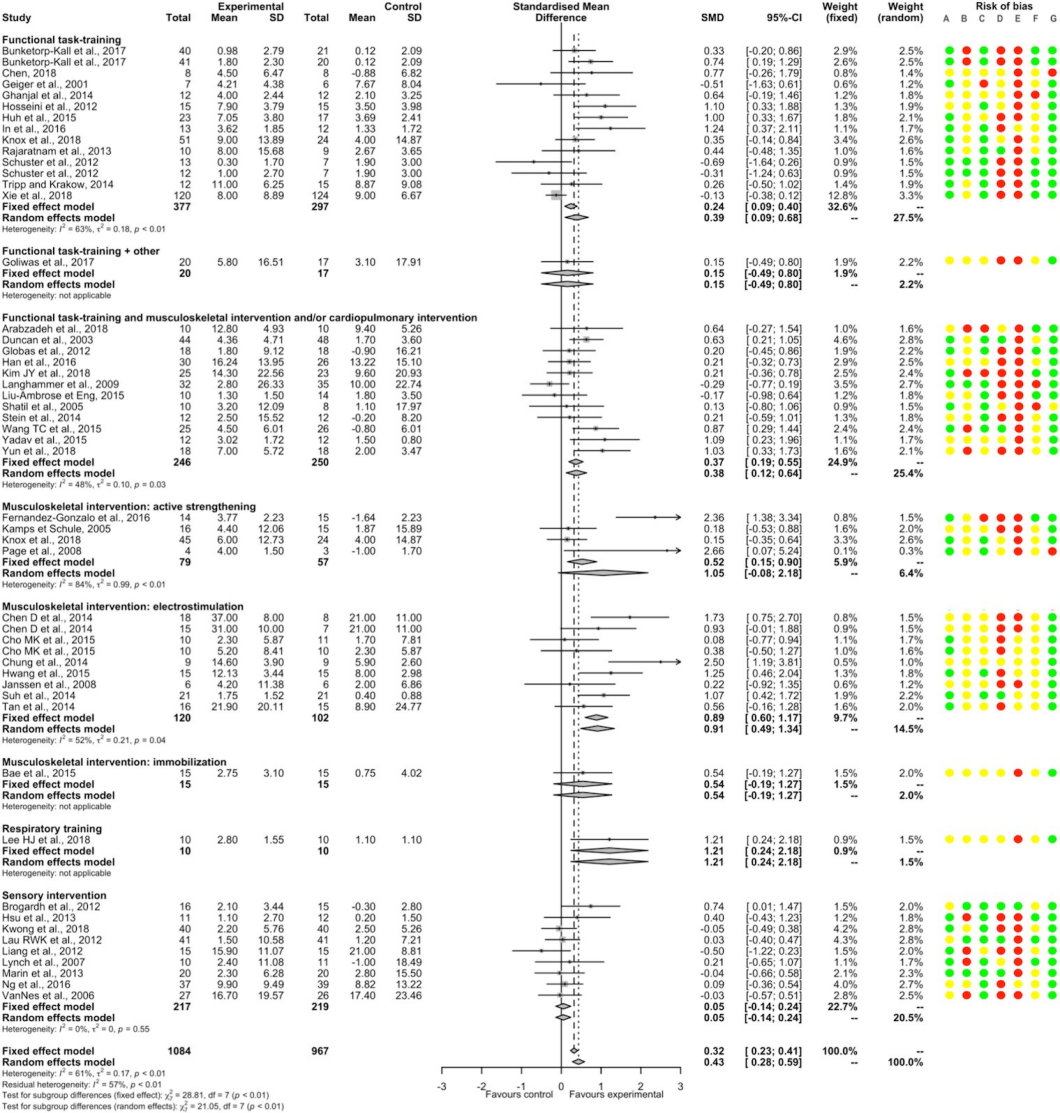
Forest plot of PT versus ST/UC. **Outcome: Balance, post-intervention effects.** Risk of bias: A, Random sequence generation; B, Allocation concealment; C, Blinding of outcome assessment; D, Incomplete outcome data; E, Blinding of participants and therapists; F, Selective reporting; G, Other bias. Risk of bias: green color corresponds to low risk, yellow color unclear risk, and red color high risk. Abbreviations: CI, Confidence interval; SD, Standard deviation; SMD, Standardized mean difference.

PT had a significantly beneficial post-intervention effect compared to ST/UC (46 studies, 2051 participants, SMD 0.43, 95%CI [0.28; 0.59]) with substantial heterogeneity (I^2^ = 61%). There was a significant between-subgroup difference (p<0.01). There were significant positive SMDs for functional task-training alone or associated with musculoskeletal intervention and/or cardiopulmonary intervention, musculoskeletal intervention by electrostimulation, and respiratory training; and non-significant SMDs for musculoskeletal intervention by active strengthening or by immobilization and sensory interventions (Fig 4). There were significant positive SMDs for acute-subacute stroke patient and chronic stroke patient subgroups, without between significant between-subgroup difference (p = 0.16; S9 Table). A non-significant SMD was found for a subgroup of studies that included only supratentorial stroke patients (S10 Table). There was a significant negative meta-regression between SMD and the number of weeks of PT (p = 0.04; S4 Fig). Removing all studies judged as having high or unclear risk for random sequence generation, blinding of participants and therapists, blinding of outcome assessment, incomplete outcome data, and other bias found a similar direction of SMDs favoring PT, whereas for allocation concealment and selective reporting SMDs became non-significant (S5 Fig). The summary post-intervention effect estimate adjusted for the potential publication bias concerning balance for the comparison PT versus ST/UC was similar and still in favor of PT (1 missing point, SMD 0.43, 95%CI [0.27; 0.58], I^2^ = 61% according to the trim and fill method).

**Fig 5:**
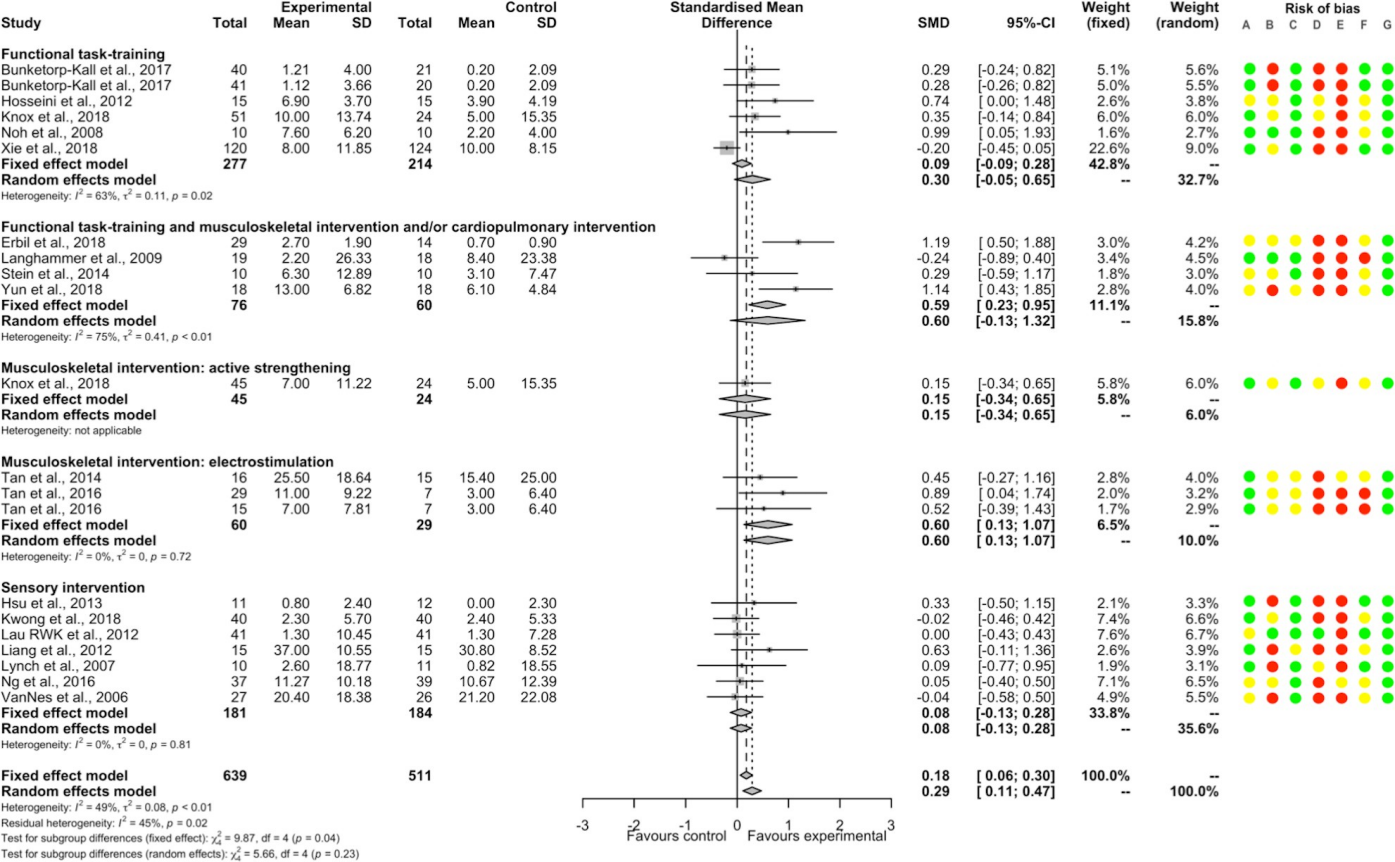
Forest plot of PT versus ST/UC. **Outcome: Balance, persisting effects.** Risk of bias: A, Random sequence generation; B, Allocation concealment; C, Blinding of outcome assessment; D, Incomplete outcome data; E, Blinding of participants and therapists; F, Selective reporting; G, Other bias. Risk of bias: green color corresponds to low risk, yellow color unclear risk, and red color high risk. Abbreviations: CI, Confidence interval; SD, Standard deviation; SMD, Standardized mean difference.

PT had a significantly beneficial persisting effect compared to ST/UC (18 studies, 1150 participants, SMD 0.18, 95%CI [0.06; 0.30]) with moderate heterogeneity (I^2^ = 49%). A significant positive SMD was only found for the subgroup of musculoskeletal intervention by electrostimulation (Fig 5). There were significant positive SMDs for acute-subacute stroke patient and chronic stroke patient subgroups (S9 Table); and a non-significant SMD for a subgroup of studies that included only supratentorial stroke patients (S10 Table). The summary persisting effect estimate adjusted for the potential publication bias on balance for the comparison PT versus ST/UC became non-significant (9 missing points, SMD 0.03, 95%CI [-0.17; 0.23], I^2^ = 67%, according to the trim and fill method).

#### Mediolateral postural deviation

**Fig 6:**
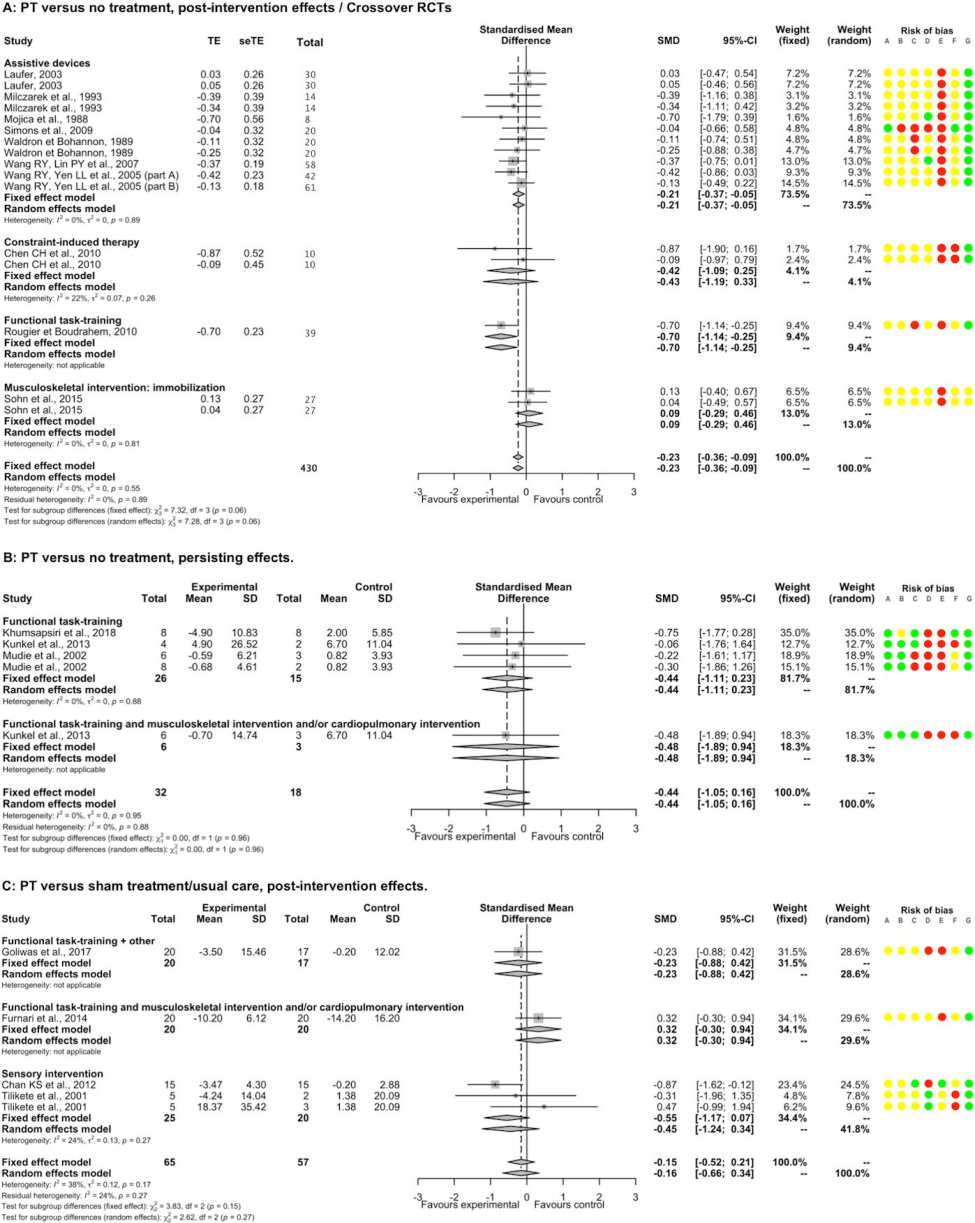
Forest plot of PT versus NT and versus ST/UC. **Outcome: Mediolateral postural deviation EO.** Risk of bias: A, Random sequence generation; B, Allocation concealment; C, Blinding of outcome assessment; D, Incomplete outcome data; E, Blinding of participants and therapists; F, Selective reporting; G, Other bias. Risk of bias: green color corresponds to low risk, yellow color unclear risk, and red color high risk. Abbreviations: CI, Confidence interval; SD, Standard deviation; SMD, Standardized mean difference.

PT had a significantly beneficial post-intervention effect EO compared to NT (11 studies, 329 participants, SMD -0.23, 95%CI [-0.36; -0.09]) without heterogeneity (I^2^ = 0%). There were significant negative SMDs for assistive device and functional task-training; and a non-significant SMD for constraint-induced therapy and musculoskeletal intervention by immobilization; with a significant between-subgroup difference (p = 0.06; Fig 6). There was a significant negative SMD for the subgroup of acute-subacute stroke patients and a non-significant SMD for the subgroup of chronic stroke patients (1 study), without between significant between-subgroup difference (p = 0.34). There was a non-significant SMD for a subgroup of studies that included only supratentorial stroke patients (S9 and S10 Tables). We found no significant meta-regression with duration of PT. Removing all studies judged as having high or unclear risk for incomplete outcome data and other bias showed a similar direction of SMDs favoring PT, whereas for random sequence generation and selective reporting, SMDs became non-significant (S6 Fig).

A non-significant SMD was found between PT and NT for persisting effects EO (3 studies, 50 participants, SMD -0.44, 95%CI [-1.05; 0.16]), without heterogeneity (I^2^ = 0%) and significant difference between categories of PT (p = 0.96; Fig 6).

A non-significant SMD was found between PT and ST/UC for post-intervention effects EO (4 studies, 122 participants, SMD -0.15, 95%CI [-0.52; 0.21]) with moderate heterogeneity (I^2^ = 38%). All category of PTs such as functional task-training associated with musculoskeletal intervention and/or cardiopulmonary intervention, or with another intervention and sensory interventions had non-significant SMDs (Fig 6). There was a significant negative SMD for chronic stroke patients subgroup and a non-significant SMD for acute-subacute stroke patients subgroup (1 study), without significant between-subgroup difference (p = 0.11; S9 Table). A non-significant SMD was found for a subgroup of study that included only supratentorial stroke patients (1 study; S10 Table). There was a positive meta-regression between SMD and the overall duration of PT (5 studies, p = 0.052). Removing all studies judged as having high or unclear risk for blinding of outcome assessment and blinding of patients and therapists changed the direction of SMDs favoring PT, whereas for incomplete outcome data and other bias, SMDs still were non-significant (S7 Fig). No study investigated persisting effects of PT compared to ST/UC.

#### Postural stability

**Fig 7:**
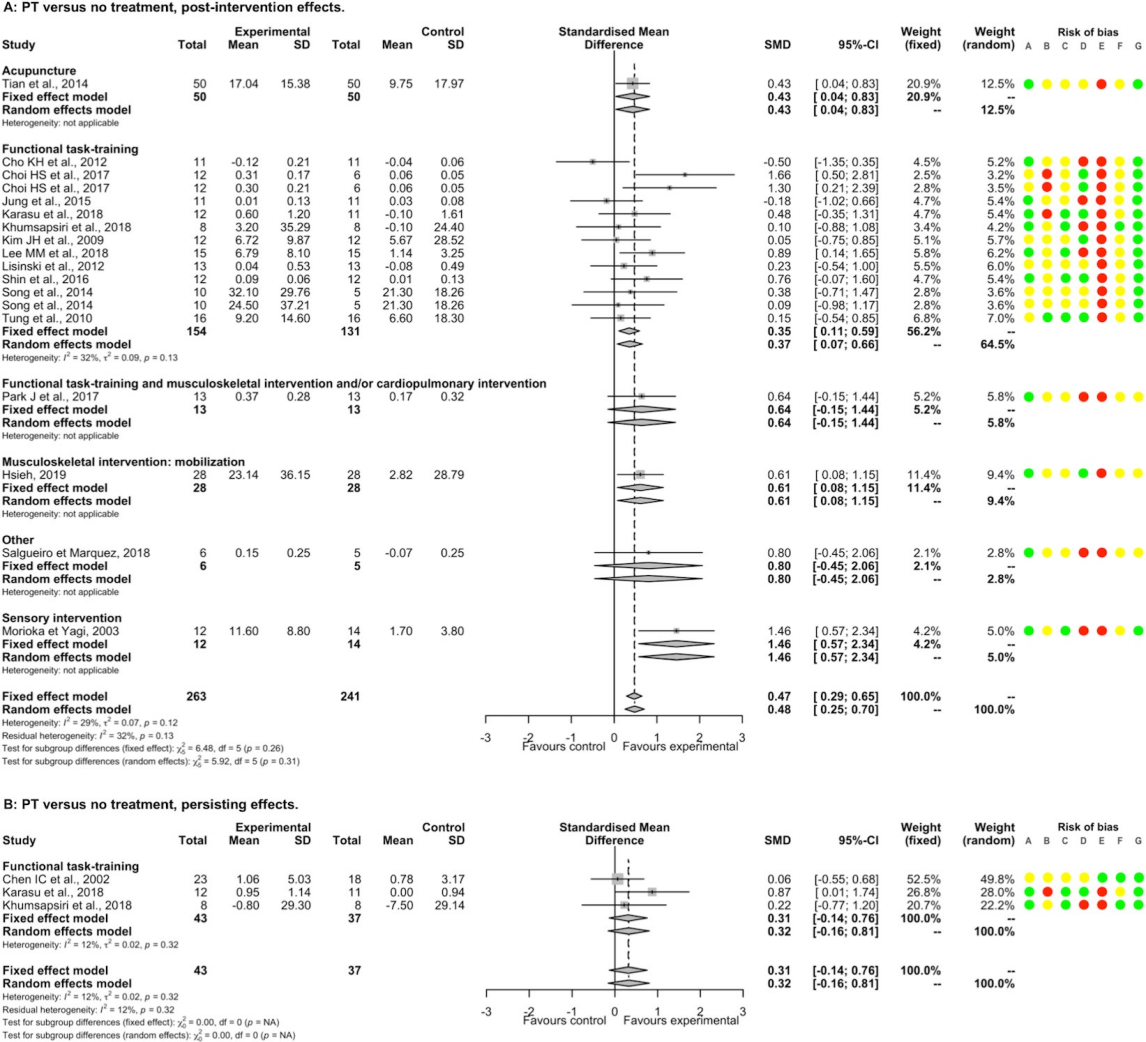
Forest plot of PT versus NT. **Outcome: Postural stability EO.** Risk of bias: A, Random sequence generation; B, Allocation concealment; C, Blinding of outcome assessment; D, Incomplete outcome data; E, Blinding of participants and therapists; F, Selective reporting; G, Other bias. Risk of bias: green color corresponds to low risk, yellow color unclear risk, and red color high risk. Abbreviations: CI, Confidence interval; SD, Standard deviation; SMD, Standardized mean difference.

PT had a significantly beneficial post-intervention effect EO compared to NT (16 studies, 504 participants, SMD 0.47, 95%CI [0.29; 0.65]) with low heterogeneity (I^2^ = 29%). There was a significant positive SMDs for acupuncture, functional task-training, musculoskeletal intervention by mobilization, and sensory interventions; and non-significant SMDs for functional task-training associated with musculoskeletal intervention and/or cardiopulmonary intervention and for other interventions; without significant between-subgroup difference (p = 0.26; Fig 7). There was a significant positive SMD for acute-subacute stroke patients subgroup, and a non-significant SMD for chronic stroke patients subgroup, without significant between-subgroup difference (p = 1.00; S9 Table). A non-significant SMD was found for a subgroup of study that included only supratentorial stroke patients (S10 Table). There was no significant meta-regression with duration of PT. Removing all studies judged as having high or unclear risk for random sequence generation, blinding of outcome assessment, incomplete outcome data and other bias showed a similar direction of SMD favoring PT, whereas for concealment allocation and selective reporting, SMDs became non-significant (S8 Fig). For EC, PT had a significantly beneficial post-intervention effect compared to NT (9 studies, 229 participants, SMD 0.34, 95%CI [0.08; 0.61]) without heterogeneity (I^2^ = 0%; S9 Fig).

**Fig 8:**
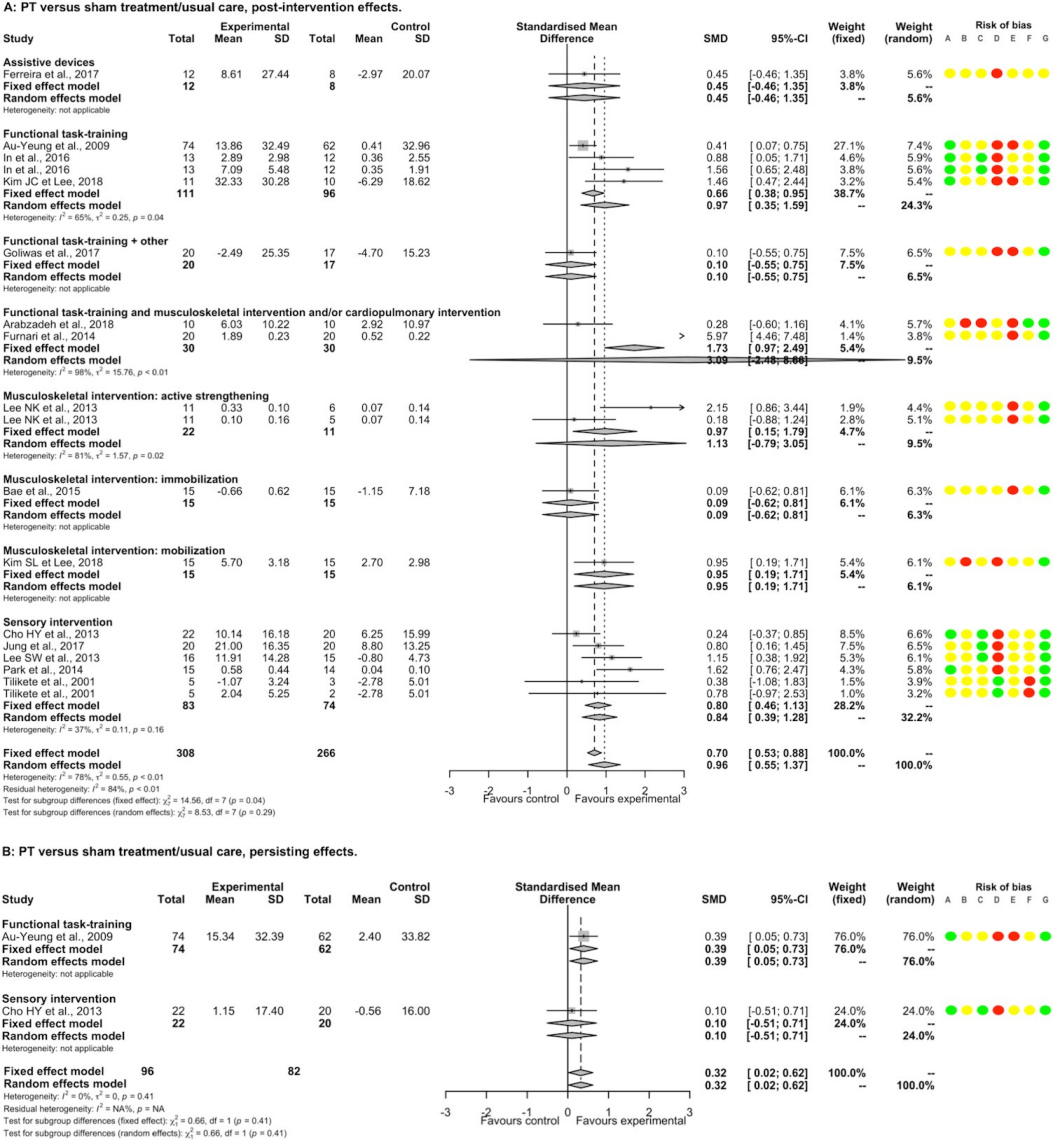
Forest plot of PT versus ST/UC. **Outcome: Postural stability EO.** Risk of bias: A, Random sequence generation; B, Allocation concealment; C, Blinding of outcome assessment; D, Incomplete outcome data; E, Blinding of participants and therapists; F, Selective reporting; G, Other bias. Risk of bias: green color corresponds to low risk, yellow color unclear risk, and red color high risk. Abbreviations: CI, Confidence interval; SD, Standard deviation; SMD, Standardized mean difference.

PT had a significantly beneficial post-intervention effect EO compared to ST/UC (15 studies, 574 participants, SMD 0.96, 95%CI [0.55; 1.37]) with substantial heterogeneity (I^2^ = 78%). There were significant positive SMDs for functional task-training, musculoskeletal intervention by mobilization, and sensory interventions; and non-significant SMDs for assistive devices, functional task-training associated with musculoskeletal intervention and/or cardiopulmonary intervention or with another intervention, musculoskeletal intervention by active strengthening and musculoskeletal intervention by immobilization; without significant between-subgroup difference (p = 0.29; Fig 8). There was a significant positive SMD for chronic stroke patients subgroup and a non-significant SMD for acute-subacute stroke patients subgroup, with a significant between-subgroup difference (p = 0.09; S9 Table). We found a non-significant SMD for a subgroup of study that included only supratentorial stroke patients (1 study; S10 Table). There was a significant positive meta-regression between post-intervention effects and the overall duration of PT for the subgroup of sensory interventions (S4 Fig). Removing all studies judged as having high or unclear risk for random sequence generation, blinding of outcome assessment, and other bias showed a similar direction of SMD favoring PT, whereas for incomplete outcome data, SMD became non-significant. All studies showed a high or unclear risk of bias for concealment allocation and for blinding of patients and therapists (S10 Fig). The summary post-intervention effect estimate adjusted on the potential publication bias concerning postural stability EO for the comparison PT versus ST/UC was not changed (0 missing point according to the trim and fill method). Considering the atypical treatment effect of a study, Furnari *et al*. (2014) [54] compared to other studies, we performed a sensitivity analysis that found a summary SMD still in favor of PT (14 studies, 534 participants, SMD 0.72, 95%CI [0.45; 0.98], I^2^ = 46%). For EC, there was a significantly beneficial post-intervention effect of PT (10 studies, 352 participants, SMD 1.02, 95%CI [0.38; 1.67]) with substantial heterogeneity (I^2^ = 86%; S9 Fig). A sensitivity analysis removing one study, Furnari *et al*. (2014) [54], found a summary SMD still in favor of PT (SMD 0.62, 95%CI [0.25; 0.98], I^2^ = 57%). For either EO or EC, the persisting effects of PT compared to NT and these of PT compared to ST/UC are reported in Figs 7 and 8 and in S9 Fig.

#### Other outcomes and quality of evidence

The results of analyses on data extracted for autonomy are presented in S11–S13 Figs and in S9 and S10 Tables. Moreover, the quality of evidence according to GRADE for all outcomes is presented in S11 Table.

## Discussion

The present study found that the overall post-intervention effects were in favor of PT compared to NT for balance, mediolateral postural deviation EO, and postural stability (EO or EC), and compared to ST/UC for balance and postural stability (EO or EC) after stroke. Few categories of PT were more effective than NT in improving balance after stroke immediately after intervention. However, caution should be taken when interpreting these results owing to a small number of studies, participants, or substantial heterogeneity within subgroups. The findings therefore only support that functional task-training alone had a beneficial effect in improving balance compared to NT, owing to the absence of heterogeneity and a sufficient number of trials and participants. For instance, a beneficial effect for functional task-training associated with musculoskeletal intervention and/or cardiopulmonary intervention could be concluded if there was less heterogeneity. The present study also found limited evidence for the effect on balance compared to ST/UC in patients with stroke. The results allow only to conclude a beneficial effect immediately after intervention of functional task-training associated with musculoskeletal intervention and/or cardiopulmonary intervention but also the lack of efficacy of sensory interventions (such as vibration or tactile stimulation); substantial heterogeneity precludes conclusions as to the efficacy of functional task-training alone, or of musculoskeletal intervention by electrostimulation. Similarly, for persisting effects of PT, only the lack of efficacy for sensory interventions compared to ST/UC could be concluded. Another point of interest of the present study is the investigation of effects on postural control. We could conclude for post-intervention effects that assistive devices were more effective than NT in reducing mediolateral postural deviation EO, and that functional task-training alone and sensory interventions were, respectively, more effective than NT and ST/UC in increasing postural stability (either EO or EC).

Another point is that the beneficial effect of functional task-training alone on both balance, which is considered as activity according to the ICF, and postural stability (EO or EC), which is considered as body structure function according to the ICF, could suggest a transfer of learning from body structure function to activity level. Van Duijnhoven *et al*. (2016) [42] found ambiguous results for outcomes addressing body structure function and beneficial effects for balance (considered as activity) and suggested an optimization of compensatory balance strategies. Fewer studies (n = 36) were included in their meta-analysis than herein, which may go some way to explain this difference. Another important finding of the present meta-analysis is that with respect to comparisons made between PT and NT, those made between PT and ST/UC had smaller effect sizes and/or greater heterogeneity, which leads us to question whether or not there are specific effects of PT. It should be also noted that the reduction or the non-significance of SMD, in most cases, between post-intervention and persisting effects supports a short-term effect of PT.

Treatment modalities, such as the dose or the way to apply the PT, were very different between studies within a category of PT. This could explain part of the heterogeneity, and a better understanding of the mechanisms of action of the various categories of PT could improve the interpretation of any potential effect. More generally, the weak methodological quality of studies and the absence of significant effect when only studies at a low risk of bias were considered indicates that caution should be taken when interpreting the results. Therefore, implications of the present findings for clinical practice are limited. To address this issue, priority should be given to conduct trials of better methodological quality, especially regarding random sequence generation, allocation concealment [55], blinding outcome [56], and incomplete outcome data. It is also of note that data regarding the included population, therapies, and the size and precision of effects were often unclear or missing in the studies identified herein, and could be a source of the heterogeneity observed. This underlines the importance of the quality of reporting, as also identified by the Stroke Recovery and Rehabilitation Roundtable [57]. The sample size of studies was often too small, increasing the risk of overestimate the effect size [58], and the outcome measures used to assess effects were too wide. Larger, multicenter trials with standardization and consensus of outcome measures, as well as a rigorous control of potential bias, should therefore be conducted to provide more robust data.

## Conclusion

PT had beneficial overall post-intervention effects on balance and postural stability after stroke. Only functional task-training associated with musculoskeletal intervention and/or cardiopulmonary intervention and sensory interventions seemed to be immediately effective in improving balance or postural stability respectively. The heterogeneity of PT studied and the weak methodological quality of studies strongly limited the meaning and the confidence in findings.

## Supporting information

S1 Checklist PRISMA 2009 checklist. (DOC)

S1 Fig Risk of bias. (DOCX)

S2 Fig Funnel plots. (DOCX)

S3 Fig Forest plot of physical therapy versus no treatment. Outcome: Balance, post-intervention effects. Subgroup: risk of bias.
(DOCX)

S4 Fig Meta-regression of effects of PT according to duration of PT. (DOCX)

S5 Fig Forest plot of physical therapy versus sham treatment or usual care. Outcome: Balance, post-intervention effects. Subgroup: risk of bias.
(DOCX)

S6 Fig Forest plot of physical therapy versus no treatment. Outcome: Mediolateral postural deviation EO, post-intervention effects. Subgroup: risk of bias.
(DOCX)

S7 Fig Forest plot of physical therapy versus sham treatment or usual care. Outcome: Mediolateral postural deviation EO, post-intervention effects. Subgroup: risk of bias.
(DOCX)

S8 Fig Forest plot of physical therapy versus no treatment. Outcome: Postural stability EO, post-intervention effects. Subgroup: risk of bias.
(DOCX)

S9 Fig Forest plot of physical therapy. Outcome: Postural stability EC, post-intervention effects.
(DOCX)

S10 Fig Forest plot of physical therapy versus sham treatment or usual care. Outcome: Postural stability EO, post-intervention effects. Subgroup: risk of bias.
(DOCX)

S11 Fig Forest plot of physical therapy. Outcome: Autonomy. Subgroup: Categories of PT.
(DOCX)

S12 Fig Forest plot of physical therapy versus no treatment. Outcome: Autonomy, post-intervention effects. Subgroup: risk of bias.
(DOCX)

S13 Fig Forest plot of physical therapy versus sham treatment or usual care. Outcome: Autonomy, post-intervention effects. Subgroup: risk of bias.
(DOCX)

S1 Table Search strategy in databases. (DOCX)

S2 Table Identification of studies included in the systematic review and meta-analysis. (DOCX)

S3 Table Characteristics of studies and participants. (DOCX)

S4 Table Overall score of risk of bias and ethic statement for each study included. (DOCX)

S5 Table Results of Egger tests detecting bias of publication. (DOCX)

S6 Table Description of PT. (DOCX)

S7 Table Duration of PT. (DOCX)

S8 Table Outcome measures. (DOCX)

S9 Table Results of subgroup analyses according to the time since post-stroke. (DOCX)

S10 Table Results of subgroup analyses according to the location of stroke lesion. (DOCX)

S11 Table Summary of findings and quality of the evidence. (DOCX)

S1 Protocol Study protocol published. (PDF)
