# Supplementary material for: Limited evidence of physical therapy on balance after stroke: A systematic review and meta-analysis
Source: PLoS One. 2019 Aug 29;14(8):e0221700. doi: 10.1371/journal.pone.0221700 (PMC6715189; doi:10.1371/journal.pone.0221700)
Supplement: S1 Fig — (DOCX) [file pone.0221700.s002.docx]

**S1 Fig. Risk of bias**

**S1A Fig. Risk of bias graph: review authors' judgements about each risk of bias item presented as percentages across all included studies**

**
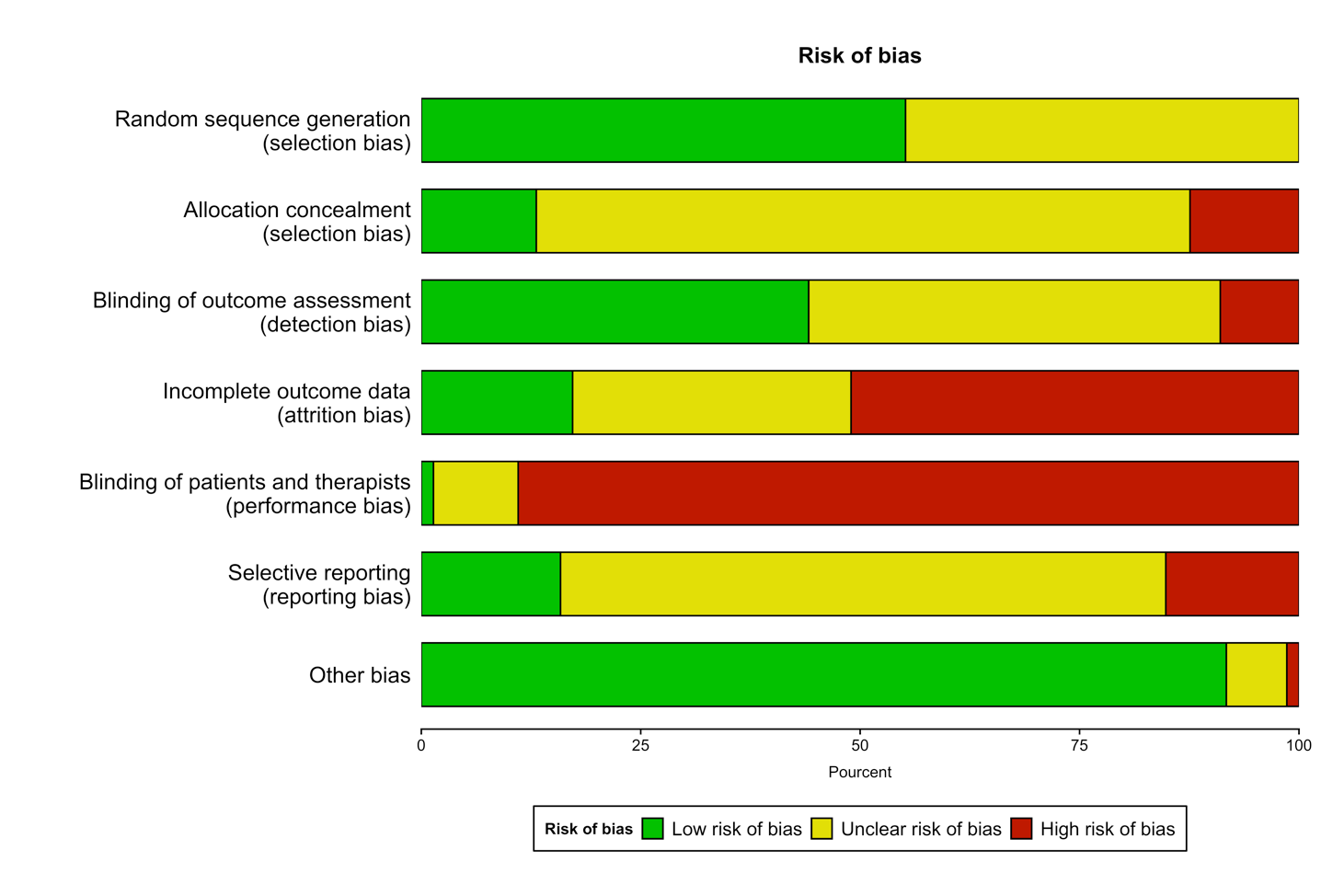
**

**S1B Fig. Risk of bias summary: review authors' judgements about each risk of bias item for each included study.**

Judgements about risk of bias: Green color means low risk, yellow color means unclear risk and red color means high risk.
