# Supplementary material for: Limited evidence of physical therapy on balance after stroke: A systematic review and meta-analysis
Source: PLoS One. 2019 Aug 29;14(8):e0221700. doi: 10.1371/journal.pone.0221700 (PMC6715189; doi:10.1371/journal.pone.0221700)
Supplement: S2 Fig — (DOCX) [file pone.0221700.s003.docx]

**S2 Fig. Funnel plots**

**S2A Fig. Funnel plots of comparison PT versus no treatment**


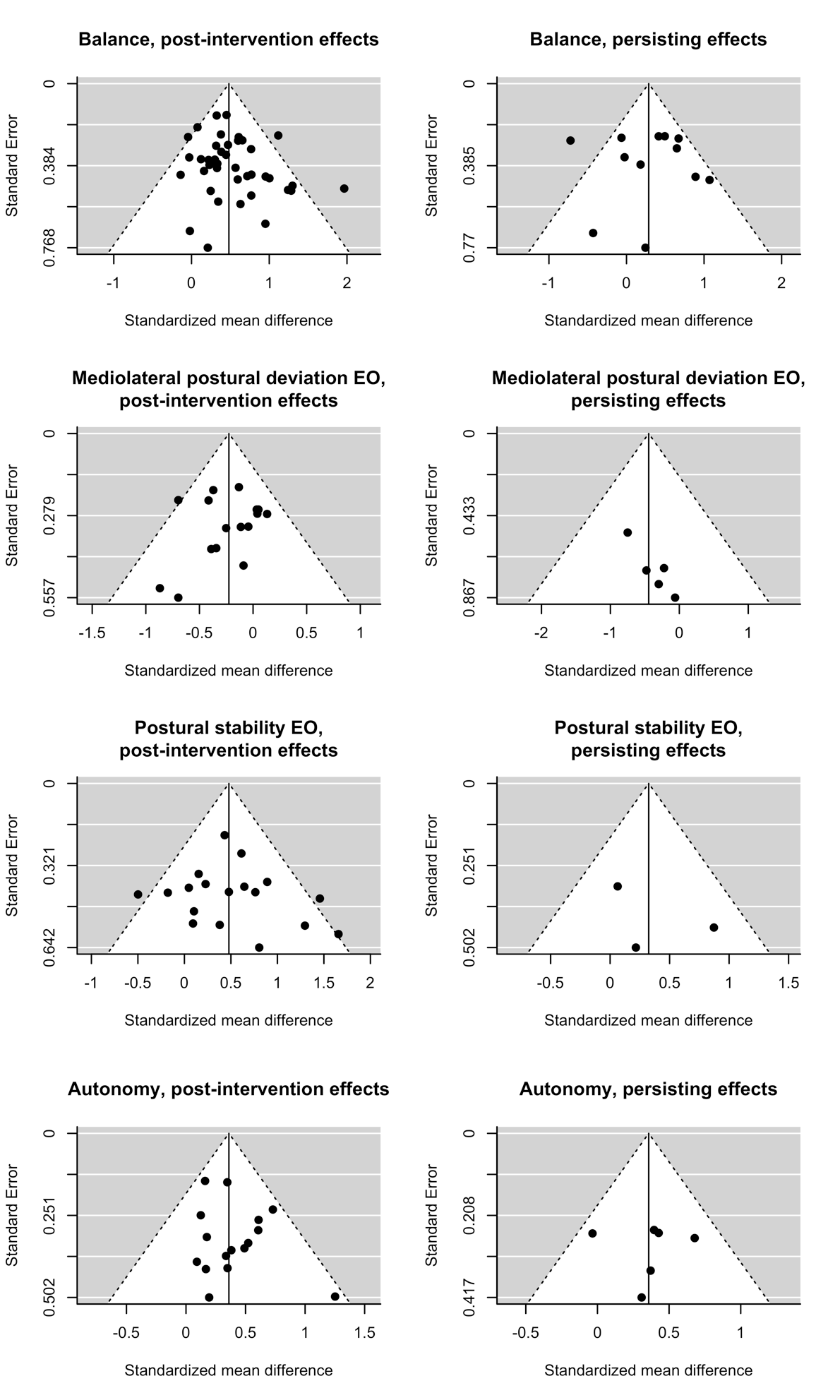


Legend: Dotted line: 95% confidence interval; black filled circle: study

Abbreviations: EO, eyes open

**S2B Fig. Funnel plots of comparison PT versus sham treatment/usual care**


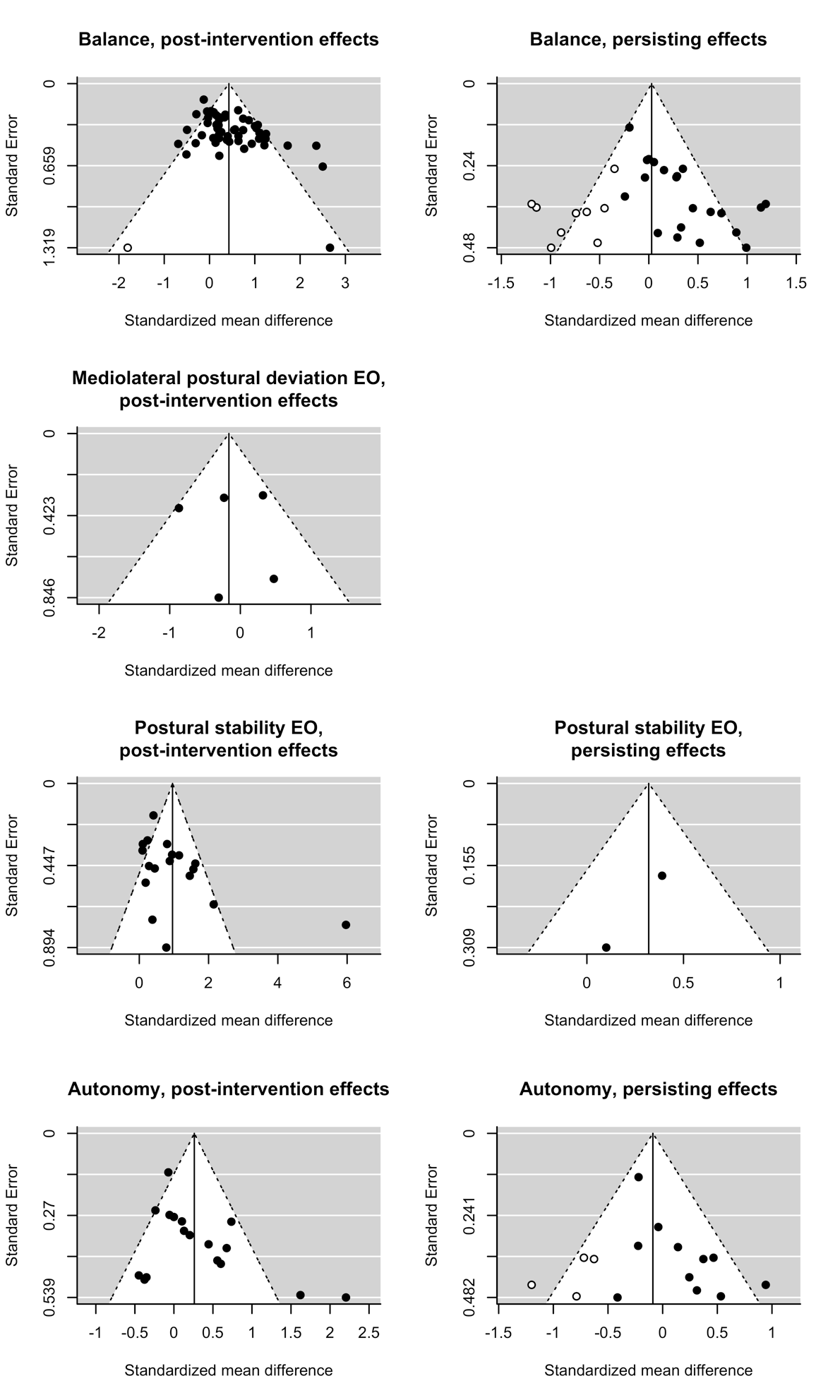


Legend: Dotted line: 95% confidence interval; black filled circle: study; white filled circle: “missing” study

Abbreviations: EO, eyes open
