# Supplementary material for: Limited evidence of physical therapy on balance after stroke: A systematic review and meta-analysis
Source: PLoS One. 2019 Aug 29;14(8):e0221700. doi: 10.1371/journal.pone.0221700 (PMC6715189; doi:10.1371/journal.pone.0221700)
Supplement: S4 Fig — (DOCX) [file pone.0221700.s005.docx]

**S4 Fig. Meta-regression of effects of PT according to duration of PT**

**S4A Fig. Meta-regression between the post-intervention effects and the number of weeks of PT. Comparison: PT compared to ST/UC. Outcome: Balance**

**
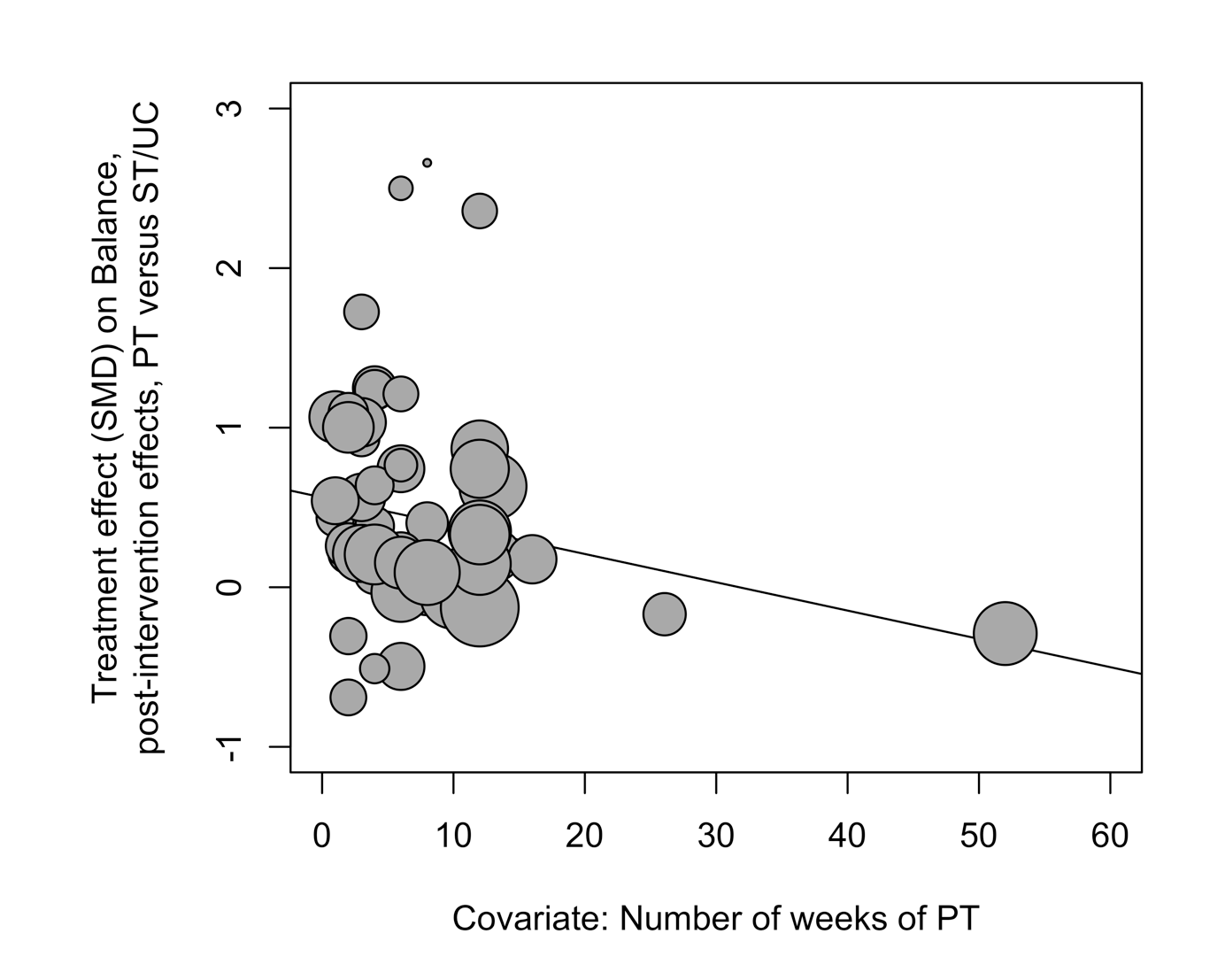
**


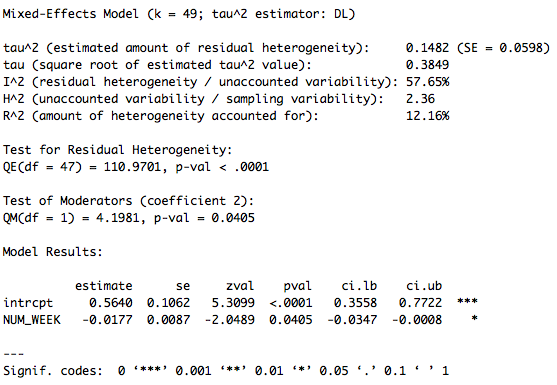


Abbreviations: PT, physical therapy; SMD, standardized mean difference; ST, sham treatment; UC, usual care

**S4B Fig. Meta-regression between the post-intervention effects and the overall duration of PT. Comparison: PT compared to ST/UC. Outcome: mediolateral postural deviation EO**


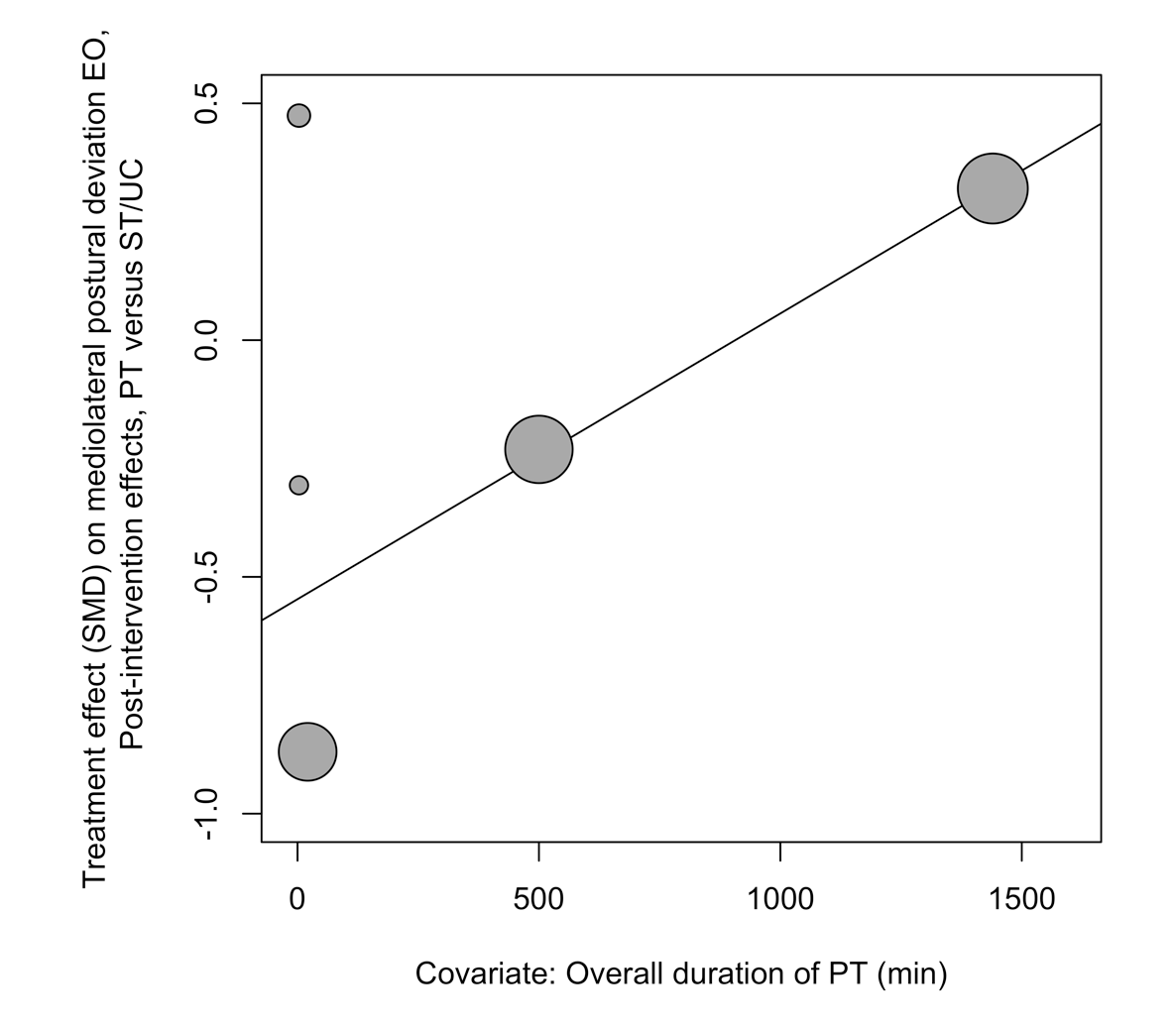


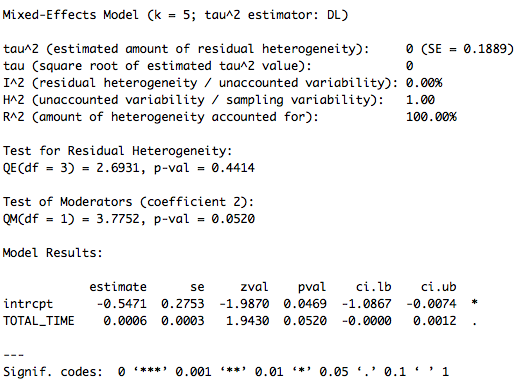


Abbreviations: EO, eyes open; PT, physical therapy; SMD, standardized mean difference; ST, sham treatment; UC, usual care

**S4C Fig. Meta-regression between the post-intervention effects and the overall duration of PT for the subgroup of sensory interventions. Comparison: PT compared to ST/UC. Outcome: postural stability EO**

**
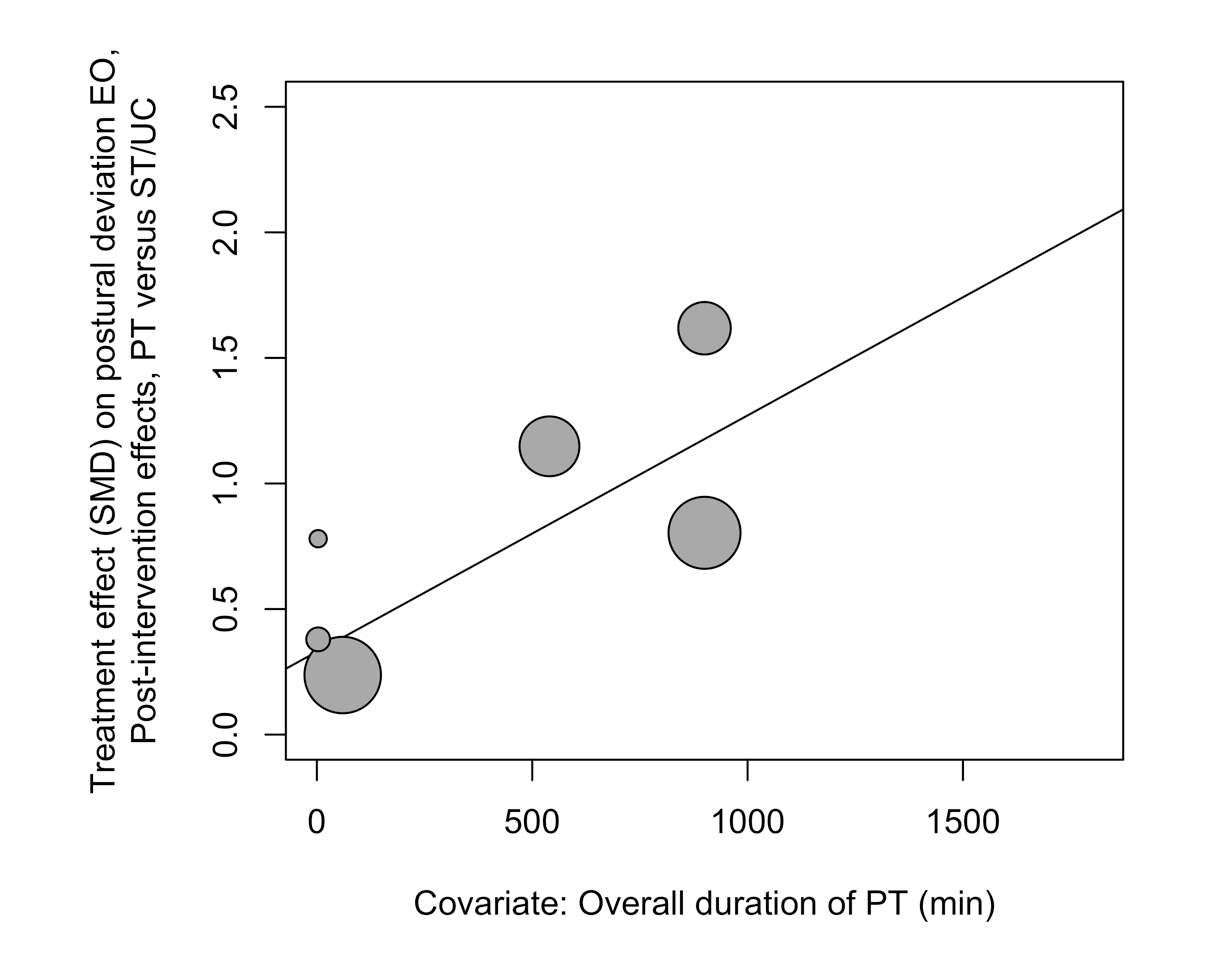
**

**
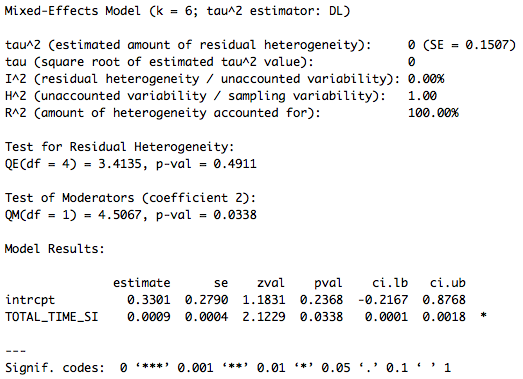
**

Abbreviations: EO, eyes open; PT, physical therapy; SMD, standardized mean difference; ST, sham treatment; UC, usual care
