# Supplementary material for: Limited evidence of physical therapy on balance after stroke: A systematic review and meta-analysis
Source: PLoS One. 2019 Aug 29;14(8):e0221700. doi: 10.1371/journal.pone.0221700 (PMC6715189; doi:10.1371/journal.pone.0221700)
Supplement: S5 Fig — Outcome: Balance, post-intervention effects. Subgroup: risk of bias. (DOCX) [file pone.0221700.s006.docx]

**S5 Fig. Forest plot of physical therapy versus sham treatment/usual care. Outcome: Balance, post-intervention effects. Subgroup: risk of bias**

**
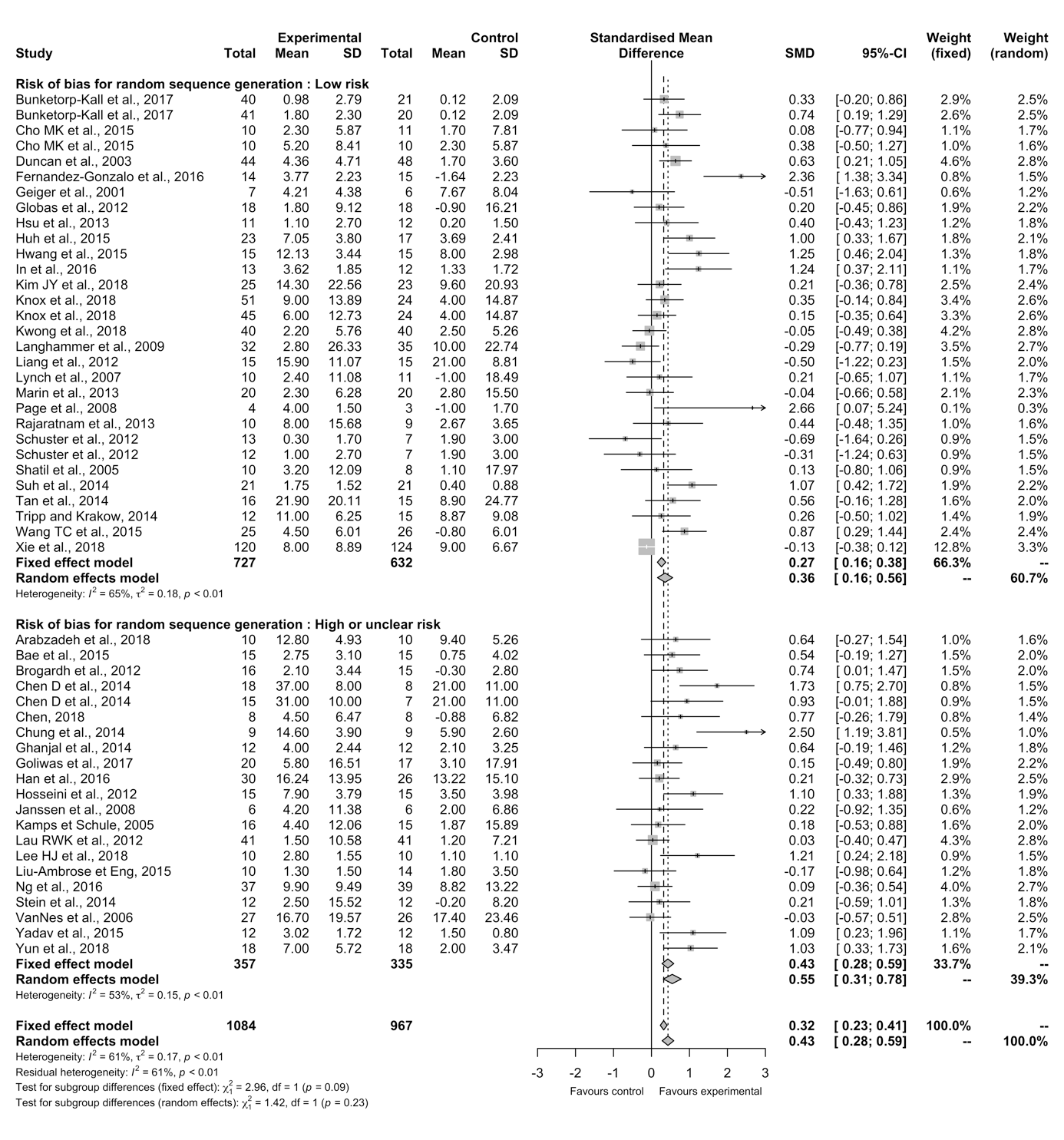
**

**
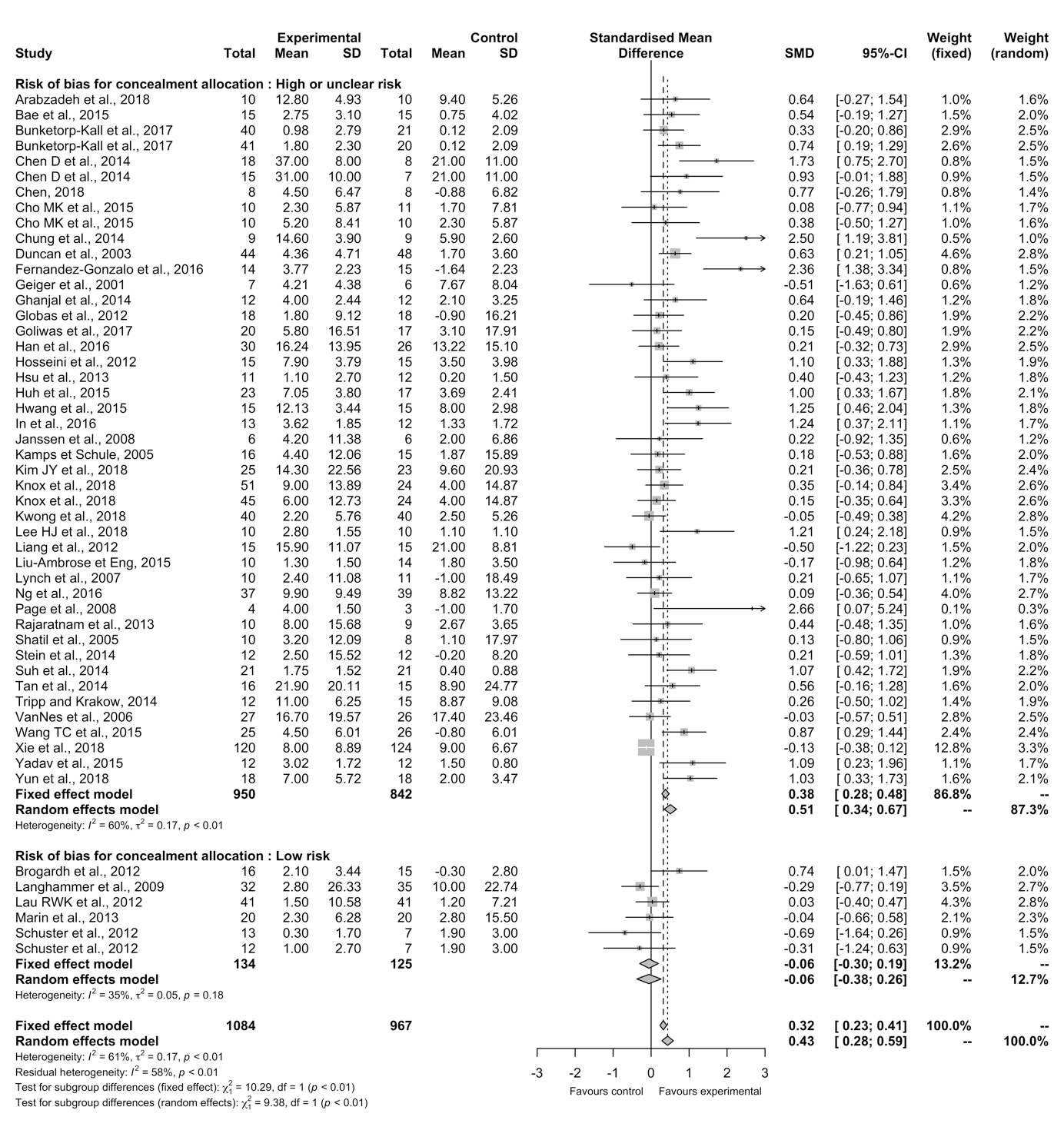

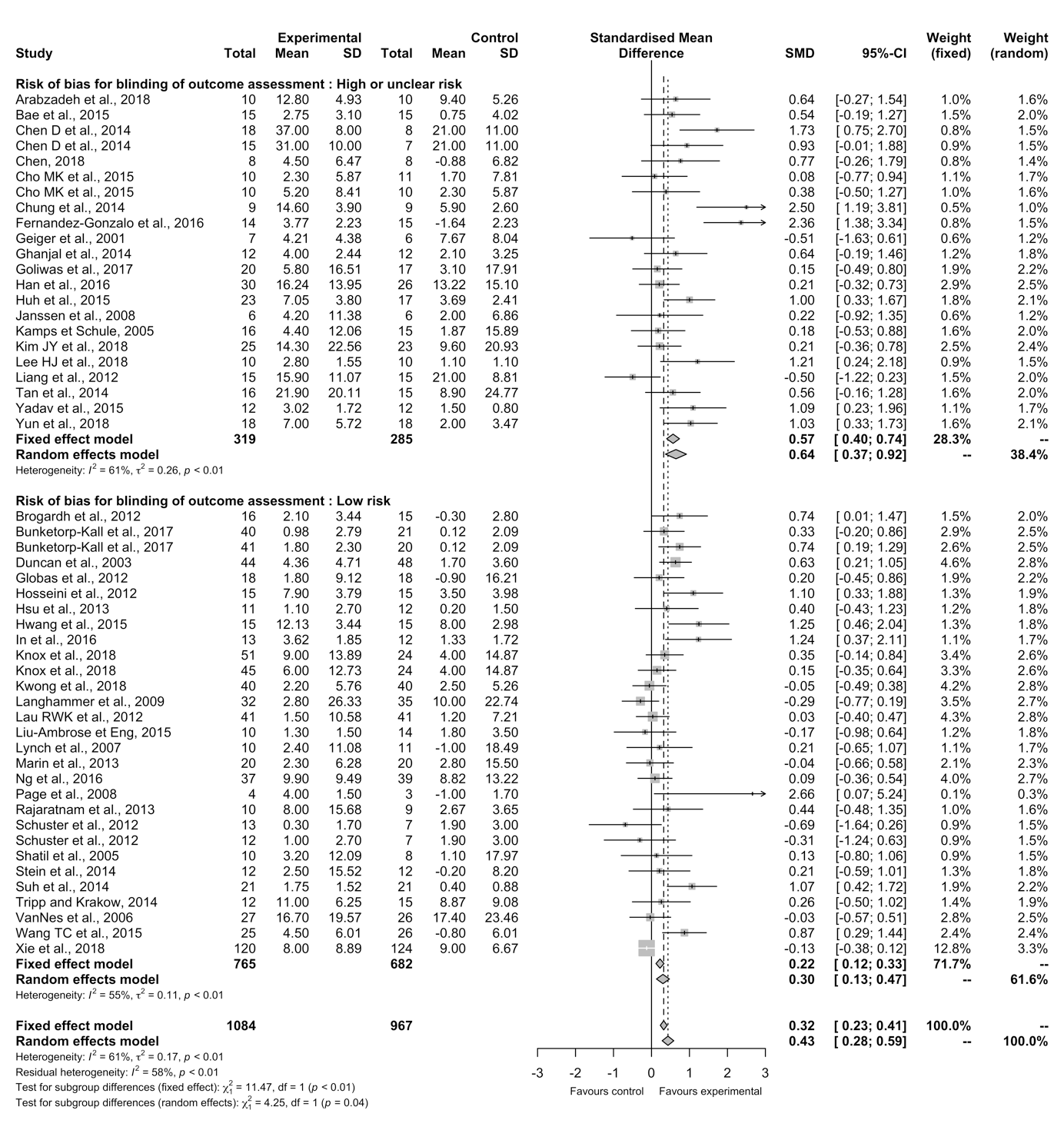

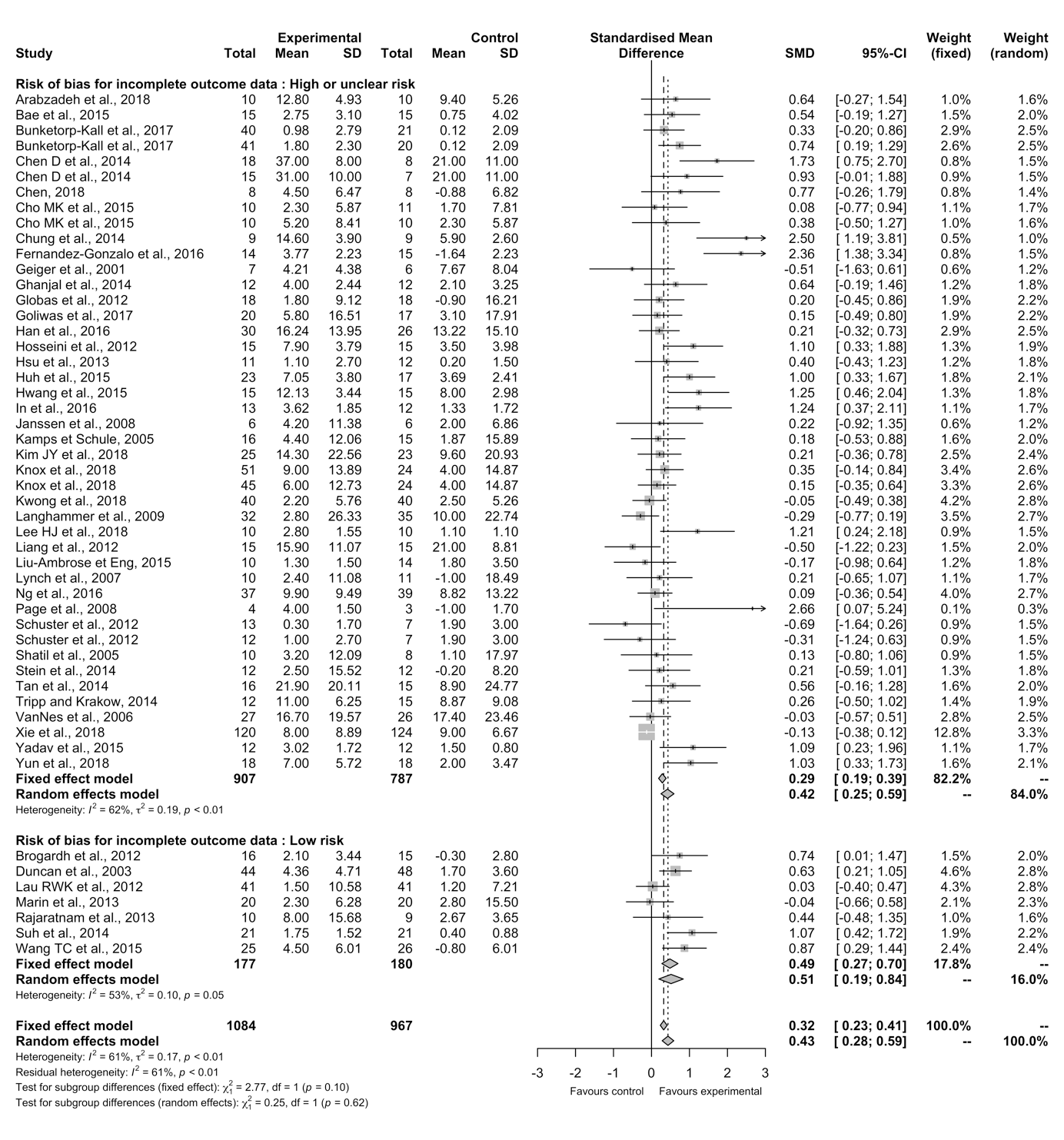

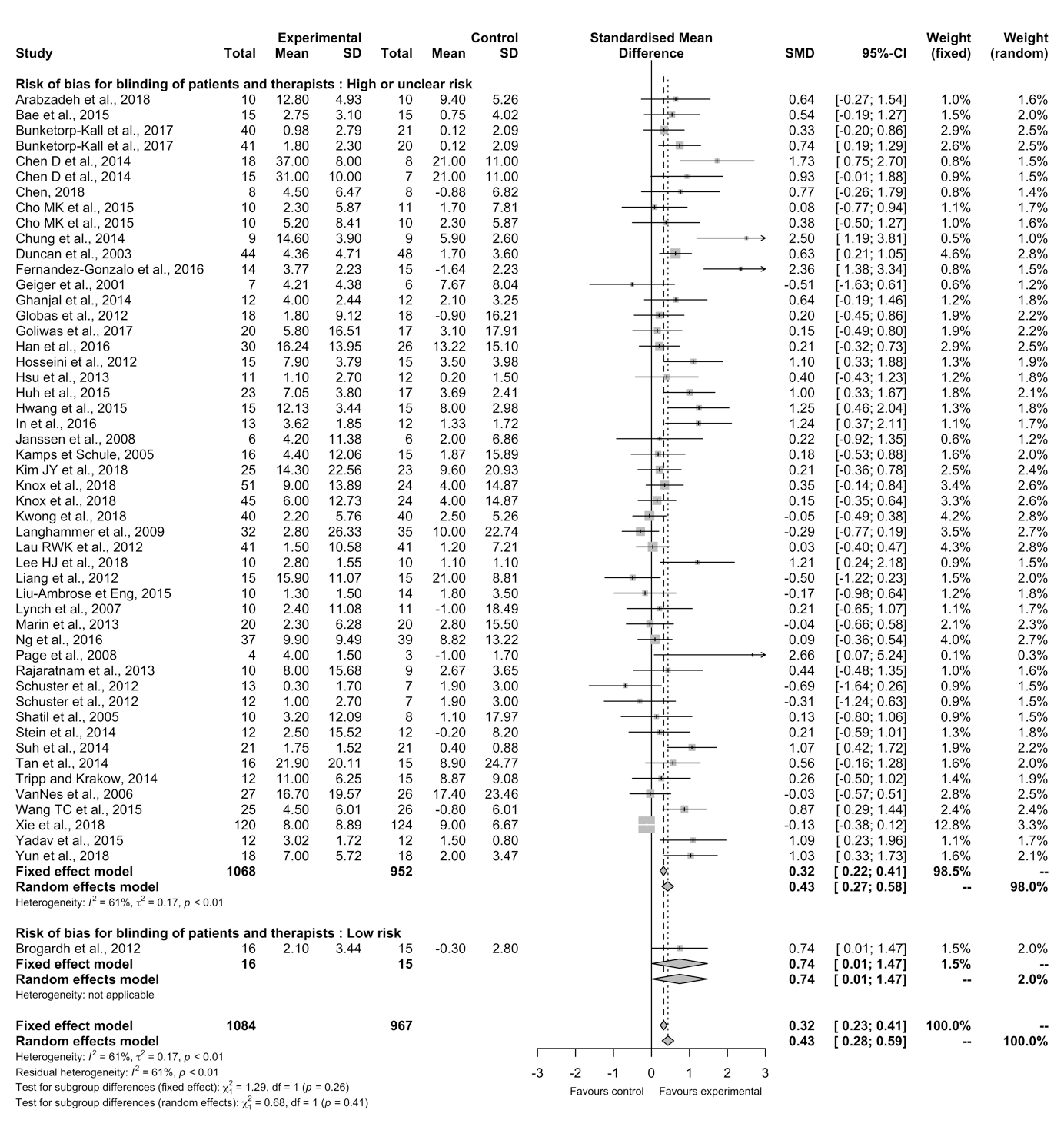

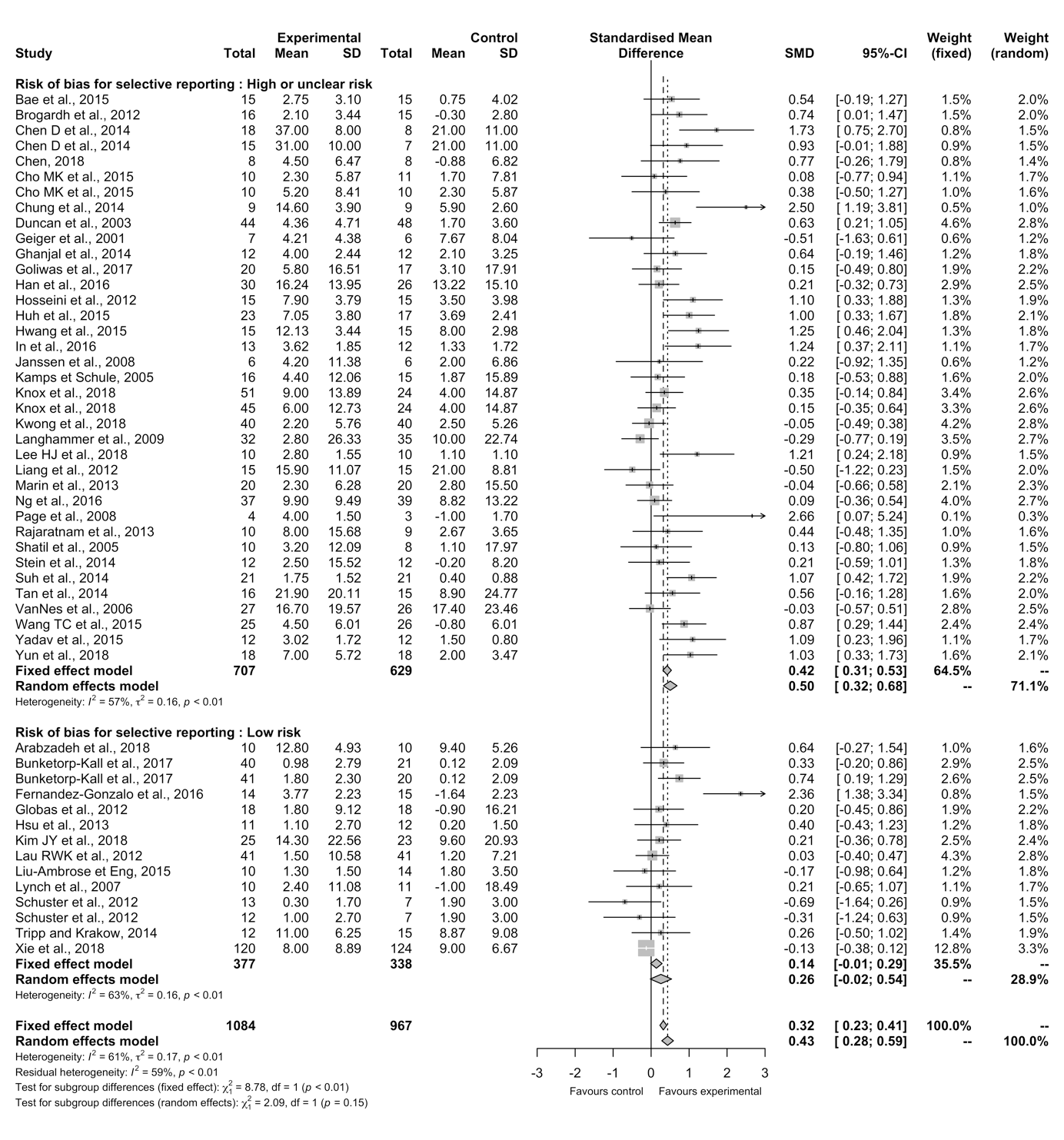
**

**
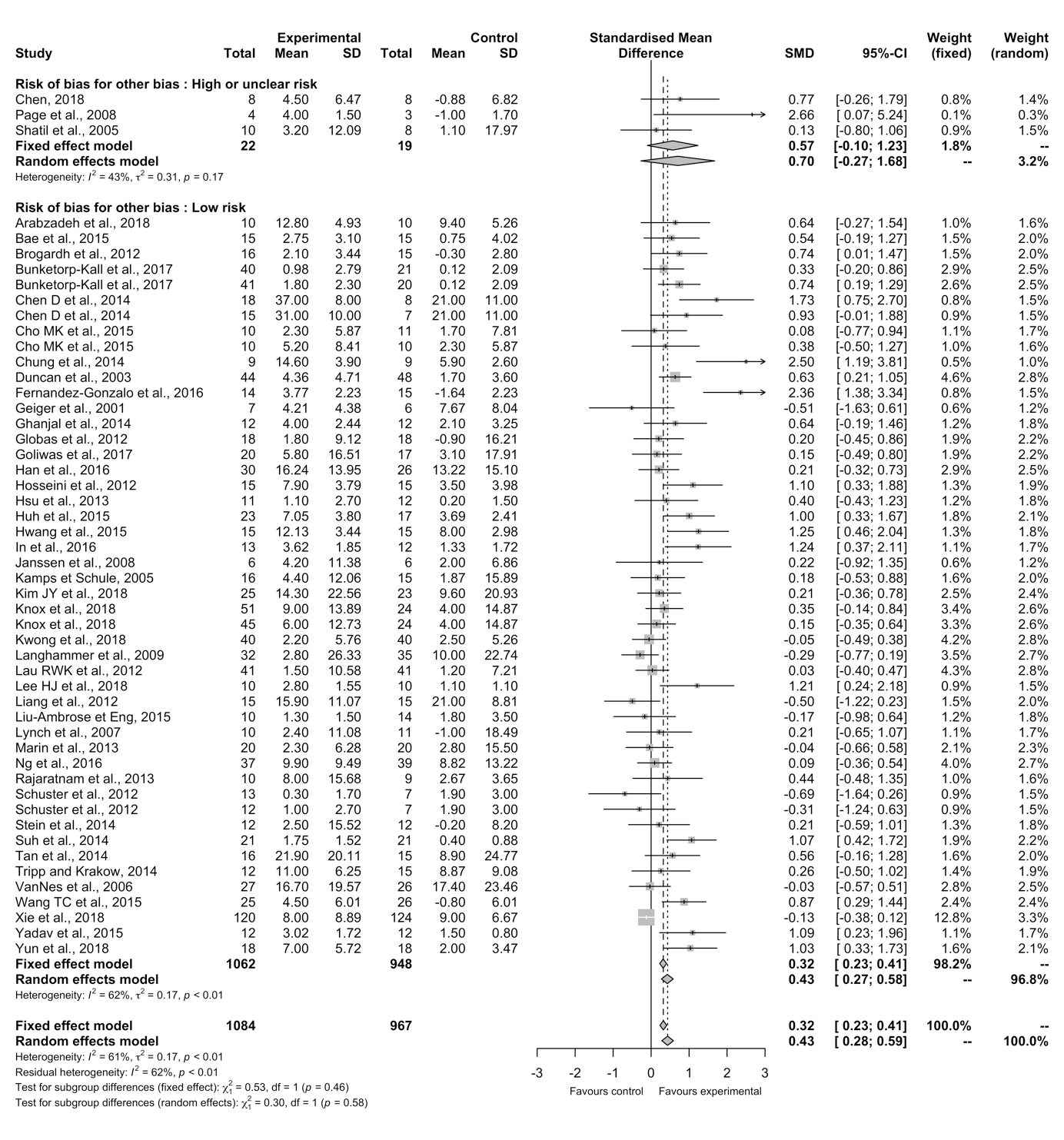
**
