# Supplementary material for: Limited evidence of physical therapy on balance after stroke: A systematic review and meta-analysis
Source: PLoS One. 2019 Aug 29;14(8):e0221700. doi: 10.1371/journal.pone.0221700 (PMC6715189; doi:10.1371/journal.pone.0221700)
Supplement: S11 Fig — Outcome: Autonomy. Subgroup: Categories of PT. (DOCX) [file pone.0221700.s012.docx]

**S11 Fig. Forest plot of physical therapy. Outcome: Autonomy. Subgroup: Categories of PT**

**S11A Fig. Forest plot of physical therapy versus no treatment. Outcome: Autonomy, post-intervention effects. Subgroup: Categories of PT**

**
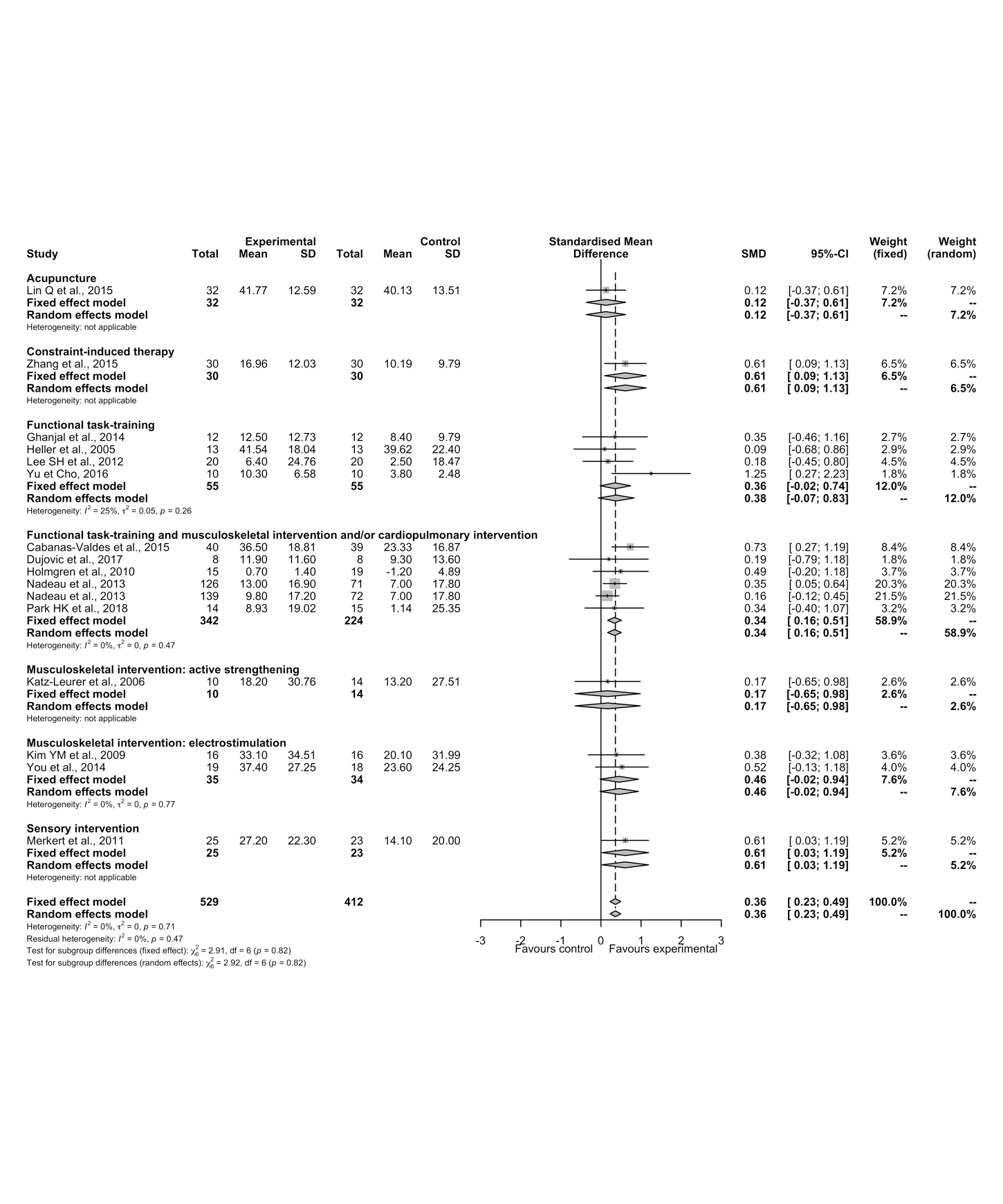
**

**S11B Fig. Forest plot of physical therapy versus no treatment. Outcome: Autonomy, persisting effects. Subgroup: Categories of PT**

**
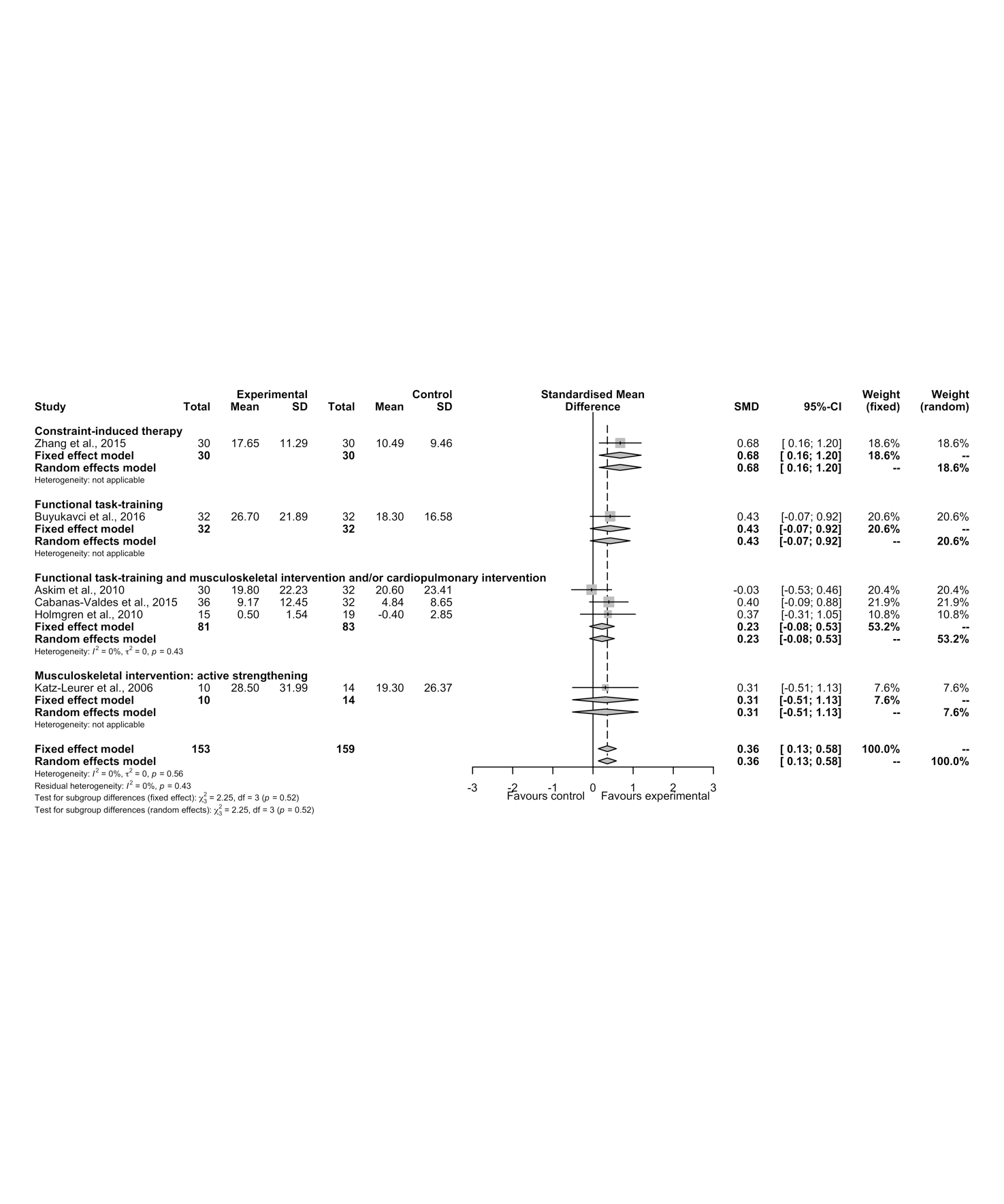
**

**S11C Fig. Forest plot of physical therapy versus sham treatment/usual care. Outcome: Autonomy, post-intervention effects. Subgroup: Categories of PT**

**
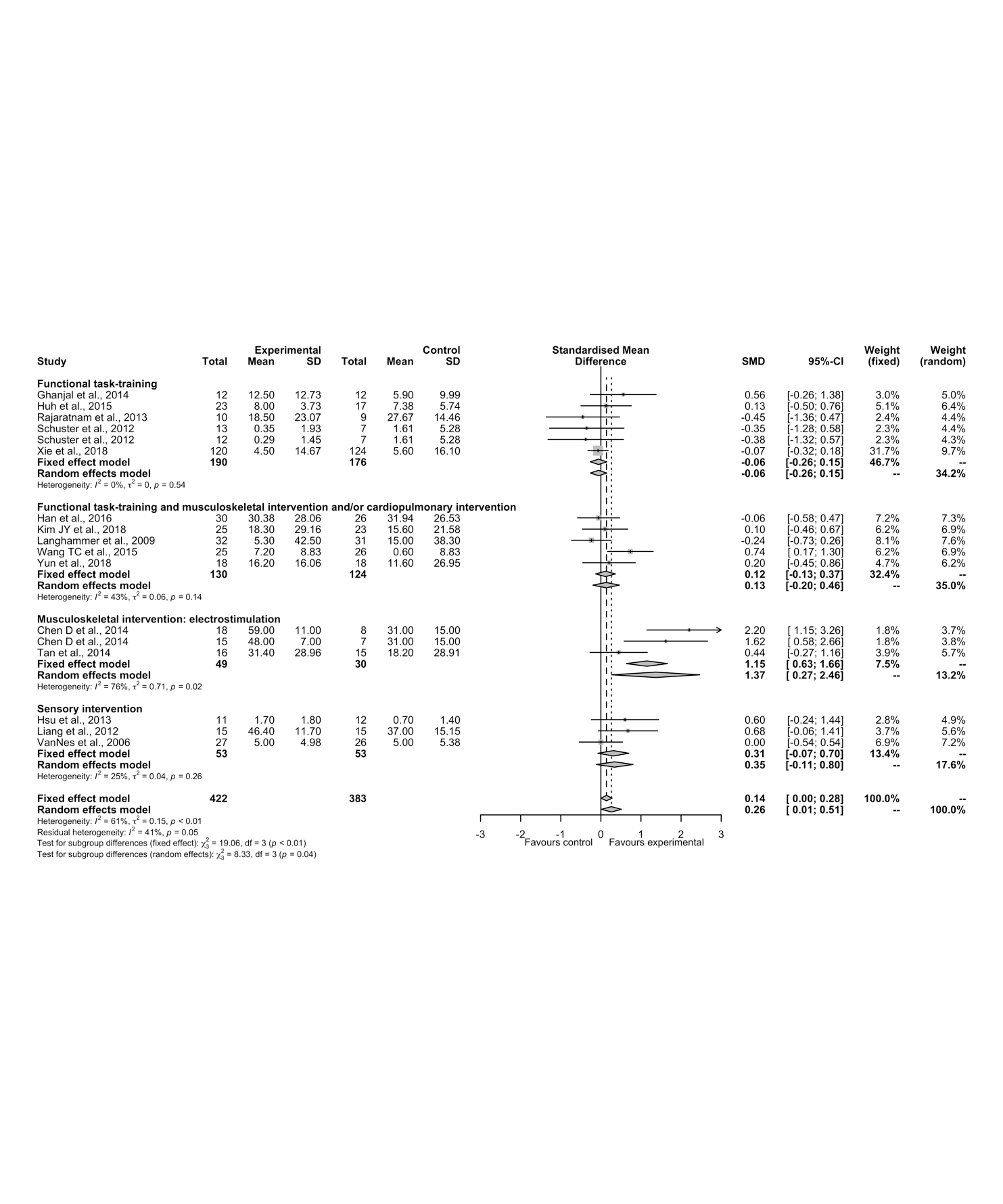
**

**S11A Fig. Forest plot of physical therapy versus sham treatment/usual care. Outcome: Autonomy, persisting effects. Subgroup: Categories of PT**

**
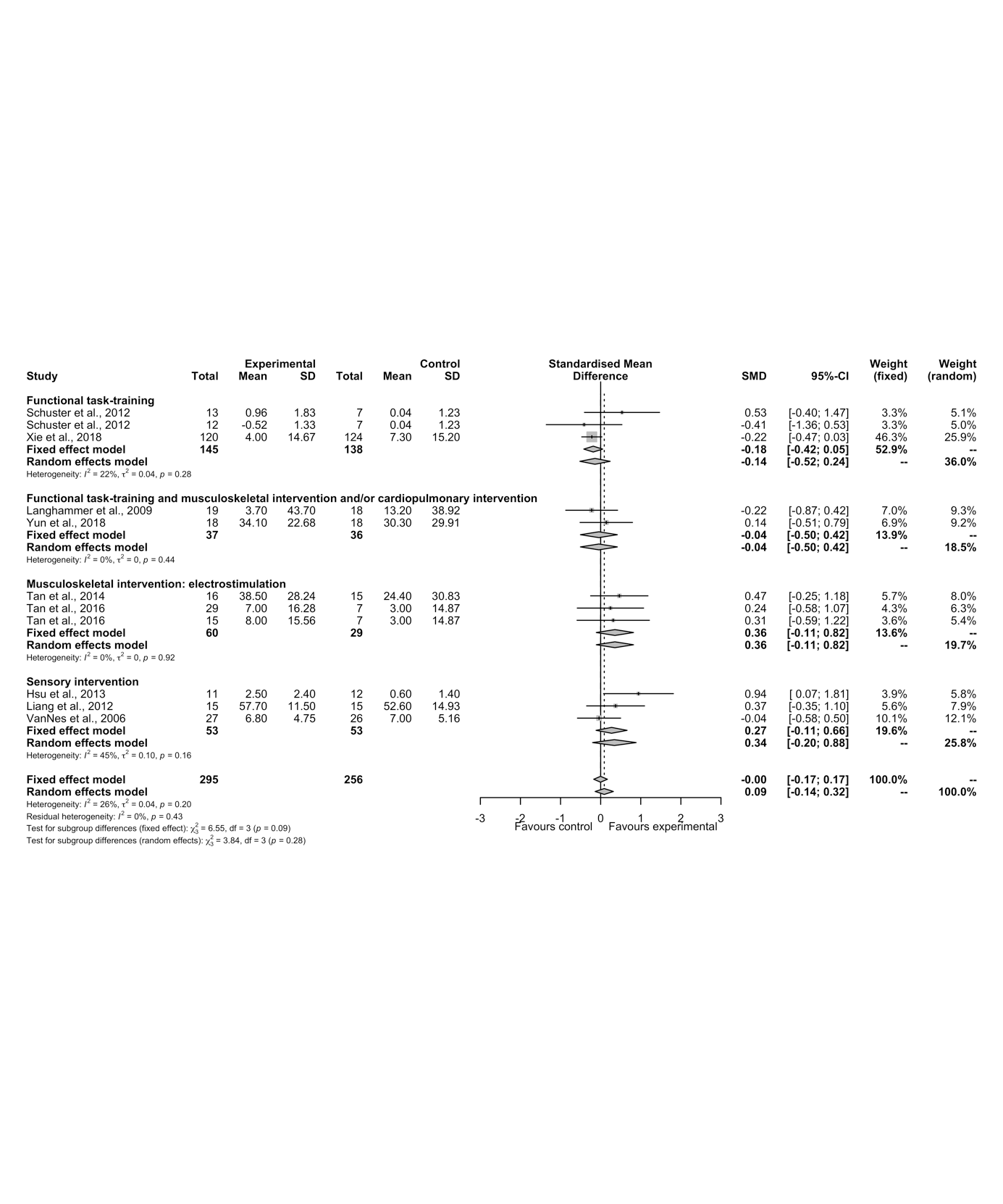
**
