# Supplementary material for: Limited evidence of physical therapy on balance after stroke: A systematic review and meta-analysis
Source: PLoS One. 2019 Aug 29;14(8):e0221700. doi: 10.1371/journal.pone.0221700 (PMC6715189; doi:10.1371/journal.pone.0221700)
Supplement: S1 Table — (DOCX) [file pone.0221700.s015.docx]

**S1 Table. Search strategy in databases**

**S1A Table. Search strategy in Pubmed – October 31, 2015**

| 1 | exercise movement techniques OR physical therapy modalities OR learning OR pract* OR train* OR rehabilitation* OR therapeutic* OR therapy OR therapies OR exercise* OR physiotherap* OR neurorehabilitation OR neurophysiological OR orthopaed* OR treatment OR approach* OR concept OR home rehabilitation OR self-guided program* OR fitness OR stretching OR sport OR program* OR movement OR protocol* OR intervention OR activit* OR regim* OR recovery |
| --- | --- |
| 2 | (occupational OR physical OR manual) AND (therapy OR therapies OR therapist OR therapeutic OR therapeutics) |
| 3 | #1 OR #2 |
| 4 | posture OR equilibrium OR balance OR postural balance OR weight bearing OR weight shift OR lateropulsion OR pusher OR pushing OR postural imbalance OR postural asymmetry OR postural control OR postural stability OR postural instability OR postural perturbation OR postural disorders OR postural deficit OR postural trouble OR postural sway OR postural tilt OR postural shift OR body sway OR upright stance OR (weight AND (distribut* OR transfer*)) |
| 5 | (cerebrovascular OR cerebro-vascular OR cerebral OR intracran* OR hemispheric) AND (accident OR hemorrhag* OR haemorrhag* OR infarct* OR ischemi* OR thrombotic OR thrombosis OR emboli* OR hematoma OR haematoma OR bleed OR damage OR lesion OR occlus*) |
| 6 | stroke OR poststroke OR post-stroke OR hemipleg* OR hemipar* OR paretic OR paresis OR CVA |
| 7 | (right OR left) AND brain AND (lesion OR damage) |
| 8 | #5 OR #6 OR #7 |
| 9 | meta-analysis OR review* OR animal* OR child* OR cerebral pals* OR case-report OR traumatic brain injury |
| 10 | #3 AND #4 AND #8 NOT #9 |

**S1B Table. Search strategy in Embase – October 31, 2015**

| 1 | 'cerebrovascular accident'/exp OR 'brain ischemia'/exp OR 'hemiplegia'/exp OR 'hemiparesis'/exp OR 'brain infarction'/de OR 'brain hemorrhage'/de OR 'stroke' OR 'poststroke' OR 'post-stroke' OR cerebrovascular OR 'cerebro vascular' OR cerebral OR intracran* OR hemispheric AND (accident OR hemorrhag* OR haemorrhag* OR infarct* OR ischemi* OR thrombotic OR thrombosis OR emboli* OR hematoma OR haematoma OR bleed OR damage OR lesion OR occlus*) OR paretic OR paresis OR cva OR (right OR left AND brain AND (lesion OR damage)) |
| --- | --- |
| 2 | 'body equilibrium'/exp OR 'balance impairment'/exp OR 'weight bearing'/de OR 'weight shift' OR 'lateropulsion' OR 'pusher' OR 'pushing' OR 'abnormal posture'/exp OR 'postural asymmetry' OR 'postural control' OR 'postural stability' OR 'postural instability'OR 'postural perturbation' OR 'postural deficit' OR 'postural trouble' OR 'postural sway' OR 'postural tilt or postural shift' OR 'body posture'/exp OR 'body sway' OR 'upright stance' OR 'weight distribution' OR 'weight transfert' |
| 3 | 'training'/exp OR 'rehabilitation medicine'/exp OR 'occupational therapy'/exp OR 'home rehabilitation'/exp OR 'home physiotherapy'/exp OR 'exercise' OR 'neurorehabilitation' OR 'neurophysiological' OR 'orthopaedic' OR 'therapy' OR 'treatment' OR 'approach' OR 'concept' OR 'physical medicine'/exp OR 'physical medicine' OR 'learning'/exp OR 'learning' OR pract* OR therapeutic* OR 'fitness' OR 'stretching' OR program* OR 'movement' OR protocol* OR 'intervention' OR activit* OR regim* OR 'recovery' |
| 4 | 'meta-analysis' OR 'review' OR 'animal' OR 'children' OR 'cerebral palsy' |
| 5 | #1 AND #2 AND #3 NOT #4 |

**S1C Table. Search strategy in Pubmed – January 14, 2019**

| 1 | exercise movement techniques OR physical therapy modalities OR learning OR pract* OR train* OR rehabilitation* OR therapeutic* OR therapy OR therapies OR exercise* OR physiotherap* OR neurorehabilitation OR neurophysiological OR orthopaed* OR treatment OR approach* OR concept OR home rehabilitation OR self-guided program* OR fitness OR stretching OR sport OR program* OR movement OR protocol* OR intervention OR activit* OR regim* OR recovery |
| --- | --- |
| 2 | (occupational OR physical OR manual) AND (therapy OR therapies OR therapist OR therapeutic OR therapeutics) |
| 3 | #1 OR #2 |
| 4 | posture OR equilibrium OR balance OR postural balance OR weight bearing OR weight shift OR lateropulsion OR pusher OR pushing OR postural imbalance OR postural asymmetry OR postural control OR postural stability OR postural instability OR postural perturbation OR postural disorders OR postural deficit OR postural trouble OR postural sway OR postural tilt OR postural shift OR body sway OR upright stance OR (weight AND (distribut* OR transfer*)) |
| 5 | (cerebrovascular OR cerebro-vascular OR cerebral OR intracran* OR hemispheric) AND (accident OR hemorrhag* OR haemorrhag* OR infarct* OR ischemi* OR thrombotic OR thrombosis OR emboli* OR hematoma OR haematoma OR bleed OR damage OR lesion OR occlus*) |
| 6 | stroke OR poststroke OR post-stroke OR hemipleg* OR hemipar* OR paretic OR paresis OR CVA |
| 7 | (right OR left) AND brain AND (lesion OR damage) |
| 8 | #5 OR #6 OR #7 |
| 9 | Randomized Controlled Trial[Publication Type] OR randomized controlled trial[Publication Type] OR Randomised Controlled Trial[Publication Type] OR randomised controlled trial[Publication Type] OR randomized controlled trials as topic[MeSH Terms] OR randomized controlled trial OR randomised controlled trial OR RCT |
| 10 | meta-analysis OR review* OR animal* OR child* OR cerebral pals* OR case-report OR traumatic brain injury |
| 11 | #3 AND #4 AND #8 AND #9 NOT #10 |
