# Supplementary material for: Limited evidence of physical therapy on balance after stroke: A systematic review and meta-analysis
Source: PLoS One. 2019 Aug 29;14(8):e0221700. doi: 10.1371/journal.pone.0221700 (PMC6715189; doi:10.1371/journal.pone.0221700)
Supplement: S2 Table — (DOCX) [file pone.0221700.s016.docx]

**S2 Table. Identification of studies included in the systematic review and meta-analysis**

| Author | Title | Publication | Year of publication | Issue | Volume | Pages |
| --- | --- | --- | --- | --- | --- | --- |
| Allison, R; Dennett, R | Pilot randomized controlled trial to assess the impact of additional supported standing practice on functional ability post stroke | Clinical Rehabilitation | 2007 | 7 | 21 | 614-619 |
| Arabzadeh, S; Goljaryan, S; Salahzadeh, Z; et al. | Effects of a Task-Oriented Exercise Program on Balance in Patients with Hemiplegia Following Stroke | Iranian Red Crescent Medical Journal | 2016 | 1 | 20 | NA |
| Askim, T; Morkved, S; Engen, A; et al. | Effects of a community-based intensive motor training program combined with early supported discharge after treatment in a comprehensive stroke unit: a randomized, controlled trial | Stroke | 2010 | 8 | 41 | 1697-1703 |
| Au-Yeung, S; Hui-Chan, C; Tang, J | Short-form Tai Chi Improves Standing Balance of People With Chronic Stroke | Neurorehabilitation and Neural Repair | 2009 | 5 | 23 | 515-522 |
| Bae, YH; Kim, HG; Min, KS; et al. | Effects of Lower-Leg Kinesiology Taping on Balance Ability in Stroke Patients with Foot Drop | Evidence-Based Complementary and Alternative Medicine | 2015 | NA | NA | NA |
| Barcala, L; Colella, F; Araujo, MC; et al. | Análise do equilíbrio em pacientes hemiparéticos  após o treino com o programa Wii Fit | Fisioterapia em Movimento | 2011 | 2 | 24 | 337-343 |
| Brogardh, C; Flansbjer, UB; Lexell, J | No specific effect of whole-body vibration training in chronic stroke: a double-blind randomized controlled study | Archives of Physical Medicine and Rehabilitation | 2012 | 2 | 93 | 253-258 |
| Bunketorp-Käll, L; Lundgren-Nilsson, Å; Samuelsson, H; et al. | Long-Term Improvements After Multimodal Rehabilitation in Late Phase After Stroke: A Randomized Controlled Trial | Stroke | 2017 | 7 | 48 | 1916-1924 |
| Büyükavcı, R | The impact of additional trunk balance exercises on balance, functional condition and ambulation in early stroke patients: Randomized controlled trial | Türkiye Fiziksel Tıp ve Rehabilitasyon Dergisi | 2016 | 3 | 62 | 248-256 |
| Büyükvural SS, Özbudak DS, Ekiz T, Özgirgin N. | Effects of the bilateral isokinetic strengthening training on functional parameters, gait, and the quality of life in patients with stroke | International Journal of Clinical and Experimental Medicine | 2015 | 9 | 8 | 16871-16879 |
| Cabanas-Valdés, R; Bagur-Calafat, C; Girabent-Farrés, M; et al. | The effect of additional core stability exercises on improving dynamic sitting balance and trunk control for subacute stroke patients: a randomized controlled trial | Clinical Rehabilitation | 2016 | 10 | 30 | 1024-1033 |
| Chan, KS; Liu, CW; Chen, TW; et al. | Effects of a single session of whole body vibration on ankle plantarflexion spasticity and gait performance in patients with chronic stroke: a randomized controlled trial | Clinical Rehabilitation | 2012 | 12 | 26 | 1087-1095 |
| Chen, CH; Lin, KH; Lu, TW; et al. | Immediate effect of lateral-wedged insole on stance and ambulation after stroke: | American Journal of Physical Medicine & Rehabilitation | 2010 | 1 | 89 | 48-55 |
| Chen, CL; Chen, FF; Lin, CH; et al. | Effect of anterior ankle-foot orthoses on weight shift in persons with stroke | Archives of Physical Medicine and Rehabilitation | 2015 | 10 | 96 | 1795-1801 |
| Chen, D; Yan, T; Li, G; et al. | Functional electrical stimulation based on a working pattern influences function of lower extremity in subjects with early stroke and effects on diffusion tensor imaging: a randomized controlled trial | Zhonghua Yi Xue Za Zhi | 2014 | 37 | 94 | 2886-2892 |
| Chen, IC; Cheng, PT; Chen, CL; et al. | Effects of balance training on hemiplegic stroke patients | Chang Gung Medical Journal | 2002 | 9 | 25 | 583-590 |
| Chen, JC; Lin, CH; Wei, YC; et al. | Facilitation of motor and balance recovery by thermal intervention for the paretic lower limb of acute stroke: a single-blind randomized clinical trial | Clinical Rehabilitation | 2011 | 9 | 25 | 823-832 |
| Chen, T | Effects of Martial Arts on Recovery of Motor Function and Nerve Excitability of Stroke Patients | NeuroQuantology | 2018 | 6 | 16 | 894-898 |
| Chern, JS; Chang, HS; Lung, CW; et al. | Static ankle-foot orthosis improves static balance and gait functions in hemiplegic patients after stroke | 35th Annual International Conference of the IEEE EMBS | 2013 | NA | NA | 5009-5012 |
| Cho, HY; In, TS; Cho, KH; Song, CH | A single trial of transcutaneous electrical nerve stimulation (TENS) improves spasticity and balance in patients with chronic stroke | The Tohoku Journal of Experimental Medicine | 2013 | 3 | 229 | 187-193 |
| Cho, KH; Lee, KJ; Song, CH | Virtual-reality balance training with a video-game system improves dynamic balance in chronic stroke patients | The Tohoku Journal of Experimental Medicine | 2012 | 1 | 228 | 69-74 |
| Cho, MK; Kim, JH; Chung, Y; Hwang, S | Treadmill gait training combined with functional electrical stimulation on hip abductor and ankle dorsiflexor muscles for chronic hemiparesis | Gait & Posture | 2015 | 1 | 42 | 73-78 |
| Choi, HS; Shin, WS; Bang, DH; et al. | Effects of Game-Based Constraint-Induced Movement Therapy on Balance in Patients with Stroke: A Single-Blind Randomized Controlled Trial | American Journal of Physical Medicine & Rehabilitation | 2017 | 3 | 96 | 184-190 |
| Chu, JM; Bao, YH; Zhu, M | [Effects of Acupuncture Intervention Combined with Rehabilitation on Standing-balance-walking Ability in Stroke Patients] | Zhen Ci Yan Jiu (Acupuncture Research) | 2015 | 6 | 40 | 474-478 |
| Chung, Y; Kim, JH; Cha, Y; Hwang, S | Therapeutic effect of functional electrical stimulation-triggered gait training corresponding gait cycle for stroke | Gait & Posture | 2014 | 3 | 40 | 471-475 |
| Dault, MC; de Haart, M; Geurts, ACH; Arts, IMP; Nienhuis, B | Effects of visual center of pressure feedback on postural control in young and elderly healthy adults and in stroke patients | Human Movement Science | 2003 | 3 | 22 | 221-236 |
| Dujović, SD; Malešević, J; Malešević, N; et al. | Novel multi-pad functional electrical stimulation in stroke patients: A single-blind randomized study | NeuroRehabilitation | 2017 | 4 | 41 | 791-800 |
| Duncan, P; Richards, L; Wallace, D; et al. | A randomized, controlled pilot study of a home-based exercise program for individuals with mild and moderate stroke | Stroke | 1998 | 10 | 29 | 2055-2060 |
| Duncan, P; Studenski, S; Richards, L; et al. | Randomized clinical trial of therapeutic exercise in subacute stroke | Stroke | 2003 | 9 | 34 | 2173-2180 |
| Erbil, D; Tugba, G; Murat, TH; et al. | Effects of robot-assisted gait training in chronic stroke patients treated by botulinum toxin-a: A pivotal study | Physiotherapy Research International | 2018 | 3 | 23 | e1718 |
| Fernandez-Gonzalo, R; Fernandez-Gonzalo, S; Turon, M; et al. | Muscle, functional and cognitive adaptations after flywheel resistance training in stroke patients: a pilot randomized controlled trial | Journal of NeuroEngineering and Rehabilitation | 2016 | 1 | 13 | 13-37 |
| Ferreira, LAB; Galli, M; Lazzari, RD; et al. | Stabilometric analysis of the effect of postural insoles on static balance in patients with hemiparesis: A randomized, controlled, clinical trial | Journal of Bodywork and Movement Therapies | 2017 | 2 | 21 | 290-296 |
| Fritz, SL; Peters, DM; Merlo, AM; Donley, J | Active video-Gaming effects on balance and mobility in individuals with chronic stroke: a randomized controlled trial | Topics in Stroke Rehabilitation | 2013 | 3 | 20 | 218-225 |
| Furnari, A; Calabrò, RS; Gervasi, G; et al. | Is hydrokinesitherapy effective on gait and balance in patients with stroke? A clinical and baropodometric investigation | Brain Injury | 2014 | 8 | 28 | 1109-1114 |
| Geiger, RA; Allen, JB; O'Keefe, J; et al. | Balance and Mobility Following Stroke: Effects of Physical Therapy Interventions With and Without Biofeedback/Forceplate Training | Physical Therapy | 2001 | 4 | 81 | 995-1005 |
| Ghanjal, A; Torkaman, G; Ghabaee, M; Ebrahimi,l; Motoqhey, M | Effect of action observation and imitation on improving the functional activities indices in hemiplegic patients based on mirror neurons theory | J Mazandaran Univ Med Sci | 2014 | 118 | 24 | 136-150 |
| Globas, C; Becker, C; Cerny, J; et al. | Chronic stroke survivors benefit from high-intensity aerobic treadmill exercise: a randomized control trial | Neurorehabilitation and Neural Repair | 2012 | 1 | 26 | 85-95 |
| Goliwąs, M; Kocur, P; Wiernicka, M; et al. | Effect of sensorimotor foot stimulation on the body postural, function and load of the lower limb in patients in the late phase after stroke | Fizjoterapia Polska | 2017 | 2 | 17 | 24-35 |
| Han,Y; Im, SH; Kim, BR; et al. | Robot-assisted gait training improves brachial–ankle pulse wave velocity and peak aerobic capacity in subacute stroke patients with totally dependent ambulation: Randomized controlled trial | Medicine | 2016 | 41 | 95 | NA |
| Hart, J; Kanner, H; Gilboa-Mayo, R; et al. | Tai Chi Chuan practice in community-dwelling persons after stroke | International Journal of Rehabilitation Research. | 2004 | 4 | 27 | 303-304 |
| Heller, F; Beuret-Blanquart, F; Weber, J | Barobiofeedback et rééducation de la marche de l'hémiplégique | Annales de Réadaptation et de Médecine Physique | 2005 | 4 | 48 | 187-195 |
| Hollands, KL; Pelton, TA; Wimperis, A; et al. | Feasibility and Preliminary Efficacy of Visual Cue Training to Improve Adaptability of Walking after Stroke: Multi-Centre, Single-Blind Randomised Control Pilot Trial | PLOS ONE | 2015 | 10 | 10 | e0139261 |
| Holmgren, E; Lindström, B; Gosman-Hedström, G; et al. | What is the benefit of a high intensive exercise program? A randomized controlled trial | Advances in Physiotherapy | 2010 | 3 | 12 | 115-124 |
| Hosseini, SA; Fallahpour, M; Sayadi, M; et al. | The impact of mental practice on stroke patients' postural balance | Journal of the Neurological Sciences | 2012 | 1-2 | 322 | 263-267 |
| Howe, TE; Taylor, I; Finn, P; Jones, H | Lateral weight transference exercises following acute stroke: a preliminary study of clinical effectiveness | Clinical Rehabilitation | 2005 | 1 | 19 | 45-53 |
| Hsieh, HC | Training by Using an Adaptive Foot Switch and Video Games to Improve Balance and Mobility Following Stroke: A Randomised Controlled Trial | Brain Impairment | 2019 | 1 | 20 | 16-23 |
| Hsu, HW; Lee, CL; Hsu, MJ; et al. | Effects of noxious versus innocuous thermal stimulation on lower extremity motor recovery 3 months after stroke | Archives of Physical Medicine and Rehabilitation | 2013 | 4 | 94 | 633-641 |
| Huh, JS; Lee, YS; Kim, CH; et al. | Effects of Balance Control Training on Functional Outcomes in Subacute Hemiparetic Stroke Patients | Annals of Rehabilitation Medicine | 2015 | 6 | 39 | 995 |
| Hung, JW; Yu, MY; Chang, KC; et al. | Feasibility of Using Tetrax Biofeedback Video Games for Balance Training in Patients With Chronic Hemiplegic Stroke | PM&R | 2016 | 10 | 8 | 962-970 |
| Hwang, DY; Lee, HJ; Lee, GC; Lee, SM | Treadmill training with tilt sensor functional electrical stimulation for improving balance, gait, and muscle architecture of tibialis anterior of survivors with chronic stroke: A randomized controlled trial | Technology and Health Care | 2015 | 4 | 23 | 443-452 |
| Immink, MA; Hillier, S; Petkov, J | Randomized controlled trial of yoga for chronic poststroke hemiparesis: motor function, mental health, and quality of life outcomes | Topics in Stroke Rehabilitation | 2014 | 3 | 21 | 256-271 |
| In, T; Lee, K; Song, C | Virtual Reality Reflection Therapy Improves Balance and Gait in Patients with Chronic Stroke: Randomized Controlled Trials | Medical Science Monitor | 2016 | NA | 22 | 4046-4053 |
| Janssen, TW; Beltman, JM; Elich, P; et al. | Effects of electric stimulation‚ assisted cycling training in people with chronic stroke | Archives of Physical Medicine and Rehabilitation | 2008 | 3 | 89 | 463-469 |
| Jung, J; Choi, W; Lee, S | Trunk stabilization training using visual feedback on an unstable surface improves balance and trunk stability of chronic stroke patients | Medical Science and Technology | 2015 | NA | 56 | 37-42 |
| Jung, KS; In, TS; Cho, HY | Effects of sit-to-stand training combined with transcutaneous electrical stimulation on spasticity, muscle strength and balance ability in patients with stroke: A randomized controlled study | Gait & Posture | 2017 | NA | 54 | 183-187 |
| Kamps, A; Schule, K | Cyclic movement training of the lower limb in stroke rehabilitation | Neurologie & Rehabilitation | 2005 | 3 | 11 | S1-S12 |
| Karasu, A; Batur, E; Karataş, G | Effectiveness of Wii-based rehabilitation in stroke: A randomized controlled study | Journal of Rehabilitation Medicine | 2018 | 5 | 50 | 406-412 |
| Katz-Leurer, M; Sender, I; Keren, O; Dvir, Z | The influence of early cycling training on balance in stroke patients at the subacute stage. Results of a preliminary trial | Clinical Rehabilitation | 2006 | 5 | 20 | 398-405 |
| Khumsapsiri, N; Siriphorn, A; Pooranawatthanakul, K; et al. | Training using a new multidirectional reach tool improves balance in individuals with stroke | Physiotherapy Research International | 2018 | 2 | 23 | NA |
| Kim, DH; Yi, TI; Kim, JS; et al. | The effects of isokinetic strengthening of trunk muscles on balance in hemiplegic patients | J Korean Acad Rehab Med | 2008 | 3 | 32 | 280-284 |
| Kim, JC; Lee, HM | The Effect of Action Observation Training on Balance and Sit to Walk in Chronic Stroke: A Crossover Randomized Controlled Trial | Journal of Motor Behavior | 2018 | 4 | 50 | 373-380 |
| Kim, JH; Jang, SH; Kim, CS; et al. | Use of virtual reality to enhance balance and ambulation in chronic stroke: A double-blind, randomized controlled Study: | American Journal of Physical Medicine & Rehabilitation | 2009 | 9 | 88 | 693-701 |
| Kim, JY; Kim, DY; Chun, MH; et al. | Effects of robot-(Morning Walk®) assisted gait training for patients after stroke: a randomized controlled trial | Clinical Rehabilitation | 2019 | 3 | 33 | 516-523 |
| Kim, SL; Lee, BH | The effects of posterior talar glide and dorsiflexion of the ankle plus mobilization with movement on balance and gait function in patient with chronic stroke: A randomized controlled trial | Journal of Neurosciences in Rural Practice | 2018 | 1 | 9 | 61 |
| Kim, YH; Shin, JE; Kim, DH; et al. | Effect of dynamic balance training using visual biofeedback of center of pressure in patients with stroke | J Korean Acad Rehabil Med. | 2004 | 6 | 28 | 515-522 |
| Kim, YM; Chun, MH; Kang, SH; Ahn, WH | The effect of neuromuscular electrical stimulation on trunk control in hemiparetic stroke patients | J Korean Acad Rehabil Med. | 2009 | 3 | 33 | 265-270 |
| Kılınç, M; Avcu, F; Onursal, O; et al. | The effects of Bobath-based trunk exercises on trunk control, functional capacity, balance, and gait: a pilot randomized controlled trial | Topics in Stroke Rehabilitation | 2016 | 1 | 23 | 50-58 |
| Knox, M; Stewart, A; Richards, CL | Six hours of task-oriented training optimizes walking competency post stroke: a randomized controlled trial in the public health-care system of South Africa | Clinical Rehabilitation | 2018 | 8 | 32 | 1057-1068 |
| Kunkel, D; Pickering, RM; Burnett, M; et al. | Functional electrical stimulation with exercises for standing balance and weight transfer in acute stroke patients: a Feasibility randomized controlled trial | Neuromodulation: Technology at the Neural Interface | 2013 | 2 | 16 | 168-177 |
| Kwong, PWH; Ng, GYF; Chung, RCK; et al. | Bilateral Transcutaneous Electrical Nerve Stimulation Improves Lower‐Limb Motor Function in Subjects With Chronic Stroke: A Randomized Controlled Trial | Journal of the American Heart Association | 2018 | 4 | 7 | NA |
| Langhammer, B; Stanghelle, JK; Lindmark, B | An evaluation of two different exercise regimes during the first year following stroke: A randomised controlled trial | Physiotherapy Theory and Practice | 2009 | 2 | 25 | 55-68 |
| Lau, RWK; Yip, SP; Pang, MYC | Whole-body vibration has no effect on neuromotor function and falls in chronic stroke: | Medicine & Science in Sports & Exercise | 2012 | 8 | 44 | 1409-1418 |
| Laufer, Y | The effect of walking aids on balance and weight-bearing patterns of patients with hemiparesis in various stance positions | Physical Therapy | 2003 | 2 | 83 | 112-122 |
| Lee, CH; Kim, Y; Lee, BH | Augmented reality-based postural control training improves gait function in patients with stroke: Randomized controlled trial | Hong Kong Physiotherapy Journal | 2014 | 2 | 32 | 51-57 |
| Lee, DG; Lee, GC; Jeong, JS | Mirror Therapy with Neuromuscular Electrical Stimulation for improving motor function of stroke survivors: A pilot randomized clinical study | Technology and Health Care | 2016 | 4 | 24 | 503-511 |
| Lee, HJ; Kang, TW; Kim, BR | Effects of diaphragm and deep abdominal muscle exercise on walking and balance ability in patients with hemiplegia due to stroke | Journal of Exercise Rehabilitation | 2018 | 4 | 14 | 648-653 |
| Lee, MM; Lee, KJ; Song, CH | Game-Based Virtual Reality Canoe Paddling Training to Improve Postural Balance and Upper Extremity Function: A Preliminary Randomized Controlled Study of 30 Patients with Subacute Stroke | Medical Science Monitor | 2018 | NA | 24 | 2590-2598 |
| Lee, NK; Kwon, WJ; Son, SM; et al. | The effects of closed and open kinetic chain exercises on lower limb muscle activity and balance in stroke survivors | NeuroRehabilitation | 2013 | 1 | NA | 177-183 |
| Lee, SH; Byun, SD; Kim, CH; et al. | Feasibility and effects of newly developed balance control trainer for mobility and balance in chronic stroke patients: a randomized controlled trial | Annals of Rehabilitation Medicine | 2012 | 4 | 36 | 521 |
| Lee, SW; Cho, KH; Lee, WH | Effect of a local vibration stimulus training programme on postural sway and gait in chronic stroke patients: a randomized controlled trial | Clinical Rehabilitation | 2013 | 10 | 27 | 921-931 |
| Liang, CC; Hsieh, TC; Lin, CH; et al. | Effectiveness of thermal stimulation for the moderately to severely paretic leg after stroke: serial changes at one-year follow-up | Archives of Physical Medicine and Rehabilitation | 2012 | 11 | 93 | 1903-1910 |
| Lin, Q; Chen, A; Cheng, K | Effects of acupuncture on motor function, balance function and activities of daily living of patients with Stroke | Chinese Journal of Rehabilitation Medicine | 2015 | 9 | 30 | 898-901 and 906 |
| Lindvall, MA; Forsberg, A | Body awareness therapy in persons with stroke: a pilot randomized controlled trial | Clinical Rehabilitation | 2014 | 12 | 28 | 1180-1188 |
| Lisinski, P; Huber, J; Gajewska, E; et al. | The body balance training effect on improvement of motor functions in paretic extremities in patients after stroke. A randomized, single blinded trial | Clinical Neurology and Neurosurgery | 2012 | 1 | 114 | 31-36 |
| Liu-Ambrose, T; Eng, JJ | Exercise Training and Recreational Activities to Promote Executive Functions in Chronic Stroke: A Proof-of-concept Study | Journal of Stroke and Cerebrovascular Diseases | 2015 | 1 | 24 | 130-137 |
| Lu, CL; Yu, B; Basford, JR; et al. | Influences of cane length on the stability of stroke patients | Journal of Rehabilitation Research and Development | 1997 | 1 | 34 | 91-100 |
| Lynch, EA; Hillier, SL; Stiller, K; et al. | Sensory retraining of the lower limb after acute stroke: a randomized controlled pilot trial | Archives of Physical Medicine and Rehabilitation | 2007 | 9 | 88 | 1101-1107 |
| Marin, PJ; Ferrero, CM; Menéndez, H; et al. | Effects of whole-body vibration on muscle architecture, muscle strength, and balance in stroke patients: a randomized controlled trial | American Journal of Physical Medicine & Rehabilitation | 2013 | 10 | 92 | 881-888 |
| Merkert, J; Butz, S; Nieczaj, R; et al. | Combined whole body vibration and balance training using Vibrosphere: Improvement of trunk stability, muscle tone, and postural control in stroke patients during early geriatric rehabilitation | Zeitschrift fur Gerontologie und Geriatrie | 2011 | 4 | 44 | 256-261 |
| Milczarek, JJ; Kirby, RL; Harrison, ER; MacLeod, DA | Standard and four-footed canes: their effect on the standing balance of patients with hemiparesis | Archives of Physical Medicine and Rehabilitation | 1993 | 3 | 74 | 281-285 |
| Mojica, JA; Nakamura, R; Kobayashi, T; et al.. | Effect of ankle-foot orthosis (AFO) on body sway and walking capacity of hemiparetic stroke patients | The Tohoku Journal of Experimental Medicine | 1988 | 4 | 156 | 395-401 |
| Moore, JL; Roth, EJ; Killian, C; Hornby, TG | Locomotor Training Improves Daily Stepping Activity and Gait Efficiency in Individuals Poststroke Who Have Reached a “Plateau" in Recovery | Stroke | 2010 | 1 | 41 | 129-135 |
| Morioka, S; Yagi, F | Effects of perceptual learning exercises on standing balance using a hardness discrimination task in hemiplegic patients following stroke: a randomized controlled pilot trial | Clinical Rehabilitation | 2003 | 6 | 17 | 600-607 |
| Mudie, MH; Winzeler-Mercay, U; Radwan, S; Lee, L | Training symmetry of weight distribution after stroke: a randomized controlled pilot study comparing task-related reach, Bobath and feedback training approaches | Clinical Rehabilitation | 2002 | 6 | 16 | 582-592 |
| Nadeau, SE; Wu, SS; Dobkin, Bruce H; et al. | Effects of task-specific and impairment-based training compared with usual care on functional walking ability after inpatient stroke rehabilitation: LEAPS trial | Neurorehabilitation and Neural Repair | 2013 | 4 | 27 | 370-380 |
| Ng, SSM; Lai, CWK; Tang, MWS; et al. | Cutaneous electrical stimulation to improve balance performance in patients with sub-acute stroke: a randomised controlled trial | Hong Kong medical journal | 2016 | Suppl 2 | 22 | S33-6 |
| Nikamp, CDM; Buurke, JH; van der Palen, J; et al. | Early or delayed provision of an ankle-foot orthosis in patients with acute and subacute stroke: a randomized controlled trial | Clinical Rehabilitation | 2017 | 6 | 31 | 798-808 |
| Noh, DK; Lim, JY; Shin, HI; et al. | The effect of aquatic therapy on postural balance and muscle strength in stroke survivors - a randomized controlled pilot trial | Clinical Rehabilitation | 2008 | 10-11 | 22 | 966-976 |
| Ordahan, B; Karahan, AY; Basaran, A; et al. | Impact of exercises administered to stroke patients with balance trainer on rehabilitation results: a randomized controlled study | Hippokratia | 2015 | 2 | 19 | 125-130 |
| Page, SJ; Levine, P; Teepen, J; et al. | Resistance-based, reciprocal upper and lower limb locomotor training in chronic stroke: a randomized, controlled crossover study | Clinical Rehabilitation | 2008 | 7 | 22 | 610-617 |
| Park, D; Lee, JH; Kang, TW; et al. | Immediate effects of talus-stabilizing taping on balance and gait parameters in patients with chronic stroke: a cross-sectional study | Topics in Stroke Rehabilitation | 2018 | 6 | 25 | 417-423 |
| Park, DS; Lee, DG; Lee, K; et al. | Effects of Virtual Reality Training using Xbox Kinect on Motor Function in Stroke Survivors: A Preliminary Study | Journal of Stroke and Cerebrovascular Diseases | 2017 | 10 | 26 | 2313-2319 |
| Park, HK; Lee, HJ; Lee, SJ; et al. | Land-based and acquatic trunk exercise program improve trunk control, balance and activities of daily living ability in stroke: a randomized clinical trial | European Journal of Physical and Rehabilitation Medicine | 2018 | NA | NA | NA |
| Park, J; Gong, J; Yim, J | Effects of a sitting boxing program on upper limb function, balance, gait, and quality of life in stroke patients | NeuroRehabilitation | 2017 | 1 | 40 | 77-86 |
| Park, J; Seo, D; Choi, W; Lee, S | The effects of exercise with TENS on spasticity, balance, and gait in patients with chronic stroke: a randomized controlled trial | Medical Science Monitor | 2014 | NA | 20 | 1890-1896 |
| Pollock, AS; Durward, BR; Rowe, PJ; Paul, JP | The effect of independent practice of motor tasks by stroke patients: a pilot randomized controlled trial | Clinical Rehabilitation | 2002 | 5 | 16 | 473-480 |
| Pomeroy, V M; Evans, B; Falconer, M; Jones, D; Hill, E; Giakas, G | An exploration of the effects of weighted garments on balance and gait of stroke patients with residual disability | Clinical Rehabilitation | 2001 | 4 | 15 | 390-397 |
| Rajaratnam, BS; Gui KJ; Lee JK; et al. | Does the inclusion of virtual reality games within conventional rehabilitation enhance balance retraining after a recent episode of stroke? | Rehabilitation Research and Practice | 2013 | NA | 2013 |  |
| Robertson, JA; Eng, JJ; Hung, C | The effect of functional electrical Stimulation on balance function and balance confidence in community-dwelling individuals with stroke | Physiotherapy Canada | 2010 | 2 | 62 | 114-119 |
| Rougier, P; Boudrahem, S | Effects of visual feedback of center-of-pressure displacements on undisturbed upright postural control of hemiparetic stroke patients | Restorative Neurology and Neuroscience | 2010 | 6 | NA | 749-759 |
| Salgueiro, C; Marquez, J | Influencia del entrenamiento visual en el control postural de pacientes con accidente cerebrovascular crónico: estudio piloto aleatorizado controlado | Fisioterapia | 2018 | 6 | 40 | 284-290 |
| Sánchez-Mila, Z; Salom-Moreno, J; Fernández-de-las-Peñas, C | Effects of Dry Needling on Post-Stroke Spasticity, Motor Function and Stability Limits: A Randomised Clinical Trial | Acupuncture in Medicine | 2018 | 6 | 36 | 358-366 |
| Schmid, AA; Van Puymbroeck, M; Altenburger, PA.; et al. | Poststroke balance improves with yoga: a pilot study | Stroke | 2012 | 9 | 43 | 2402-2407 |
| Schuster, C; Butler, J; Andrews, B; et al. | Comparison of embedded and added motor imagery training in patients after stroke: results of a randomised controlled pilot trial | Trials | 2012 | 1 | 13 |  |
| Shatil, S; Ivanova, TD; Mochizuki, G; Garland, SJ | Effects of therapeutic golf rehabilitation on golf performance, balance, and quality of life in individuals following stroke: pilot study | Physiotherapy Canada | 2005 | 2 | 57 | 101 |
| Shin, DC; Song, CH | Smartphone-Based Visual Feedback Trunk Control Training Using a Gyroscope and Mirroring Technology for Stroke Patients: Single-blinded, Randomized Clinical Trial of Efficacy and Feasibility | American Journal of Physical Medicine & Rehabilitation | 2016 | 5 | 95 | 319-29 |
| Simons, CDM; van Asseldonk, EHF; Kooij, H; et al. | Ankle-foot orthoses in stroke: Effects on functional balance, weight-bearing asymmetry and the contribution of each lower limb to balance control | Clinical Biomechanics | 2009 | 9 | 24 | 769-775 |
| Sohn, MK; Jee, SJ; Hwang, P; et al. | The Effects of Shoulder Slings on Balance in Patients With Hemiplegic Stroke | Annals of Rehabilitation Medicine | 2015 | 6 | 39 | 986 |
| Song, YB; Chun, MH; Kim, W; et al. | The effect of virtual reality and tetra-ataxiometric posturography programs on stroke patients with impaired standing balance | Annals of Rehabilitation Medicine | 2014 | 2 | 38 | 160 |
| Stein, J; Bishop, L; Stein, DJ; et al. | Gait Training with a Robotic Leg Brace After Stroke: A Randomized Controlled Pilot Study | American Journal of Physical Medicine & Rehabilitation | 2014 | 11 | 93 | 987-994 |
| Suh, HR; Han, HC; Cho, HY | Immediate therapeutic effect of interferential current therapy on spasticity, balance, and gait function in chronic stroke patients: a randomized control trial | Clinical Rehabilitation | 2014 | 9 | 28 | 885-891 |
| Tan, Z; Liu, H; Yan, T; et al. | The effectiveness of functional electrical stimulation based on a normal gait pattern on subjects with early stroke: a randomized controlled trial | BioMed Research International | 2014 | NA | 2014 |  |
| Tan, ZM; Jiang, WW; Yan, TB ; et al. | [Effects of functional electrical stimulation based on normal gait pattern on walking function in subjects with recovery of stroke] | Zhonghua Yi Xue Za Zhi (Chinese medical journal) | 2016 | 29 | 96 | 2342-2346 |
| Tian, FL; Li, Q; Liu, GR; et al. | Impacts of yin-yang meridians acupuncture with respiratory reinforcing and reducing manipulation on lower limbs balance function in stroke patients | Chinese Acupuncture & Moxibustion | 2014 | 11 | 34 | 1047-1050 |
| Tilikete, C; Rode, G; Rossetti, Y; et al. | Prism adaptation to rightward optical deviation improves postural imbalance in left-hemiparetic patients | Current biology | 2001 | 7 | 11 | 524-528 |
| Tripp, F; Krakow, K | Effects of an aquatic therapy approach (Halliwick-Therapy) on functional mobility in subacute stroke patients: a randomized controlled trial | Clinical Rehabilitation | 2014 | 5 | 28 | 432-439 |
| Tung, FL; Yang, YR; Lee, CC; Wang, RY | Balance outcomes after additional sit-to-stand training in subjects with stroke: a randomized controlled trial | Clinical Rehabilitation | 2010 | 6 | 24 | 533-542 |
| Vahlberg, B; Cederholm, T; Lindmark, B; et al. | Short-term and long-term effects of a progressive resistance and balance exercise program in individuals with chronic stroke: a randomized controlled trial | Disability and Rehabilitation | 2017 | 16 | 39 | 1615-1622 |
| Van Nes, IJW; Latour, H; Schils, F; et al. | Long-term effects of 6-week whole-body vibration on balance recovery and activities of daily living in the postacute phase of stroke: a randomized, controlled trial | Stroke | 2006 | 9 | 37 | 2331-2335 |
| Waldron, RM; Bohannon, RW | Weight distribution when standing: The influence of a single point cane in patients with stroke | Physiotherapy Practice | 1989 | 4 | 5 | 171-175 |
| Wang, H; Zhao, Z; Jiang, P; et al. | Effect and mechanism of mirror therapy on rehabilitation of lower limb motor function in patients with stroke hemiplegia | Biomed Research | 2017 | 22 | 28 | 6 |
| Wang, RY; Lin, PY; Lee, CC; Yang, YR | Gait and balance performance improvements attributable to ankle-foot orthosis in subjects with hemiparesis: | American Journal of Physical Medicine & Rehabilitation | 2007 | 7 | 86 | 556-562 |
| Wang, RY; Yen, LL; Lee, CC; et al. | Effects of an ankle-foot orthosis on balance performance in patients with hemiparesis of different durations | Clinical Rehabilitation | 2005 | 1 | 19 | 37-44 |
| Wang, TC; Tsai, AC; Wang, JY; et al. | Caregiver-mediated intervention can improve physical functional recovery of patients with chronic stroke: a randomized controlled trial | Neurorehabilitation and Neural Repair | 2015 | 1 | 29 | 3-12 |
| Xie, G; Rao, T; Lin, L; et al. | Effects of Tai Chi Yunshou exercise on community-based stroke patients: a cluster randomized controlled trial | European Review of Aging and Physical Activity | 2018 | 1 | 15 | NA |
| Xing, J; Wang, YJ; Li, YR | Clinical study on acupuncture combined with hyperbaric oxygenation for improving balance function of cerebral infarction | Chinese Acupuncture & Moxibustion | 2007 | 1 | 27 | 12-14 |
| Yadav, R; Walia, S; Vats, M; et al. | Comparison of Effectiveness of Specific Balance Strategy Training Programme with General Balance Training Programme on Balance Performance in Chronic Stroke | Fiziksel Tip ve Rehabilitasyon Bilimleri Dergisi [Journal of Physical Medicine and Rehabilitation Sciences] | 2016 | 1 | 19 | 1-6 |
| Yeung, LF; Ockenfeld, C; Pang, MK; et al. | Randomized controlled trial of robot-assisted gait training with dorsiflexion assistance on chronic stroke patients wearing ankle-foot-orthosis | Journal of NeuroEngineering and Rehabilitation | 2018 | 1 | 15 | NA |
| Yoo, HJ; Pyun, SB | Efficacy of Bedside Respiratory Muscle Training in Patients With Stroke: A Randomized Controlled Trial | American Journal of Physical Medicine & Rehabilitation | 2018 | 10 | 97 | 691-697 |
| Yoo, SD; Jeong, YS; Kim, DH; et al. | The efficacy of core strengthening on the trunk balance in patients with subacute stroke | Annals of Rehabilitation Medicine | 2010 | 6 | 34 | 677-682 |
| You, G; Liang, H; Yan, T | Functional electrical stimulation early after stroke improves lower limb motor function and ability in activities of daily living | NeuroRehabilitation | 2014 | 3 | NA | 381-389 |
| Yu, JH; Cho, KH | Effectiveness of Virtual Reality Game on Functional Movement and Activities of Daily Living in Hemiparetic Stroke Patients | Journal of Nanoelectronics and Optoelectronics | 2016 | 1 | 11 | 98-102 |
| Yun, N; Joo, MC; Kim, SC; et al. | Robot-assisted gait training effectively improved lateropulsion in subacute stroke patients: a single-blinded randomized controlled trial | European Journal of Physical and Rehabilitation Medicine | 2019 | 6 | 54 | 827-836 |
| Zhang, WM; Yang, S; Wang, YJ; et al. | Effect of modified constraint-induced movement therapy on the activities of daily living of patients with acute stroke | Chinese Journal of Contemporary Neurology and Neurosurgery | 2015 | 4 | 15 | 280-204 |
