# Supplementary material for: Limited evidence of physical therapy on balance after stroke: A systematic review and meta-analysis
Source: PLoS One. 2019 Aug 29;14(8):e0221700. doi: 10.1371/journal.pone.0221700 (PMC6715189; doi:10.1371/journal.pone.0221700)
Supplement: S3 Table — (DOCX) [file pone.0221700.s017.docx]

**S3 Table. Characteristics of studies and participants**

**S3A Table. Summary of characteristics of studies and participants**

|  | Both comparisons  (PT versus NT and ST/UC) | PT versus NT | PT versus ST/UC |
| --- | --- | --- | --- |
| Trials / comparisons | 145 / 172 | 76 / 91 | 70 / 81 |
| Date of publication | From 1988 to 2019 | From 1988 to 2019 | From 1998 to 2018 |
| Crossover / parallel group | 18 / 127 | 18 / 58 | 0 / 70 |
| Trials with 2, 3 or 4 groups | 121 / 21 / 3 | 63 / 10 / 3 | 58 / 12 / 0 |
| Participants (sum, mean +/- sd, min-max) | 5912 / 40.8 +/- 42.9 / 7-408 | 3049 / 40.1 +/- 48.3 / 8-408 | 2899 / 41.4 +/- 36.3 / 7-244 |
| Age of participants (years) (wtd mean +/- wtd sd, min-max) | 60.8 +/- 44.3 / 46.9-78.5  (on 136 trials, 5730 subjects) | 61.3 +/- 46.7 / 50.0-78.54  (on 70 trials, 2916 subjects) | 60.1 +/- 41.8 / 46.9-73.9  (on 67 trials, 2850 subjects) |
| Men / Women (percent) | 61% / 39% | 59% / 41% | 63% / 37% |
| Time post-stroke (days) (wtd mean +/- wtd sd, min-max) | 193.7 +/- 322.4 / 4.5-1985.6  (on 122 trials, 5258 subjects) | 152.5 +/- 282.1 / 4.5-1716.7  (on 60 trials, 2653 subjects) | 282.0 +/- 461.7 / 11-1985.6  (on 62 trials, 2695 subjects) |
| Location of stroke lesion | | | |
| Only supratentorial stroke (trials/participants) | 18 / 656 | 10 / 297 | 9 / 395 |
| Only brainstem stroke (trials/participants) | 0 / 0 | 0 / 0 | 0 / 0 |
| Only cerebellum stroke (trials/participants) | 0 / 0 | 0 / 0 | 0 / 0 |
| Only other stroke (trials/participants) | 0 / 0 | 0 / 0 | 0 / 0 |
| Mixed different location of stroke or not determined (trials/participants) | 127 / 5256 | 66 / 823 | 61 / 2504 |
| Episode of stroke | | | |
| Only first episode (trials/participants) | 68 / 3133 | 34 / 1612 | 35 / 1557 |
| Only multiple episodes (trials/participants) | 1/ 30 | 0 / 0 | 1 / 30 |
| First or multiple episodes (trials/participants) | 12 / 604 | 8 / 406 | 4 / 198 |
| Not determined (trials/participants) | 64 / 2145 | 34 / 1031 | 30 / 1114 |
| Side of stroke lesion | | | |
| Only unilateral stroke (trials/participants) | 117 / 4683 | 61 / 2416 | 57 / 2303 |
| Only bilateral stroke (trials/participants) | 0 / 0 | 0 / 0 | 0 / 0 |
| Unilateral or bilateral stroke (trials/participants) | 6 / 455 | 2 / 87 | 4 / 368 |
| Not determined (trials/participants) | 22 / 774 | 13 / 546 | 9 / 228 |
| Etiology of stroke | | | |
| Only ischemic stroke (trials/participants) | 12 / 379 | 6 / 224 | 7 / 191 |
| Only hemorrhagic stroke (trials/participants) | 0 / 0 | 0 / 0 | 0 / 0 |
| Only ischemic or hemorrhagic stroke (trials/participants) | 90 / 3589 | 43 / 1597 | 47 / 1992 |
| Other stroke or not determined (trials/participants) | 43 / 1944 | 27 / 1228 | 16 / 716 |
| Stage of stroke for eligibility or inclusion of participants in trials | | | |
| Only acute stroke (trials/participants) | 11 / 389 | 7 / 240 | 4 / 149 |
| Only subacute stroke (trials/participants) | 8 / 759 | 6 / 647 | 3 / 148 |
| Only chronic stroke (trials/participants) | 57 / 1910 | 24 / 686 | 33 / 1224 |
| Mixed stages or not determined (trials/participants) | 69 / 2854 | 39 / 1476 | 30 / 1378 |
| Description of stroke lesion using brain imagery | | | |
| No imagery used (trials/participants) | 90 / 3193 | 46 / 1526 | 44 / 1667 |
| Use of imagery reported but without description of lesion (trials/participants) | 44 / 2421 | 24 / 1381 | 21 / 1076 |
| Imagery used with description of lesion in text (trials/participants) | 11 / 298 | 6 / 142 | 5 / 156 |
| Ethic | | | |
| Consultation of ethic committee (trials/participants) | 115 / 4807 | 57 / 2245 | 59 / 2598 |
| Respect of Helsinki declaration (trials/participants) | 24 / 1407 | 10 / 840 | 14 / 567 |

Abbreviations: min, minimum; max, maximum; NA, not applicable; NT, no treatment; PT, physical therapy; ST, sham treatment; sd, standard deviation; UC, usual care; wtd, weighted.

**S3B Table. Characteristics of studies and participants for each study included**

| Study | Date | Age of participant | | Participants | Man | | Woman | | Size of group (Num) | | | | Type of RCT |
| --- | --- | --- | --- | --- | --- | --- | --- | --- | --- | --- | --- | --- | --- |
|  |  | Mean (y) | SD (y) | Num | Num | Percent | Num | Percent | A | B | C | D |  |
| Allison et Dennett, 2007 | 2007 | 75.69 | 12.78 | 17 | nd | nd | nd | nd | 7 | NA | 10 | NA | PG |
| Arabzadeh et al., 2018 | 2018 | 59.25 | 19.10 | 20 | 15 | 75.00 | 5 | 25.00 | 10 | NA | 10 | NA | CO |
| Askim et al., 2010 | 2010 | 76.54 | 8.82 | 62 | 29 | 47 | 33 | 53 | 30 | NA | 32 | NA | PG |
| Au-Yeung et al., 2009 | 2009 | 64.51 | 10.74 | 136 | 79 | 58.09 | 57 | 41.91 | 74 | NA | 62 | NA | PG |
| Bae et al., 2015 | 2015 | 64.29 | 7.71 | 30 | nd | nd | nd | nd | 15 | NA | 15 | NA | CO |
| Barcala et al., 2011 | 2011 | 58.00 | 12.57 | 12 | 5 | 42 | 7 | 58 | 6 | NA | 6 | NA | PG |
| Brogardh et al., 2012 | 2012 | 62.00 | 7.00 | 31 | 25 | 81 | 6 | 19 | 16 | NA | 15 | NA | PG |
| Bunketorp-Kall et al., 2017 | 2017 | 63.00 | 6.60 | 123 | 69 | 56.10 | 54 | 43.90 | 41 | 41 | 41 | NA | CO |
| Buyukavci et al., 2016 | 2016 | 63.09 | 10.38 | 65 | 33 | 50.77 | 32 | 49.23 | 33 | NA | 32 | NA | PG |
| Büyükvural Şen et al., 2015 | 2015 | 53.35 | 11.35 | 50 | 33 | 66 | 17 | 34 | 25 | NA | 25 | NA | PG |
| Cabanas-Valdés et al., 2015 | 2015 | 75.31 | 10.01 | 80 | 40 | 50 | 40 | 50 | 40 | NA | 40 | NA | PG |
| Chan KS et al., 2012 | 2012 | 55.50 | 9.27 | 30 | 21 | 70 | 9 | 30 | 15 | NA | 15 | NA | PG |
| Chen CH et al., 2010 | 2010 | 57.50 | 10.44 | 10 | 10 | 100 | 0 | 0 | 10 | 10 | 10 | NA | CO |
| Chen CL et al., 2015 | 2015 | 57.10 | 12.70 | 24 | 13 | 54 | 11 | 46 | 24 | NA | 24 | NA | CO |
| Chen D et al., 2014 | 2014 | 59.31 | 9.6 | 48 | 27 | 56 | 21 | 44 | 18 | 15 | 15 | NA | PG |
| Chen IC et al., 2002 | 2002 | 57.22 | 10.91 | 41 | 13 | 32 | 28 | 68 | 23 | NA | 18 | NA | PG |
| Chen JC et al., 2011 | 2011 | 60.08 | 11.43 | 33 | 22 | 67 | 11 | 33 | 17 | NA | 16 | NA | PG |
| Chen, 2018 | 2018 | nd | nd | 16 | nd | nd | nd | nd | 8 | NA | 8 | NA | CO |
| Chern et al., 2013 | 2013 | nd | nd | 15 | 11 | 73 | 4 | 27 | 15 | 15 | 15 | 15 | CO |
| Cho HY et al., 2013 | 2013 | 55.41 | 10.10 | 42 | 27 | 64 | 15 | 36 | 22 | NA | 20 | NA | PG |
| Cho KH et al., 2012 | 2012 | 64.20 | 7.54 | 22 | 14 | 64 | 8 | 36 | 11 | NA | 11 | NA | PG |
| Cho MK et al., 2015 | 2015 | 56.00 | 8.69 | 31 | 19 | 61 | 12 | 39 | 10 | 10 | 11 | NA | PG |
| Choi HS et al., 2017 | 2017 | 61.92 | 5.59 | 36 | 21 | 58.33 | 15 | 41.67 | 12 | 12 | 12 | NA | CO |
| Chu et al., 2015 | 2015 | 67.03 | 10.99 | 145 | 89 | 61.38 | 56 | 38.62 | 48 | 49 | 48 | NA | PG |
| Chung et al., 2014 | 2014 | 58.30 | 7.53 | 18 | 14 | 78 | 4 | 22 | 9 | NA | 9 | NA | PG |
| Dault et al., 2003 | 2003 | 57.80 | 10.80 | 10 | 5 | 50 | 5 | 50 | 10 | NA | 10 | NA | CO |
| Dujovic et al., 2017 | 2017 | nd | nd | 16 | 10 | 62.50 | 6 | 37.50 | 8 | NA | 8 | NA | CO |
| Duncan et al., 1998 | 1998 | 67.55 | 8.26 | 20 | nd | nd | nd | nd | 10 | NA | 10 | NA | PG |
| Duncan et al., 2003 | 2003 | 69.39 | 10.30 | 92 | 50 | 54 | 42 | 46 | 44 | NA | 48 | NA | PG |
| Erbil et al., 2018 | 2018 | 49.64 | 11.26 | 43 | 27 | 62.79 | 16 | 37.21 | 29 | NA | 14 | NA | PG |
| Fernandez-Gonzalo et al., 2016 | 2016 | 63.53 | 11.42 | 29 | 22 | 75.86 | 7 | 24.14 | 14 | NA | 15 | NA | PG |
| Ferreira et al., 2017 | 2017 | 59.20 | 10.40 | 20 | 14 | 70.00 | 6 | 30.00 | 12 | NA | 8 | NA | PG |
| Fritz et al., 2013 | 2013 | 66.16 | 9.63 | 28 | nd | nd | nd | nd | 15 | NA | 13 | NA | PG |
| Furnari et al., 2014 | 2014 | 70.00 | 6.00 | 40 | 20 | 50.00 | 20 | 50.00 | 20 | NA | 20 | NA | PG |
| Geiger et al., 2001 | 2001 | 60.38 | 15.39 | 13 | 9 | 69.23 | 4 | 30.77 | 7 | NA | 6 | NA | PG |
| Ghanjal et al., 2014 | 2014 | 54.43 | 7.39 | 36 | 26 | 72 | 10 | 28 | 12 | 12 | 12 | NA | PG |
| Globas et al., 2012 | 2012 | 68.70 | 6.30 | 36 | 29 | 81 | 7 | 19 | 18 | NA | 18 | NA | PG |
| Goliwas et al., 2017 | 2017 | 63.42 | 8.74 | 37 | 21 | 56.76 | 16 | 43.24 | 20 | NA | 17 | NA | PG |
| Han et al., 2016 | 2016 | 65.71 | 13.22 | 56 | 32 | 57.14 | 24 | 42.86 | 30 | NA | 26 | NA | PG |
| Hart et al., 2004 | 2004 | 54.77 | nd | 18 | 16 | 89 | 2 | 11 | 9 | NA | 9 | NA | PG |
| Heller et al., 2005 | 2005 | 60.42 | 12.80 | 26 | 15 | 58 | 11 | 42 | 13 | NA | 13 | NA | PG |
| Hollands et al., 2015 | 2015 | 58.36 | 14.57 | 56 | 33 | 58.93 | 23 | 41.07 | 18 | 19 | 19 | NA | PG |
| Holmgren et al., 2010 | 2010 | 78.54 | 7.47 | 34 | 21 | 61.76 | 13 | 38.24 | 15 | NA | 19 | NA | PG |
| Hosseini et al., 2012 | 2012 | 48.10 | 10.50 | 30 | 16 | 53.33 | 14 | 46.67 | 15 | NA | 15 | NA | PG |
| Howe et al., 2005 | 2005 | 71.09 | 9.22 | 35 | 18 | 51 | 17 | 49 | 17 | NA | 18 | NA | PG |
| Hsieh, 2019 | 2019 | 58.80 | 12.00 | 56 | 33 | 58.93 | 23 | 41.07 | 28 | NA | 28 | NA | CO |
| Hsu et al., 2013 | 2013 | 51.88 | 13.35 | 23 | 14 | 61 | 9 | 39 | 11 | NA | 12 | NA | PG |
| Huh et al., 2015 | 2015 | 65.84 | 5.88 | 40 | 26 | 65.00 | 14 | 35.00 | 23 | NA | 17 | NA | PG |
| Hung et al., 2016 | 2016 | 53.92 | 13.24 | 23 | 16 | 69.57 | 7 | 30.43 | 12 | NA | 11 | NA | PG |
| Hwang et al., 2015 | 2015 | 49.74 | 6.30 | 30 | 17 | 57 | 13 | 43 | 15 | NA | 15 | NA | PG |
| Immink et al., 2014 | 2014 | 59.60 | 15.70 | 22 | 9 | 41 | 13 | 59 | 11 | NA | 11 | NA | PG |
| In et al., 2016 | 2016 | 55.92 | 10.85 | 25 | 15 | 60.00 | 10 | 40.00 | 13 | NA | 12 | NA | PG |
| Janssen et al., 2008 | 2008 | 54.75 | 10.08 | 12 | 6 | 50 | 6 | 50 | 6 | NA | 6 | NA | PG |
| Jung et al., 2015 | 2015 | 53.59 | 13.14 | 22 | 11 | 50 | 11 | 50 | 11 | NA | 11 | NA | PG |
| Jung et al., 2017 | 2017 | 56.25 | 10.17 | 40 | 23 | 57.50 | 17 | 42.50 | 20 | NA | 20 | NA | CO |
| Kamps et Schule, 2005 | 2005 | 64.41 | 9.39 | 31 | 22 | 71 | 9 | 29 | 16 | NA | 15 | NA | PG |
| Karasu et al., 2018 | 2018 | 63.20 | 11.70 | 23 | 10 | 43.48 | 13 | 56.52 | 12 | NA | 11 | NA | PG |
| Katz-Leurer et al., 2006 | 2006 | 62.50 | 8.94 | 24 | 13 | 54 | 11 | 46 | 10 | NA | 14 | NA | PG |
| Khumsapsiri et al., 2018 | 2018 | 59.25 | 10.02 | 16 | 7 | 43.75 | 9 | 56.25 | 8 | NA | 8 | NA | CO |
| Kilinc et al., 2015 | 2015 | 55.08 | 10.62 | 22 | 9 | 40.91 | 13 | 59.09 | 12 | NA | 10 | NA | PG |
| Kim DH et al., 2008 | 2008 | 52.44 | 10.20 | 16 | 7 | 88 | 1 | 13 | 8 | NA | 8 | NA | PG |
| Kim JC et Lee, 2018 | 2018 | 55.10 | 7.84 | 21 | 19 | 90.48 | 2 | 9.52 | 11 | NA | 10 | NA | PG |
| Kim JH et al., 2009 | 2009 | 51.96 | 8.40 | 24 | 13 | 54 | 11 | 46 | 12 | NA | 12 | NA | PG |
| Kim JY et al., 2018 | 2018 | 58.99 | 12.98 | 48 | 33 | 68.75 | 15 | 31.25 | 25 | NA | 23 | NA | PG |
| Kim SL et Lee, 2018 | 2018 | 46.94 | 12.90 | 30 | 18 | 60.00 | 12 | 40.00 | 15 | NA | 15 | NA | CO |
| Kim YH et al., 2004 | 2004 | 58.85 | 25.32 | 38 | 24 | 63 | 14 | 37 | 13 | 13 | 12 | NA | PG |
| Kim YM et al., 2009 | 2009 | 64.80 | 12.74 | 32 | 17 | 53 | 15 | 47 | 16 | NA | 16 | NA | PG |
| Knox et al., 2018 | 2018 | 50.00 | 13.76 | 144 | 72 | 50.00 | 72 | 50.00 | 51 | 45 | 48 | NA | PG |
| Kunkel et al., 2013 | 2013 | 68.37 | 14.90 | 21 | 12 | 57 | 9 | 43 | 7 | 7 | 7 | NA | PG |
| Kwong et al., 2018 | 2018 | 62.00 | 5.40 | 80 | 50 | 62.50 | 30 | 37.50 | 40 | NA | 40 | NA | CO |
| Langhammer et al., 2009 | 2009 | 73.87 | 13.25 | 67 | 43 | 57 | 32 | 43 | 32 | NA | 35 | NA | PG |
| Lau RWK et al., 2012 | 2012 | 57.35 | 11.13 | 82 | 58 | 71 | 24 | 29 | 41 | NA | 41 | NA | PG |
| Laufer, 2003 | 2003 | 71.20 | 7.00 | 30 | 18 | 60 | 12 | 40 | 30 | 30 | 30 | NA | CO |
| Lee CH et al., 2014 | 2014 | 51.10 | 12.06 | 21 | 14 | 67 | 7 | 33 | 10 | NA | 11 | NA | PG |
| Lee D et al., 2016 | 2016 | 54.70 | 6.47 | 27 | 14 | 51.85 | 13 | 48.15 | 14 | NA | 13 | NA | PG |
| Lee HJ et al., 2018 | 2018 | 60.00 | 7.41 | 20 | 10 | 50.00 | 10 | 50.00 | 10 | NA | 10 | NA | CO |
| Lee MM et al., 2018 | 2018 | 61.57 | 7.53 | 30 | 18 | 60.00 | 12 | 40.00 | 15 | NA | 15 | NA | CO |
| Lee NK et al., 2013 | 2013 | 59.40 | 7.40 | 33 | 20 | 61 | 13 | 39 | 11 | 11 | 11 | NA | PG |
| Lee SH et al., 2012 | 2012 | 53.93 | 11.07 | 40 | 25 | 63 | 15 | 38 | 20 | NA | 20 | NA | PG |
| Lee SW et al., 2013 | 2013 | 54.48 | 8.27 | 31 | 24 | 77 | 7 | 23 | 16 | NA | 15 | NA | PG |
| Liang et al., 2012 | 2012 | 57.92 | 11.69 | 30 | 19 | 63 | 11 | 37 | 15 | NA | 15 | NA | PG |
| Lin Q et al., 2015 | 2015 | 60.95 | 7.53 | 64 | 41 | 64.06 | 23 | 35.94 | 32 | NA | 32 | NA | PG |
| Lindvall et Forsberg, 2014 | 2014 | 63.77 | 10.44 | 46 | 27 | 59 | 19 | 41 | 24 | NA | 22 | NA | PG |
| Lisinski et al., 2012 | 2012 | 64.00 | 16.40 | 26 | 10 | 38 | 16 | 62 | 13 | NA | 13 | NA | PG |
| Liu-Ambrose et Eng, 2015 | 2015 | 65.20 | 10.40 | 25 | 15 | 60 | 10 | 40 | 11 | NA | 14 | NA | PG |
| Lu et al., 1997 | 1997 | 58.90 | 6.90 | 10 | 10 | 100 | 0 | 0 | 10 | 10 | 10 | NA | CO |
| Lynch et al., 2007 | 2007 | 61.52 | 13.72 | 21 | 16 | 76 | 5 | 24 | 10 | NA | 11 | NA | PG |
| Marin et al., 2013 | 2013 | 63.25 | 9.20 | 20 | 11 | 55 | 9 | 45 | 11 | NA | 9 | NA | PG |
| Merkert et al., 2011 | 2011 | 74.50 | 8.39 | 66 | 22 | 33 | 44 | 67 | 33 | NA | 33 | NA | PG |
| Milczarek et al., 1993 | 1993 | 59.93 | 13.18 | 14 | 8 | 57 | 6 | 43 | 14 | NA | 14 | NA | CO |
| Mojica et al., 1988 | 1988 | nd | nd | 8 | 5 | 63 | 3 | 38 | 8 | NA | 8 | NA | CO |
| Moore JL et al., 2010 | 2010 | 50.00 | 15.00 | 20 | 14 | 70 | 6 | 30 | nd | NA | nd | NA | CO |
| Morioka et Yagi, 2003 | 2003 | 61.90 | 11.88 | 26 | 17 | 65 | 9 | 35 | 12 | NA | 14 | NA | PG |
| Mudie et al., 2002 | 2002 | 72.40 | 9.01 | 40 | 21 | 53 | 19 | 48 | 10 | 10 | 10 | 10 | PG |
| Nadeau et al., 2013 | 2013 | 61.99 | 12.73 | 408 | 224 | 55 | 184 | 45 | 139 | 126 | 143 | NA | PG |
| Ng et al., 2016 | 2016 | 70.10 | 9.90 | 76 | 48 | 63.16 | 28 | 36.84 | 37 | NA | 39 | NA | PG |
| Nikamp et al., 2017 | 2017 | 57.20 | 9.20 | 33 | 20 | 60.61 | 13 | 39.39 | 16 | NA | 17 | NA | PG |
| Noh et al., 2008 | 2008 | 63.87 | 10.72 | 25 | 11 | 44.00 | 14 | 56.00 | 13 | NA | 12 | NA | PG |
| Ordahan et al., 2015 | 2015 | 57.10 | 9.20 | 50 | 31 | 62.00 | 19 | 38.00 | 25 | NA | 25 | NA | CO |
| Page et al., 2008 | 2008 | 61.29 | 12.30 | 7 | 5 | 71.43 | 2 | 28.57 | 4 | NA | 3 | NA | PG |
| Park D et al., 2018 | 2018 | 58.50 | 10.07 | 20 | 12 | 60.00 | 8 | 40.00 | 20 | 20 | 20 | NA | CO |
| Park DS et al., 2017 | 2017 | 63.65 | 13.94 | 20 | 10 | 50.00 | 10 | 50.00 | 10 | NA | 10 | NA | CO |
| Park et al., 2014 | 2014 | 71.17 | 3.57 | 29 | 20 | 69 | 9 | 31 | 15 | NA | 14 | NA | PG |
| Park HK et al., 2018 | 2018 | 56.70 | 12.52 | 29 | 22 | 75.86 | 7 | 24.14 | 14 | NA | 15 | NA | PG |
| Park J et al., 2017 | 2017 | nd | nd | 30 | nd | nd | nd | nd | 15 | NA | 15 | NA | CO |
| Pollock et al., 2002 | 2002 | 69.91 | 12.50 | 28 | 12 | 43 | 16 | 57 | 9 | NA | 19 | NA | PG |
| Pomeroy et al., 2001 | 2001 | nd | nd | 24 | 18 | 75 | 6 | 25 | 12 | NA | 12 | NA | PG |
| Rajaratnam et al., 2013 | 2013 | 61.60 | 7.80 | 19 | 7 | 37 | 12 | 63 | 9 | NA | 10 | NA | PG |
| Robertson et al., 2010 | 2010 | 54.40 | 10.40 | 15 | 12 | 80 | 3 | 20 | 15 | NA | 15 | NA | CO |
| Rougier et Boudrahem, 2010 | 2010 | 61.79 | 11.42 | 39 | 24 | 62 | 15 | 38 | 39 | NA | 39 | NA | CO |
| Salgueiro et Marquez, 2018 | 2018 | 57.30 | 8.54 | 12 | 9 | 75.00 | 3 | 25.00 | 6 | NA | 6 | NA | PG |
| Sanchez-Mila et al., 2018 | 2018 | 57.00 | 12.00 | 26 | 13 | 50.00 | 13 | 50.00 | 14 | NA | 12 | NA | PG |
| Schmid et al., 2012 | 2012 | 63.10 | 8.80 | 47 | 38 | 81 | 9 | 19 | 37 | NA | 10 | NA | PG |
| Schuster et al., 2012 | 2012 | 63.42 | 10.21 | 39 | 27 | 69 | 12 | 31 | 13 | 14 | 12 | NA | PG |
| Shatil et al., 2005 | 2005 | 63.8 | 15.8 | 18 | 11 | 61 | 7 | 39 | 10 | NA | 8 | NA | PG |
| Shin et al., 2016 | 2016 | 58.50 | 11.84 | 24 | nd | nd | nd | nd | 12 | NA | 12 | NA | CO |
| Simons et al., 2009 | 2009 | 57.20 | 10.23 | 20 | 14 | 70 | 6 | 30 | 20 | NA | 20 | NA | CO |
| Sohn et al., 2015 | 2015 | 57.00 | 12.00 | 27 | 17 | 62.96 | 10 | 37.04 | 27 | NA | 27 | NA | CO |
| Song et al., 2014 | 2014 | 62.47 | 14.95 | 30 | 16 | 53 | 14 | 47 | 10 | 10 | 10 | NA | PG |
| Stein et al., 2014 | 2014 | 57.10 | 8.21 | 24 | 16.92 | 70.50 | 7.08 | 29.50 | 12 | NA | 12 | NA | PG |
| Suh et al., 2014 | 2014 | 54.13 | 12.10 | 42 | 29 | 69 | 13 | 31 | 21 | NA | 21 | NA | PG |
| Tan et al., 2014 | 2014 | 64.97 | 9.32 | 45 | 24 | 51 | 23 | 49 | 16 | 15 | 14 | NA | PG |
| Tan et al., 2016 | 2016 | 58.26 | 12.59 | 58 | 32 | 55.17 | 26 | 44.83 | 29 | 15 | 14 | NA | PG |
| Tian et al., 2014 | 2014 | 56.5 | 14.72 | 100 | 54 | 54 | 46 | 46 | 50 | NA | 50 | NA | PG |
| Tilikete et al., 2001 | 2001 | 54.67 | nd | 15 | 6 | 40 | 9 | 60 | 5 | 5 | 5 | NA | PG |
| Tripp and Krakow, 2014 | 2014 | 64.91 | 14.79 | 30 | 19 | 63 | 11 | 37 | 14 | NA | 16 | NA | PG |
| Tung et al., 2010 | 2010 | 51.85 | 12.95 | 32 | 20 | 63 | 12 | 38 | 16 | NA | 16 | NA | PG |
| Vahlberg et al., 2017 | 2017 | 73.14 | 5.39 | 67 | 51 | 76.12 | 16 | 23.88 | 34 | NA | 33 | NA | PG |
| VanNes et al., 2006 | 2006 | 61.12 | 10.27 | 53 | 30 | 57 | 23 | 43 | 27 | NA | 26 | NA | PG |
| Waldron et Bohannon, 1989 | 1989 | 63.20 | 10.10 | 20 | nd | nd | nd | nd | 20 | 20 | 20 | 20 | CO |
| Wang et al., 2017 | 2017 | 52.73 | 2.82 | 36 | 26 | 72.22 | 10 | 27.78 | 18 | NA | 18 | NA | CO |
| Wang RY, Lin PY et al., 2007 | 2007 | 60.36 | 13.95 | 58 | 44 | 76 | 14 | 24 | 58 | NA | 58 | NA | CO |
| Wang RY, Yen LL et al., 2005 part 1 | 2005 | 59.90 | 13.00 | 42 | 23 | 55 | 19 | 45 | 42 | NA | 42 | NA | CO |
| Wang RY, Yen LL et al., 2005 part 2 | 2005 | 62.30 | 11.80 | 61 | 51 | 84 | 10 | 16 | 61 | NA | 61 | NA | CO |
| Wang TC et al., 2015 | 2015 | 63.73 | 10.12 | 51 | 30 | 59 | 21 | 41 | 25 | NA | 26 | NA | PG |
| Xie et al., 2018 | 2018 | 60.49 | 8.64 | 244 | 182 | 74.59 | 62 | 25.41 | 120 | NA | 124 | NA | PG |
| Xing et al., 2007 | 2007 | 50.87 | 11.66 | 72 | 52 | 72 | 20 | 28 | 36 | NA | 36 | NA | PG |
| Yadav et al., 2015 | 2015 | 55.29 | 10.06 | 24 | 19 | 79.17 | 5 | 20.83 | 12 | NA | 12 | NA | CO |
| Yeung et al., 2018 | 2018 | 57.90 | 12.00 | 19 | 13 | 68.42 | 6 | 31.58 | 9 | NA | 10 | NA | PG |
| Yoo et al., 2010 | 2010 | 60.74 | 15.38 | 59 | 30 | 51 | 29 | 49 | 28 | NA | 31 | NA | PG |
| Yoo et al., 2018 | 2018 | 61.00 | nd | 40 | 26 | 65.00 | 14 | 35.00 | 20 | NA | 20 | NA | CO |
| You et al., 2014 | 2014 | 62.41 | 10.27 | 37 | 21 | 57 | 16 | 43 | 19 | NA | 18 | NA | PG |
| Yu et Cho, 2016 | 2016 | 63.75 | 5.36 | 20 | 12 | 60.00 | 8 | 40.00 | 10 | NA | 10 | NA | CO |
| Yun et al., 2018 | 2018 | 63.90 | 8.20 | 36 | 19 | 52.78 | 17 | 47.22 | 18 | NA | 18 | NA | CO |
| Zhang et al., 2015 | 2015 | 59.22 | 5.19 | 60 | 33 | 55 | 27 | 45 | 30 | NA | 30 | NA | PG |

Abbreviations: CO, crossover; NA, not applicable; nd, not documented; Num, number; PG, parallel group.

**S3C Table. Characteristics of participants for each study included**

| Study | Supratentorial stroke | | Right supratentorial stroke | | Left supratentorial stroke | | Brainstem stroke | | Cerebellum stroke | | Other location of stroke | |
| --- | --- | --- | --- | --- | --- | --- | --- | --- | --- | --- | --- | --- |
|  | Trial | Participant | Trial | Participant | Trial | Participant | Trial | Participant | Trial | Participant | Trial | Participant |
| Allison et Dennett, 2007 | nd | nd | nd | nd | nd | nd | nd | nd | nd | nd | nd | nd |
| Arabzadeh et al., 2018 | nd | nd | nd | nd | nd | nd | nd | nd | nd | nd | nd | nd |
| Askim et al., 2010 | nd | nd | nd | nd | nd | nd | nd | nd | nd | nd | nd | nd |
| Au-Yeung et al., 2009 | nd | nd | nd | nd | nd | nd | nd | nd | nd | nd | nd | nd |
| Bae et al., 2015 | nd | nd | nd | nd | nd | nd | nd | nd | nd | nd | nd | nd |
| Barcala et al., 2011 | nd | nd | nd | nd | nd | nd | nd | nd | nd | nd | nd | nd |
| Brogardh et al., 2012 | nd | nd | nd | nd | nd | nd | nd | nd | nd | nd | nd | nd |
| Bunketorp-Kall et al., 2017 | nd | nd | nd | nd | nd | nd | nd | nd | nd | nd | nd | nd |
| Buyukavci et al., 2016 | nd | nd | nd | nd | nd | nd | nd | nd | No | 0 | nd | nd |
| Büyükvural Şen et al., 2015 | nd | nd | nd | nd | nd | nd | nd | nd | nd | nd | nd | nd |
| Cabanas-Valdés et al., 2015 | Yes | 67 | Yes | 40 | Yes | 27 | Yes | 10 | Yes | 3 | No | 0 |
| Chan KS et al., 2012 | nd | nd | nd | nd | nd | nd | nd | nd | nd | nd | nd | nd |
| Chen CH et al., 2010 | Yes | 9 | Yes | 4 | Yes | 5 | Yes | 1 | No | 0 | No | 0 |
| Chen CL et al., 2015 | nd | nd | nd | nd | nd | nd | nd | nd | nd | nd | nd | nd |
| Chen D et al., 2014 | Yes | nd | nd | nd | nd | nd | nd | nd | nd | nd | No | 0 |
| Chen IC et al., 2002 | Yes | 41 | Yes | 21 | Yes | 20 | No | 0 | No | 0 | No | 0 |
| Chen JC et al., 2011 | nd | nd | nd | nd | nd | nd | nd | nd | nd | nd | nd | nd |
| Chen, 2018 | nd | nd | nd | nd | nd | nd | nd | nd | nd | nd | nd | nd |
| Chern et al., 2013 | nd | nd | nd | nd | nd | nd | nd | nd | nd | nd | nd | nd |
| Cho HY et al., 2013 | nd | nd | nd | nd | nd | nd | nd | nd | nd | nd | nd | nd |
| Cho KH et al., 2012 | Yes | nd | nd | nd | nd | nd | Yes | nd | No | 0 | No | 0 |
| Cho MK et al., 2015 | nd | nd | nd | nd | nd | nd | nd | nd | nd | nd | nd | nd |
| Choi HS et al., 2017 | nd | nd | nd | nd | nd | nd | nd | nd | nd | nd | nd | nd |
| Chu et al., 2015 | nd | nd | nd | nd | nd | nd | nd | nd | nd | nd | nd | nd |
| Chung et al., 2014 | Yes | nd | nd | nd | nd | nd | Yes | nd | No | 0 | No | 0 |
| Dault et al., 2003 | Yes | 9 | Yes | 8 | Yes | 1 | Yes | 1 | No | 0 | No | 0 |
| Dujovic et al., 2017 | nd | nd | nd | nd | nd | nd | nd | nd | nd | nd | nd | nd |
| Duncan et al., 1998 | Yes | nd | nd | nd | nd | nd | Yes | 1 | nd | nd | No | 0 |
| Duncan et al., 2003 | Yes | 84 | Yes | 44 | Yes | 40 | Yes | nd | nd | nd | Yes | nd |
| Erbil et al., 2018 | nd | nd | nd | nd | nd | nd | nd | nd | nd | nd | nd | nd |
| Fernandez-Gonzalo et al., 2016 | Yes | 29 | Yes | 15 | Yes | 14 | nd | nd | No | 0 | No | 0 |
| Ferreira et al., 2017 | nd | nd | nd | nd | nd | nd | nd | nd | nd | nd | nd | nd |
| Fritz et al., 2013 | nd | nd | nd | nd | nd | nd | nd | nd | nd | nd | nd | nd |
| Furnari et al., 2014 | nd | nd | nd | nd | nd | nd | nd | nd | nd | nd | nd | nd |
| Geiger et al., 2001 | nd | nd | nd | nd | nd | nd | nd | nd | nd | nd | nd | nd |
| Ghanjal et al., 2014 | Yes | 36 | Yes | 14 | Yes | 22 | No | 0 | No | 0 | No | 0 |
| Globas et al., 2012 | Yes | 36 | Yes | 13 | Yes | 23 | No | 0 | No | 0 | No | 0 |
| Goliwas et al., 2017 | nd | nd | nd | nd | nd | nd | nd | nd | nd | nd | nd | nd |
| Han et al., 2016 | Yes | 56 | Yes | 22 | Yes | 34 | No | 0 | No | 0 | No | 0 |
| Hart et al., 2004 | nd | nd | nd | nd | nd | nd | nd | nd | nd | nd | nd | nd |
| Heller et al., 2005 | nd | nd | nd | nd | nd | nd | nd | nd | nd | nd | nd | nd |
| Hollands et al., 2015 | nd | nd | nd | nd | nd | nd | nd | nd | nd | nd | nd | nd |
| Holmgren et al., 2010 | Yes | 28 | nd | nd | nd | nd | nd | nd | nd | nd | No | 0 |
| Hosseini et al., 2012 | nd | nd | nd | nd | nd | nd | nd | nd | nd | nd | nd | nd |
| Howe et al., 2005 | Yes | 26 | nd | nd | nd | nd | nd | nd | nd | nd | Yes | 5 |
| Hsieh, 2019 | nd | nd | nd | nd | nd | nd | nd | nd | nd | nd | nd | nd |
| Hsu et al., 2013 | nd | nd | nd | nd | nd | nd | nd | nd | nd | nd | nd | nd |
| Huh et al., 2015 | nd | nd | nd | nd | nd | nd | nd | nd | nd | nd | nd | nd |
| Hung et al., 2016 | nd | nd | nd | nd | nd | nd | nd | nd | No | 0 | nd | nd |
| Hwang et al., 2015 | nd | nd | nd | nd | nd | nd | nd | nd | nd | nd | nd | nd |
| Immink et al., 2014 | nd | nd | nd | nd | nd | nd | nd | nd | nd | nd | nd | nd |
| In et al., 2016 | nd | nd | nd | nd | nd | nd | nd | nd | nd | nd | nd | nd |
| Janssen et al., 2008 | nd | nd | nd | nd | nd | nd | nd | nd | nd | nd | nd | nd |
| Jung et al., 2015 | nd | nd | nd | nd | nd | nd | nd | nd | nd | nd | nd | nd |
| Jung et al., 2017 | nd | nd | nd | nd | nd | nd | nd | nd | nd | nd | nd | nd |
| Kamps et Schule, 2005 | nd | nd | nd | nd | nd | nd | nd | nd | nd | nd | nd | nd |
| Karasu et al., 2018 | nd | nd | nd | nd | nd | nd | nd | nd | No | 0 | nd | nd |
| Katz-Leurer et al., 2006 | Yes | nd | nd | nd | nd | nd | No | 0 | nd | nd | No | 0 |
| Khumsapsiri et al., 2018 | Yes | 16 | nd | nd | nd | nd | No | 0 | No | 0 | No | 0 |
| Kilinc et al., 2015 | nd | nd | nd | nd | nd | nd | nd | nd | nd | nd | nd | nd |
| Kim DH et al., 2008 | nd | nd | nd | nd | nd | nd | nd | nd | nd | nd | nd | nd |
| Kim JC et Lee, 2018 | nd | nd | nd | nd | nd | nd | nd | nd | nd | nd | nd | nd |
| Kim JH et al., 2009 | Yes | 23 | Yes | 13 | Yes | 10 | Yes | 1 | No | 0 | No | 0 |
| Kim JY et al., 2018 | Yes | 42 | nd | nd | nd | nd | Yes | 5 | No | 0 | Yes | 2 |
| Kim SL et Lee, 2018 | Yes | 29 | Yes | nd | Yes | nd | Yes | 1 | No | 0 | No | 0 |
| Kim YH et al., 2004 | nd | nd | nd | nd | nd | nd | nd | nd | nd | nd | nd | nd |
| Kim YM et al., 2009 | nd | nd | nd | nd | nd | nd | nd | nd | nd | nd | nd | nd |
| Knox et al., 2018 | nd | nd | nd | nd | nd | nd | nd | nd | nd | nd | nd | nd |
| Kunkel et al., 2013 | Yes | 14 | nd | nd | nd | nd | nd | nd | nd | nd | No | 0 |
| Kwong et al., 2018 | nd | nd | nd | nd | nd | nd | nd | nd | nd | nd | nd | nd |
| Langhammer et al., 2009 | Yes | 67 | Yes | nd | Yes | nd | No | 0 | No | 0 | No | 0 |
| Lau RWK et al., 2012 | Yes | nd | nd | nd | nd | nd | nd | nd | nd | nd | No | 0 |
| Laufer, 2003 | Yes | 30 | Yes | 12 | Yes | 18 | No | 0 | No | 0 | No | 0 |
| Lee CH et al., 2014 | nd | nd | nd | nd | nd | nd | nd | nd | nd | nd | nd | nd |
| Lee D et al., 2016 | nd | nd | nd | nd | nd | nd | nd | nd | nd | nd | nd | nd |
| Lee HJ et al., 2018 | nd | nd | nd | nd | nd | nd | nd | nd | nd | nd | nd | nd |
| Lee MM et al., 2018 | nd | nd | nd | nd | nd | nd | nd | nd | No | 0 | No | 0 |
| Lee NK et al., 2013 | nd | nd | nd | nd | nd | nd | nd | nd | nd | nd | nd | nd |
| Lee SH et al., 2012 | Yes | 40 | Yes | 24 | Yes | 16 | No | 0 | No | 0 | No | 0 |
| Lee SW et al., 2013 | Yes | nd | nd | nd | nd | nd | Yes | nd | No | 0 | No | 0 |
| Liang et al., 2012 | nd | nd | nd | nd | nd | nd | nd | nd | nd | nd | nd | nd |
| Lin Q et al., 2015 | nd | nd | nd | nd | nd | nd | nd | nd | nd | nd | nd | nd |
| Lindvall et Forsberg, 2014 | nd | nd | nd | nd | nd | nd | nd | nd | nd | nd | nd | nd |
| Lisinski et al., 2012 | nd | nd | nd | nd | nd | nd | nd | nd | nd | nd | nd | nd |
| Liu-Ambrose et Eng, 2015 | nd | nd | nd | nd | nd | nd | nd | nd | nd | nd | nd | nd |
| Lu et al., 1997 | nd | nd | nd | nd | nd | nd | nd | nd | nd | nd | nd | nd |
| Lynch et al., 2007 | nd | nd | nd | nd | nd | nd | nd | nd | nd | nd | nd | nd |
| Marin et al., 2013 | nd | nd | nd | nd | nd | nd | nd | nd | nd | nd | nd | nd |
| Merkert et al., 2011 | nd | nd | nd | nd | nd | nd | nd | nd | nd | nd | nd | nd |
| Milczarek et al., 1993 | Yes | 11 | Yes | 4 | Yes | 9 | Yes | 1 | No | 0 | No | 0 |
| Mojica et al., 1988 | nd | nd | nd | nd | nd | nd | nd | nd | nd | nd | nd | nd |
| Moore JL et al., 2010 | Yes | 20 | Yes | 16 | Yes | 4 | No | 0 | No | 0 | No | 0 |
| Morioka et Yagi, 2003 | nd | nd | nd | nd | nd | nd | nd | nd | nd | nd | nd | nd |
| Mudie et al., 2002 | Yes | 22 | nd | nd | nd | nd | nd | nd | Yes | 3 | nd | nd |
| Nadeau et al., 2013 | Yes | 300 | Yes | nd | Yes | nd | Yes | 59 | Yes | 1 | Yes | 48 |
| Ng et al., 2016 | nd | nd | nd | nd | nd | nd | nd | nd | nd | nd | nd | nd |
| Nikamp et al., 2017 | nd | nd | nd | nd | nd | nd | nd | nd | nd | nd | nd | nd |
| Noh et al., 2008 | nd | nd | nd | nd | nd | nd | nd | nd | nd | nd | nd | nd |
| Ordahan et al., 2015 | Yes | 50 | Yes | 29 | Yes | 21 | No | 0 | No | 0 | No | 0 |
| Page et al., 2008 | Yes | nd | Yes | 3 | nd | nd | nd | nd | nd | nd | nd | nd |
| Park D et al., 2018 | Yes | 20 | Yes | 7 | Yes | 13 | No | 0 | No | 0 | No | 0 |
| Park DS et al., 2017 | nd | nd | nd | nd | nd | nd | nd | nd | nd | nd | nd | nd |
| Park et al., 2014 | Yes | nd | nd | nd | nd | nd | Yes | nd | No | 0 | No | 0 |
| Park HK et al., 2018 | nd | nd | nd | nd | nd | nd | nd | nd | nd | nd | nd | nd |
| Park J et al., 2017 | nd | nd | nd | nd | nd | nd | nd | nd | nd | nd | nd | nd |
| Pollock et al., 2002 | Yes | 23 | nd | nd | nd | nd | nd | nd | nd | nd | No | 0 |
| Pomeroy et al., 2001 | nd | nd | nd | nd | nd | nd | nd | nd | nd | nd | nd | nd |
| Rajaratnam et al., 2013 | nd | nd | nd | nd | nd | nd | nd | nd | nd | nd | nd | nd |
| Robertson et al., 2010 | nd | nd | nd | nd | nd | nd | nd | nd | nd | nd | nd | nd |
| Rougier et Boudrahem, 2010 | Yes | 37 | Yes | 20 | Yes | 17 | Yes | 2 | No | 0 | No | 0 |
| Salgueiro et Marquez, 2018 | nd | nd | nd | nd | nd | nd | nd | nd | No | 0 | No | 0 |
| Sanchez-Mila et al., 2018 | nd | nd | nd | nd | nd | nd | nd | nd | nd | nd | nd | nd |
| Schmid et al., 2012 | nd | nd | nd | nd | nd | nd | nd | nd | nd | nd | nd | nd |
| Schuster et al., 2012 | Yes | 27 | Yes | 13 | Yes | 14 | Yes | 7 | Yes | 2 | Yes | 3 |
| Shatil et al., 2005 | nd | nd | nd | nd | nd | nd | nd | nd | nd | nd | nd | nd |
| Shin et al., 2016 | Yes | 24 | Yes | 9 | Yes | 15 | No | 0 | No | 0 | No | 0 |
| Simons et al., 2009 | Yes | 20 | Yes | 10 | Yes | 10 | No | 0 | No | 0 | No | 0 |
| Sohn et al., 2015 | nd | nd | nd | nd | nd | nd | nd | nd | nd | nd | nd | nd |
| Song et al., 2014 | nd | nd | nd | nd | nd | nd | nd | nd | nd | nd | nd | nd |
| Stein et al., 2014 | nd | nd | nd | nd | nd | nd | nd | nd | nd | nd | nd | nd |
| Suh et al., 2014 | nd | nd | nd | nd | nd | nd | nd | nd | nd | nd | nd | nd |
| Tan et al., 2014 | Yes | 45 | nd | nd | nd | nd | No | 0 | No | 0 | No | 0 |
| Tan et al., 2016 | Yes | nd | nd | nd | nd | nd | nd | nd | nd | nd | No | 0 |
| Tian et al., 2014 | Yes | nd | nd | nd | nd | nd | nd | nd | nd | nd | nd | nd |
| Tilikete et al., 2001 | Yes | 15 | Yes | 15 | No | 0 | No | 0 | No | 0 | No | 0 |
| Tripp and Krakow, 2014 | Yes | 20 | Yes | 11 | Yes | 9 | Yes | 4 | Yes | 1 | Yes | 5 |
| Tung et al., 2010 | nd | nd | nd | nd | nd | nd | nd | nd | nd | nd | nd | nd |
| Vahlberg et al., 2017 | nd | nd | nd | nd | nd | nd | nd | nd | nd | nd | nd | nd |
| VanNes et al., 2006 | Yes | 53 | Yes | 28 | Yes | 25 | No | 0 | No | 0 | No | 0 |
| Waldron et Bohannon, 1989 | nd | nd | nd | nd | nd | nd | nd | nd | nd | nd | nd | nd |
| Wang et al., 2017 | Yes | 36 | Yes | 19 | Yes | 17 | No | 0 | No | 0 | No | 0 |
| Wang RY, Lin PY et al., 2007 | nd | nd | nd | nd | nd | nd | nd | nd | nd | nd | nd | nd |
| Wang RY, Yen LL et al., 2005 part 1 | nd | nd | nd | nd | nd | nd | nd | nd | nd | nd | nd | nd |
| Wang RY, Yen LL et al., 2005 part 2 | nd | nd | nd | nd | nd | nd | nd | nd | nd | nd | nd | nd |
| Wang TC et al., 2015 | Yes | 51 | Yes | 27 | Yes | 24 | No | 0 | No | 0 | No | 0 |
| Xie et al., 2018 | nd | nd | nd | nd | nd | nd | nd | nd | nd | nd | nd | nd |
| Xing et al., 2007 | Yes | 64 | nd | nd | nd | nd | Yes | 5 | Yes | 3 | No | 0 |
| Yadav et al., 2015 | nd | nd | nd | nd | nd | nd | nd | nd | nd | nd | nd | nd |
| Yeung et al., 2018 | nd | nd | nd | nd | nd | nd | nd | nd | nd | nd | nd | nd |
| Yoo et al., 2010 | nd | nd | nd | nd | nd | nd | nd | nd | nd | nd | nd | nd |
| Yoo et al., 2018 | nd | nd | nd | nd | nd | nd | nd | nd | nd | nd | nd | nd |
| You et al., 2014 | nd | nd | nd | nd | nd | nd | nd | nd | nd | nd | nd | nd |
| Yu et Cho, 2016 | nd | nd | nd | nd | nd | nd | nd | nd | No | 0 | No | 0 |
| Yun et al., 2018 | Yes | nd | nd | nd | nd | nd | nd | nd | nd | nd | nd | nd |
| Zhang et al., 2015 | nd | nd | nd | nd | nd | nd | nd | nd | nd | nd | nd | nd |

Abbreviations: nd, not documented.

**S3D Table. Characteristics of participants for each study included**

| Study | Episode of stroke | First stroke (Participant) | Multiple stroke (Participant) | Unilateral / bilateral stroke | Unilateral stroke (Participant) | Bilateral stroke (Participant) | Ischemic stroke | | Hemorrhagic stroke | | Other etiology of stroke | |
| --- | --- | --- | --- | --- | --- | --- | --- | --- | --- | --- | --- | --- |
|  |  |  |  |  |  |  | Trial | Participant | Trial | Participant | Trial | Participant |
| Allison et Dennett, 2007 | nd | nd | nd | nd | nd | nd | nd | nd | nd | nd | nd | nd |
| Arabzadeh et al., 2018 | nd | nd | nd | U | 20 | 0 | 1 | 20 | 0 | 0 | 0 | 0 |
| Askim et al., 2010 | F & M | 55 | 7 | nd | nd | nd | nd | nd | Yes | 3 | nd | nd |
| Au-Yeung et al., 2009 | nd | nd | nd | U | 136 | 0 | nd | nd | nd | nd | nd | nd |
| Bae et al., 2015 | nd | nd | nd | nd | nd | nd | nd | nd | nd | nd | nd | nd |
| Barcala et al., 2011 | nd | nd | nd | nd | nd | nd | nd | nd | nd | nd | nd | nd |
| Brogardh et al., 2012 | nd | nd | nd | U | 31 | 0 | Yes | 27 | Yes | 4 | No | 0 |
| Bunketorp-Kall et al., 2017 | F & M | nd | nd | U | 123 | 0 | 1 | 87 | 1 | 36 | 0 | 0 |
| Buyukavci et al., 2016 | F | 65 | 0 | nd | nd | nd | 1 | 52 | 1 | 12 | 0 | 0 |
| Büyükvural Şen et al., 2015 | nd | nd | nd | U | 50 | 0 | Yes | 44 | Yes | 6 | No | 0 |
| Cabanas-Valdés et al., 2015 | F | 80 | 0 | U | 80 | 0 | Yes | 65 | Yes | 15 | No | 0 |
| Chan KS et al., 2012 | F | 30 | 0 | U | 30 | 0 | Yes | 15 | Yes | 15 | No | 0 |
| Chen CH et al., 2010 | F | 10 | 0 | U | 10 | 0 | Yes | 3 | Yes | 7 | No | 0 |
| Chen CL et al., 2015 | nd | nd | nd | U | 24 | 0 | Yes | 16 | Yes | 7 | Yes | 1 |
| Chen D et al., 2014 | nd | nd | nd | U | 48 | 0 | Yes | 37 | Yes | 11 | No | 0 |
| Chen IC et al., 2002 | F | 41 | 0 | U | 41 | 0 | nd | nd | nd | nd | nd | nd |
| Chen JC et al., 2011 | F | 33 | 0 | U | 33 | 0 | Yes | 11 | Yes | 22 | No | 0 |
| Chen, 2018 | F & M | nd | nd | nd | nd | nd | nd | nd | nd | nd | nd | nd |
| Chern et al., 2013 | nd | nd | nd | U | 15 | 0 | nd | nd | nd | nd | nd | nd |
| Cho HY et al., 2013 | F | 42 | 0 | nd | nd | nd | Yes | 29 | Yes | 13 | No | 0 |
| Cho KH et al., 2012 | F | 22 | 0 | U | 22 | 0 | Yes | 13 | Yes | 9 | No | 0 |
| Cho MK et al., 2015 | F | 31 | 0 | U | 31 | 0 | Yes | 14 | Yes | 17 | No | 0 |
| Choi HS et al., 2017 | nd | nd | nd | U | 36 | 0 | nd | nd | nd | nd | nd | nd |
| Chu et al., 2015 | F | 145 | 0 | U | 145 | 0 | 1 | 113 | 1 | 32 | 0 | 0 |
| Chung et al., 2014 | F | 18 | 0 | U | 18 | 0 | Yes | 9 | Yes | 9 | No | 0 |
| Dault et al., 2003 | F | 10 | 0 | U | 10 | 0 | Yes | 10 | No | 0 | No | 0 |
| Dujovic et al., 2017 | F | 16 | 0 | U | 16 | 0 | 1 | 13 | 1 | 3 | 0 | 0 |
| Duncan et al., 1998 | nd | nd | nd | U | 19 | nd | Yes | 18 | Yes | 2 | No | 0 |
| Duncan et al., 2003 | nd | nd | nd | U | 84 | nd | Yes | 83 | nd | nd | nd | nd |
| Erbil et al., 2018 | nd | nd | nd | U | 43 | 0 | 1 | 28 | 1 | 15 | 0 | 0 |
| Fernandez-Gonzalo et al., 2016 | F & M | 28 | 1 | U | 29 | 0 | 1 | 20 | 1 | 9 | 0 | 0 |
| Ferreira et al., 2017 | nd | nd | nd | U | 20 | 0 | 1 | 16 | 1 | 4 | 0 | 0 |
| Fritz et al., 2013 | nd | nd | nd | U | 28 | 0 | nd | nd | nd | nd | nd | nd |
| Furnari et al., 2014 | F | 40 | 0 | U | 40 | 0 | nd | nd | nd | nd | nd | nd |
| Geiger et al., 2001 | nd | nd | nd | U | 13 | 0 | nd | nd | nd | nd | nd | nd |
| Ghanjal et al., 2014 | F | 36 | 0 | U | 36 | 0 | Yes | 36 | No | 0 | No | 0 |
| Globas et al., 2012 | nd | nd | nd | U | 36 | 0 | nd | nd | nd | nd | nd | nd |
| Goliwas et al., 2017 | nd | nd | nd | U | 37 | 0 | 1 | 37 | 0 | 0 | 0 | 0 |
| Han et al., 2016 | F | 56 | 0 | U | 56 | 0 | 1 | 33 | 1 | 23 | 0 | 0 |
| Hart et al., 2004 | F | 18 | 0 | U | 18 | 0 | nd | nd | nd | nd | nd | nd |
| Heller et al., 2005 | F | 26 | 0 | U | 26 | 0 | Yes | 15 | Yes | 11 | No | 0 |
| Hollands et al., 2015 | nd | nd | nd | U & B | 51 | 5 | 1 | 49 | 1 | 7 | 0 | 0 |
| Holmgren et al., 2010 | F & M | 19 | 15 | nd | nd | nd | 1 | 23 | 1 | 1 | 1 | 10 |
| Hosseini et al., 2012 | M | 0 | 30 | nd | nd | nd | nd | nd | nd | nd | nd | nd |
| Howe et al., 2005 | F & M | 28 | 7 | U | 35 | 0 | Yes | nd | Yes | nd | No | nd |
| Hsieh, 2019 | nd | nd | nd | U | 56 | 0 | 1 | 56 | 0 | 0 | 0 | 0 |
| Hsu et al., 2013 | F | 23 | 0 | U | 23 | 0 | Yes | 11 | Yes | 12 | No | 0 |
| Huh et al., 2015 | nd | nd | nd | U | 40 | 0 | 1 | 22 | 1 | 18 | 0 | 0 |
| Hung et al., 2016 | nd | nd | nd | U | 23 | 0 | 1 | 17 | 1 | 6 | 0 | 0 |
| Hwang et al., 2015 | F & M | nd | nd | U | 30 | 0 | Yes | 14 | Yes | 16 | No | 0 |
| Immink et al., 2014 | nd | nd | nd | U | 22 | 0 | nd | nd | nd | nd | nd | nd |
| In et al., 2016 | nd | nd | nd | U | 25 | 0 | 1 | 16 | 1 | 9 | 0 | 0 |
| Janssen et al., 2008 | nd | nd | nd | U | 12 | 0 | Yes | 11 | Yes | 1 | No | 0 |
| Jung et al., 2015 | nd | nd | nd | U | 22 | 0 | nd | nd | nd | nd | nd | nd |
| Jung et al., 2017 | F | 40 | 0 | U | 40 | 0 | 1 | 23 | 1 | 17 | 0 | 0 |
| Kamps et Schule, 2005 | nd | nd | nd | U | 31 | 0 | Yes | 31 | No | 0 | No | 0 |
| Karasu et al., 2018 | F | 23 | 0 | U | 23 | 0 | 1 | 18 | 1 | 5 | 0 | 0 |
| Katz-Leurer et al., 2006 | F | 24 | 0 | U | 24 | 0 | Yes | 24 | No | 0 | No | 0 |
| Khumsapsiri et al., 2018 | F | 16 | 0 | U | 16 | 0 | 1 | 11 | 1 | 5 | 0 | 0 |
| Kilinc et al., 2015 | F | 22 | 0 | U | 22 | 0 | 1 | 11 | 1 | 11 | 0 | 0 |
| Kim DH et al., 2008 | nd | nd | nd | U | 16 | 0 | nd | nd | nd | nd | nd | nd |
| Kim JC et Lee, 2018 | nd | nd | nd | U | 21 | 0 | 1 | 14 | 1 | 7 | 0 | 0 |
| Kim JH et al., 2009 | F | 24 | 0 | U | 24 | 0 | Yes | 11 | Yes | 13 | No | 0 |
| Kim JY et al., 2018 | F | 48 | 0 | U | 48 | 0 | 1 | 32 | 1 | 16 | 0 | 0 |
| Kim SL et Lee, 2018 | F | 30 | 0 | U | 30 | 0 | 1 | 12 | 1 | 18 | 0 | 0 |
| Kim YH et al., 2004 | nd | nd | nd | U | 38 | 0 | Yes | 28 | Yes | 10 | No | 0 |
| Kim YM et al., 2009 | nd | nd | nd | U | 32 | 0 | Yes | 23 | Yes | 9 | No | 0 |
| Knox et al., 2018 | F | 144 | 0 | U | 144 | 0 | 1 | nd | 1 | nd | 0 | 0 |
| Kunkel et al., 2013 | F | 21 | 0 | U & B | 18 | 3 | Yes | 20 | Yes | 1 | No | 0 |
| Kwong et al., 2018 | nd | nd | nd | U | 80 | 0 | 1 | 49 | 1 | 30 | 1 | 1 |
| Langhammer et al., 2009 | F | 67 | 0 | U | 67 | 0 | Yes | nd | Yes | nd | No | 0 |
| Lau RWK et al., 2012 | nd | nd | nd | U | 82 | 0 | Yes | 41 | nd | nd | nd | nd |
| Laufer, 2003 | F | 30 | 0 | U | 30 | 0 | Yes | 29 | Yes | 1 | No | 0 |
| Lee CH et al., 2014 | nd | nd | nd | nd | nd | nd | nd | nd | nd | nd | nd | nd |
| Lee D et al., 2016 | nd | nd | nd | U | 27 | 0 | 1 | 8 | 1 | 20 | 0 | 0 |
| Lee HJ et al., 2018 | nd | nd | nd | U | 20 | 0 | 1 | 18 | 1 | 2 | 0 | 0 |
| Lee MM et al., 2018 | nd | nd | nd | U | 30 | 0 | 1 | 19 | 1 | 11 | 0 | 0 |
| Lee NK et al., 2013 | F | 33 | 0 | U | 33 | 0 | Yes | 20 | Yes | 13 | No | 0 |
| Lee SH et al., 2012 | F | 40 | 0 | U | 40 | 0 | Yes | 27 | Yes | 13 | No | 0 |
| Lee SW et al., 2013 | F | 31 | 0 | U | 31 | 0 | Yes | 19 | Yes | 12 | No | 0 |
| Liang et al., 2012 | F | 30 | 0 | U | 30 | 0 | Yes | 18 | Yes | 12 | No | 0 |
| Lin Q et al., 2015 | nd | nd | nd | nd | nd | nd | 1 | 42 | 1 | 22 | 0 | 0 |
| Lindvall et Forsberg, 2014 | F & M | 38 | 8 | U | 46 | 0 | Yes | 34 | Yes | 12 | No | 0 |
| Lisinski et al., 2012 | nd | nd | nd | U | 26 | 0 | Yes | 26 | No | 0 | No | 0 |
| Liu-Ambrose et Eng, 2015 | F | 25 | 0 | nd | nd | nd | Yes | 14 | Yes | 10 | No | 0 |
| Lu et al., 1997 | nd | nd | nd | nd | nd | nd | nd | nd | nd | nd | nd | nd |
| Lynch et al., 2007 | F | 21 | 0 | U | 21 | 0 | Yes | 18 | Yes | 3 | No | 0 |
| Marin et al., 2013 | nd | nd | nd | U | 39 | 0 | Yes | 17 | Yes | 3 | No | 0 |
| Merkert et al., 2011 | F & M | 46 | 20 | U & B | 54 | 12 | Yes | 53 | Yes | 7 | Yes | 6 |
| Milczarek et al., 1993 | nd | nd | nd | U | 14 | 0 | Yes | 11 | Yes | 3 | No | 0 |
| Mojica et al., 1988 | nd | nd | nd | U | 8 | 0 | nd | nd | nd | nd | nd | nd |
| Moore JL et al., 2010 | nd | nd | nd | U | 20 | 0 | Yes | 10 | Yes | 10 | No | 0 |
| Morioka et Yagi, 2003 | nd | nd | nd | U | 26 | 0 | nd | nd | nd | nd | nd | nd |
| Mudie et al., 2002 | nd | nd | nd | U | 40 | 0 | Yes | 29 | Yes | 11 | No | 0 |
| Nadeau et al., 2013 | F | 408 | 0 | U | 408 | 0 | Yes | 290 | Yes | 70 | Yes | 48 |
| Ng et al., 2016 | F | 76 | 0 | U | 76 | 0 | 1 | 65 | 1 | 11 | 0 | 0 |
| Nikamp et al., 2017 | F | 33 | 0 | U | 33 | 0 | 1 | 27 | 1 | 6 | 0 | 0 |
| Noh et al., 2008 | F | 25 | 0 | U | 25 | 0 | 1 | 13 | 1 | 12 | 0 | 0 |
| Ordahan et al., 2015 | F | 50 | 0 | U | 50 | 0 | 1 | 41 | 1 | 9 | 0 | 0 |
| Page et al., 2008 | nd | nd | nd | nd | nd | nd | 1 | 7 | 0 | 0 | 0 | 0 |
| Park D et al., 2018 | F | 20 | 0 | U | 20 | 0 | 1 | 11 | 1 | 9 | 0 | 0 |
| Park DS et al., 2017 | nd | nd | nd | U | 20 | 0 | 1 | 13 | 1 | 7 | 0 | 0 |
| Park et al., 2014 | nd | nd | nd | U & B | 29 | 1 | nd | nd | nd | nd | nd | nd |
| Park HK et al., 2018 | F | 29 | 0 | U | 29 | 0 | 1 | 15 | 1 | 14 | 0 | 0 |
| Park J et al., 2017 | nd | nd | nd | nd | nd | nd | nd | nd | nd | nd | nd | nd |
| Pollock et al., 2002 | nd | nd | nd | U | 28 | 0 | Yes | 25 | Yes | 3 | No | 0 |
| Pomeroy et al., 2001 | F & M | nd | nd | U | 24 | 0 | nd | nd | nd | nd | nd | nd |
| Rajaratnam et al., 2013 | F | 19 | 0 | U | 19 | 0 | Yes | 16 | Yes | 3 | No | 0 |
| Robertson et al., 2010 | F | 15 | 0 | U | 15 | 0 | Yes | 8 | Yes | 7 | No | 0 |
| Rougier et Boudrahem, 2010 | F | 39 | 0 | U | 39 | 0 | Yes | 28 | Yes | 11 | No | 0 |
| Salgueiro et Marquez, 2018 | nd | nd | nd | U | 12 | 0 | 1 | 7 | 1 | 5 | 0 | 0 |
| Sanchez-Mila et al., 2018 | F | 26 | 0 | U | 26 | 0 | nd | nd | nd | nd | nd | nd |
| Schmid et al., 2012 | nd | nd | nd | nd | nd | nd | Yes | 31 | nd | nd | Yes | nd |
| Schuster et al., 2012 | F | 39 | 0 | U & B | 38 | 1 | Yes | 30 | Yes | 9 | No | 0 |
| Shatil et al., 2005 | nd | nd | nd | U | 18 | 0 | Yes | 14 | Yes | 4 | No | 0 |
| Shin et al., 2016 | F | 24 | 0 | U | 24 | 0 | 1 | 5 | 1 | 19 | 0 | 0 |
| Simons et al., 2009 | F | 20 | 0 | U | 20 | 0 | Yes | 17 | Yes | 3 | No | 0 |
| Sohn et al., 2015 | nd | nd | nd | U | 27 | 0 | 1 | 16 | 1 | 11 | 0 | 0 |
| Song et al., 2014 | F | 30 | 0 | U | 30 | 0 | nd | nd | nd | nd | nd | nd |
| Stein et al., 2014 | F | 24 | 0 | nd | nd | nd | 1 | nd | 1 | nd | 0 | 0 |
| Suh et al., 2014 | nd | nd | nd | nd | nd | nd | Yes | 29 | Yes | 11 | Yes | 2 |
| Tan et al., 2014 | F | 45 | 0 | U | 45 | 0 | Yes | 45 | No | 0 | No | 0 |
| Tan et al., 2016 | F | 58 | 0 | U | 58 | 0 | 1 | 30 | 1 | 28 | 0 | 0 |
| Tian et al., 2014 | F | 100 | 0 | U | 100 | 0 | Yes | 68 | Yes | 32 | No | 0 |
| Tilikete et al., 2001 | F | 15 | 0 | U | 15 | 0 | Yes | 15 | No | 0 | No | 0 |
| Tripp and Krakow, 2014 | F | 30 | 0 | U | 30 | 0 | Yes | 27 | Yes | 3 | No | 0 |
| Tung et al., 2010 | F | 32 | 0 | U | 32 | 0 | nd | nd | nd | nd | nd | nd |
| Vahlberg et al., 2017 | F & M | 53 | 14 | nd | nd | nd | 1 | 55 | 1 | 12 | 0 | 0 |
| VanNes et al., 2006 | F | 53 | 0 | U | 53 | 0 | Yes | 38 | Yes | 15 | No | 0 |
| Waldron et Bohannon, 1989 | nd | nd | nd | nd | nd | nd | nd | nd | nd | nd | nd | nd |
| Wang et al., 2017 | F | 36 | 0 | U | 36 | 0 | nd | nd | nd | nd | nd | nd |
| Wang RY, Lin PY et al., 2007 | nd | nd | nd | U | 58 | 0 | Yes | 39 | Yes | 19 | No | 0 |
| Wang RY, Yen LL et al., 2005 part 1 | nd | nd | nd | U | 42 | 0 | nd | nd | nd | nd | nd | nd |
| Wang RY, Yen LL et al., 2005 part 2 | nd | nd | nd | U | 61 | 0 | nd | nd | nd | nd | nd | nd |
| Wang TC et al., 2015 | F | 51 | 0 | U | 51 | 0 | Yes | nd | Yes | nd | No | 0 |
| Xie et al., 2018 | F | 244 | 0 | U & B | 239 | 5 | 1 | 160.608 | 1 | 63 | 0 | 0 |
| Xing et al., 2007 | F & M | nd | nd | nd | nd | nd | Yes | 72 | No | 0 | No | 0 |
| Yadav et al., 2015 | F | 24 | 0 | U | 24 | 0 | nd | nd | nd | nd | nd | nd |
| Yeung et al., 2018 | nd | nd | nd | U | 19 | 0 | 1 | 14 | 1 | 5 | 0 | 0 |
| Yoo et al., 2010 | nd | nd | nd | U | 59 | 0 | Yes | 29 | Yes | 30 | No | 0 |
| Yoo et al., 2018 | F | 40 | 0 | U | 40 | 0 | 1 | 23 | 1 | 17 | 0 | 0 |
| You et al., 2014 | F | 37 | 0 | nd | nd | nd | Yes | 33 | Yes | 4 | No | 0 |
| Yu et Cho, 2016 | F | 20 | 0 | U | 20 | 0 | 1 | 12 | 1 | 8 | 0 | 0 |
| Yun et al., 2018 | nd | nd | nd | U | 36 | 0 | 1 | 25 | 1 | 11 | 0 | 0 |
| Zhang et al., 2015 | F | 60 | 0 | U | 60 | 0 | nd | nd | nd | nd | nd | nd |

Abbreviations: F, first; M, multiple; nd, not documented; U, unilateral.

**S3E Table. Characteristics of participants for each study included**

| Study | Description of stroke lesion using brain imagery | Time post-stroke | | Eligibility / Inclusion criteria of participants in trials | | |
| --- | --- | --- | --- | --- | --- | --- |
|  |  | Mean (d) | SD (d) |  |  |  |
|  |  |  |  | Acute stroke | Subacute stroke | Chronic stroke |
| Allison et Dennett, 2007 | No imagery used | 17.36 | 17.59 | Yes | No | No |
| Arabzadeh et al., 2018 | No imagery used | 32.65 | 15.82 | Yes | Yes | No |
| Askim et al., 2010 | No imagery used | 14.61 | 6.94 | Yes | No | No |
| Au-Yeung et al., 2009 | No imagery used | 1742.16 | 2635.20 | No | No | Yes |
| Bae et al., 2015 | Use of imagery reported without description of lesion | nd | nd | Yes | Yes | No |
| Barcala et al., 2011 | No imagery used | 427.00 | 201.91 | No | No | No |
| Brogardh et al., 2012 | No imagery used | 1077.24 | 919.20 | No | No | Yes |
| Bunketorp-Kall et al., 2017 | No imagery used | 1056.00 | 484.14 | No | No | Yes |
| Buyukavci et al., 2016 | No imagery used | 35.91 | 16.23 | No | Yes | No |
| Büyükvural Şen et al., 2015 | No imagery used | 91.50 | nd | No | Yes | Yes |
| Cabanas-Valdés et al., 2015 | Use of imagery reported without description of lesion | 23.25 | 16.66 | Yes | Yes | No |
| Chan KS et al., 2012 | Use of imagery reported without description of lesion | 1056.37 | 986.00 | No | No | Yes |
| Chen CH et al., 2010 | Imagery used with description of lesion in text | 66.10 | 41.43 | Yes | Yes | No |
| Chen CL et al., 2015 | No imagery used | 832.65 | 957.70 | No | No | No |
| Chen D et al., 2014 | Use of imagery reported without description of lesion | 20.31 | 11.71 | Yes | Yes | No |
| Chen IC et al., 2002 | Use of imagery reported without description of lesion | 101.94 | nd | No | No | No |
| Chen JC et al., 2011 | Use of imagery reported without description of lesion | 11 | nd | Yes | No | No |
| Chen, 2018 | Use of imagery reported without description of lesion | nd | nd | No | Yes | Yes |
| Chern et al., 2013 | No imagery used | nd | nd | No | No | No |
| Cho HY et al., 2013 | No imagery used | 441.52 | 151.33 | No | No | Yes |
| Cho KH et al., 2012 | No imagery used | 383.84 | 76.21 | No | No | Yes |
| Cho MK et al., 2015 | No imagery used | 676.80 | 340.99 | No | No | Yes |
| Choi HS et al., 2017 | No imagery used | 422.73 | 141.91 | No | No | Yes |
| Chu et al., 2015 | Use of imagery reported without description of lesion | 40.97 | 33.24 | Yes | Yes | No |
| Chung et al., 2014 | No imagery used | 767.08 | 93.55 | No | No | Yes |
| Dault et al., 2003 | No imagery used | 83.02 | 32.97 | No | No | No |
| Dujovic et al., 2017 | Use of imagery reported without description of lesion | nd | nd | Yes | Yes | Yes |
| Duncan et al., 1998 | No imagery used | 61.00 | nd | No | Yes | No |
| Duncan et al., 2003 | No imagery used | 75.41 | 27.80 | No | Yes | No |
| Erbil et al., 2018 | No imagery used | 1059.41 | 969.41 | No | No | Yes |
| Fernandez-Gonzalo et al., 2016 | Use of imagery reported without description of lesion | 1429.51 | 1557.68 | No | No | Yes |
| Ferreira et al., 2017 | No imagery used | 1347.77 | 511.35 | No | No | Yes |
| Fritz et al., 2013 | No imagery used | 1099.66 | 1056.60 | No | No | Yes |
| Furnari et al., 2014 | No imagery used | 192.15 | 42.70 | No | No | No |
| Geiger et al., 2001 | No imagery used | 115.00 | 148.90 | No | No | No |
| Ghanjal et al., 2014 | Use of imagery reported without description of lesion | nd | nd | No | Yes | No |
| Globas et al., 2012 | Imagery used with description of lesion in text | 1985.55 | 1747.65 | No | No | Yes |
| Goliwas et al., 2017 | No imagery used | 1345.50 | 865.15 | No | No | Yes |
| Han et al., 2016 | Use of imagery reported without description of lesion | 19.95 | 8.95 | Yes | Yes | No |
| Hart et al., 2004 | No imagery used | 823.50 | nd | No | No | No |
| Heller et al., 2005 | No imagery used | 24.70 | 9.73 | Yes | No | No |
| Hollands et al., 2015 | No imagery used | 247.36 | 380.34 | Yes | Yes | Yes |
| Holmgren et al., 2010 | No imagery used | 132.49 | 32.65 | No | Yes | Yes |
| Hosseini et al., 2012 | No imagery used | 588.65 | 230.28 | No | Yes | Yes |
| Howe et al., 2005 | Imagery used with description of lesion in text | 24.75 | 16.50 | Yes | Yes | No |
| Hsieh, 2019 | No imagery used | 382.78 | 204.74 | No | Yes | Yes |
| Hsu et al., 2013 | No imagery used | 234.19 | 184.53 | No | Yes | Yes |
| Huh et al., 2015 | No imagery used | 125.44 | 38.41 | No | Yes | Yes |
| Hung et al., 2016 | No imagery used | 549.80 | 381.52 | No | No | Yes |
| Hwang et al., 2015 | No imagery used | 193.30 | 18.56 | No | No | Yes |
| Immink et al., 2014 | No imagery used | 1601.25 | 1887.95 | No | No | Yes |
| In et al., 2016 | No imagery used | 397.70 | 141.85 | No | No | Yes |
| Janssen et al., 2008 | No imagery used | 466.65 | 250.81 | No | No | Yes |
| Jung et al., 2015 | No imagery used | 492.12 | 125.37 | No | No | Yes |
| Jung et al., 2017 | Use of imagery reported without description of lesion | 200.69 | 78.37 | No | No | No |
| Kamps et Schule, 2005 | No imagery used | 416.18 | 329.11 | No | No | No |
| Karasu et al., 2018 | Use of imagery reported without description of lesion | 29.96 | nd | Yes | Yes | Yes |
| Katz-Leurer et al., 2006 | No imagery used | <30 | nd | Yes | No | No |
| Khumsapsiri et al., 2018 | Use of imagery reported without description of lesion | 710.41 | 712.74 | No | Yes | Yes |
| Kilinc et al., 2015 | Use of imagery reported without description of lesion | 1907.53 | 1507.08 | Yes | Yes | No |
| Kim DH et al., 2008 | No imagery used | 1229.61 | 810.62 | No | No | Yes |
| Kim JC et Lee, 2018 | No imagery used | 1157.66 | 957.66 | No | No | Yes |
| Kim JH et al., 2009 | Imagery used with description of lesion in text | 764.94 | 282.50 | No | No | Yes |
| Kim JY et al., 2018 | Use of imagery reported without description of lesion | 69.77 | 83.70 | Yes | Yes | Yes |
| Kim SL et Lee, 2018 | Imagery used with description of lesion in text | 287.77 | 85.44 | No | No | Yes |
| Kim YH et al., 2004 | No imagery used | 552.66 | 907.60 | No | Yes | Yes |
| Kim YM et al., 2009 | No imagery used | 12.80 | 7.41 | Yes | Yes | No |
| Knox et al., 2018 | No imagery used | 66.50 | 51.59 | Yes | Yes | No |
| Kunkel et al., 2013 | Imagery used with description of lesion in text | 32.20 | nd | Yes | No | No |
| Kwong et al., 2018 | Use of imagery reported without description of lesion | 1899.30 | 1132.28 | No | No | Yes |
| Langhammer et al., 2009 | Use of imagery reported without description of lesion | 18.80 | 11.81 | Yes | No | No |
| Lau RWK et al., 2012 | No imagery used | 1807.99 | 1409.15 | No | No | Yes |
| Laufer, 2003 | Use of imagery reported without description of lesion | 77.70 | 61.70 | No | No | No |
| Lee CH et al., 2014 | No imagery used | 345.67 | 137.37 | No | No | Yes |
| Lee D et al., 2016 | Use of imagery reported without description of lesion | 1206.53 | 904.16 | No | No | Yes |
| Lee HJ et al., 2018 | No imagery used | 535.28 | 118.17 | No | No | Yes |
| Lee MM et al., 2018 | No imagery used | 100.04 | 43.51 | No | Yes | No |
| Lee NK et al., 2013 | No imagery used | 609.09 | 248.58 | No | No | Yes |
| Lee SH et al., 2012 | Use of imagery reported without description of lesion | 416.33 | 184.54 | No | No | Yes |
| Lee SW et al., 2013 | No imagery used | 1633.22 | 842.41 | No | No | Yes |
| Liang et al., 2012 | No imagery used | 12.25 | 5.98 | Yes | No | No |
| Lin Q et al., 2015 | Use of imagery reported without description of lesion | nd | nd | Yes | Yes | No |
| Lindvall et Forsberg, 2014 | No imagery used | 1515.79 | 1497.53 | No | No | Yes |
| Lisinski et al., 2012 | Use of imagery reported without description of lesion | 518.50 | 91.50 | No | No | No |
| Liu-Ambrose et Eng, 2015 | No imagery used | 986.18 | 401.78 | No | No | Yes |
| Lu et al., 1997 | No imagery used | 1500.60 | 1256.60 | No | No | No |
| Lynch et al., 2007 | No imagery used | 48.23 | 28.62 | No | No | No |
| Marin et al., 2013 | No imagery used | 1570.58 | 886.79 | No | No | Yes |
| Merkert et al., 2011 | Use of imagery reported without description of lesion | 54.15 | 203.65 | No | No | No |
| Milczarek et al., 1993 | Use of imagery reported without description of lesion | 161.71 | 170.71 | No | No | No |
| Mojica et al., 1988 | No imagery used | 144.90 | nd | No | No | No |
| Moore JL et al., 2010 | No imagery used | 396.5 | 244 | No | No | Yes |
| Morioka et Yagi, 2003 | No imagery used | 63.52 | 19.50 | No | No | No |
| Mudie et al., 2002 | No imagery used | nd | nd | Yes | Yes | No |
| Nadeau et al., 2013 | Use of imagery reported without description of lesion | 63.80 | 8.50 | No | Yes | No |
| Ng et al., 2016 | No imagery used | 43.42 | 19.51 | Yes | Yes | No |
| Nikamp et al., 2017 | No imagery used | 31.40 | 6.30 | Yes | Yes | No |
| Noh et al., 2008 | No imagery used | 812.32 | 1090.81 | No | No | Yes |
| Ordahan et al., 2015 | Use of imagery reported without description of lesion | 87.30 | 26.30 | No | Yes | No |
| Page et al., 2008 | No imagery used | 1355.12 | 746.64 | No | No | Yes |
| Park D et al., 2018 | Use of imagery reported without description of lesion | 338.55 | 108.81 | No | No | Yes |
| Park DS et al., 2017 | No imagery used | 379.42 | 225.81 | No | No | Yes |
| Park et al., 2014 | No imagery used | 567.91 | 64.36 | No | No | Yes |
| Park HK et al., 2018 | Use of imagery reported without description of lesion | 339.59 | 175.89 | No | No | Yes |
| Park J et al., 2017 | No imagery used | nd | nd | Yes | Yes | No |
| Pollock et al., 2002 | Imagery used with description of lesion in text | nd | nd | Yes | Yes | No |
| Pomeroy et al., 2001 | No imagery used | nd | nd | No | No | Yes |
| Rajaratnam et al., 2013 | No imagery used | 14.85 | 6.80 | Yes | No | No |
| Robertson et al., 2010 | Use of imagery reported without description of lesion | 1716.68 | 1972.35 | No | No | Yes |
| Rougier et Boudrahem, 2010 | Use of imagery reported without description of lesion | 92.79 | 55.63 | No | No | No |
| Salgueiro et Marquez, 2018 | No imagery used | 1568.75 | 1091.34 | No | No | Yes |
| Sanchez-Mila et al., 2018 | No imagery used | nd | nd | No | No | No |
| Schmid et al., 2012 | No imagery used | 1555.50 | 1232.20 | No | No | Yes |
| Schuster et al., 2012 | Imagery used with description of lesion in text | 1295.25 | 1178.92 | No | Yes | Yes |
| Shatil et al., 2005 | No imagery used | 1528.05 | 1567.70 | No | No | Yes |
| Shin et al., 2016 | Imagery used with description of lesion in text | 499.44 | 262.44 | No | No | Yes |
| Simons et al., 2009 | No imagery used | 1198.65 | 1138.26 | No | Yes | Yes |
| Sohn et al., 2015 | Use of imagery reported without description of lesion | 64.40 | 57.40 | No | No | No |
| Song et al., 2014 | No imagery used | 12.90 | 3.23 | Yes | No | No |
| Stein et al., 2014 | Use of imagery reported without description of lesion | 68.80 | 111.01 | No | No | Yes |
| Suh et al., 2014 | No imagery used | 440.73 | 150.90 | No | No | Yes |
| Tan et al., 2014 | Use of imagery reported without description of lesion | 41.46 | 23.90 | Yes | Yes | No |
| Tan et al., 2016 | Use of imagery reported without description of lesion | 213.50 | 114.99 | No | Yes | Yes |
| Tian et al., 2014 | No imagery used | 34.60 | 16.47 | Yes | Yes | No |
| Tilikete et al., 2001 | Imagery used with description of lesion in text | 77.27 | nd | Yes | Yes | No |
| Tripp and Krakow, 2014 | Use of imagery reported without description of lesion | 45.03 | 32.99 | Yes | Yes | No |
| Tung et al., 2010 | No imagery used | 605.43 | 480.68 | No | No | No |
| Vahlberg et al., 2017 | No imagery used | 396.50 | 71.23 | No | No | Yes |
| VanNes et al., 2006 | Use of imagery reported without description of lesion | 36.59 | 10.35 | Yes | Yes | No |
| Waldron et Bohannon, 1989 | No imagery used | 130 | 211.7 | No | No | No |
| Wang et al., 2017 | Imagery used with description of lesion in text | nd | nd | Yes | Yes | No |
| Wang RY, Lin PY et al., 2007 | No imagery used | 100.35 | 35.69 | No | Yes | No |
| Wang RY, Yen LL et al., 2005 part 1 | No imagery used | 101.00 | 51.30 | Yes | Yes | No |
| Wang RY, Yen LL et al., 2005 part 2 | No imagery used | 1043.60 | 1104.90 | No | No | Yes |
| Wang TC et al., 2015 | Use of imagery reported without description of lesion | 191.625 | 487.8841 | No | No | Yes |
| Xie et al., 2018 | Use of imagery reported without description of lesion | 439.15 | 615.82 | No | Yes | Yes |
| Xing et al., 2007 | Use of imagery reported without description of lesion | 19.64 | 11.88 | No | No | No |
| Yadav et al., 2015 | No imagery used | 588.50 | 490.01 | No | No | Yes |
| Yeung et al., 2018 | No imagery used | 1899.30 | 1351.43 | No | No | Yes |
| Yoo et al., 2010 | No imagery used | 45.58 | 32.07 | Yes | Yes | No |
| Yoo et al., 2018 | No imagery used | 24.25 | nd | Yes | Yes | No |
| You et al., 2014 | Use of imagery reported without description of lesion | 24.34 | 18.96 | Yes | Yes | No |
| Yu et Cho, 2016 | No imagery used | 384.30 | 78.63 | No | No | Yes |
| Yun et al., 2018 | Use of imagery reported without description of lesion | 30.10 | 7.20 | Yes | Yes | No |
| Zhang et al., 2015 | Use of imagery reported without description of lesion | 4.52 | 2.02 | Yes | No | No |

Abbreviations: nd, not documented.
