# Supplementary material for: Limited evidence of physical therapy on balance after stroke: A systematic review and meta-analysis
Source: PLoS One. 2019 Aug 29;14(8):e0221700. doi: 10.1371/journal.pone.0221700 (PMC6715189; doi:10.1371/journal.pone.0221700)
Supplement: S4 Table — (DOCX) [file pone.0221700.s018.docx]

**S4 Table. Overall score of risk of bias and ethic statement for each study included**

| Study | Overall score of ROB  (/14 points) | Ethic committee | Helsinki statement |
| --- | --- | --- | --- |
| Allison et Dennett, 2007 | 9 | Yes | No |
| Arabzadeh et al., 2018 | 6 | Yes | No |
| Askim et al., 2010 | 7 | Yes | No |
| Au-Yeung et al., 2009 | 7 | Yes | No |
| Bae et al., 2015 | 7 | No | No |
| Barcala et al., 2011 | 6 | Yes | No |
| Brogardh et al., 2012 | 12 | Yes | Yes |
| Bunketorp-Kall et al., 2017 | 8 | Yes | No |
| Buyukavci et al., 2016 | 8 | Yes | Yes |
| Büyükvural Şen et al., 2015 | 6 | Yes | No |
| Cabanas-Valdés et al., 2015 | 10 | Yes | Yes |
| Chan KS et al., 2012 | 9 | No | No |
| Chen CH et al., 2010 | 6 | Yes | No |
| Chen CL et al., 2015 | 8 | Yes | No |
| Chen D et al., 2014 | 6 | No | No |
| Chen IC et al., 2002 | 6 | No | No |
| Chen JC et al., 2011 | 7 | Yes | No |
| Chen, 2018 | 5 | No | No |
| Chern et al., 2013 | 6 | No | No |
| Cho HY et al., 2013 | 9 | Yes | No |
| Cho KH et al., 2012 | 7 | Yes | No |
| Cho MK et al., 2015 | 8 | Yes | No |
| Choi HS et al., 2017 | 6 | Yes | Yes |
| Chu et al., 2015 | 7 | No | No |
| Chung et al., 2014 | 8 | Yes | No |
| Dault et al., 2003 | 5 | Yes | No |
| Dujovic et al., 2017 | 11 | Yes | Yes |
| Duncan et al., 1998 | 8 | No | No |
| Duncan et al., 2003 | 10 | Yes | No |
| Erbil et al., 2018 | 6 | Yes | No |
| Fernandez-Gonzalo et al., 2016 | 7 | Yes | No |
| Ferreira et al., 2017 | 6 | Yes | No |
| Fritz et al., 2013 | 7 | Yes | No |
| Furnari et al., 2014 | 7 | Yes | No |
| Geiger et al., 2001 | 7 | Yes | No |
| Ghanjal et al., 2014 | 6 | Yes | No |
| Globas et al., 2012 | 9 | Yes | No |
| Goliwas et al., 2017 | 6 | Yes | No |
| Han et al., 2016 | 6 | Yes | No |
| Hart et al., 2004 | 7 | No | No |
| Heller et al., 2005 | 7 | No | No |
| Hollands et al., 2015 | 9 | Yes | No |
| Holmgren et al., 2010 | 10 | Yes | No |
| Hosseini et al., 2012 | 8 | Yes | No |
| Howe et al., 2005 | 7 | Yes | No |
| Hsieh, 2019 | 8 | Yes | Yes |
| Hsu et al., 2013 | 8 | Yes | No |
| Huh et al., 2015 | 7 | Yes | Yes |
| Hung et al., 2016 | 7 | Yes | No |
| Hwang et al., 2015 | 9 | Yes | No |
| Immink et al., 2014 | 9 | Yes | No |
| In et al., 2016 | 9 | Yes | Yes |
| Janssen et al., 2008 | 6 | Yes | No |
| Jung et al., 2015 | 7 | Yes | No |
| Jung et al., 2017 | 8 | Yes | No |
| Kamps et Schule, 2005 | 6 | Yes | No |
| Karasu et al., 2018 | 10 | Yes | Yes |
| Katz-Leurer et al., 2006 | 8 | Yes | No |
| Khumsapsiri et al., 2018 | 9 | Yes | No |
| Kilinc et al., 2015 | 8 | Yes | No |
| Kim DH et al., 2008 | 7 | No | No |
| Kim JC et Lee, 2018 | 7 | Yes | No |
| Kim JH et al., 2009 | 8 | Yes | No |
| Kim JY et al., 2018 | 6 | Yes | No |
| Kim SL et Lee, 2018 | 6 | Yes | No |
| Kim YH et al., 2004 | 7 | No | No |
| Kim YM et al., 2009 | 8 | No | No |
| Knox et al., 2018 | 8 | Yes | No |
| Kunkel et al., 2013 | 8 | Yes | No |
| Kwong et al., 2018 | 8 | Yes | Yes |
| Langhammer et al., 2009 | 8 | Yes | Yes |
| Lau RWK et al., 2012 | 11 | Yes | Yes |
| Laufer, 2003 | 7 | Yes | No |
| Lee CH et al., 2014 | 10 | Yes | No |
| Lee D et al., 2016 | 8 | Yes | No |
| Lee HJ et al., 2018 | 7 | No | No |
| Lee MM et al., 2018 | 8 | Yes | No |
| Lee NK et al., 2013 | 7 | Yes | Yes |
| Lee SH et al., 2012 | 8 | Yes | No |
| Lee SW et al., 2013 | 8 | Yes | No |
| Liang et al., 2012 | 6 | Yes | No |
| Lin Q et al., 2015 | 8 | No | No |
| Lindvall et Forsberg, 2014 | 7 | Yes | No |
| Lisinski et al., 2012 | 7 | Yes | No |
| Liu-Ambrose et Eng, 2015 | 8 | Yes | Yes |
| Lu et al., 1997 | 6 | Yes | No |
| Lynch et al., 2007 | 9 | Yes | No |
| Marin et al., 2013 | 11 | Yes | Yes |
| Merkert et al., 2011 | 6 | Yes | No |
| Milczarek et al., 1993 | 7 | No | No |
| Mojica et al., 1988 | 8 | No | No |
| Moore JL et al., 2010 | 6 | Yes | No |
| Morioka et Yagi, 2003 | 8 | No | No |
| Mudie et al., 2002 | 7 | Yes | No |
| Nadeau et al., 2013 | 10 | Yes | Yes |
| Ng et al., 2016 | 8 | Yes | No |
| Nikamp et al., 2017 | 5 | Yes | No |
| Noh et al., 2008 | 9 | Yes | Yes |
| Ordahan et al., 2015 | 11 | Yes | Yes |
| Page et al., 2008 | 7 | Yes | No |
| Park D et al., 2018 | 8 | Yes | No |
| Park DS et al., 2017 | 9 | Yes | No |
| Park et al., 2014 | 8 | Yes | No |
| Park HK et al., 2018 | 7 | No | No |
| Park J et al., 2017 | 6 | Yes | No |
| Pollock et al., 2002 | 3 | Yes | No |
| Pomeroy et al., 2001 | 8 | Yes | No |
| Rajaratnam et al., 2013 | 10 | Yes | No |
| Robertson et al., 2010 | 10 | Yes | No |
| Rougier et Boudrahem, 2010 | 6 | Yes | Yes |
| Salgueiro et Marquez, 2018 | 6 | Yes | No |
| Sanchez-Mila et al., 2018 | 10 | Yes | No |
| Schmid et al., 2012 | 6 | Yes | No |
| Schuster et al., 2012 | 10 | Yes | Yes |
| Shatil et al., 2005 | 7 | Yes | No |
| Shin et al., 2016 | 10 | Yes | No |
| Simons et al., 2009 | 6 | Yes | No |
| Sohn et al., 2015 | 6 | No | No |
| Song et al., 2014 | 7 | No | No |
| Stein et al., 2014 | 7 | Yes | No |
| Suh et al., 2014 | 11 | Yes | No |
| Tan et al., 2014 | 8 | Yes | No |
| Tan et al., 2016 | 6 | Yes | No |
| Tian et al., 2014 | 8 | No | No |
| Tilikete et al., 2001 | 8 | No | No |
| Tripp and Krakow, 2014 | 9 | No | Yes |
| Tung et al., 2010 | 10 | Yes | No |
| Vahlberg et al., 2017 | 8 | Yes | Yes |
| VanNes et al., 2006 | 6 | Yes | No |
| Waldron et Bohannon, 1989 | 6 | No | No |
| Wang et al., 2017 | 8 | No | No |
| Wang RY, Lin PY et al., 2007 | 8 | Yes | No |
| Wang RY, Yen LL et al., 2005 part 1 | 7 | Yes | No |
| Wang RY, Yen LL et al., 2005 part 2 | 7 | Yes | No |
| Wang TC et al., 2015 | 9 | Yes | Yes |
| Xie et al., 2018 | 9 | Yes | No |
| Xing et al., 2007 | 7 | No | No |
| Yadav et al., 2015 | 7 | Yes | No |
| Yeung et al., 2018 | 7 | Yes | Yes |
| Yoo et al., 2010 | 7 | No | No |
| Yoo et al., 2018 | 8 | Yes | No |
| You et al., 2014 | 8 | Yes | No |
| Yu et Cho, 2016 | 6 | No | No |
| Yun et al., 2018 | 5 | Yes | No |
| Zhang et al., 2015 | 8 | No | No |

higher score means lower ROB, lower score means higher ROB

Abbreviations: ROB, risk of bias.
