# Supplementary material for: Limited evidence of physical therapy on balance after stroke: A systematic review and meta-analysis
Source: PLoS One. 2019 Aug 29;14(8):e0221700. doi: 10.1371/journal.pone.0221700 (PMC6715189; doi:10.1371/journal.pone.0221700)
Supplement: S6 Table — (DOCX) [file pone.0221700.s020.docx]

**S6 Table. Description of PT**

**S6A Table. Summary of comparisons**

| Characteristics | PT versus NT | PT versus ST/UC |
| --- | --- | --- |
| Design of comparison | | |
| Direct comparison (e.g. A vs B) | 33 | 34 |
| Comparison “on-top” (e.g. A+B versus C+B) | 58 | 47 |
| Comparator group (control treatment) | | |
| No treatment (NT) | 91 | NA |
| Sham treatment (ST) | NA | 33 |
| Usual care (UC) | NA | 48 |
| Main therapeutic goal of PT | | |
| PT aiming mainly at the recovery of balance (direct PT) | 38 | 7 |
| PT not specifically focused on the recovery of balance (indirect PT) | 53 | 74 |

Abbreviations: NA, not applicable; NT, no treatment; PT, physical therapy; ST, sham treatment; UC, usual care

**S6B Table. Summary of categories of PT investigated in studies included**

| Comparison PT versus NT | Comparison PT versus ST/UC |
| --- | --- |
| Acupuncture (5) | Acupuncture (0) |
| Assistive devices (19) | Assistive devices (2) |
| Constraint-induced therapy (4) | Constraint-induced therapy (0) |
| Functional task-training (32) | Functional task-training (24) |
| Functional task-training and other intervention (0) | Functional task-training and other intervention (1) |
| Functional task-training associated with musculoskeletal intervention and/or cardiopulmonary intervention (14) | Functional task-training associated with musculoskeletal intervention and/or cardiopulmonary intervention (15) |
| Functional task-training and neurophysiological intervention (1) | Functional task-training and neurophysiological intervention (1) |
| Musculoskeletal intervention and body awareness therapy (1) | Musculoskeletal intervention and body awareness therapy (0) |
| Musculoskeletal intervention by active strengthening (3) | Musculoskeletal intervention by active strengthening (6) |
| Musculoskeletal intervention by electrostimulation (4) | Musculoskeletal intervention by electrostimulation (12) |
| Musculoskeletal intervention by immobilization (3) | Musculoskeletal intervention by immobilization (1) |
| Musculoskeletal intervention by mobilization (1) | Musculoskeletal intervention by mobilization (1) |
| Respiratory training (1) | Respiratory training (1) |
| Sensory interventions (2) | Sensory interventions (17) |
| Other intervention (1) | Other intervention (0) |

(n) number of comparisons

Abbreviations: NT, no treatment; PT, physical therapy; ST, sham treatment; UC, usual care.

**S6C Table. Characteristics of interventions for each study included**

| Study | Intervention (PT) | Design of comparison | Comparison | Type/nature of PT of comparison | Category of PT |
| --- | --- | --- | --- | --- | --- |
| Allison et Dennett, 2007 | additional standing practice + CPT versus CPT | A+B vs B | PT vs NT | BT + sit to stand training | FTT and MI and/or CPI |
| Arabzadeh et al., 2018 | task-oriented exercise program versus traditional physiotherapy | A vs B | PT vs ST/UC | FTT + MS | FTT and MI and/or CPI |
| Askim et al., 2010 | intensive motor training after early supported discharge + standard training (PT) versus standard treatment (PT) after early supported discharge | A+B vs B | PT vs NT | FTT + CPI | FTT and MI and/or CPI |
| Au-Yeung et al., 2009 | Tai chi versus exercices | A vs B | PT vs ST/UC | Tai Chi | FTT |
| Bae et al., 2015 | lower-leg flexible kinesiology taping of paralyzed side versus lower-leg inflexible (sham) taping | A vs B | PT vs ST/UC | taping | MI by immobilization |
| Barcala et al., 2011 | Wii Fit program + CPT versus CPT | A+B vs C+B | PT vs ST or UC | BT + VR | FTT alone |
| Brogardh et al., 2012 | whole body vibration (standing static posture) versus sham whole body vibration (standing static posture) | A vs B | PT vs ST or UC | sensory intervention | Sensory intervention |
| Bunketorp-Kall et al., 2017 | multimodal interventions based on rhythm-and-music therapy versus UC | A vs B | PT vs ST/UC | FTT | FTT |
| Bunketorp-Kall et al., 2017 | multimodal interventions based on horse-riding therapy versus UC | A vs B | PT vs ST/UC | BT + FTT | FTT |
| Buyukavci et al., 2016 | trunk balance exercises + CR program versus CR program | A+B vs B | PT vs NT | BT | FTT |
| Büyükvural Şen et al., 2015 | isokinetic strengthening training (knee + ankle) + CR versus CR | A+B vs B | PT vs NT | MS | MI: active strengthening |
| Cabanas-Valdés et al., 2015 | core stability exercises + CT versus CT | A+B vs B | PT vs NT | truncal exercises / core stability exercises | FTT and MI and/or CPI |
| Chan KS et al., 2012 | whole body vibration versus sham vibration | A vs B | PT vs ST or UC | sensory intervention | Sensory intervention |
| Chen CH et al., 2010 | lateral wedge on the paretic side versus no lateral wedge | A vs 0 | PT vs NT | constraint-induced therapy | Constraint-induced therapy |
| Chen CH et al., 2010 | lateral wedge on the non paretic side versus no lateral wedge | A vs 0 | PT vs NT | constraint-induced therapy | Constraint-induced therapy |
| Chen CL et al., 2015 | anterior ankle-foot orthosis versus no anterior ankle-foot orthosis | A vs 0 | PT vs NT | orthoses | Assistive devices |
| Chen D et al., 2014 | FES + routine treatment (medication + basic rehabilitation) versus comfort stimulation | A+B vs C+B | PT vs ST or UC | FES | MI by electrostimulation |
| Chen D et al., 2014 | FES + routine treatment (medication + basic rehabilitation) versus comfort stimulation | A+B vs C+B | PT vs ST or UC | FES | MI by electrostimulation |
| Chen IC et al., 2002 | Visual feedback BT + CPT + OT versus CPT + OT | A+B vs B | PT vs NT | BT + biofeedback | FTT alone |
| Chen JC et al., 2011 | thermal stimulation + SR versus visits and discussions + SR | A+B vs C+B | PT vs ST or UC | sensory intervention | Sensory intervention |
| Chen, 2018 | Tai Chi Yunshou versus traditional rehabilitation | A vs B | PT vs ST/UC | Tai Chi | FTT |
| Chern et al., 2013 | shoes versus shoes + anterior ankle foot orthosis | A vs 0 | PT vs NT | orthoses | Assistive devices |
| Chern et al., 2013 | shoes versus shoes + posterior ankle foot orthosis | A vs 0 | PT vs NT | orthoses | Assistive devices |
| Cho HY et al., 2013 | TENS + PT (bobath) versus sham TENS + PT (bobath) | A+B vs C+B | PT vs ST or UC | sensory intervention | Sensory intervention |
| Cho KH et al., 2012 | BT with VR + SR versus SR | A+B vs B | PT vs NT | BT + VR | FTT alone |
| Cho MK et al., 2015 | treadmill GT with FES GM+TA + regular PT versus treadmill GT + sham FES GM+TA + regular PT | A+B vs C+B | PT vs ST or UC | FES | MI by electrostimulation |
| Cho MK et al., 2015 | treadmill GT with FES GM+TA + regular PT versus treadmill GT with FES TA + sham FES GM + regular PT | A+B vs C+B | PT vs ST or UC | FES | MI by electrostimulation |
| Cho MK et al., 2015 | treadmill GT with FES TA + sham FES GM + regular PT versus treadmill GT + sham FES GM+TA + regular PT | A+B vs C+B | PT vs ST or UC | FES | MI by electrostimulation |
| Choi HS et al., 2017 | game-based constraint-induced movement therapy + traditional PT versus traditional PT | A+B vs B | PT vs NT | BT + VR | FTT |
| Choi HS et al., 2017 | General game-based training + traditional PT versus traditional PT | A+B vs B | PT vs NT | BT + VR | FTT |
| Chu et al., 2015 | electroacupuncture stimulation of lateral side of Tianzhu (para-BL 10) + traditional acupuncture + CBT + CRT + routine treatment of neurology versus CBT + CRT + routine treatment of neurology | A+B vs B | PT vs NT | acupuncture | Acupuncture |
| Chung et al., 2014 | GT with FES (GM + TA) versus GT with sham FES (GM + TA) | A+B vs C+B | PT vs ST or UC | FES | MI by electrostimulation |
| Dault et al., 2003 | standing balance with visual feedback versus standing balance EO | A vs 0 | PT vs NT | BT + biofeedback | FTT alone |
| Dujovic et al., 2017 | Novel multi-pad functional electrical stimulation + CSR versus CSR | A+B vs B | PT vs NT | FES + GT | FTT and MI and/or CPI |
| Duncan et al., 1998 | exercise program at home versus UC at home or outpatient | A vs B | PT vs ST or UC | MS + FTT + NPI + CPI | FTT and MI and/or CPI |
| Duncan et al., 2003 | exercise program at home versus UC at home | A vs B | PT vs ST or UC | MS + CPI + FTT + muscle stretching + active MM + NPI | FTT and MI and/or CPI |
| Erbil et al., 2018 | Robot‐assisted training + PT versus PT | A+B vs C+B | PT vs ST/UC | GT + BWS + EMA + biofeedback | FTT and MI and/or CPI |
| Fernandez-Gonzalo et al., 2016 | Eccentric-overload flywheel resistance exercise training program versus daily routines | A vs B | PT vs ST/UC | MS | MI by active strengthening |
| Ferreira et al., 2017 | Insole with corrective elements specifically designed for equinovarus foot on paretic limb (+ an insole without corrective elements on the non-paretic limb) + CPT versus Placebo insoles without corrective elements + CPT | A+B vs C+B | PT vs ST/UC | corrective insole | Assistive devices |
| Fritz et al., 2013 | video games (Wii and PS) versus no treatment | A vs 0 | PT vs NT | BT + VR | FTT alone |
| Furnari et al., 2014 | hydrokinesitherapy + CPT versus CPT | A+B vs C+B | PT vs ST/UC | BT + MS + CPI + aquatic environment | FTT and MI and/or CPI |
| Geiger et al., 2001 | balance training with biofeedback + PT versus PT | A+B vs C+B | PT vs ST/UC | BT + biofeedback | FTT |
| Ghanjal et al., 2014 | observation and imitation of functional tasks + standard PT versus standard PT | A+B vs B | PT vs NT | observation and imitation of functional tasks | FTT alone |
| Ghanjal et al., 2014 | observation and imitation of functional tasks + standard PT versus observation of symbols (no functional tasks) + standard PT | A+B vs C+B | PT vs ST or UC | observation and imitation of functional tasks | FTT alone |
| Globas et al., 2012 | aerobic treadmill GT versus CPT | A vs B | PT vs ST or UC | GT + CPI | FTT and MI and/or CPI |
| Goliwas et al., 2017 | Sensorimotor stimulation of the paretic foot + PT versus PT | A+B vs C+B | PT vs ST/UC | sensory intervention (ankle, foot) + BT + MM (ankle, foot) + muscle stretching (ankle, foot) + NPI | FTT + other |
| Han et al., 2016 | Robot-assisted gait training + CR therapy versus CPT + CR therapy | A+B vs C+B | PT vs ST/UC | GT + BWS + EMA + biofeedback | FTT and MI and/or CPI |
| Hart et al., 2004 | Tai Chi Chuan versus balance group exercises | A vs B | PT vs ST or UC | Tai Chi | FTT alone |
| Heller et al., 2005 | BT with biofeedback (barofeedback) + CR (neuromotor therapy) versus CR (neuromotor therapy) | A+B vs B | PT vs NT | BT + biofeedback | FTT alone |
| Hollands et al., 2015 | Treadmill visual cue training + SR versus UC + SR | A+B vs C+B | PT vs ST/UC | GT + cue | FTT |
| Hollands et al., 2015 | Over-ground visual cue training + SR versus UC + SR | A+B vs C+B | PT vs ST/UC | GT + cue | FTT |
| Holmgren et al., 2010 | high intensity functional exercises versus educational program | A vs B | PT vs NT | FTT + exercise + movement + intensive | FTT and MI and/or CPI |
| Hosseini et al., 2012 | mental practice + PT versus PT | A+B vs B | PT vs ST/UC | mental imagery | FTT |
| Howe et al., 2005 | lateral weight transference exercises + UC versus UC | A+B vs B | PT vs NT | BT | FTT alone |
| Hsieh, 2019 | Training with an adaptive foot switch and video games + regular physiotherapy + regular walking versus regular physiotherapy + regular walking | A+B vs B | PT vs NT | ankle movement | MI by mobilization |
| Hsu et al., 2013 | noxious thermal stimulation + PT + OT versus innocuous thermal stimulation + PT + OT | A+B vs C+B | PT vs ST or UC | sensory intervention | Sensory intervention |
| Huh et al., 2015 | balance training with a new lower limb balance control trainer + CR therapy versus CR therapy | A+B vs C+B | PT vs ST/UC | BT + biofeedback | FTT |
| Hung et al., 2016 | Tetrax biofeedback games + conventional outpatient rehabilitation therapy versus conventional outpatient rehabilitation therapy | A+B vs B | PT vs NT | BT + biofeedback | FTT |
| Hwang et al., 2015 | treadmill GT combined with FES using a tilt sensor + CPT versus treadmill GT combined with sham FES using a tilt sensor + CPT | A+B vs C+B | PT vs ST or UC | FES | MI by electrostimulation |
| Immink et al., 2014 | yoga (group + home) + usual treatment and lifestyle behavior versus wait list (no treatment) + usual treatment and lifestyle behavior | A+B vs B | PT vs NT | yoga | FTT alone |
| In et al., 2016 | virtual reality reflection therapy (mirror therapy) + CR program versus sham virtual reality reflection therapy + CR program | A+B vs C+B | PT vs ST/UC | mirror therapy (by VR) + FTT | FTT |
| Janssen et al., 2008 | leg cycling exercise with maximally tolerable electrical stimulation versus leg cycling exercise with just sensible electrical stimulation | A+B vs C+B | PT vs ST or UC | FES | MI by electrostimulation |
| Jung et al., 2015 | Trunk stabilization training on an unstable surface using visual feedback + general exercises versus general exercises | A+B vs B | PT vs NT | BT + biofeedback | FTT alone |
| Jung et al., 2017 | sit-to-stand training combined with transcutaneous electrical stimulation + CT versus sit-to-stand training combined with sham transcutaneous electrical stimulation + CT | A+B vs C+B | PT vs ST/UC | TENS | sensory intervention |
| Kamps et Schule, 2005 | Cyclic movement training of the lower limb versus CPT + conventional OT | A vs B | PT vs ST or UC | MS + CPI + assistance + resistance + biofeedback | MI: active strengthening |
| Karasu et al., 2018 | Wii Fit-based balance rehabilitation + CR versus CR | A+B vs B | PT vs NT | BT + VR | FTT |
| Katz-Leurer et al., 2006 | Early cycling training + regular rehabilitation versus regular rehabilitation | A+B vs B | PT vs NT | MS | MI: active strengthening |
| Khumsapsiri et al., 2018 | Training using a new multidirectional reach tool + CPT versus CPT | A+B vs B | PT vs NT | BT | FTT |
| Kilinc et al., 2015 | Bobath-based trunk training versus physiotherapy | A vs B | PT vs ST/UC | NPI + FTT | FTT and NPI |
| Kim DH et al., 2008 | Isokinetic strengthening of trunk muscles + NDT and GT versus NDT and GT | A+B vs B | PT vs NT | truncal exercise / core stability exercises | MI: active strengthening |
| Kim JC et Lee, 2018 | action observation physical training versus landscape imagery observation physical training | A+B vs C+B | PT vs ST/UC | observation and imitation of functional tasks | FTT |
| Kim JH et al., 2009 | VR + CPT versus CPT | A+B vs B | PT vs NT | BT + VR | FTT alone |
| Kim JY et al., 2018 | robot-(Morning Walk®) assisted gait training + conventional physiotherapy versus conventional physiotherapy | A+B vs C+B | PT vs ST/UC | GT + BWS + EMA + biofeedback | FTT and MI and/or CPI |
| Kim SL et Lee, 2018 | weight‐bearing‐based mobilization with movement + UC versus weight‐bearing with placebo mobilization with movement + UC | A+B vs C+B | PT vs ST/UC | MM | MI by mobilization |
| Kim YH et al., 2004 | static BT with visual biofeedback versus traditional rehabilitation | A vs B | PT vs ST or UC | BT + biofeedback | FTT alone |
| Kim YH et al., 2004 | dynamic BT with visual biofeedback versus traditional rehabilitation | A vs B | PT vs ST or UC | BT + biofeedback | FTT alone |
| Kim YM et al., 2009 | ES of trunc muscles + ES of lower limb muscles + PT + OT versus ES of lower limb muscles + PT + OT | A+B vs B | PT vs NT | FES | MI by electrostimulation |
| Knox et al., 2018 | task-oriented circuit gait training versus educational therapy | A vs B | PT vs ST/UC | FTT | FTT |
| Knox et al., 2018 | conventional strength training versus educational therapy | A vs B | PT vs ST/UC | MS | MI by active strengthening |
| Kunkel et al., 2013 | Exercises + UC versus UC | A+B vs B | PT vs NT | BT | FTT alone |
| Kunkel et al., 2013 | FES during exercises + UC versus UC | A+B vs B | PT vs NT | BT + FES | FTT and MI and/or CPI |
| Kwong et al., 2018 | Bilateral Transcutaneous Electrical Nerve Stimulation during functional task-oriented training versus Unilateral Transcutaneous Electrical Nerve Stimulation (on paretic lower limb et sham on non-paretic lower limb) during functional task-oriented training | A+B vs C+B | PT vs ST/UC | sensory intervention | Sensory intervention |
| Langhammer et al., 2009 | Specific intensive exercises versus exercises (UC) | A vs B | PT vs ST or UC | MS + CPI + FTT | FTT and MI and/or CPI |
| Lau RWK et al., 2012 | whole body vibration + (while) exercise and BT versus sham whole body vibration + (while) exercise and BT | A+B vs C+B | PT vs ST or UC | sensory intervention | Sensory intervention |
| Laufer, 2003 | Standard cane versus no cane | A vs 0 | PT vs NT | Cane | Assistive devices |
| Laufer, 2003 | Quad cane versus no cane | A vs 0 | PT vs NT | Cane | Assistive devices |
| Lee CH et al., 2014 | VR + general PT versus general PT | A+B vs B | PT vs NT | BT + VR | FTT alone |
| Lee D et al., 2016 | Mirror Therapy combined with NeuroMuscular Electrical Stimulation + CPT versus CPT | A+B vs B | PT vs NT | FES + mirror therapy | MI by electrostimulation |
| Lee HJ et al., 2018 | diaphragm and deep abdominal muscle exercise versus Traditional exercise | A vs B | PT vs ST/UC | respiratory training | Respiratory training |
| Lee MM et al., 2018 | game-based VR canoe paddling training + CPR versus CPR | A+B vs B | PT vs NT | BT + VR | FTT |
| Lee NK et al., 2013 | Close kinetic chain exercises versus routine activities (no regular exercise program) | A vs B | PT vs ST or UC | MS | MI: active strengthening |
| Lee NK et al., 2013 | Open kinetic chain exercises versus routine activities (no regular exercise program) | A vs B | PT vs ST or UC | MS | MI: active strengthening |
| Lee SH et al., 2012 | Visual feedback BT (standing) + CPT versus CPT | A+B vs B | PT vs NT | BT + biofeedback | FTT alone |
| Lee SW et al., 2013 | Local vibration stimulus training during weight-bearing and weight-shift training + SR versus sham local vibration stimulus training during weight-bearing and weight-shift training + SR | A+B vs C+B | PT vs ST or UC | sensory intervention | Sensory intervention |
| Liang et al., 2012 | Thermal stimulation + PT + OT versus PT + OT + discussion sessions | A+B vs C+B | PT vs ST or UC | sensory intervention | Sensory intervention |
| Lin Q et al., 2015 | Acupuncture + routine rehabilitation training versus routine rehabilitation training | A+B vs B | PT vs NT | acupuncture | Acupuncture |
| Lindvall et Forsberg, 2014 | Body awareness therapy + usual daily activities, ongoing PT or other training versus no treatment + usual daily activities, ongoing PT or other training | A+B vs B | PT vs NT | MM + body awareness therapy | MI and body awareness therapy |
| Lisinski et al., 2012 | BT with visual feedback versus no treatment | A vs 0 | PT vs NT | BT + biofeedback | FTT alone |
| Liu-Ambrose et Eng, 2015 | immediate community-based exercises + recreation and leisure activities versus UC | A vs B | PT vs ST or UC | MS + CPI + FTT | FTT and MI and/or CPI |
| Lu et al., 1997 | greater trochanter length cane versus no cane | A vs 0 | PT vs NT | Cane | Assistive devices |
| Lu et al., 1997 | wrist crease length cane versus no cane | A vs 0 | PT vs NT | Cane | Assistive devices |
| Lynch et al., 2007 | sensory retraining + standard PT versus relaxation + standard standard PT | A+B vs C+B | PT vs ST or UC | sensory intervention + relearning | Sensory intervention |
| Marin et al., 2013 | whole body vibration during isometric position exercises + SR versus isometric position (without vibration) + SR | A+B vs C+B | PT vs ST or UC | sensory intervention | Sensory intervention |
| Merkert et al., 2011 | vibration training + conventional comprehensive geriatric rehabilitation versus conventional comprehensive geriatric rehabilitation | A+B vs B | PT vs NT | sensory intervention | Sensory intervention |
| Milczarek et al., 1993 | four-footed cane versus no cane | A vs 0 | PT vs NT | Cane | Assistive devices |
| Milczarek et al., 1993 | standard cane versus no cane | A vs 0 | PT vs NT | Cane | Assistive devices |
| Mojica et al., 1988 | ankle-foot orthosis versus no ankle-foot orthosis | A vs 0 | PT vs NT | orthoses | Assistive devices |
| Moore JL et al., 2010 | intensive LT (intensity stepping practice + BWS) versus no treatment | A vs 0 | PT vs NT | GT + BWS + CPI | FTT and MI and/or CPI |
| Morioka et Yagi, 2003 | perceptual learning exercise + PT and OT versus PT and OT | A+B vs B | PT vs NT | sensory intervention + relearning | Sensory intervention |
| Mudie et al., 2002 | sitting BT with visual feedback + standard PT and OT versus standard PT and OT | A+B vs B | PT vs NT | BT + biofeedback | FTT alone |
| Mudie et al., 2002 | bobath + standard PT and OT versus standard PT and OT | A+B vs B | PT vs NT | BT (NPI) | FTT alone |
| Mudie et al., 2002 | sitting task-related reach training + standard PT and OT versus standard PT and OT | A+B vs B | PT vs NT | BT + task-related reach training | FTT alone |
| Nadeau et al., 2013 | GT + BWS + UC versus UC | A+B vs B | PT vs NT | GT + BWS | FTT and MI and/or CPI |
| Nadeau et al., 2013 | home exercise program + UC versus UC | A+B vs B | PT vs NT | FTT + MS + MM | FTT and MI and/or CPI |
| Ng et al., 2016 | Transcutaneous electrical nerve stimulation during task-oriented balance training + CR versus placebo-TENS during task-oriented balance training + CR | A+B vs C+B | PT vs ST/UC | sensory intervention | Sensory intervention |
| Nikamp et al., 2017 | Early ankle-foot orthosis + UC versus Delayed ankle-foot orthosis + UC | A+B vs B | PT vs NT | orthoses | Assistive devices |
| Noh et al., 2008 | aquatic therapy (Halliwick and Ai Chi methods) versus CT | A vs B | PT vs ST/UC | BT + aquatic environment | FTT |
| Ordahan et al., 2015 | Balance training with balance trainer + CR versus CR | A+B vs B | PT vs NT | BT + biofeedback + verticalization (support) | FTT and MI and/or CPI |
| Page et al., 2008 | bilateral reciprocal locomotor training with device versus home exercice program | A vs B | PT vs ST/UC | MS + EMR | MI by active strengthening |
| Park D et al., 2018 | talus-stabilizing taping versus barefoot | A vs 0 | PT vs NT | taping | MI by immobilization |
| Park D et al., 2018 | ankle foot orthosis versus barefoot | A vs 0 | PT vs NT | orthosis | Assistive devices |
| Park DS et al., 2017 | virtual reality training using Xbox Kinect + CPT versus CPT | A+B vs B | PT vs NT | BT + VR | FTT |
| Park et al., 2014 | TENS + (during) therapeutic exercises versus placebo TENS + (during) therapeutic exercises | A+B vs C+B | PT vs ST or UC | sensory intervention | Sensory intervention |
| Park HK et al., 2018 | land trunk exercise + aquatic trunk exercises (Halliwick) + CPT versus CPT | A+B vs B | PT vs NT | truncal exercice / core stability exercises + BT + aquatic environment | FTT and MI and/or CPI |
| Park J et al., 2017 | boxing program + CPT versus CPT | A+B vs B | PT vs NT | FTT + muscle stretching | FTT and MI and/or CPI |
| Pollock et al., 2002 | independent practice of motor task training + standard PT (bobath) versus standard PT (bobath) | A+B vs B | PT vs NT | BT + FTT | FTT alone |
| Pomeroy et al., 2001 | weight garments versus no weighted garment | A vs 0 | PT vs NT | constraint-induced therapy | Constraint-induced therapy |
| Rajaratnam et al., 2013 | BT with biofeedback + CR versus CR | A+B vs C+B | PT vs ST or UC | BT + VR | FTT alone |
| Robertson et al., 2010 | FES versus no FES | A vs 0 | PT vs NT | FES | MI by electrostimulation |
| Rougier et Boudrahem, 2010 | standing balance with visual feedback versus standing balance EO | A vs 0 | PT vs NT | BT + biofeedback | FTT alone |
| Salgueiro et Marquez, 2018 | visual and occulomotor training + CT versus CT | A+B vs B | PT vs NT | visual and occulomotor training | visual and occulomotor training |
| Sanchez-Mila et al., 2018 | dry needling + multimodal neuro-rehabilitation (bobath) versus multimodal neuro-rehabilitation (bobath) | A+B vs B | PT vs NT | acupuncture | Acupuncture |
| Schmid et al., 2012 | yoga +/- relaxation audio recording versus no treatment | A vs 0 | PT vs NT | yoga | FTT alone |
| Schuster et al., 2012 | PT + embedded motor imagery training versus PT + audio tapes with information related to stroke | A+B vs C+B | PT vs ST or UC | mental imagery | FTT alone |
| Schuster et al., 2012 | PT + added motor imagery training versus PT + audio tapes with information related to stroke | A+B vs C+B | PT vs ST or UC | mental imagery | FTT alone |
| Shatil et al., 2005 | Therapeutic golf rehabilitation (PT + golf) + regular routine versus hand therapy + regular routine (regular routine = no treatment) | A+B vs C+B | PT vs ST or UC | FTT + MS + CPI + MM + muscle stretching | FTT and MI and/or CPI |
| Shin et al., 2016 | Smartphone-based visual feedback trunk control training + CR versus CR | A+B vs B | PT vs NT | BT + biofeedback | FTT |
| Simons et al., 2009 | ankle-foot orthosis versus no ankle-foot orthosis | A vs 0 | PT vs NT | orthoses | Assistive devices |
| Sohn et al., 2015 | Bobath sling versus no sling | A vs 0 | PT vs NT | Sling | MI by immobilization |
| Sohn et al., 2015 | simple arm sling versus no sling | A vs 0 | PT vs NT | Sling | MI by immobilization |
| Song et al., 2014 | tetra-ataxiometric posturography program + CBT versus CBT | A+B vs B | PT vs NT | BT + biofeedback | FTT alone |
| Song et al., 2014 | VR during BT + CBT versus CBT | A+B vs B | PT vs NT | BT + VR | FTT alone |
| Stein et al., 2014 | PT with robotic knee brace versus exercises | A vs B | PT vs ST/UC | FTT + EMA | FTT and MI and/or CPI |
| Suh et al., 2014 | Interferential current therapy + SR versus sham Interferential current therapy + SR | A+B vs C+B | PT vs ST or UC | FES | MI by electrostimulation |
| Tan et al., 2014 | four-channel FES (tibialis anterior, quadriceps, hamstrings, and gastrocnemius of the affected leg) + CR versus placebo four-channel FES (tibialis anterior, quadriceps, hamstrings, and gastrocnemius of the affected leg) + CR | A+B vs C+B | PT vs ST or UC | FES | MI by electrostimulation |
| Tan et al., 2016 | Four-channel functional electrical stimulation + medicines and routine rehabilitation training versus Placebo functional electrical stimulation + medicines and routine rehabilitation training | A+B vs C+B | PT vs ST/UC | FES | MI by electrostimulation |
| Tan et al., 2016 | Single channel functional electrical stimulation + medicines and routine rehabilitation training versus Placebo functional electrical stimulation + medicines and routine rehabilitation training | A+B vs C+B | PT vs ST/UC | FES | MI by electrostimulation |
| Tian et al., 2014 | Acupuncture + medical treatment versus medical treatment | A+B vs B | PT vs NT | acupuncture | Other intervention |
| Tilikete et al., 2001 | prism adaptation (optical shift 10 deg to the right) versus sham prism adaptation (no optical shift) | A vs B | PT vs ST or UC | sensory intervention | Sensory intervention |
| Tilikete et al., 2001 | prism adaptation (optical shift 10 deg to the left) versus sham prism adaptation (no optical shift) | A vs B | PT vs ST or UC | sensory intervention | Sensory intervention |
| Tripp and Krakow, 2014 | Halliwick-therapy + CPT versus CPT | A+B vs C+B | PT vs ST or UC | BT + aquatic therapy | FTT alone |
| Tung et al., 2010 | sit-to-stand + general PT versus general PT | A+B vs B | PT vs NT | sit to stand training | FTT alone |
| Vahlberg et al., 2017 | progressive resistance and balance exercise program + motivational group discussions versus regular activities | A vs B | PT vs NT | FTT + MS + CPI | FTT and MI and/or CPI |
| VanNes et al., 2006 | whole body vibration (standing posture) + PT rehabilitation (individualized and group) (+ occupational and speech therapy and psychologic treatment) versus exercise therapy on music + PT rehabilitation (individualized and group) (+ occupational and speech therapy and psychologic treatment) | A+B vs C+B | PT vs ST or UC | sensory intervention | Sensory intervention |
| Waldron et Bohannon, 1989 | cane (cane - elbow 40 deg) versus no cane | A vs 0 | PT vs NT | Cane | Assistive devices |
| Waldron et Bohannon, 1989 | cane (cane - elbow 30 deg) versus no cane | A vs 0 | PT vs NT | Cane | Assistive devices |
| Waldron et Bohannon, 1989 | cane (cane - elbow 20 deg) versus no cane | A vs 0 | PT vs NT | Cane | Assistive devices |
| Wang et al., 2017 | Mirror therapy + CR versus passive or active supplementary training of the lower extremities of the affected side (as mirror therapy) + CR | A+B vs C+B | PT vs ST/UC | mirror therapy | FTT |
| Wang RY, Lin PY et al., 2007 | Ankle-foot orthosis versus no ankle-foot orthosis | A vs 0 | PT vs NT | orthoses | Assistive devices |
| Wang RY, Yen LL et al., 2005 part 1 | ankle-foot orthosis versus no ankle-foot orthosis | A vs 0 | PT vs NT | orthoses | Assistive devices |
| Wang RY, Yen LL et al., 2005 part 2 | ankle-foot orthosis versus no ankle-foot orthosis | A vs 0 | PT vs NT | orthoses | Assistive devices |
| Wang TC et al., 2015 | caregiver-mediated training (home-based intervention) versus routine care + visits or telephone calls | A vs B | PT vs ST or UC | MS + MM + FTT | FTT and MI and/or CPI |
| Xie et al., 2018 | Tai Chi Yunshou exercise versus Balance rehabilitation training | A vs B | PT vs ST/UC | Tai Chi | FTT |
| Xing et al., 2007 | acupuncture + hyperbaric oxygen therapy + conventional drug treatment versus hyperbaric oxygen therapy + conventional drug treatment | A+B vs B | PT vs NT | acupuncture | Other intervention |
| Yadav et al., 2015 | specific balance strategy training versus general balance exercise | A vs B | PT vs ST/UC | BT + muscle stretching + MS | FTT and MI and/or CPI |
| Yeung et al., 2018 | gait training + Robot-assisted ankle foot orthosis with dorsiflexion assistance versus gait training + sham Robot-assisted ankle foot orthosis with dorsiflexion assistance | A vs B | PT vs ST/UC | EMA in orthoses | Assistive devices |
| Yoo et al., 2010 | core strengthening program + CPT versus CPT | A+B vs B | PT vs NT | FTT + truncal exercises / core stability exercises (MS) | FTT and MI and/or CPI |
| Yoo et al., 2018 | bedside respiratory muscle training + CSR versus CSR | A+B vs B | PT vs NT | respiratory training | Respiratory training |
| You et al., 2014 | FES + SR (PT + OT) versus SR (PT + OT) | A+B vs B | PT vs NT | FES | MI by electrostimulation |
| Yu et Cho, 2016 | Virtual reality game + SR program versus SR program | A+B vs B | PT vs NT | BT + VR | FTT |
| Yun et al., 2018 | Robot-assisted gait training versus CPT | A vs B | PT vs ST/UC | GT + BWS + EMA + biofeedback | FTT and MI and/or CPI |
| Zhang et al., 2015 | modified CITUL + routine rehabilitation versus routine rehabilitation | A+B vs B | PT vs NT | CITUL | Constraint-induced therapy |

Abbreviations: BT, balance training; BWS, body weight support; CBT, conventional balance training; CITUL, constraint-induced movement therapy of upper limb; CPI, cardiopulmonary intervention; CPT, conventional physical therapy; CPR, conventional physical rehabilitation; CR, conventional rehabilitation; CRT, comprehensive rehabilitation therapy; CSR, conventional stroke rehabilitation; CT, conventional therapy; Deg, degree; EMA, electromechanical assistance; EMR, electromechanical resistance; EO, eyes opened; FES, functional electrostimulation; FTT, functional task training; GM, gluteus medius; GT, gait training; LT, locomotor training; LT-RGO, locomotor training with a robotic gait orthosis; MI, musculoskeletal intervention; MM, musculoskeletal mobilization; MS, muscle strengthening; Nd, not documented; NDT, neurodevelopmental treatment; NPI, neurophysiological intervention; OT, occupational therapy; PT, physical therapy; rTMS, repetitive transcranial magnetic stimulation; SR, standard rehabilitation; TA, tibial anterior; tDCS, transcranial direct current stimulation; TENS, transcutaneous electrical nerve stimulation; UC, usual care; VR, virtual reality
