# Supplementary material for: Limited evidence of physical therapy on balance after stroke: A systematic review and meta-analysis
Source: PLoS One. 2019 Aug 29;14(8):e0221700. doi: 10.1371/journal.pone.0221700 (PMC6715189; doi:10.1371/journal.pone.0221700)
Supplement: S7 Table — (DOCX) [file pone.0221700.s021.docx]

**S7 Table. Duration of PT**

**S7A Table. Summary of duration**

|  | PT versus NT | | PT versus ST/UC | | |
| --- | --- | --- | --- | --- | --- |
| All interventions (comparison + concomitant therapy) | | | | | |
|  | Experimental group | Control group | | Experimental group | Control group |
|  | mean +/- standard deviation / median / minimum-maximum | | | | |
| Mean duration of session (min) | 58.3 +/- 76.9 / 48.3 / 0.5-600.0 | 33.1 +/- 39.0 / 30.0 / 0.0-160.0 | | 67.0 +/- 41.8 / 60.0 / 3.0-236.5 | 66.8 +/- 44.3 / 60.0 / 3.0-272.0 |
| Number of sessions by week | 3.8 +/- 3.0 / 4.0 / 0.8-21.9 | 2.9 +/- 2.7 / 3.0 / 0.0-14.5 | | 3.5 +/- 1.7 / 3.0 / 0.5-10.0 | 3.5 +/- 1.7 / 3.0 / 0.1-6.0 |
| Number of weeks | 3.7 +/- 2.9 / 3.0 / 1.0-12.0 | 3.7 +/- 2.9 / 3.0 / 1.0-12.0 | | 6.6 +/- 6.6 / 6.0 / 1.0-52.0 | 6.3 +/- 5.3 / 6.0 / 1.0-40.0 |
| Total number of sessions | 17.4 +/- 22.0 / 15.0 / 1.0-164.0 | 12.3 +/- 14.4 / 11.0 / 0.0-78.6 | | 21.2 +/- 22.3 / 17.0 / 1.0-160.0 | 17.9 +/- 14.1 / 15.0 / 1.0-84.0 |
| Total duration (min) | 1444.3 +/- 3043.8 / 720.0 / 0.5-25200.0 | 711.0 +/- 1153.8 / 360.0 / 0.0-7071.0 | | 1402.0 +/- 1282.4 / 960.0 / 3.0-5700.0 | 1203.0 +/- 1127.7 / 900.0 / 3.0-5700.0 |
| Comparison only | | | | | |
|  | mean +/- standard deviation / median / minimum-maximum | | | | |
| Mean duration of session (min) | 33.1 +/- 67.6 / 20.2 / 0.5-600.0 | | 40.5 +/- 32.0 / 30.0 / 1.0-236.5 | | |
| Number of sessions by week | 3.3 +/- 1.9 / 3.0 / 1.0-7.5 | | 3.4 +/- 1.7 / 3.0 / 0.5-10.0 | | |
| Number of weeks | 3.7 +/- 2.9 / 3.0 / 1.0-12.0 | | 6.6 +/- 6.6 / 5.0 / 1.0-52.0 | | |
| Total number of sessions | 14.3 +/- 14.4 / 12.0 / 1.0-72.0 | | 20.7 +/- 22.3 / 16.0 / 1.0-160.0 | | |
| Total duration (min) | 811.6 +/- 2821.1 / 300.0 / 0.5-25200.0 | | 911.4 +/- 1054.9 / 570.0 / 3.0-5460.0 | | |
| Trial with duration of PT ≥30min per working day | 24 | | 43 | | |

Abbreviations: min, minute; NT, no treatment; PT, physical therapy; ST, sham treatment; UC, usual care;

**S7B Table. Duration of PT compared for each study included**

| Study | Type/nature of PT of comparison | Comparison | | | | |
| --- | --- | --- | --- | --- | --- | --- |
|  |  | Mean duration of session (min) | Weekly frequency of session | Number of weeks | Number of session | Overall duration (min) |
| Allison et Dennett, 2007 | BT + sit to stand training | 45.00 | 5.00 | 2.00 | 10.00 | 450.00 |
| Arabzadeh et al., 2018 | FTT + MS | 50.00 | 3.00 | 4.00 | 12.00 | 600.00 |
| Askim et al., 2010 | FTT + CPI | 19.09 | 6.00 | 12.00 | 72.00 | 1374.80 |
| Au-Yeung et al., 2009 | Tai Chi | 236.50 | 1.00 | 12.00 | 11.30 | 2838.00 |
| Bae et al., 2015 | taping | 5.00 | 1.00 | 1.00 | 1.00 | 5.00 |
| Barcala et al., 2011 | BT + VR | 30.00 | 2.00 | 5.00 | 10.00 | 300.00 |
| Brogardh et al., 2012 | sensory intervention | 45.00 | 2.00 | 6.00 | 12.00 | 540.00 |
| Bunketorp-Kall et al., 2017 | FTT | 90.00 | 2.00 | 12.00 | 24.00 | 2160.00 |
| Bunketorp-Kall et al., 2017 | BT + FTT | 120.00 | 2.00 | 12.00 | 24.00 | 2880.00 |
| Buyukavci et al., 2016 | BT | 120.00 | 3.00 | 3.00 | 9.00 | 1080.00 |
| Büyükvural Şen et al., 2015 | MS | nd | 5.00 | 3.00 | nd | nd |
| Cabanas-Valdés et al., 2015 | truncal exercises / core stability exercises | 14.76 | 5.00 | 5.00 | 25.00 | 369.00 |
| Chan KS et al., 2012 | sensory intervention | 21.00 | 1.00 | 1.00 | 1.00 | 21.00 |
| Chen CH et al., 2010 | constraint-induced therapy | 3.00 | 1.00 | 1.00 | 1.00 | 3.00 |
| Chen CH et al., 2010 | constraint-induced therapy | 3.00 | 1.00 | 1.00 | 1.00 | 3.00 |
| Chen CL et al., 2015 | orthoses | 5.00 | 1.00 | 1.00 | 1.00 | 5.00 |
| Chen D et al., 2014 | FES | 30.00 | 5.00 | 3.00 | 15.00 | 450.00 |
| Chen D et al., 2014 | FES | 30.00 | 5.00 | 3.00 | 15.00 | 450.00 |
| Chen IC et al., 2002 | BT + biofeedback | 20.00 | 5.00 | 2.00 | 10.00 | 200.00 |
| Chen JC et al., 2011 | sensory intervention | 48.00 | 5.00 | 6.00 | 30.00 | 1440.00 |
| Chen, 2018 | Tai Chi | nd | nd | 6.00 | nd | nd |
| Chern et al., 2013 | orthoses | 2.00 | 1.00 | 1.00 | 1.00 | 2.00 |
| Chern et al., 2013 | orthoses | 2.00 | 1.00 | 1.00 | 1.00 | 2.00 |
| Cho HY et al., 2013 | sensory intervention | 60.00 | 1.00 | 1.00 | 1.00 | 60.00 |
| Cho KH et al., 2012 | BT + VR | 30.00 | 3.00 | 6.00 | 18.00 | 540.00 |
| Cho MK et al., 2015 | FES | 30.00 | 5.00 | 4.00 | 20.00 | 600.00 |
| Cho MK et al., 2015 | FES | 30.00 | 5.00 | 4.00 | 20.00 | 600.00 |
| Cho MK et al., 2015 | FES | 30.00 | 5.00 | 4.00 | 20.00 | 600.00 |
| Choi HS et al., 2017 | BT + VR | 30.00 | 3.00 | 4.00 | 12.00 | 360.00 |
| Choi HS et al., 2017 | BT + VR | 30.00 | 3.00 | 4.00 | 12.00 | 360.00 |
| Chu et al., 2015 | acupuncture | nd | 5.00 | 8.00 | 40.00 | nd |
| Chung et al., 2014 | FES | 30.00 | 5.00 | 6.00 | 30.00 | 900.00 |
| Dault et al., 2003 | BT + biofeedback | 1.50 | 1.00 | 1.00 | 1.00 | 1.50 |
| Dujovic et al., 2017 | FES + GT | 30.00 | 5.00 | 4.00 | 20.00 | 600.00 |
| Duncan et al., 1998 | MS + FTT + NPI + CPI | 90.00 | 3.00 | 12.00 | 36.00 | 3240.00 |
| Duncan et al., 2003 | MS + CPI + FTT + muscle stretching + active MM + NPI | 91.00 | 2.57 | 13.00 | 33.40 | 3039.00 |
| Erbil et al., 2018 | GT + BWS + EMA + biofeedback | 30.00 | 5.00 | 3.00 | 15.00 | 450.00 |
| Fernandez-Gonzalo et al., 2016 | MS | 20.00 | 2.00 | 12.00 | 24.00 | 480.00 |
| Ferreira et al., 2017 | corrective insole | nd | nd | 12.00 | nd | nd |
| Fritz et al., 2013 | BT + VR | 50.00 | 4.00 | 5.00 | 20.00 | 1000.00 |
| Furnari et al., 2014 | BT + MS + CPI + aquatic environment | 60.00 | 3.00 | 8.00 | 24.00 | 1440.00 |
| Geiger et al., 2001 | BT + biofeedback | 15.00 | 2.50 | 4.00 | 10.00 | 150.00 |
| Ghanjal et al., 2014 | observation and imitation of functional tasks | 15.00 | nd | nd | 12.00 | 180.00 |
| Ghanjal et al., 2014 | observation and imitation of functional tasks | 15.00 | nd | nd | 12.00 | 180.00 |
| Globas et al., 2012 | GT + CPI | 40.00 | 2.95 | 13.20 | 39.00 | 1560.00 |
| Goliwas et al., 2017 | sensory intervention (ankle, foot) + BT + MM (ankle, foot) + muscle stretching (ankle, foot) + NPI | 20.00 | 4.17 | 6.00 | 25.00 | 500.00 |
| Han et al., 2016 | GT + BWS + EMA + biofeedback | 30.00 | 5.00 | 4.00 | 20.00 | 600.00 |
| Hart et al., 2004 | Tai Chi | 60.00 | 2.00 | 12.00 | 24.00 | 1440.00 |
| Heller et al., 2005 | BT + biofeedback | 30.00 | 5.00 | 7.86 | 39.29 | 1178.57 |
| Hollands et al., 2015 | GT + cue | 60.00 | 2.00 | 8.00 | 16.00 | 960.00 |
| Hollands et al., 2015 | GT + cue | 60.00 | 2.00 | 8.00 | 16.00 | 960.00 |
| Holmgren et al., 2010 | FTT + exercise + movement + intensive | 72.00 | 6.00 | 5.00 | 30.00 | 2160.00 |
| Hosseini et al., 2012 | mental imagery | 15.00 | nd | nd | nd | nd |
| Howe et al., 2005 | BT | 25.90 | nd | 4.00 | 10.00 | 258.95 |
| Hsieh, 2019 | ankle movement | 30.00 | 7.00 | 10.00 | 70.00 | 2100.00 |
| Hsu et al., 2013 | sensory intervention | 30.00 | 3.00 | 8.00 | 24.00 | 720.00 |
| Huh et al., 2015 | BT + biofeedback | 30.00 | 5.00 | 2.00 | 10.00 | 300.00 |
| Hung et al., 2016 | BT + biofeedback | 20.00 | 3.00 | 6.00 | 18.00 | 360.00 |
| Hwang et al., 2015 | FES | 30.00 | 5.00 | 4.00 | 20.00 | 600.00 |
| Immink et al., 2014 | yoga | 57.31 | 5.20 | 10.00 | 52.00 | 2980.00 |
| In et al., 2016 | mirror therapy (by VR) + FTT | 30.00 | 5.00 | 4.00 | 20.00 | 600.00 |
| Janssen et al., 2008 | FES | 27.50 | 2.00 | 6.00 | 12.00 | 330.00 |
| Jung et al., 2015 | BT + biofeedback | 20.00 | 3.00 | 4.00 | 12.00 | 240.00 |
| Jung et al., 2017 | TENS | 30.00 | 5.00 | 6.00 | 30.00 | 900.00 |
| Kamps et Schule, 2005 | MS + CPI + assistance + resistance + biofeedback | 18.87 | 10.00 | 16.00 | 160.00 | 3019.00 |
| Karasu et al., 2018 | BT + VR | 20.00 | 5.00 | 4.00 | 20.00 | 400.00 |
| Katz-Leurer et al., 2006 | MS | 25.07 | 5.00 | 3.00 | 15.00 | 376.00 |
| Khumsapsiri et al., 2018 | BT | 30.00 | 3.00 | 4.00 | 12.00 | 360.00 |
| Kilinc et al., 2015 | NPI + FTT | 60.00 | 3.00 | 12.00 | 36.00 | 2160.00 |
| Kim DH et al., 2008 | truncal exercise / core stability exercises | nd | 3.00 | 4.00 | 12.00 | nd |
| Kim JC et Lee, 2018 | observation and imitation of functional tasks | 10.00 | 3.00 | 3.00 | 9.00 | 90.00 |
| Kim JH et al., 2009 | BT + VR | 30.00 | 4.00 | 4.00 | 16.00 | 480.00 |
| Kim JY et al., 2018 | GT + BWS + EMA + biofeedback | 30.00 | 5.00 | 3.00 | 15.00 | 450.00 |
| Kim SL et Lee, 2018 | MM | 80.00 | 5.00 | 4.00 | 20.00 | 1600.00 |
| Kim YH et al., 2004 | BT + biofeedback | 30.00 | 3.00 | 3.00 | 9.00 | 270.00 |
| Kim YH et al., 2004 | BT + biofeedback | 30.00 | 3.00 | 3.00 | 9.00 | 270.00 |
| Kim YM et al., 2009 | FES | 30.00 | 5.00 | 3.00 | 15.00 | 450.00 |
| Knox et al., 2018 | FTT | 60.00 | 0.50 | 12.00 | 6.00 | 360.00 |
| Knox et al., 2018 | MS | 60.00 | 0.50 | 12.00 | 6.00 | 360.00 |
| Kunkel et al., 2013 | BT | 20.38 | 4.00 | 2.00 | 8.00 | 163.00 |
| Kunkel et al., 2013 | BT + FES | 24.50 | 4.00 | 2.00 | 8.00 | 196.00 |
| Kwong et al., 2018 | sensory intervention | 60.00 | 2.00 | 10.00 | 20.00 | 1200.00 |
| Langhammer et al., 2009 | MS + CPI + FTT | 50.00 | 2.10 | 52.00 | 109.20 | 5460.00 |
| Lau RWK et al., 2012 | sensory intervention | 10.50 | 3.00 | 8.00 | 24.00 | 252.00 |
| Laufer, 2003 | Cane | 0.50 | 1.00 | 1.00 | 1.00 | 0.50 |
| Laufer, 2003 | Cane | 0.50 | 1.00 | 1.00 | 1.00 | 0.50 |
| Lee CH et al., 2014 | BT + VR | 30.00 | 3.00 | 4.00 | 12.00 | 360.00 |
| Lee D et al., 2016 | FES + mirror therapy | nd | 5.00 | 4.00 | 20.00 | nd |
| Lee HJ et al., 2018 | respiratory training | 40.00 | 5.00 | 6.00 | 30.00 | 1200.00 |
| Lee MM et al., 2018 | BT + VR | 30.00 | 3.00 | 3.00 | 9.00 | 450.00 |
| Lee NK et al., 2013 | MS | nd | nd | nd | nd | nd |
| Lee NK et al., 2013 | MS | nd | nd | nd | nd | nd |
| Lee SH et al., 2012 | BT + biofeedback | 20.00 | 5.00 | 4.00 | 20.00 | 400.00 |
| Lee SW et al., 2013 | sensory intervention | 30.00 | 3.00 | 6.00 | 18.00 | 540.00 |
| Liang et al., 2012 | sensory intervention | 40.00 | 5.00 | 6.00 | 30.00 | 1200.00 |
| Lin Q et al., 2015 | acupuncture | 20.00 | 5.00 | 4.00 | 20.00 | 400.00 |
| Lindvall et Forsberg, 2014 | MM + body awareness therapy | 80.00 | 1.00 | 8.00 | 6.00 | 480.00 |
| Lisinski et al., 2012 | BT + biofeedback | nd | nd | 2.86 | nd | nd |
| Liu-Ambrose et Eng, 2015 | MS + CPI + FTT | 60.00 | 3.00 | 26.07 | 78.21 | 4692.60 |
| Lu et al., 1997 | Cane | 1.00 | 1.00 | 1.00 | 1.00 | 1.00 |
| Lu et al., 1997 | Cane | 1.00 | 1.00 | 1.00 | 1.00 | 1.00 |
| Lynch et al., 2007 | sensory intervention + relearning | 30.00 | 5.00 | 2.00 | 10.00 | 300.00 |
| Marin et al., 2013 | sensory intervention | 15.08 | 1.42 | 12.00 | 17.00 | 256.33 |
| Merkert et al., 2011 | sensory intervention | 4.97 | 7.50 | 2.00 | 15.00 | 74.54 |
| Milczarek et al., 1993 | Cane | 0.50 | 1.00 | 1.00 | 1.00 | 0.50 |
| Milczarek et al., 1993 | Cane | 0.50 | 1.00 | 1.00 | 1.00 | 0.50 |
| Mojica et al., 1988 | orthoses | 5.00 | 1.00 | 1.00 | 1.00 | 5.00 |
| Moore JL et al., 2010 | GT + BWS + CPI | nd | 3.50 | 4.00 | 14.00 | nd |
| Morioka et Yagi, 2003 | sensory intervention + relearning | nd | 5.00 | 2.00 | 10.00 | nd |
| Mudie et al., 2002 | BT + biofeedback | 30.00 | 5.00 | 2.00 | 10.00 | 300.00 |
| Mudie et al., 2002 | BT (NPI) | 30.00 | 5.00 | 2.00 | 10.00 | 300.00 |
| Mudie et al., 2002 | BT + task-related reach training | 30.00 | 5.00 | 2.00 | 10.00 | 300.00 |
| Nadeau et al., 2013 | GT + BWS | 76.58 | 3.00 | 12.00 | 33.00 | 2527.00 |
| Nadeau et al., 2013 | FTT + MS + MM | 79.31 | 3.00 | 12.00 | 36.00 | 2855.00 |
| Ng et al., 2016 | sensory intervention | 60.00 | 2.00 | 8.00 | 16.00 | 960.00 |
| Nikamp et al., 2017 | orthoses | nd | nd | 9.00 | nd | nd |
| Noh et al., 2008 | BT + aquatic environment | 60.00 | 3.00 | 8.00 | 24.00 | 1440.00 |
| Ordahan et al., 2015 | BT + biofeedback + verticalization (support) | 20.00 | 5.00 | 6.00 | 30.00 | 600.00 |
| Page et al., 2008 | MS + EMR | 38.50 | 3.00 | 8.00 | 24.00 | 924.00 |
| Park D et al., 2018 | taping | 15.00 | 1.00 | 1.00 | 1.00 | 15.00 |
| Park D et al., 2018 | orthosis | 15.00 | 1.00 | 1.00 | 1.00 | 15.00 |
| Park DS et al., 2017 | BT + VR | 30.00 | 5.00 | 6.00 | 30.00 | 900.00 |
| Park et al., 2014 | sensory intervention | 30.00 | 5.00 | 6.00 | 30.00 | 900.00 |
| Park HK et al., 2018 | truncal exercice / core stability exercises + BT + aquatic environment | 30.00 | 5.00 | 4.00 | 20.00 | 600.00 |
| Park J et al., 2017 | FTT + muscle stretching | 30.00 | 3.00 | 6.00 | 18.00 | 540.00 |
| Pollock et al., 2002 | BT + FTT | nd | 5.00 | 4.00 | 20.00 | nd |
| Pomeroy et al., 2001 | constraint-induced therapy | 600.00 | 7.00 | 6.00 | 42.00 | 25200.00 |
| Rajaratnam et al., 2013 | BT + VR | 20.00 | 1.00 | 1.00 | 1.00 | 20.00 |
| Robertson et al., 2010 | FES | 5.00 | 1.00 | 1.00 | 1.00 | 5.00 |
| Rougier et Boudrahem, 2010 | BT + biofeedback | 2.67 | 1.00 | 1.00 | 1.00 | 2.67 |
| Salgueiro et Marquez, 2018 | visual and occulomotor training | 15.00 | 1.67 | 3.00 | 5.00 | 75.00 |
| Sanchez-Mila et al., 2018 | acupuncture | 15.00 | 1.00 | 1.00 | 1.00 | 15.00 |
| Schmid et al., 2012 | yoga | 44.20 | 3.46 | 8.00 | 27.00 | 1193.51 |
| Schuster et al., 2012 | mental imagery | 15.00 | 3.00 | 2.00 | 6.00 | 90.00 |
| Schuster et al., 2012 | mental imagery | 15.00 | 3.00 | 2.00 | 6.00 | 90.00 |
| Shatil et al., 2005 | FTT + MS + CPI + MM + muscle stretching | 55.00 | 3.00 | 6.00 | 18.00 | 990.00 |
| Shin et al., 2016 | BT + biofeedback | 20.00 | 3.00 | 4.00 | 12.00 | 240.00 |
| Simons et al., 2009 | orthoses | 1.50 | 1.00 | 1.00 | 1.00 | 1.50 |
| Sohn et al., 2015 | Sling | 13.33 | 1.00 | 1.00 | 1.00 | 13.33 |
| Sohn et al., 2015 | Sling | 13.33 | 1.00 | 1.00 | 1.00 | 13.33 |
| Song et al., 2014 | BT + biofeedback | 25.00 | 3.00 | 3.00 | 9.00 | 225.00 |
| Song et al., 2014 | BT + VR | 25.00 | 3.00 | 3.00 | 9.00 | 225.00 |
| Stein et al., 2014 | FTT + EMA | 60.00 | 3.00 | 6.00 | 18.00 | 1080.00 |
| Suh et al., 2014 | FES | 60.00 | 1.00 | 1.00 | 1.00 | 60.00 |
| Tan et al., 2014 | FES | 30.00 | 5.00 | 3.00 | 15.00 | 450.00 |
| Tan et al., 2016 | FES | 30.00 | 5.00 | 3.00 | 15.00 | 450.00 |
| Tan et al., 2016 | FES | 30.00 | 5.00 | 3.00 | 15.00 | 450.00 |
| Tian et al., 2014 | acupuncture | 30.00 | 7.00 | 4.00 | 28.00 | 840.00 |
| Tilikete et al., 2001 | sensory intervention | 3.00 | 1.00 | 1.00 | 1.00 | 3.00 |
| Tilikete et al., 2001 | sensory intervention | 3.00 | 1.00 | 1.00 | 1.00 | 3.00 |
| Tripp and Krakow, 2014 | BT + aquatic therapy | 45.00 | 3.00 | 2.00 | 6.00 | 270.00 |
| Tung et al., 2010 | sit to stand training | 15.00 | 3.00 | 4.00 | 12.00 | 180.00 |
| Vahlberg et al., 2017 | FTT + MS + CPI | 75.00 | 2.50 | 12.00 | 30.00 | 2250.00 |
| VanNes et al., 2006 | sensory intervention | 1.00 | 5.00 | 6.00 | 30.00 | 29.89 |
| Waldron et Bohannon, 1989 | Cane | 15.00 | 1.00 | 1.00 | 1.00 | 15.00 |
| Waldron et Bohannon, 1989 | Cane | 15.00 | 1.00 | 1.00 | 1.00 | 15.00 |
| Waldron et Bohannon, 1989 | Cane | 15.00 | 1.00 | 1.00 | 1.00 | 15.00 |
| Wang et al., 2017 | mirror therapy | 40.00 | 5.00 | 6.00 | 30.00 | 1200.00 |
| Wang RY, Lin PY et al., 2007 | orthoses | 5.00 | 1.00 | 1.00 | 1.00 | 5.00 |
| Wang RY, Yen LL et al., 2005 part 1 | orthoses | 15.00 | 1.00 | 1.00 | 1.00 | 15.00 |
| Wang RY, Yen LL et al., 2005 part 2 | orthoses | 15.00 | 1.00 | 1.00 | 1.00 | 15.00 |
| Wang TC et al., 2015 | MS + MM + FTT | 100.00 | 2.00 | 12.00 | 24.00 | 2400.00 |
| Xie et al., 2018 | Tai Chi | 60.00 | 5.00 | 12.00 | 60.00 | 3600.00 |
| Xing et al., 2007 | acupuncture | 30.00 | 5.00 | 4.00 | 20.00 | 600.00 |
| Yadav et al., 2015 | BT + muscle stretching + MS | 60.00 | 5.00 | 2.00 | 10.00 | 600.00 |
| Yeung et al., 2018 | EMA in orthoses | 30.00 | 4.00 | 5.00 | 20.00 | 600.00 |
| Yoo et al., 2010 | FTT + truncal exercises / core stability exercises (MS) | 30.00 | 3.00 | 4.00 | 12.00 | 360.00 |
| Yoo et al., 2018 | respiratory training | 80.00 | 7.00 | 3.00 | 21.00 | 1680.00 |
| You et al., 2014 | FES | 30.00 | 5.00 | 3.00 | 15.00 | 450.00 |
| Yu et Cho, 2016 | BT + VR | 30.00 | 3.00 | 6.00 | 18.00 | 540.00 |
| Yun et al., 2018 | GT + BWS + EMA + biofeedback | 30.00 | 5.00 | 3.00 | 15.00 | 450.00 |
| Zhang et al., 2015 | CITUL | 100.00 | 5.00 | 6.00 | 30.00 | 3000.00 |

Abbreviations: BT, balance training; BWS, body weight support; CITUL, constraint-induced movement therapy of upper limb; CPI, cardiopulmonary intervention; EMA, electromechanical assistance; EMR, electromechanical resistance; FES, functional electrostimulation; FTT, functional task training; GT, gait training; Min, minute; MM, musculoskeletal mobilization; MS, muscle strengthening; Nd, not documented; NPI, neurophysiological intervention; PT, physical therapy; UC, usual care; VR, virtual reality
