# Supplementary material for: Limited evidence of physical therapy on balance after stroke: A systematic review and meta-analysis
Source: PLoS One. 2019 Aug 29;14(8):e0221700. doi: 10.1371/journal.pone.0221700 (PMC6715189; doi:10.1371/journal.pone.0221700)
Supplement: S8 Table — (DOCX) [file pone.0221700.s022.docx]

**S8 Table. Outcome measures**

**S8A Table. Summary of outcome measures**

| Measures of outcomes | Number of studies |
| --- | --- |
| Balance |  |
| BBS (post-intervention) | 98 (68%) |
| BBS (follow-up) | 31 (21%) |
| PASS (post-intervention) | 11 (8%) |
| PASS (follow-up) | 6 (4%) |
| Autonomy |  |
| Barthel index (post-intervention) | 30 (21%) |
| Barthel index (follow-up) | 13 (9%) |
| ADL (post-intervention) | 2 (1%) |
| IADL (post-intervention) | 0 (0%) |
| IADL (follow-up) | 4 (3%) |
| FIM (post-intervention) | 1 (1%) |
| FIM (follow-up) | 3 (2%) |
| Mediolateral and anteroposterior postural deviation (COP, WBA) |  |
| WB on paretic limb, EO (post-intervention) | 23 (16%) |
| WB on paretic limb, EO (follow-up) | 4 (3%) |
| WB on paretic limb, EC (post-intervention) | 2 (1%) |
| Mediolateral position of COP, EO (post-intervention) | 6 (4%) |
| Anteroposterior position of COP, EO (post-intervention) | 5 (3%) |
| Anteroposterior position of COP, EO (follow-up) | 1 (1%) |
| Postural stability (COP, LOS) |  |
| Sway length EO (post-intervention) | 13 (9%) |
| Sway area EO (post-intervention) | 10 (7%) |
| Sway length EC (post-intervention) | 9 (6%) |
| Velocity COP EO (post-intervention) | 9 (6%) |
| Anteroposterior velocity COP EO (post-intervention) | 8 (6%) |
| Mediolateral velocity COP EO (post-intervention) | 8 (6%) |
| Anteroposterior velocity COP EC (post-intervention) | 7 (5%) |
| LOS, maximum excursion, affected side EO (post-intervention) | 7 (5%) |
| LOS, maximum excursion, anterior, EO (post-intervention) | 7 (5%) |
| LOS, maximum excursion, non-affected side EO (post-intervention) | 7 (5%) |
| Mediolateral velocity COP EC (post-intervention) | 7 (5%) |
| LOS, Movement velocity, affected side EO (post-intervention) | 6 (4%) |
| LOS, Movement velocity, anterior, EO (post-intervention) | 6 (4%) |
| LOS, Movement velocity, non-affected side EO (post-intervention) | 6 (4%) |
| LOS, directional control non-affected side EO (post-intervention) | 5 (3%) |
| LOS, directional control, affected side EO (post-intervention) | 5 (3%) |
| LOS, directional control, anterior, EO (post-intervention) | 5 (3%) |
| LOS, maximum excursion, posterior EO (post-intervention) | 5 (3%) |
| LOS, Movement velocity, posterior EO (post-intervention) | 5 (3%) |
| LOS, directional control posterior EO (post-intervention) | 4 (3%) |
| Sway area EC (post-intervention) | 4 (3%) |
| Velocity COP EC (post-intervention) | 4 (3%) |
| Anteroposterior sway length EO (post-intervention) | 3 (2%) |
| LOS, end-point excursion, affected side, EO (post-intervention) | 3 (2%) |
| LOS, end-point excursion, backward, EO (post-intervention) | 3 (2%) |
| LOS, end-point excursion, forward, EO (post-intervention) | 3 (2%) |
| LOS, end-point excursion, non-affected side, EO (post-intervention) | 3 (2%) |
| Mediolateral sway length EO (post-intervention) | 3 (2%) |
| Anteroposterior variability COP EO (post-intervention) | 3 (2%) |
| Mediolateral variability COP EO (post-intervention) | 3 (2%) |
| Velocity moment EC (post-intervention) | 3 (2%) |
| Velocity moment EO (post-intervention) | 3 (2%) |
| LOS EO (post-intervention) | 2 (1%) |
| LOS, end-point excursion, affected side, EO (follow-up) | 2 (1%) |
| LOS, end-point excursion, backward, EO (follow-up) | 2 (1%) |
| LOS, end-point excursion, forward, EO (follow-up) | 2 (1%) |
| LOS, end-point excursion, non-affected side, EO (follow-up) | 2 (1%) |
| Maximum COP displacement in anteroposterior direction EO (post-intervention) | 2 (1%) |
| Maximum COP displacement in mediolateral direction EO (post-intervention) | 2 (1%) |
| Stability index EO (post-intervention) | 2 (1%) |
| Velocity COP EC (post-intervention) | 2 (1%) |
| Velocity COP EO (post-intervention) | 2 (1%) |
| Anteroposterior sway length EC (post-intervention) | 1 (1%) |
| COP excursion EO (post-intervention) | 1 (1%) |
| LOS, Ankle strategy, EC (follow up) | 1 (1%) |
| LOS, Ankle strategy, EO (follow up) | 1 (1%) |
| LOS, COG alignment, EC (follow up) | 1 (1%) |
| LOS, COG alignment, EO (follow up) | 1 (1%) |
| LOS, Maximal stability, EC (follow up) | 1 (1%) |
| LOS, Maximal stability, EO (follow up) | 1 (1%) |
| LOS, maximum excursion, affected side EO (follow-up) | 1 (1%) |
| LOS, maximum excursion, anterior, EO (follow-up) | 1 (1%) |
| LOS, maximum excursion, non-affected side EO (follow-up) | 1 (1%) |
| LOS, maximum excursion, posterior EO (follow-up) | 1 (1%) |
| LOS, Movement velocity, affected side EO (follow-up) | 1 (1%) |
| LOS, Movement velocity, anterior, EO (follow-up) | 1 (1%) |
| LOS, Movement velocity, non-affected side EO (follow-up) | 1 (1%) |
| LOS, Movement velocity, posterior EO (follow-up) | 1 (1%) |
| LOS, reaction time, affected side, EO (post-intervention) | 1 (1%) |
| LOS, reaction time, affected side, EO (follow-up) | 1 (1%) |
| LOS, reaction time, backward, EO (post-intervention) | 1 (1%) |
| LOS, reaction time, backward, EO (follow-up) | 1 (1%) |
| LOS, reaction time, forward, EO (post-intervention) | 1 (1%) |
| LOS, reaction time, forward, EO (follow-up) | 1 (1%) |
| LOS, reaction time, non-affected side, EO (post-intervention) | 1 (1%) |
| LOS, reaction time, non-affected side, EO (follow-up) | 1 (1%) |
| Maximum COP displacement in anteroposterior direction EC (post-intervention) | 1 (1%) |
| Maximum COP displacement in anteroposterior direction EC (follow-up) | 1 (1%) |
| Maximum COP displacement in anteroposterior direction EO (follow-up) | 1 (1%) |
| Maximum COP displacement in mediolateral direction EC (post-intervention) | 1 (1%) |
| Maximum COP displacement in mediolateral direction EC (follow-up) | 1 (1%) |
| Maximum COP displacement in mediolateral direction EO (follow-up) | 1 (1%) |
| Mediolateral sway length EC (post-intervention) | 1 (1%) |
| Stability index EC (post-intervention) | 1 (1%) |
| Sway area EC (follow-up) | 1 (1%) |
| Sway length EC (follow-up) | 1 (1%) |
| Sway length EO (follow-up) | 1 (1%) |
| Anteroposterior variability COP EC (post-intervention) | 1 (1%) |
| Mediolateral variability COP EC (post-intervention) | 1 (1%) |

Abbreviations: ADL, activities of daily living; BBS, berg balance scale; COP, center of pressure; EC, eyes closed; EO, eyes open; FIM, functional independence measure; IADL, instrumental activities of daily living; LOS, limit of stability; PASS, postural assessment scale for stroke; NT, no treatment; ST, sham treatment; UC, usual care; WB, weight bearing; WBA, weight bearing asymmetry

**S8B Table. Characteristics of outcome measures for each study included**

| Study | Outcomes | Post-intervention assessment | Follow-up assessment |
| --- | --- | --- | --- |
| Allison et Dennett, 2007 | RMA, TCT, BBS | 2w | 12 w |
| Askim et al., 2010 | BBS, MAS, BI, ST, 5mWT, SIS, Borg, time spent on home exercises, the training diaries, adverse events | 12w | 26 w |
| Barcala et al., 2011 | BBS, X and Y sway COP | 5w | No |
| Brogardh et al., 2012 | strength (knee), mAS (hip, knee, ankle), BBS, TUG, 6MWT, 10mWT, SIS | 6w | No |
| Büyükvural Şen et al., 2015 | FIM, SS-QOL, 10mWT, 6MWT, TUG, SCT, BBS, RMI, Strength (knee, ankle) | 3w | No |
| Cabanas-Valdés et al., 2015 | TIS, The Function in Sitting Test, BBA, BBS, PASS, BI, Tinetti | 5w | No |
| Chan KS et al., 2012 | mAS, H-reflex, Hmax/Max, deep tendon reflex of the Achilles tendon, VAS, TUG, 10mWT, BWD, pressure (foot area) | 21min | No |
| Chen CH et al., 2010 | gait, WBD (Symmetry index) | few min | No |
| Chen CL et al., 2015 | LOS (Movement velocity, Maximum excursion, Directional control), RWS | few min | No |
| Chen D et al., 2014 | FMA-LL, BBA, BBS, PASS, MBI | 3w | No |
| Chen IC et al., 2002 | Brunstrom, FIM, LOS (Maximal stability, ankle strategy, COG alignment, Axis velocity, directional control and end point excursion) | No | 6m + 2w |
| Chen JC et al., 2011 | FMA-LE, MRC-LE, mMAS, PASS-TC, BBS, FAC, mAS (hip, knee and ankle), AE | 6w | No |
| Chern et al., 2013 | COP total path excursion, maximum COP displacement, bilateral weight bearing difference, 10mWT and voluntary weight shifting | few min | No |
| Cho HY et al., 2013 | COP sway length, MAS, hand strength | 90min | 1d |
| Cho KH et al., 2012 | X and Y COP velocity, BBS, TUG | 6w | No |
| Cho MK et al., 2015 | gait (spatiotemporal parameters), 6MWT, BBS, strength, MMT | 4w | No |
| Chung et al., 2014 | gait (spatiotemporal parameters), strength, BBS | 6w | No |
| Dault et al., 2003 | COP sway, weight shifting | post-intervention | No |
| Duncan et al., 1998 | OPS, FMA, BI, IADL, MOS-36, 10mWT, 6MWT, BBS, JTHF | 12w | No |
| Duncan et al., 2003 | OPS, FMA, strength (Grip, ankle, knee extension), WMT, 10mWT, BBS, FR, 6MWT, EST, AE | 12 to 14w | No |
| Fritz et al., 2013 | FMA, BBS, DGI, 6MWT, 3mWT, SIS, TUG | 5w | 3m |
| Ghanjal et al., 2014 | TUG, 6MWT, BI, BBS | nd (after 12 sessions) | No |
| Globas et al., 2012 | peak V02, 6MWT, 10mW, 5CR, BBS, RMI, SF-12 | 3w | No |
| Hart et al., 2004 | Romberg test, EFAP, BBS, TUG, Standing on the unaffected leg, DHP | 12w | No |
| Heller et al., 2005 | FMA-LL, AS, PASS, FIM, FAC, gait | 9w | No |
| Howe et al., 2005 | LRT, STS test, COP sway | 4w | 8w |
| Hsu et al., 2013 | LE-STREAM, MOB-STREAM, FAC, BI, PASS , MAS | 8w | 12w |
| Hwang et al., 2015 | TUG, BBS, 10mWT, Muscle architecture | 4w | No |
| Immink et al., 2014 | 9HPT, MAS, BBS, 2MWD, CGS, GDS15, STAI, SIS | 10w | No |
| Janssen et al., 2008 | 6MWT, BBS, RMI, V02 max, POmax, MVC IL, MVC CL, HR | 6w | No |
| Jung et al., 2015 | TIS, mFRT, X and Y sway velocity | 4w | No |
| Kamps et Schule, 2005 | 10mWT, 2MWT, 6MWT, TUG, Tinetti, BBS | 16w | No |
| Katz-Leurer et al., 2006 | PASS, FMA-LL, FIM, MAS, Standing Balance test | 3w | 6w |
| Kim DH et al., 2008 | strength, TUG, BBS, 10mWT, step-up | 4w | No |
| Kim JH et al., 2009 | BBS, 10mWT, mMAS, gait (spatiotemporal parameters), mean balance, COP sway (area, path, maximal sway velocity, X and Y sway angle) | 4w | No |
| Kim YH et al., 2004 | COP sway | 3w | No |
| Kim YM et al., 2009 | PASS, BBS, TCT, mBI, MI | 3w | No |
| Kunkel et al., 2013 | WBD, max weight transferred, 10mWT, BBS, RMI, acceptability | 2w | 4w |
| Langhammer et al., 2009 | IADL, MAS, 6MWT, BBS, TUG, strength, AS, heart rate, interview | 12m | 4y |
| Lau RWK et al., 2012 | BBS, LOS (MVL, EPE, MXE, DCL), 10mWT, 6MWT, strength, ABC, falls | 8w | 1m + 8w |
| Laufer, 2003 | Sway Index, BWD | few min | No |
| Lee CH et al., 2014 | TUG, BBS, gait (spatiotemporal parameters) | 4w | No |
| Lee NK et al., 2013 | MVIC, Y and X sway velocities | 6w + 1d | No |
| Lee SH et al., 2012 | FAC, 10mWT, TUG, BBS, mBI, MMT | 4w | No |
| Lee SW et al., 2013 | COP sway (velocity and length) | 6w | No |
| Liang et al., 2012 | FMA-LE, MRC-LE, FAC, BBS, mMAS, BI | 6w | 12m |
| Lindvall et Forsberg, 2014 | TUG, TUG Cognitive Test, 6MWT, BBS, Timed-stands Test, ABC, SF-36 | 9w | 14w |
| Lisinski et al., 2012 | WBD, COP sway (X and Y velocity), Brunnström scale | 20d | No |
| Liu-Ambrose et Eng, 2015 | Stroop Test, Trail Making Tests, verbal digits forward and backward tests, GDS, 6MWT, BBS | 6m | No |
| Lu et al., 1997 | COP sway (maximum sway, total travel distance, mean travel speed of the COP), gait | post-intervention | No |
| Lynch et al., 2007 | Light touch (wmes-Weinstein monofilaments), DPT, BBS, 10mWT, 14mWT, ILAS | 2w | 4w |
| Marin et al., 2013 | muscle architecture, strength (knee), BBS | 12w | No |
| Merkert et al., 2011 | BBS, BI, F-test, Tinetti, TUG | 2w | No |
| Milczarek et al., 1993 | COP sway travel, X and Y COP position | post-intervention | No |
| Mojica et al., 1988 | body sway, X and Y COP position, gait (spatiotemporal parameters) | 15min | No |
| Moore JL et al., 2010 | gait speed, 12MWT, 02 cost (gait), Peak treadmill velocity, peak VO2, BBS, TUG, number of step | 4w | No |
| Morioka et Yagi, 2003 | COP sway (total locus length, enveloped area, and rectangular area) | 2w | No |
| Mudie et al., 2002 | WBD, BI | 2w | 14w |
| Nadeau et al., 2013 | gait (speed), 6MWT, step activity, FMA, BBS, ABC, SIS, falls, AE | 4m | No |
| Park et al., 2014 | mAS (ankle), COP sway velocity and speed moment, TUG, gait (spatiotemporal parameters) | 6w | No |
| Pollock et al., 2002 | SI, maximum weight transference | 4w | 6w |
| Pomeroy et al., 2001 | BBS, gait (spatiotemporal parameters), patient experience | 6w | No |
| Rajaratnam et al., 2013 | FRT, BBS, TUG, COP sway | 60min | No |
| Robertson et al., 2010 | ABC, BBS, TUG, 10mWT, gait speed, toe height (foot clearance) | few min | No |
| Rougier et Boudrahem, 2010 | COP sway (area, velocity, variance, DC, mean time interval, mean square distances), X and Y COP position | post-intervention | No |
| Schmid et al., 2012 | mRS, BBS, ABC, FoF, SS-QoL | 8w | No |
| Schuster et al., 2012 | motor task (time), CMSA, Stage of the motor task (Bergland and Lake), Imagination inflation (time), EBI, BBS, KVIQ, Imaprax software, ABC, diary (motivation), 11-point VAS | 2w | 4w |
| Shatil et al., 2005 | golf performance, COP excursion velocity, peak arm acceleration, EMG, BBS, CMPCI, SIP | 6w | No |
| Simons et al., 2009 | BBS, TUG, TBT, 10mWT, FAC, WBD (static and dynamic), DBC | few min | No |
| Song et al., 2014 | BBS, FI, SI, WDI | 3w | No |
| Suh et al., 2014 | mAS, FRT, BBS, TUG, 10mWT | 2h 30min | No |
| Tan et al., 2014 | FMA-LL, PASS, BBS, BI, FAC | 3w | 3m + 3w |
| Tian et al., 2014 | track length, peripheral square, track length of per unit square, left-right offset and rectangle square, Mean-X, Mean-Y, Max-X, Max-Y, LSKG, SSKG, LFS, SI, WDI | 4w | No |
| Tilikete et al., 2001 | X and Y COP position, COP sway area | 3min | No |
| Tripp and Krakow, 2014 | BBS, FRT, FAC, RMI | 2w | No |
| Tung et al., 2010 | BBS, WBD, LOS (MXE, DCL), strength | 4w | No |
| VanNes et al., 2006 | BBS, BI, TCT, RMI, FAC, MI, Somatosensory threshold, AE | 6w | 12w |
| Waldron et Bohannon, 1989 | WBD | 15min | No |
| Wang RY, Lin PY et al., 2007 | gait (spatiotemporal parameters), WBD, LOS (MXE, MVL) | few min | No |
| Wang RY, Yen LL et al., 2005 part 1 | WBD, COP sway, LOS (MVL, DCL, MXE), sit-to-stand test (rising time, weight transfer and COP sway), BBS, 10mWT | 15min | No |
| Wang RY, Yen LL et al., 2005 part 2 | WBD, COP sway, LOS (MVL, DCL, MXE), sit-to-stand test (rising time, weight transfer and COP sway), BBS, 10mWT | 15min | No |
| Wang TC et al., 2015 | BBS, 10mWT, 6MWT, SIS, BI, caregiver burden test | 12w | No |
| Xing et al., 2007 | BBS, Brunnstrom | 2 to 3w | No |
| Yoo et al., 2010 | BBS, TIS, TCT (K-MMSE et K-MBI for experimental group only) | 4w | No |
| You et al., 2014 | CSS, FMA-LE, PASS, BBS, mBI | 3w | No |
| Zhang et al., 2015 | mBI, FMA, BBS | 6w | 12w |
| Au-Yeung et al., 2009 | LOS (reaction time and EPE), SOT, TUG | 12w | 18w |
| Furnari et al., 2014 | length of the ball (COP), index of energy expenditure, plantar load for each foot, Plantar Surface for each foot, gait (spatiotemporal parameters) | 8w | No |
| Geiger et al., 2001 | TUG, BBS | 4w | No |
| Hollands et al., 2015 | gait (spatiotemporal parameters), time to turn 180°, adaptability of gait (success rate in target stepping), FMA-LE, SF-12, TUG, FAC, Falls Efficacy Scale, BBS | 8w | 12w |
| Holmgren et al., 2010 | BBS, BI, FES-I, number of falls, FAI-3, Borg scale | 5w | 3m |
| Hosseini et al., 2012 | TUG, BBS | nd | nd |
| Kilinc et al., 2015 | BBS, STREAM, TIS, FRT, 10mWT, TUG | 12w | No |
| Noh et al., 2008 | BBS , WBA, strength, mMAS | No | 12w |
| Page et al., 2008 | FMA-LL, BBS | 8w | No |
| Stein et al., 2014 | TUG, BBS, 10mWT, 6MWT, Five-Times-Sit-to-Stand test, EFAP, California Functional Evaluation 40 | 6w | 19w |
| In et al., 2016 | BBS, FRT, TUG, posturography (medial-lateral distance, anterior-posterior distance, and total sway distance), 10mWT | 4w | No |
| Kwong et al., 2018 | ankle and knee muscle strength, BBS, TUG, Step Test and the Lower Extremity Motor Coordination Test | 10w | 3m + 10w |
| Arabzadeh et al., 2018 | BBS, the postural sway parameters, including COP path length and COP area, and WBA | 4w | No |
| Kim JY et al., 2018 | FAC, Motricity index (lower limb subscale), 10mWT, mBI, RMI, BBS | 3w | No |
| Erbil et al., 2018 | TUG, BBS, mAS, Tardieu Scale, Rivermead Visual Gait Assessment | No | 12w |
| Park HK et al., 2018 | TIS, PASS-3L (5 items), BBS-3L (7 items), FRT, mBI | 4w | No |
| Xie et al., 2018 | BBS, Single leg stance test (SLST), FMA, mBI, 36-Item Short-Form health survey, Beck depression inventory, Modified falls efficacy scale, TUG | 12w | 24w |
| Choi HS et al., 2017 | WBA, the deviation of the COP (the medial-lateral axis distance, and anteroposterior axis distance, sway mean velocity, sway area), FRT, mFRT, TUG | 4w | No |
| Lee D et al., 2016 | strength of the lower extremity, mAS, BBS, TUG, 6MWT | 4w | No |
| Fernandez-Gonzalo et al., 2016 | maximal dynamic and isometric force and power tests, and m. quadriceps femoris cross sectional area and volume (MRI), BBS, TUG, Dual-task cost on walking, mAS | 3m | No |
| Dujovic et al., 2017 | 10mWT (gait speed), FMA, BBS, BI | 4w | No |
| Yeung et al., 2018 | FAC, FMA, MAS, BBS, 10mWT, 6MWT, gait analysis | 5w | 10w |
| Yun et al., 2018 | BLS, BBS, PASS, FMA, K-mBI, Somatosensory Evoked potentials | 3w | 7w |
| Han et al., 2016 | brachial-ankle Pulse wave velocity, cardiopulmonary fitness (VO2, respiratory exchange ratio, the peak values of each of these parameters and peak heart rate, instantaneous systolic blood pressure (SBP) and diastolic blood pressure (DBP). Resting and peak SBP and DBP values, resting and peak heart rate values, peak RER, and duration of exercise treadmill test, K-mBI, BBS, FMA-LL, FAC | 4w | No |
| Vahlberg et al., 2017 | BBS, Short Physical Performance Battery (balance, gait speed and the ability to rise from a seated position five times), 6MWT, 10mWT, PASE, EQ-5D, Geriatric Depression Scale-20, Fall-related self-efficacy | 12w | 15m |
| Knox et al., 2018 | BBS, 6mWT, 10mWT (comfortable and fast speed), TUG | 12w | 24w |
| Goliwas et al., 2017 | SI, BBS, FMA-LL, mAS, Exteroceptive sensation | 6w | No |
| Shin et al., 2016 | mFRT, TIS, TUG, posturography (ML and AP sway speed, velocity moment) | 4w | No |
| Kim SL et Lee, 2018 | weight‐bearing lunge test, COP sway path length, COP sway speed, TUG, DGI | 4w | No |
| Khumsapsiri et al., 2018 | LOS (MMVL, EPE, MXE), WBA, FAB | 4w | 8w |
| Yu et Cho, 2016 | BBS, TUG, mBI | 6w | No |
| Sohn et al., 2015 | overall index, AP index, ML index, BBS, TIS | 15min | No |
| Hsieh, 2019 | 10mWT, maximum CoP sway in the AP direction, maximum CoP sway in the ML direction, sway area | 10w | No |
| Ferreira et al., 2017 | AP COP, ML COP | 12w | No |
| Kim JC et Lee, 2018 | WDI, LOS (LOS-surface area, LOS-area ratio), TUG, DGI | 3w | No |
| Park D et al., 2018 | Static balance ability, TUG, gait (spatiotemporal parameters) | 15min | No |
| Buyukavci et al., 2016 | Brunnstrom, TIS, BBS, FIM, RMI | No | 3m |
| Hung et al., 2016 | Adherence, safety, and satisfaction, TUG, FRT, Physiologic Profile Assessment Subtests, WBA | 6w | No |
| Lee MM et al., 2018 | COP path length, COP sway velocity, mFRT, MFT | 5w | No |
| Yoo et al., 2018 | pulmonary function (FVC, FEV1, Peak flow), NIHSS, mBI, BBS, FMA, K-MMSE, incidence of pneumonia | 3w | No |
| Karasu et al., 2018 | BBS, FRT, PASS, TUG, SBI, Postural sway, FIM (locomotion and transfert) | 4w | 8w |
| Bunketorp-Kall et al., 2017 | SIS, TUG, BBS, the Bäckstrand, Dahlberg and Liljenäs Balance Scale, Hand strength, Barrow Neurological Institute screen, working memory (letter–number sequencing test) | 12w | 6m |
| Wang et al., 2017 | Brunnstrom staging of motor function of lower extremity, BBS, FAC, FIM (transporting as well as walking part), BOLD-fMRI scan | 6w | No |
| Park DS et al., 2017 | FMA-LL, BBS, TUG, 10mWT | 6w | No |
| Jung et al., 2017 | Postural sway distance, strength (hip, knee and ankle), Composite Spasticity Score | 6w | No |
| Ordahan et al., 2015 | BBS, TUG, FIM | 6w | No |
| Nikamp et al., 2017 | 10mWT, BBS, 6MWT, TUG, stairs test, FAC, RMI, BI | 9w | No |
| Chen, 2018 | Body composition, mood state scale, flexibility, muscle endurance, and maximum oxygen uptake, BBS, single-legged standing ability test, motor evoked potentials, EEG | 6w | No |
| Sanchez-Mila et al., 2018 | mAS, FMA, LOS (MVL, MXE, EPE, DCL), equitest | 60min | No |
| Ng et al., 2016 | BBS, 6MWT, RMI, TUG, SF-36 | 8w | 8w + 3m |
| Bae et al., 2015 | BBS, COP sway area, LOS | few min | No |
| Lee HJ et al., 2018 | 10mWT, 6MWT, TUG, BBS | 6w | No |
| Park J et al., 2017 | MFT, strength, BBS, pCOP velocity moment, COP AP sway, COP ML sway, 10mWT, SS-QOL | 6w | No |
| Yadav et al., 2015 | BBS, TUG | 2w | No |
| Huh et al., 2015 | BBS, FAC, 6MWT, TUG, K-mBI, MMT | 2w | No |
| Lin Q et al., 2015 | FMA, BBS, BI | 4w | No |
| Salgueiro et Marquez, 2018 | BBS, TUG, COP ML displacement, COP AP displacement, COP sway velocity, X COP, Y COP | 3w | No |
| Chu et al., 2015 | BBS, Sheikh Trunk Control Scale, FMA-LL, Holden FAC, 10mWT | 8w | No |
| Tan et al., 2016 | FMA-LL, 10mWT, BBS, mBI, EMG | No | 6w |

Abbreviations: 10mWT, 10-meter walking Test; 2 MWT, 2 minutes walking test; 2MWD, two-minute walking distance; 3mWT, 3-meter walking test; 5CR, the 5-chair-rise test; 5mTW, the 5-meter timed walk; 5mWT, 5-meter walking test; 6MWT, 6 minutes walking test; 9HPT, 9-hole peg test; ABC, activities-specific balance confidence scale; AE, adverse effects; AP, anteroposterior; AS, ashworth scale; BBA, brunel balance assessment; BBS, berg balance scale; BC, balance coefficient; BI, barthel index; BWD, body weight distribution; CGS, comfortable gait speed; CMPCI, chedoke-McMaster postural control inventory; d, day; DBC, dynamic balance contribution; DC, dependency coefficient; DCL, directional control; DGI, dynamic gait index; DHP, duke health profile; DPT, the distal proprioception test; EBI, extended barthel index; EFAP, emory functional ambulation profile; EMG, electromyogram; EPE, end point excursions; ES, electrostimulation; EST, exercise stress test; FAC, functional ambulation category; FI, falling index; FIM, functional Independence measure; FMA-LE, lower limb part of fugl-meyer assessment; FoF, fear or fall; FR, functional reach; GDS15, geriatric depression scale 15-item; H, hour; IADL, instrumental activity of daily living; ILAS, iowa level of assistance scale; JTHF, jebsen test of hand function; LE-STREAM, lower extremity part of stroke rehabilitation assessment of movement measure; LFS, square ratio of weight shift; LOS, limit of stability; LSKG, weight shift distance; M, month; MAS, motor assessment scale; mAS, modified ashworth scale; Max, maximum; Max-X, maximum of X axis weight shift distance; Max-Y, maximum of Y axis weight shift distance; mBI, modified barthel index; Mean-X, mean of X axis weight shift distance; Mean-Y, mean of Y axis weight shift distance; MEP, motor evoked potentials; mFRT, modified functional reach test; MFT, manual function test; MI, Motricity index; MI-LE, lower limb part of motricity Index; Min, minute; ML, mediolateral; mMAS, modified motor assessment scale; MMT, manual muscle test; MOB-STREAM, the mobility subscale of the stroke rehabilitation assessment of movement measure; MOS-36, 36-item short-form health survey (SF-36); MRC-LE, the medical research council scale for lower extremity; MRI, magnetic resonance imaging; MVC CL, isometric maximal voluntary contractions of contralateral leg; MVC IL, isometric maximal voluntary contractions of ipsilateral leg; MVIC, maximum voluntary isometric contraction; MVL, movement velocity; MXE, maximum excursion; NRS, numerical rating scales; Num, number; OPS, orpington prognostic scale; PASS, postural assessment scale for stroke; PASS-TC, items on trunk control of postural assessment scale for stroke; POMA, tinetti performance oriented mobility assessment; RMA, rivermead motor assessment; RMI, rivermead mobility index; RWS, rhythmic weight shift; SC, sway coefficient; SCT, stair-climbing test; SF-12, the 12-item short form health survey; SI, symmetry index; SIP, sickness impact profile; SIS, stroke impact scale; SIS-16, 16 items of stroke impact scale; SS-QOL, stroke specific quality of life scale; SSKG, weight shift square; ST, step test; STAI, state trait anxiety inventory; TCT, trunk control test; TIS, trunk impairment scale; TUG, time up and go test; UC, usual Care; VAS, visual analogue scale; W, week; WDI, weight distribution index; WMT, wolf motor function; X, mediolateral; Y, anteroposterior
