# Supplementary material for: Limited evidence of physical therapy on balance after stroke: A systematic review and meta-analysis
Source: PLoS One. 2019 Aug 29;14(8):e0221700. doi: 10.1371/journal.pone.0221700 (PMC6715189; doi:10.1371/journal.pone.0221700)
Supplement: S9 Table — (DOCX) [file pone.0221700.s023.docx]

**S9 Table. Results of subgroup analyses according to the time since post-stroke**

| Outcome or Subgroup | Studies, No. | Participants, No. | Statistical Method | Effect Estimate  SMD (95% CI) | Heterogeneity I^2^ & test for subgroup differences Chi^2^ |  |
| --- | --- | --- | --- | --- | --- | --- |
| 1. PT versus no treatment, post-intervention effect | | | | | | |
| 1.1 Balance | 37 | 1721 | Fixed | 0.46 [0.37; 0.56] | 19.1%  SgD: Chi2 p=0.50 |  |
| 1.1.1 Acute and subacute stroke | 15 | 1032 | Fixed | 0.45 [0.33; 0.58] | 44.1% |  |
| 1.1.2 Chronic stroke | 16 | 464 | Fixed | 0.54 [0.35; 0.74] | 0% |  |
| 1.1.3 Mixed or not determined | 6 | 225 | Fixed | 0.35 [0.09; 0.62] | 0% |  |
| 1.2 Mediolateral postural deviation, EO | 11 | 430 | Fixed | -0.23 [-0.36; -0.09] | 0%  SgD: Chi2 p=0.34 |  |
| 1.2.1 Acute and subacute stroke | 3 | 120 | Fixed | -0.39 [-0.66; -0.13] | 0% |  |
| 1.2.2 Chronic stroke | 1 | 61 | Fixed | -0.13 [-0.49; 0.22] | NA |  |
| 1.2.3 Mixed or not determined | 7 | 249 | Fixed | -0.17 [-0.35; 0.00] | 2.6% |  |
| 1.3 Postural stability, EO | 16 | 504 | Random | 0.48 [0.25; 0.70] | 29.3%  SgD: Chi2 p=1.00 |  |
| 1.3.1 Acute and subacute stroke | 4 | 186 | Random | 0.50 [0.21; 0.80] | 0% |  |
| 1.3.2 Chronic stroke | 6 | 139 | Random | 0.48 [-0.09; 1.05] | 60.3% |  |
| 1.3.3 Mixed or not determined | 6 | 179 | Random | 0.50 [0.14; 0.86] | 26% |  |
| 1.4 Autonomy | 15 | 941 | Fixed | 0.36 [0.23; 0.49] | 0%  SgD: Chi2 p=0.68 |  |
| 1.4.1 Acute and subacute stroke | 9 | 754 | Fixed | 0.33 [0.19; 0.48] | 0% |  |
| 1.4.2 Chronic stroke | 3 | 89 | Fixed | 0.43 [0.01; 0.86] | 41.6% |  |
| 1.4.3 Mixed or not determined | 3 | 98 | Fixed | 0.50 [0.09; 0.90] | 0% |  |
| 2. PT versus no treatment, persisting effect | | | | | | |
| 2.1 Balance | 11 | 493 | Random | 0.29 [-0.02; 0.59] | 60.2%  SgD: Chi2 p=0.64 |  |
| 2.1.1 Acute and subacute stroke | 6 | 295 | Random | 0.16 [-0.07; 0.40] | 71.4% |  |
| 2.1.2 Chronic stroke | 3 | 141 | Random | 0.44 [0.11; 0.78] | 0% |  |
| 2.1.3 Mixed or not determined | 2 | 57 | Random | 0.38 [-0.16; 0.92] | 73.0% |  |
| 2.2 Mediolateral postural deviation, EO | 3 | 50 | Fixed | -0.44 [-1.05; 0.16] | 0%  SgD: Chi2 p=0.47 |  |
| 2.2.1 Acute and subacute stroke | 2 | 36 | Fixed | -0.28 [-1.03; 0.47] | 0% |  |
| 2.2.2 Chronic stroke | 0 | 0 | Fixed | Not estimable | NA |  |
| 2.2.3 Mixed or not determined | 1 | 16 | Fixed | -0.75 [-1.77; 0.28] | NA |  |
| 2.3 Postural stability, EO | 3 | 80 | Fixed | 0.31 [-0.14; 0.76] | 12.1%  SgD: Chi2 NA |  |
| 2.3.1 Acute and subacute stroke | 0 | 0 | Fixed | Not estimable | NA |  |
| 2.3.2 Chronic stroke | 0 | 0 | Fixed | Not estimable | NA |  |
| 2.3.3 Mixed or not determined | 3 | 80 | Fixed | 0.31 [-0.14; 0.76] | 12.1% |  |
| 2.4 Autonomy | 6 | 312 | Fixed | 0.36 [0.13; 0.58] | 0%  SgD: Chi2 p=0.97 |  |
| 2.4.1 Acute and subacute stroke | 5 | 278 | Fixed | 0.36 [0.12; 0.59] | 0% |  |
| 2.4.2 Chronic stroke | 0 | 0 | Fixed | Not estimable | NA |  |
| 2.4.3 Mixed or not determined | 1 | 34 | Fixed | 0.37 [-0.31; 1.05] | NA |  |
| 3. PT versus sham treatment/usual care, post-intervention effect | | | | | | |
| 3.1 Balance | 46 | 2051 | Random | 0.43 [0.28; 0.59] | 60.9%  SgD: Chi2 p=0.16 |  |
| 3.1.1 Acute and subacute stroke | 15 | 753 | Random | 0.36 [0.14; 0.58] | 50% |  |
| 3.1.2 Chronic stroke | 21 | 793 | Random | 0.60 [0.35; 0.86] | 64.5% |  |
| 3.1.3 Mixed or not determined | 10 | 505 | Random | 0.22 [-0.10; 0.55] | 57.4% |  |
| 3.2 Mediolateral postural deviation, EO | 4 | 122 | Fixed | -0.15 [-0.52; 0.21] | 38.2%  SgD: Chi2 p=0.11 |  |
| 3.2.1 Acute and subacute stroke | 1 | 15 | Fixed | 0.13 [-0.97; 1.23] | 0% |  |
| 3.2.2 Chronic stroke | 2 | 67 | Fixed | -0.50 [-0.99; -0.01] | 36.9% |  |
| 3.2.3 Mixed or not determined | 1 | 40 | Fixed | 0.32 [-0.30; 0.94] | NA |  |
| 3.3 Postural stability, EO | 15 | 574 | Random | 0.96 [0.55; 1.37] | 77.9%  SgD: Chi2 p=0.09 |  |
| 3.3.1 Acute and subacute stroke | 3 | 65 | Random | 0.24 [-0.26; 0.74] | 0% |  |
| 3.3.2 Chronic stroke | 10 | 429 | Random | 0.83 [0.49; 1.17] | 58.2% |  |
| 3.3.3 Mixed or not determined | 2 | 80 | Random | 3.34 [-1.72; 8.40] | 97.4% |  |
| 3.4 Autonomy | 15 | 805 | Random | 0.26 [0.01; 0.51] | 61.1%  SgD: Chi2 p=0.02 |  |
| 3.4.1 Acute and subacute stroke | 9 | 360 | Random | 0.24 [0.02; 0.45] | 75% |  |
| 3.4.2 Chronic stroke | 1 | 51 | Random | 0.74 [0.17; 1.30] | NA |  |
| 3.4.3 Mixed or not determined | 5 | 394 | Random | -0.02 [-0.22; 0.18] | 36% |  |
| 4. PT versus sham treatment/usual care, persisting effect | | | | | | |
| 4.1 Balance | 18 | 1150 | Fixed | 0.18 [0.06; 0.30] | 48.8%  SgD: Chi2 p=0.23 |  |
| 4.1.1 Acute and subacute stroke | 7 | 407 | Fixed | 0.24 [0.04; 0.44] | 40% |  |
| 4.1.2 Chronic stroke | 6 | 367 | Fixed | 0.27 [0.05; 0.48] | 51% |  |
| 4.1.3 Mixed or not determined | 5 | 376 | Fixed | 0.03 [-0.18; 0.24] | 59% |  |
| 4.2 Postural stability, EO | 2 | 178 | Fixed | 0.32 [0.02; 0.62] | 0%  SgD: Chi2 NA |  |
| 4.2.1 Acute and subacute stroke | 0 | 0 | Fixed | Not estimable | NA |  |
| 4.2.2 Chronic stroke | 2 | 178 | Fixed | 0.32 [0.02; 0.62] | 0% |  |
| 4.2.3 Mixed or not determined | 0 | 0 | Fixed | Not estimable | NA |  |
| 4.3 Autonomy | 9 | 551 | Fixed | -0.00 [-0.17; 0.17] | 26%  SgD: Chi2 p=0.36 |  |
| 4.3.1 Acute and subacute stroke | 5 | 187 | Fixed | 0.11 [-0.18; 0.39] | 0% |  |
| 4.3.2 Chronic stroke | 0 | 0 | Fixed | Not estimable | NA |  |
| 4.3.3 Mixed or not determined | 4 | 364 | Fixed | -0.06 [-0.27; 0.15] | 49.5% |  |

Legend:

Autonomy: combination of barthel index, functional independence measure, activities of daily living and instrumental activities of daily living scales.

Mediolateral postural deviation: combination of weight bearing asymmetry and mediolateral position of center of pressure

Abbreviations: ADL, activities of daily living; BBS, berg balance scale; CI, confidence interval; COP, center of pressure; CPI, cardiopulmonary intervention; EO, eyes open; EC, eyes closed; IADL, instrumental activities of daily living; FIM, functional independence measure; FTT, functional task training; MD, mean difference; MS, muscle strengthening; MM, musculoskeletal mobilization; NA, not applicable; NPI, neurophysiological intervention; PASS, postural assessment scale for stroke; PT, physical therapy; RCT, randomized controlled trials; SgD, subgroup difference; SMD, standardized mean difference; X, mediolateral position of COP; Y, anteroposterior position of COP; WB, weight bearing.
