# Supplementary material for: Limited evidence of physical therapy on balance after stroke: A systematic review and meta-analysis
Source: PLoS One. 2019 Aug 29;14(8):e0221700. doi: 10.1371/journal.pone.0221700 (PMC6715189; doi:10.1371/journal.pone.0221700)
Supplement: S10 Table — (DOCX) [file pone.0221700.s024.docx]

**S10 Table. Results of subgroup analyses according to the location of stroke lesion**

| Outcome or Subgroup | Studies, No. | Participants, No. | Statistical Method | Effect Estimate  SMD (95% CI) | Heterogeneity I^2^  & test for subgroup differences Chi^2^ |  |
| --- | --- | --- | --- | --- | --- | --- |
| 1. PT versus no treatment, post-intervention effect | | | | | | |
| 1.1 Balance | 37 | 1721 | Fixed | 0.46 [0.37; 0.56] | 19.1%  SgD: Chi2 p=0.71 |  |
| 1.1.1 Supratentorial stroke | 3 | 114 | Fixed | 0.53 [0.16; 0.91] | 0% |  |
| 1.1.2 Brainstem stroke | 0 | 0 | Fixed | Not estimable | NA |  |
| 1.1.3 Cerebellum stroke | 0 | 0 | Fixed | Not estimable | NA |  |
| 1.1.4 Mixed or not determined | 34 | 1607 | Fixed | 0.46 [0.36; 0.56] | 23.2% |  |
| 1.2 Mediolateral postural deviation, EO | 11 | 430 | Fixed | -0.23 [-0.36; -0.09] | 0%  SgD: Chi2 p=0.08 |  |
| 1.2.1 Supratentorial stroke | 2 | 80 | Fixed | 0.02 [-0.29; 0.33] | 0% |  |
| 1.2.2 Brainstem stroke | 0 | 0 | Fixed | Not estimable | NA |  |
| 1.2.3 Cerebellum stroke | 0 | 0 | Fixed | Not estimable | NA |  |
| 1.2.4 Mixed or not determined | 9 | 350 | Fixed | -0.28 [-0.44; -0.13] | 0% |  |
| 1.3 Postural stability, EO | 16 | 504 | Fixed | 0.47 [0.29; 0.65] | 29.3%  SgD: Chi2 p=0.96 |  |
| 1.3.1 Supratentorial stroke | 2 | 40 | Fixed | 0.49 [-0.15; 1.12] | 0.9% |  |
| 1.3.2 Brainstem stroke | 0 | 0 | Fixed | Not estimable | NA |  |
| 1.3.3 Cerebellum stroke | 0 | 0 | Fixed | Not estimable | NA |  |
| 1.3.4 Mixed or not determined | 14 | 464 | Fixed | 0.47 [0.28; 0.66] | 34.9% |  |
| 1.4 Autonomy | 15 | 941 | Fixed | 0.36 [0.23; 0.49] | 0%  SgD: Chi2 p=0.62 |  |
| 1.4.1 Supratentorial stroke | 2 | 64 | Fixed | 0.24 [-0.25; 0.73] | 0% |  |
| 1.4.2 Brainstem stroke | 0 | 0 | Fixed | Not estimable | NA |  |
| 1.4.3 Cerebellum stroke | 0 | 0 | Fixed | Not estimable | NA |  |
| 1.4.4 Mixed or not determined | 13 | 877 | Fixed | 0.37 [0.23; 0.51] | 0% |  |
| 2. PT versus no treatment, persisting effect | | | | | | |
| 2.1 Balance | 11 | 493 | Random | 0.29 [-0.02; 0.59] | 60.2%  SgD: Chi2 NA |  |
| 2.1.1 Supratentorial stroke | 0 | 0 | Random | Not estimable | NA |  |
| 2.1.2 Brainstem stroke | 0 | 0 | Random | Not estimable | NA |  |
| 2.1.3 Cerebellum stroke | 0 | 0 | Random | Not estimable | NA |  |
| 2.1.4 Mixed or not determined | 11 | 493 | Random | 0.29 [-0.02; 0.59] | 60.2% |  |
| 2.2 Mediolateral postural deviation, EO | 3 | 50 | Fixed | -0.44 [-1.05; 0.16] | 0%  SgD: Chi2 p=0.47 |  |
| 2.2.1 Supratentorial stroke | 1 | 16 | Fixed | -0.75 [-1.77; 0.28] | NA |  |
| 2.2.2 Brainstem stroke | 0 | 0 | Fixed | Not estimable | NA |  |
| 2.2.3 Cerebellum stroke | 0 | 0 | Fixed | Not estimable | NA |  |
| 2.2.4 Mixed or not determined | 1 | 34 | Fixed | -0.28 [-1.03; 0.47] | 0% |  |
| 2.3 Postural stability, EO | 3 | 80 | Fixed | 0.31 [-0.14; 0.76] | 12.1%  SgD: Chi2 p=0.14 |  |
| 2.3.1 Supratentorial stroke | 2 | 57 | Fixed | 0.11 [-0.42; 0.63] | 0% |  |
| 2.3.2 Brainstem stroke | 0 | 0 | Fixed | Not estimable | NA |  |
| 2.3.3 Cerebellum stroke | 0 | 0 | Fixed | Not estimable | NA |  |
| 2.3.4 Mixed or not determined | 1 | 23 | Fixed | 0.87 [0.01; 1.74] | NA |  |
| 2.4 Autonomy | 6 | 312 | Fixed | 0.36 [0.13; 0.58] | 0%  SgD: Chi2 NA |  |
| 2.4.1 Supratentorial stroke | 0 | 0 | Fixed | Not estimable | NA |  |
| 2.4.2 Brainstem stroke | 0 | 0 | Fixed | Not estimable | NA |  |
| 2.4.3 Cerebellum stroke | 0 | 0 | Fixed | Not estimable | NA |  |
| 2.4.4 Mixed or not determined | 6 | 312 | Fixed | 0.36 [0.13; 0.58] | 0% |  |
| 3. PT versus sham treatment/usual care, post-intervention effect | | | | | | |
| 3.1 Balance | 46 | 2051 | Random | 0.43 [0.28; 0.59] | 60.9%  SgD: Chi2 p=0.27 |  |
| 3.1.1 Supratentorial stroke | 7 | 318 | Random | 0.26 [-0.06; 0.58] | 49.5% |  |
| 3.1.2 Brainstem stroke | 0 | 0 | Random | Not estimable | NA |  |
| 3.1.3 Cerebellum stroke | 0 | 0 | Random | Not estimable | NA |  |
| 3.1.4 Mixed or not determined | 39 | 1733 | Random | 0.47 [0.30; 0.64] | 62.6% |  |
| 3.2 Mediolateral postural deviation, EO | 4 | 122 | Fixed | -0.15 [-0.52; 0.21] | 38.2%  SgD: Chi2 p=0.59 |  |
| 3.2.1 Supratentorial stroke | 1 | 15 | Fixed | 0.13 [-0.97; 1.23] | 0% |  |
| 3.2.2 Brainstem stroke | 0 | 0 | Fixed | Not estimable | NA |  |
| 3.2.3 Cerebellum stroke | 0 | 0 | Fixed | Not estimable | NA |  |
| 3.2.4 Mixed or not determined | 3 | 107 | Fixed | -0.19 [-0.57; 0.20] | 64.9% |  |
| 3.3 Postural stability, EO | 15 | 574 | Random | 0.96 [0.55; 1.37] | 77.9%  SgD: Chi2 p=0.46 |  |
| 3.3.1 Supratentorial stroke | 1 | 15 | Random | 0.54 [-0.58; 1.66] | 0% |  |
| 3.3.2 Brainstem stroke | 0 | 0 | Random | Not estimable | NA |  |
| 3.3.3 Cerebellum stroke | 0 | 0 | Random | Not estimable | NA |  |
| 3.3.4 Mixed or not determined | 14 | 559 | Random | 1.00 [0.56; 1.43] | 80.5% |  |
| 3.4 Autonomy | 15 | 805 | Random | 0.26 [0.01; 0.51] | 61.1%  SgD: Chi2 p=0.62 |  |
| 3.4.1 Supratentorial stroke | 6 | 278 | Random | 0.19 [-0.13; 0.51] | 43.8% |  |
| 3.4.2 Brainstem stroke | 0 | 0 | Random | Not estimable | NA |  |
| 3.4.3 Cerebellum stroke | 0 | 0 | Random | Not estimable | NA |  |
| 3.4.4 Mixed or not determined | 9 | 527 | Random | 0.32 [-0.05; 0.69] | 68.9% |  |
| 4. PT versus sham treatment/usual care, persisting effect | | | | | | |
| 4.1 Balance | 18 | 1150 | Fixed | 0.18 [0.06; 0.30] | 48.8%  SgD: Chi2 p=0.36 |  |
| 4.1.1 Supratentorial stroke | 3 | 121 | Fixed | 0.02 [-0.34; 0.38] | 3% |  |
| 4.1.2 Brainstem stroke | 0 | 0 | Fixed | Not estimable | NA |  |
| 4.1.3 Cerebellum stroke | 0 | 0 | Fixed | Not estimable | NA |  |
| 4.1.4 Mixed or not determined | 15 | 1029 | Fixed | 0.20 [0.07; 0.33] | 53% |  |
| 4.2 Postural stability, EO | 2 | 178 | Fixed | 0.32 [0.02; 0.62] | 0%  SgD: Chi2 NA |  |
| 4.2.1 Supratentorial stroke | 0 | 0 | Fixed | Not estimable | NA |  |
| 4.2.2 Brainstem stroke | 0 | 0 | Fixed | Not estimable | NA |  |
| 4.2.3 Cerebellum stroke | 0 | 0 | Fixed | Not estimable | NA |  |
| 4.2.4 Mixed or not determined | 2 | 178 | Fixed | 0.32 [0.02; 0.62] | 0% |  |
| 4.3 Autonomy | 9 | 551 | Fixed | -0.00 [-0.17; 0.17] | 26%  SgD: Chi2 p=0.84 |  |
| 4.3.1 Supratentorial stroke | 3 | 121 | Fixed | 0.03 [-0.33; 0.39] | 3.9% |  |
| 4.3.2 Brainstem stroke | 0 | 0 | Fixed | Not estimable | NA |  |
| 4.3.3 Cerebellum stroke | 0 | 0 | Fixed | Not estimable | NA |  |
| 4.3.4 Mixed or not determined | 6 | 430 | Fixed | -0.01 [-0.21; 0.18] | 38.6% |  |

Legend:

Autonomy: combination of barthel index, functional independence measure, activities of daily living and instrumental activities of daily living scales.

Mediolateral postural deviation: combination of weight bearing asymmetry and mediolateral position of center of pressure

Abbreviations: ADL, activities of daily living; BBS, berg balance scale; CI, confidence interval; COP, center of pressure; CPI, cardiopulmonary intervention; EO, eyes open; EC, eyes closed; IADL, instrumental activities of daily living; FIM, functional independence measure; FTT, functional task training; MD, mean difference; MS, muscle strengthening; MM, musculoskeletal mobilization; NA, not applicable; NPI, neurophysiological intervention; PASS, postural assessment scale for stroke; PT, physical therapy; RCT, randomized controlled trials; SgD, subgroup difference; SMD, standardized mean difference; X, mediolateral position of COP; Y, anteroposterior position of COP; WB, weight bearing.
