# Supplementary material for: Limited evidence of physical therapy on balance after stroke: A systematic review and meta-analysis
Source: PLoS One. 2019 Aug 29;14(8):e0221700. doi: 10.1371/journal.pone.0221700 (PMC6715189; doi:10.1371/journal.pone.0221700)
Supplement: S11 Table — (DOCX) [file pone.0221700.s025.docx]

**S10 Table. Summary of findings and quality of the evidence**

**S10A Table. For PT compared to no treatment**

| Physical therapy compared to no treatment after stroke | | | | |
| --- | --- | --- | --- | --- |
| Patient or population: Adult stroke patients  Intervention: Physical therapy  Comparison: No treatment | | | | |
| Outcomes | **Standardized mean difference [95% CI]** | **No of participants (studies)** | **Quality of the evidence (GRADE)** | **Comments** |
| Balance  Post-intervention effects | 0.46 [0.37; 0.56] | 37 studies,  1721 participants | ⊕⊕⊕⊝ **moderate** | Low heterogeneity. Many studies had high and unclear risks of bias, but sensitive analyses based on the methodological quality did not change the direction of the effect. |
| Balance  Persisting effects | 0.29 [-0.02; 0.59] | 11 studies,  493 participants | ⊕⊝⊝⊝⊝ **very low** | Substantial heterogeneity. Many studies had high and unclear risks of bias. |
| Mediolateral postural deviation EO  Post-intervention effects | -0.23 [-0.36; -0.09] | 11 studies, 329 participants | ⊕⊕⊝⊝ **low** | No heterogeneity. Most studies had high and unclear risks of bias. |
| Mediolateral postural deviation, EO  Persisting effects | -0.28 [-1.03, 0.47] | 2 studies,  34 participants | ⊕⊝⊝⊝ **very low** | No heterogeneity. Most studies had high and unclear risks of bias. |
| Postural stability, EO  Post-intervention effects | 0.47 [0.29; 0.65] | 16 studies,  504 participants | ⊕⊕⊝⊝ **low** | Low heterogeneity. Many studies had high and unclear risks of bias. |
| Postural stability, EO  Persisting effects | 0.31 [-0.14, 0.76] | 3 studies,  80 participants | ⊕⊝⊝⊝ **very low** | Low heterogeneity. Studies had high and unclear risks of bias. |
| Autonomy  Post-intervention effects | 0.36 [0.23, 0.49] | 15 studies,  941 participants | ⊕⊕⊕⊝ **moderate** | No heterogeneity. Many studies had high and unclear risks of bias, but sensitive analyses based on the methodological quality did not change the direction of the effect. |
| Autonomy  Persisting effects | 0.36 [0.13; 0.58] | 6 studies,  312 participants | ⊕⊝⊝⊝ **very low** | No heterogeneity. Many studies had high and unclear risks of bias. |
| EO, eyes open; CI, Confidence interval | | | | |
| GRADE Working Group grades of evidence High quality: Further research is very unlikely to change our confidence in the estimate of effect. Moderate quality: Further research is likely to have an important impact on our confidence in the estimate of effect and may change the estimate. Low quality: Further research is very likely to have an important impact on our confidence in the estimate of effect and is likely to change the estimate. Very low quality: We are very uncertain about the estimate. | | | | |

**S10B Table. For PT compared to sham treatment/usual care**

| Physical therapy compared to sham treatment or usual care after stroke | | | | |
| --- | --- | --- | --- | --- |
| Patient or population: Adult stroke patients  Intervention: Physical therapy  Comparison: sham treatment or usual care | | | | |
| Outcomes | **Standardized mean difference (95% CI)** | **No of participants (studies)** | **Quality of the evidence (GRADE)** | **Comments** |
| Balance  Post-intervention effects | 0.43 [0.28; 0.59] | 46 studies,  2051 participants | ⊕⊕⊝⊝ **low** | Substantial heterogeneity. Many studies had high or unclear risks of bias. |
| Balance  Persisting effects | 0.18 [0.06; 0.30] | 18 studies,  1150 participants | ⊕⊝⊝⊝ **very low** | Moderate heterogeneity. Many studies had high and unclear risks of bias. Potential publication bias. |
| Mediolateral postural deviation, EO  Post-intervention effects | -0.15 [-0.52; 0.21] | 4 studies,  122 participants | ⊕⊝⊝⊝ **very low** | Moderate heterogeneity. Most studies had high and unclear risks of bias. |
| Mediolateral postural deviation, EO  Persisting effects | NA | 0 study,  0 participant | NA | No data |
| Postural stability, EO  Post-intervention effects | 0.96 [0.55; 1.37] | 15 studies,  574 participants | ⊕⊝⊝⊝ **very low** | Substantial heterogeneity. Most studies had high or unclear risks of bias. |
| Postural stability, EO  Persisting effects | 0.32 [0.02, 0.62] | 2 studies,  178 participants | ⊕⊝⊝⊝  **very low** | No heterogeneity. Studies had high and unclear risks of bias. |
| Autonomy  Post-intervention effects | 0.26 [0.01; 0.51] | 16 studies,  805 participants | ⊕⊝⊝⊝  **very low** | Substantial heterogeneity. Many studies had high or unclear risks of bias. |
| Autonomy  Persisting effects | -0.002 [-0.17; 0.17] | 9 studies,  551 participants | ⊕⊝⊝⊝  **very low** | Low heterogeneity. Many studies had high or unclear risks of bias. Potential publication bias. |
| EO, eyes open; CI, Confidence interval; NA, Not applicable | | | | |
| GRADE Working Group grades of evidence High quality: Further research is very unlikely to change our confidence in the estimate of effect. Moderate quality: Further research is likely to have an important impact on our confidence in the estimate of effect and may change the estimate. Low quality: Further research is very likely to have an important impact on our confidence in the estimate of effect and is likely to change the estimate. Very low quality: We are very uncertain about the estimate. | | | | |
