# Supplementary material for: Limited evidence of physical therapy on balance after stroke: A systematic review and meta-analysis
Source: PLoS One. 2019 Aug 29;14(8):e0221700. doi: 10.1371/journal.pone.0221700 (PMC6715189; doi:10.1371/journal.pone.0221700)
Supplement: S1 Protocol — (PDF) [file pone.0221700.s026.pdf]

BMJ Open

Efficiency of physical therapy on postural imbalance after stroke: study protocol for a systematic review and meta-analysis

A Hugues,[1,2,3] J Di Marco,[4] P Janiaud,[5] Y Xue,[6] J Pires,[7,8] H Khademi,[9] M Cucherat,[5] I Bonan,[10] F Gueyffier,[5,11] G Rode[1,2,3]

To cite: Hugues A, Di Marco J, Janiaud P, *et al*. Efficiency of physical therapy on postural imbalance after stroke: study protocol for a systematic review and meta-analysis. *BMJ Open* 2017;**7**: e013348. doi:10.1136/bmjopen-2016-013348

▸ Prepublication history for this paper is available online. To view these files please visit the journal online (http://dx.doi.org/10.1136/bmjopen-2016-013348).

This protocol study has been presented at the 9th World Congress for NeuroRehabilitation in Philadelphia (USA) from 10 to 13 May 2016 (http://wcnr2016.org/).

Received 6 July 2016
Revised 11 November 2016
Accepted 3 January 2017

For numbered affiliations see end of article.

Correspondence to
A Hugues;
huguesaurelien@gmail.com

ABSTRACT

Introduction: Stroke frequently results in balance disorders, leading to lower levels of activity and a diminution in autonomy. Current physical therapies (PT) aiming to reduce postural imbalance have shown a large variety of effects with low levels of evidence. The objectives are to determine the efficiency of PT in recovering from postural imbalance in patients after a stroke and to assess which PT is more effective.

Methods and analysis: We will search several databases from inception to October 2015. Only randomised controlled trials assessing PT to recover from poststroke postural imbalance in adults will be considered.

Outcome measures will be the Berg Balance Scale (BBS), the Postural Assessment Scale for Stroke (PASS), the 'weight-bearing asymmetry' (WBA), the 'centre of pressure' (COP) and the 'limit of stability' (LOS). WBA, COP and LOS are measured by a (sitting or standing) static evaluation on force plate or another device.

Two independent reviewers will screen titles, abstracts and full-text articles, evaluate the risk of bias and will perform data extraction. In addition to the outcomes, measures of independence will be analysed. This study will aim at determining the effects of PT on the function (WBA, COP, LOS), the activity (BBS, PASS) and the independence of patients. Subgroup analyses will be planned according to the location of brain lesion (hemispheric, brainstem or cerebellum), the time since stroke (early, late, chronic), the PT (type, main aim (direct effect or generalisation), overall duration), the type of approaches (top-down or bottom-up) and the methodological quality of studies.

Ethics and dissemination: No ethical statement will be required. The results will be published in a peer-reviewed journal. This meta-analysis aims at managing the rehabilitation after postural imbalance by PT after a stroke.

Trial registration number: Prospero CRD42016037966; Pre-results.

Strengths and limitations of this study

- To the best of our knowledge, there are few systematic reviews and meta-analyses in the literature that assess the evidence of physical therapies (PT) for rehabilitation of postural imbalance after a stroke.
- This study will compare the efficiency of all PT used after a stroke to one another.
- A series of subgroup analyses will address relevant clinical issues.
- There are several outcomes to assess postural imbalance (function and activity) that may limit comparison across studies.
- The results of this meta-analysis will be helpful for clinicians to define rehabilitation strategies for improving postural imbalance after stroke.

INTRODUCTION

Background

A stroke is defined as 'rapidly developing clinical signs of focal (at times global) disturbance of cerebral function, lasting more than 24 h or leading to death with no apparent cause other than that of vascular origin'.[1] Stroke is the third cause of death and the first cause of acquired adult disability in the world (WHO). In the USA, 795 000 people suffer from a stroke every year.[2] Stroke leads to a long-term limitation of activity and disability. In France, 80.5% of the people with self-reported stroke declare a limitation (light or severe) in activities of daily living (ADL) and one in three stroke survivors are dependent.[3] In New Zealand, 71% of 5 years poststroke patients present a neurological impairment, assessed by the National Institutes of Health Stroke Scale. A restriction of activity was present in 31.4% of the patients assessed by the Modified Rankin Scale and in 35.4% assessed by the Barthel Index (BI).[4] Among limitations of activity, the postural imbalance is frequently found. Eighty-three per cent of patients with acute stroke present a postural imbalance.[5] The risk of fall is increased by 73% in the 6 months following a stroke.[6] At a chronic

stage, the quality of life is associated with the postural imbalance.[7] Postural rehabilitation seems to be crucial to achieve independence in ADL after stroke.

Human posture refers to the relative disposition of body parts.[8] Postural control aims to maintain body stabilisation based on a sensorimotor complex skill and body orientation, based on internal representation of body scheme.[9 10] Postural imbalance following stroke is defined by: (1) a larger weight-bearing asymmetry (WBA) toward the unaffected limb, in a quiet standing posture;[11–18] (2) an increased body sway of the centre of the pressure (COP);[12 13 15 19] (3) a decrease in the limits of stability (LOS);[12 20] (4) an excessive reliance on visual input[21–24] and (5) an impairment of anticipatory postural adjustments and postural reactions after external perturbations.[25 26]

State of the art

Different physical therapies (PT) aim at reducing postural imbalance. Current recommendations are limited for daily clinical practice: the level of evidence is too low and it is based on few systematic reviews and meta-analyses. The recommendations in the French evidence-based clinical practice guidelines for PT in patients after stroke were based on only 16 clinical studies.[27] Furthermore, these guidelines are not specific to postural disability and propose a rather global rehabilitation.[28 29] It is therefore necessary to assess the efficiency of PT in the recovery of postural control after stroke.

Regarding the literature, some meta-analyses have evaluated the effects of a single technique on postural imbalance like balance training using a platform with biofeedback,[30] functional electrical stimulation,[31] repetitive task training,[32] water-based exercises,[33] virtual reality,[34–36] ankle-foot orthosis,[37] aerobic exercises,[38] physical fitness training[39] or whole body vibration.[40 41] In view of the tremendous growth in the number of randomised controlled trials, it seems to be essential to evaluate one PT compared with another or the association of PT compared with control or usual care. Veerbeek *et al*[42] have evaluated the effects of PT after stroke on all outcomes based on the International Classification of Functioning, Disability and Health (ICF) and not only the balance. Pollock *et al*[43] have investigated the function and mobility recovery by PT after stroke. Compared with previous studies, the aim of this systematic review and meta-analysis is to perform a review only focused on the effects of PT on postural imbalance after stroke with identification of different parameters.

Finally, in this review and meta-analysis, we also propose to categorise the different PT according to the involved 'top-down' and 'bottom-up' processing. This processing refers to two types of interaction between sensorimotor (implicit) and cognitive (explicit) representations involved in rehabilitation. Top-down approach aims at training the patient to voluntarily compensate for his deficit and requires awareness of the disorder although bottom-up approach does not require awareness of the disorder. This categorisation has already been used in a previous Cochrane meta-analysis about cognitive rehabilitation for another spatial cognition deficit (spatial neglect).[44–46]

OBJECTIVES

The aims are: (1) to determine the efficiency of PT on the recovery of postural imbalance in adult patients after stroke and (2) to assess which PT is more effective when compared with one another.

METHODS

We will use the guide from The Cochrane Collaboration entitled 'Cochrane Handbook for Systematic Reviews of Interventions' (V.5.1.0)[47] and the software (RevMan 5.3) to construct this meta-analysis. The recommendations from Preferred Reporting Items for Systematic Reviews and Meta-Analyses (PRISMA) statement will also be followed.([48]) No ethical statement will be required for this review and meta-analysis. Results of this research will be published. These results will contribute to improve the therapeutic strategy of patients with stroke.

Criteria for considering studies for this review

Type of studies

We will include all randomised controlled trials. The allocation between two or several groups will have to be correctly randomised. Trials without control group or those with quasi-random allocation will be excluded.

Types of participants

We will include all trials which have included human adult patients (over 18 years old) after a first or recurrent stroke. Stroke is defined, according to the WHO, as 'rapidly developing clinical signs of focal (at times global) disturbance of cerebral function, lasting more than 24 h or leading to death with no apparent cause other than that of vascular origin'.[1] Therefore, the positive diagnosis is based on clinical examination. It is not compulsory to include the imaging diagnosis. Transient ischaemic accidents (TIAs) will be excluded because all neurological symptoms disappear ('TIAs are brief episodes of neurological dysfunction resulting from focal cerebral ischaemia not associated with permanent cerebral infarction.').[49]

Types of interventions

The selection process will not be based on the type or the nature of the PT in trials. We will select all trials assessing a PT whatever it may be and whatever its aim (upper limb, lower limb, posture, gait, spasticity and so on). This meta-analysis will not be limited to PT, the direct and immediate objective of which is to reduce postural imbalance. This possible expansion or generalisation of effects may be observed after intervention in rehabilitation.

The PT is defined by the World Confederation for Physical Therapy (WCPT) as 'services to individuals and populations to develop, maintain and restore maximum movement and functional ability throughout the lifespan' and 'physical therapy is concerned with identifying and maximising quality of life and movement potential within the spheres of promotion, prevention, treatment/intervention, habilitation and rehabilitation' (http://www.wcpt.org/policy/ps-descriptionPT).

Types of outcome measures

Outcomes will be selected following the recommendations of the ICF. Immediate outcomes after the end of PT and delayed outcomes after a follow-up time will be included.

Primary outcomes

The Berg Balance Scale (BBS) assesses the functional postural abilities of patients in several conditions (lying on the back, sitting, standing, leaning forward, change of position and so on). This scale is composed of 14 items. The maximal score, reflecting the best functional postural abilities, is 56 points. The choice of the scale is based on its validation in patients with stroke and on its good metrological qualities, making it a reference scale.[50–54]

The Postural Assessment Scale for Stroke (PASS) also evaluates the functional postural abilities of patients with stroke in several conditions (lying on the back, sitting, standing and while changing (these) positions). This scale is composed of 12 items. The maximal score, reflecting the best functional postural abilities, is 36 points. Its metrological qualities are good, particularly during the first 3 months.[55 56]

The two scales exhibit a clinical relevance in assessment of postural imbalance in patients with stroke. They express the level of activity. Therefore, measured changes reflect modifications of postural abilities of patients in daily living.

The outcomes pertaining to balance and postural control will be the WBA, the COP and the LOS. These parameters will be measured by a (sitting or standing) static evaluation on force plate or another device.[17 51 52]

Secondary outcomes

The outcomes will be the BI, the Functional Independence Measure (FIM), the scale for instrumental activities of daily living (IADL) and the scale for ADL, reflecting the level of autonomy.

Only the primary outcomes will be considered for selection of trials.

Search methods for identification of studies

We will search the following electronic bases from their inception to October 2015: Medline, Embase, PEDro, Cochrane Central Register of Controlled Trials, Pascal and Francis. The search strategy will involve three kinds of terms: 'stroke', 'posture' and 'physical therapy'. This search strategy is described in table 1.

All published and unpublished studies, conferences or presentations will be searched without restriction in languages. The library services of three universities (Université Claude Bernard Lyon1, Université Paris 5 Descartes and Université Paris 6 Pierre et Marie Curie) and two hospital centres (Hospices Civils de Lyon, Assistance Publique-Hôpitaux de Paris) will be requested to access the unpublished and published documents.

Data collection and analysis

Selection of studies

The process of selecting the search results will be carried out on the basis of the selection criteria in three successive steps: (1) by reading the titles, (2) by reading the abstracts and then (3) by reading the full texts. Each one of these steps will be separately performed by two

Table 1 Search strategy in PubMed

1	exercise movement techniques OR physical therapy modalities OR learning OR pract* OR train* OR rehabilitation* OR therapeutic* OR therapy OR therapies OR exercise* OR physiotherap* OR neurorehabilitation OR neurophysiological OR orthopaed* OR treatment OR approach* OR concept OR home rehabilitation OR self-guided program* OR fitness OR stretching OR sport OR program* OR movement OR protocol* OR intervention OR activit* OR regim* OR recovery
2	(occupational OR physical OR manual) AND (therapy OR therapies OR therapist OR therapeutic OR therapeutics)
3	#1 OR #2
4	posture OR equilibrium OR balance OR postural balance OR weight bearing OR weight shift OR lateropulsion OR pusher OR pushing OR postural imbalance OR postural asymmetry OR postural control OR postural stability OR postural instability OR postural perturbation OR postural disorders OR postural deficit OR postural trouble OR postural sway OR postural tilt OR postural shift OR body sway OR upright stance OR (weight AND (distribut* OR transfer*))
5	(cerebrovascular OR cerebro-vascular OR cerebral OR intracran* OR hemispheric) AND (accident OR hemorrhag* OR haemorrhag* OR infarct* OR ischemi* OR thrombotic OR thrombosis OR emboli* OR hematoma OR haematoma OR bleed OR damage OR lesion OR occlus*)
6	stroke OR poststroke OR post-stroke OR hemipleg* OR hemipar* OR paretic OR paresis OR CVA
7	(right OR left) AND brain AND (lesion OR damage)
8	#5 OR #6 OR #7
9	meta-analysis OR review* OR animal* OR child* OR cerebral pals* OR case-report OR traumatic brain injury
10	#3 AND #4 AND #8 NOT #9

independent authors (AH and JDM). For the selection on the basis of titles, all studies, selected by one of these two authors, will be accepted for the next step of the selection process. For the two subsequent steps of selection, an agreement between the two authors will have to be found. In case of disagreement, three more authors (IB, FG, GR) will have to decide by consensus. The authors of the trials will be contacted if information needed for the selection process is unclear or missing.

The studies published in journals judged as stand-alone according to the analysis of Jeffrey Beall (https://scholarlyoa.com/individual-journals/), which is based on objective and clearly identified criteria (https://scholarlyoa.files.wordpress.com/2015/01/criteria-2015.pdf), will be excluded.

Cross-over trials will be included if: (1) the order of interventions has been randomised and if (2) the potential effects of the first intervention have not impacted the potential effects of the second one. They will be considered as randomised controlled trials. Moreover, some cross-over trials can present a special design: a single assessment during the intervention instead of an assessment before and one after, as is usually the case. These types of design are specifically used for some types of intervention (orthosis and so on). These cross-over trials will be included if: (1) the conditions set above regarding cross-over trials are validated (the randomised order and the absence of impact of the first intervention on the second one) and if (2) a spontaneous recovery is not possible during the time between the two interventions.

No study will be excluded because of the language of the report. Those written in languages other than French or English will be translated by the authors: YX for those written in Chinese, HK for those written in Persian, JP for those written in Portuguese.

Data extraction and management

Data extraction will be carried out independently by two authors (AH and JDM). Agreement between these two authors will have to be found. In case of disagreement, three more authors (IB, FG, GR) will have to decide by consensus. The authors of included trials will be contacted if some data are unclear or missing. Data extraction will include:

1. the design of study;
2. the details of the population: size of the population, age, gender, time since stroke, side of the paresis, unilateral or bilateral stroke, first ever or the recurrent stroke, the imaging diagnostic with the aetiologic and the localisation of stroke lesions;
3. the methodological quality of trials: details of random process, blinding, dropout, reporting and others;
4. the PT: overall duration of PT, the aims and the most important characteristics of each PT;
5. the outcomes: all outcomes measured and specifically the BBS, the PASS, the WBA, the COP, the LOS, the BI, the FIM, the IADL and the ADL will also be extracted;
6. the prior submission to an ethics committee or the respect of the declaration of Helsinki on human clinical trials.

Assessment of the risk of bias in included studies

The methodological quality of all included trials will be separately assessed by two independent authors (AH and JDM). Agreement between these two authors will have to be found. In case of disagreement, three more authors (IB, FG, GR) will have to decide by consensus. This evaluation will be based on the seven relevant domains in the 'risk of bias' tool of Cochrane Handbook for Systematic Review of Interventions: (1) random sequence generation, (2) allocation concealment, (3) blinding of participants and personnel, (4) blinding of outcome assessment, (5) incomplete outcome data, (6) selective reporting and (7) other biases.

The level of risk of bias will be determined for each domain: (1) high level, (2) unclear level or (3) low level.

Measures of treatment effect

The statistical analysis will be performed according to the recommendations of the Cochrane Handbook and using the software of Cochrane Collaboration, RevMan 5.3, available from the Cochrane website (http://tech.cochrane.org/revman). All outcomes will be continuous variables. The measurement of effects will be determined based on the change scores from baseline. Initially, a fixed-effect model will be used to compare the outcomes expressed in the same scale. The heterogeneity of the effects of trials will be evaluated by the χ^2 test and the I^2 test. Heterogeneity will be considered as substantial if the I^2 statistic ≥50% and $p<0.10$. If heterogeneity is considered as substantial, reasons for this heterogeneity will be searched for and a random-effect model could be used for comparison. So, the mean difference, which is the absolute difference between the mean value in two groups in a trial, and its 95% CIs will be calculated. To express the PT effects on the function and the activity, it will be necessary to combine the outcomes measured in a variety of scales (measures of WBA, COP and LOS for the function, PASS and BBS for the activity). Thus, the standardised mean difference (SMD) and its 95% CIs will be calculated. The SMD expresses the size of the intervention effect in each trial relative to the variability observed in that trial. In Revman, the SMD is calculated based on the Hedges' g.

For the trials with more than two PT groups and to prevent a group being counted twice, we will determine which PT groups are relevant for pair-wise comparisons. Or, if all are relevant, a further possibility will be to include each pair-wise comparison separately and to divide evenly the shared group among the comparisons. For the trials for which results for a rehabilitation group are stratified, the absence of substantial heterogeneity will be verified before mixing the two subgroups of the same PT.

Data synthesis

The comparisons will focus on the effects of active PT versus: (1) no PT, (2) usual care, placebo or control PT and (3) another active PT. First, immediate outcomes will be analysed, then delayed outcomes (follow-up tests), if they have been evaluated.

Subgroup analysis and investigation of heterogeneity

Several subgroup analyses will investigate the effects of PT according to:

1. the type/nature of PT (eg, electromechanical devices including biofeedback, robotics and functional electrical stimulation, virtual reality, task-oriented training, gait training, vibration, non-invasive cerebral stimulation and so on);
2. the main therapeutic goal of PT; two groups will be established: (1) PT aiming mainly at the recovery of postural imbalance and (2) PT not specifically focused on the recovery of postural imbalance;
3. the localisation of brain lesion; to this purpose, three subgroups will be identified: (1) hemispheric stroke, (2) brainstem stroke and (3) cerebellum stroke; a subgroup analysis will also investigate the effects of PT according to the side of the hemispheric lesion (right/left);
4. the type of processing 'bottom-up' or 'top-down';
5. the methodological quality of trials; two subgroups will be identified: (1) the trials in which all criteria of methodological quality, detailed in the part entitled 'assessment of risk of bias in included studies', will present a low risk and (2) the trials in which at least one of these criteria will present an unclear or high risk;
6. the trials assessing or not the level of autonomy (BI, FIM, IADL, ADL);
7. the time since stroke; to this purpose, three subgroups will be identified: (1) early (≤30 days), (2) late (<180 days) and (3) chronic stroke (≥180 days).[57]

We will plan a metaregression of the effects according to the overall duration of PT.

Considering the high risk of heterogeneity for the different PT investigated, a network meta-analysis is, at the present, not envisaged.

DISCUSSION

Postural imbalance is frequent in patients with stroke at early, late or chronic stage. It affects walking abilities, independence and quality of life.[7] Therefore, reduction of postural imbalance in patients with stroke is a relevant objective of PT, in order to increase the level of autonomy. This meta-analysis aims (1) at determining the efficiency of PT on the recovery of postural imbalance in adult patients after stroke and (2) at assessing which PT is more effective when compared with one another. To this purpose, this systematic review and meta-analysis aims at upgrading and improving the rehabilitation of postural imbalance by a complete analysis of all PT. But our objective is to compare the effects of PT and to improve the understanding of these PT effects, using subgroup analyses.

Stroke leads to a large range of clinical subtypes of postural imbalance and related underlying disorders. One of the major issues regarding the rehabilitation of postural imbalance after stroke is the heterogeneity of stroke and the patients' deficits. For example, postural imbalance differs depending on the location and the size of the brain damage.[58] The patients with right hemispheric lesions show a greater WBA and weaker balance abilities.[13 18 58] Moreover, a second major issue is the variety of PT: human practice and/or electromechanical devices, several different (re)learning methods (biofeedback, repetitive tasks, tasks oriented and so on), 'top-down' and 'bottom-up' approaches and so on.

Therefore, many relevant issues regarding the rehabilitation of postural imbalance after stroke are asked: Which PT is the best? What is the most relevant between specific PT focused on postural imbalance and generalisation effects of non-specific PT? Does the postural imbalance rehabilitation only involve a sensorimotor approach? What is the advantage of technology? What is the efficiency of PT according to the time since stroke? Which intensity of PT is the most efficient? What are the effects on the autonomy and the quality of life? The previously detailed subgroup analyses could describe the effects of each PT and, thus, contribute to propose a guideline for rehabilitation of postural imbalance in patients with stroke. One relevant issue may be to better identify the appropriate PT for one patient at one time after stroke.

Author affiliations
[1]Service de médecine physique et réadaptation, Hôpital Henry-Gabrielle, Hospices Civils de Lyon, Saint-Genis-Laval, France
[2]Inserm UMR-S 1028, CNRS UMR 5292, ImpAct, Centre de Recherche en Neurosciences de Lyon, Université Lyon, Bron, France
[3]Plate-forme Mouvement et Handicap, Hôpital Henry-Gabrielle, Hospices Civils de Lyon, Saint-Genis-Laval, France
[4]Service de médecine physique et réadaptation, Hôpital Raymond Poincaré, Assistance Publique des Hôpitaux de Paris, Garches, France
[5]UMR 5558 CNRS Lyon, Université de Lyon 1, Lyon, France
[6]Pôle Information Médicale Évaluation Recherche, Hospices Civils de Lyon, Lyon, France
[7]Rovisco Pais Rehabilitation Centre, Tocha, Portugal
[8]Medicine Faculty of Oporto University, Oporto, Portugal
[9]International Agency of Research on Cancer, World Health Organisation, Lyon, France
[10]Service de médecine physique et de réadaptation, CHU Rennes, Rennes, France
[11]Service de Pharmacologie Toxicologie, Groupement Hospitalier Est, Hospices Civils de Lyon, Bron, France

Twitter Follow AURELIEN HUGUES @A_Hugues

Acknowledgements We sincerely acknowledge Mrs Fanny Blanchon, English Professor and Mr Philip Robinson of the Research Department of the Hospices Civils de Lyon for their help in English translation.

Contributors AH and JDM have participated and performed all steps of this meta-analysis: preliminary search, conception and design of the protocol, and drafting of this publication. All this work was assisted and supervised by IB,

FG and GR. MC has supported AH and JDM for the statistical processing. PJ has supported AH and JDM for the search in electronic databases. YE, JP and HK have been in charge to translate the studies in Chinese, Portuguese and Persian languages, respectively.

Funding This research received no specific grant from any funding agency in the public, commercial or not-for-profit sectors.

Competing interests PJ presents a conflict of interest with GlaxoSmithKline. Her PhD thesis was supported by GSK. PJ will contribute in this study by helping to search electronic databases. She will not participate in the selection process and in the analyses.

Provenance and peer review Not commissioned; externally peer reviewed.

Data sharing statement Unpublished data from this study will be available by contacting corresponding author. Unpublished data will be shared.

Open Access This is an Open Access article distributed in accordance with the Creative Commons Attribution Non Commercial (CC BY-NC 4.0) license, which permits others to distribute, remix, adapt, build upon this work non-commercially, and license their derivative works on different terms, provided the original work is properly cited and the use is non-commercial. See: http://creativecommons.org/licenses/by-nc/4.0/

REFERENCES

1. Hatano S. Experience from a multicentre stroke register: a preliminary report. *Bull World Health Organ* 1976;54:541–53.
2. Mozaffarian D, Benjamin EJ, Go AS, *et al*. Heart disease and stroke statistics—2015 update: a report from the American Heart Association. *Circulation* 2015;131:e29–322.
3. Schnitzler A, Woimant F, Tuppin P, *et al*. Prevalence of self-reported stroke and disability in the French adult Population: A Transversal Study. *PLoS ONE* 2014;9:e115375.
4. Feigin VL, Barker-Collo S, Parag V, *et al*. Auckland Stroke Outcomes Study. Part 1: gender, stroke types, ethnicity, and functional outcomes 5 years poststroke. *Neurology* 2010;75:1597–607.
5. Tyson SF, Hanley M, Chillala J, *et al*. Balance disability after stroke. *Phys Ther* 2006;86:30–8.
6. Forster A, Young J. Incidence and consequences of falls due to stroke: a systematic inquiry. *BMJ* 1995;311:83–6.
7. Schmid AA, Van Puymbroeck M, Altenburger PA, *et al*. Balance is associated with quality of life in chronic stroke. *Top Stroke Rehabil* 2013;20:340–6.
8. Thomas A. *Equilibre et equilibration*. Paris: Masson, 1940.
9. Amblard B, Crémieux J, Marchand AR, *et al*. Lateral orientation and stabilization of human stance: static versus dynamic visual cues. *Exp Brain Res.* 1985;61:21–37.
10. Massion J. *Cerveau et motricité, fonctions sensori-motrices*. Presses Universitaires de France, 1997.
11. Sackley C. The relationships between weight-bearing asymmetry after stroke, motor function and activities of daily living. *Physiotherapy Theory and Practice* 1990;6:179–85.
12. Pérennou D, Pélissier J, Amblard B. La posture et le contrôle postural du patient cérébrolésé vasculaire : une revue de la littérature. *Ann Réadaptation Médecine Phys* 1996;39:497–513.
13. Rode G, Tilikete C, Boisson D. Predominance of postural imbalance in left hemiparetic patients. *Scand J Rehab Med* 1997;29:155–64.
14. Rode G, Tiliket C, Charlopain P, *et al*. Postural asymmetry reduction by vestibular caloric stimulation in left hemiparetic patients. *Scand J Rehab Med* 1998;30:9–14.
15. Cheng PT, Liaw MY, Wong MK, *et al*. The sit-to-stand movement in stroke patients and its correlation with falling. *Arch Phys Med Rehabil* 1998;79:1043–6.
16. Geurts ACH, de Haart M, van Nes IJW, *et al*. A review of standing balance recovery from stroke. *Gait Posture* 2005;22:267–81.
17. Genthon N, Rougier P, Gissot AS, *et al*. Contribution of each lower limb to upright standing in stroke patients. *Stroke* 2008;39:1793–9.
18. Ishii F, Matsukawa N, Horiba M, *et al*. Impaired ability to shift weight onto the non-paretic leg in right-cortical brain-damaged patients. *Clin Neurol Neurosurg* 2010;112:406–12.
19. Dickstein R, Abulaffio N. Postural sway of the affected and nonaffected pelvis and leg in stance of hemiparetic patients. *Arch Phys Med Rehabil* 2000;81:364–7.
20. Di Fabio RP, Badke MB. Extraneous movement associated with hemiplegic postural sway during dynamic goal-directed weight redistribution. *Arch Phys Med Rehabil* 1990;71:365–71.
21. Bonan IV, Yelnik A, Laffont I, *et al*. Sélection des afférences sensorielles dans l'équilibration de l'hémiplégique après accident vasculaire cérébral. *Ann. Réadaptation Médecine Phys* 1996;39:157–63.
22. Bonan IV, Colle FM, Guichard JP, *et al*. Reliance on visual information after stroke. Part I: balance on dynamic posturography. *Arch Phys Med Rehabil* 2004;85:268–73.
23. Bonan IV, Marquer A, Eskiizmirliler S, *et al*. Sensory reweighting in controls and stroke patients. *Clin Neurophysiol* 2013;124:713–22.
24. Yelnik AP, Kassouha A, Bonan IV, *et al*. Postural visual dependence after recent stroke: Assessment by optokinetic stimulation. *Gait Posture* 2006;24:262–9.
25. Di Fabio RP. Lower extremity antagonist muscle response following standing perturbation in subjects with cerebrovascular disease. *Brain Res* 1987;406:43–51.
26. Garland SJ, Stevenson TJ, Ivanova T. Postural responses to unilateral arm perturbation in young, elderly, and hemiplegic subjects. *Arch Phys Med Rehabil* 1997;78:1072–7. http://dx.doi.org/10.1016/S0003-9993(97)90130-1
27. Haute Autorité de Santé. Accident vasculaire cérébral : méthodes de rééducation de la fonction motrice chez l'adulte Méthode "Recommandations pour la pratique clinique" 2012.
28. Duncan PW, Zorowitz R, Bates B, *et al*. Management of adult stroke rehabilitation care: a clinical practice guideline. *Stroke* 2005;36:e100–43.
29. National Health Service. Stroke rehabilitation in adults clinical guideline. 2013.
30. Barclay-Goddard R, Stevenson T, Poluha W, *et al*. Force platform feedback for standing balance training after stroke. *Cochrane Database Syst Rev* 2004;(4):CD004129.
31. Glanz M, Klawansky S, Stason W, *et al*. Functional electrostimulation in poststroke rehabilitation: a meta-analysis of the randomized controlled trials. *Arch Phys Med Rehabil* 1996;77:549–53.
32. French B, Thomas L, Leathley M, *et al*. Does repetitive task training improve functional activity after stroke? A Cochrane systematic review and meta-analysis. *J Rehabil Med* 2010;42:9–14.
33. Mehrholz J, Kugler J, Pohl M. Water-based exercises for improving activities of daily living after stroke. *Cochrane Database Syst Rev* 2011(1):CD008186.
34. Laver KE, George S, Thomas S, *et al*. Virtual reality for stroke rehabilitation. *Cochrane Database Syst Rev* 2015;2:CD008349.
35. Li Z, Han XG, Sheng J, *et al*. Virtual reality for improving balance in patients after stroke: a systematic review and meta-analysis. *Clin Rehabil* 2016;30:432–40.
36. Corbetta D, Imeri F, Gatti R. Rehabilitation that incorporates virtual reality is more effective than standard rehabilitation for improving walking speed, balance and mobility after stroke: a systematic review. *J Physiother* 2015;61:117–24.
37. Tyson SF, Kent RM. Effects of an ankle-foot orthosis on balance and walking after stroke: a systematic review and pooled meta-analysis. *Arch Phys Med Rehabil* 2013;94:1377–85.
38. Pang MY, Charlesworth SA, Lau RW, *et al*. Using aerobic exercise to improve health outcomes and quality of life in stroke: evidence-based exercise prescription recommendations. *Cerebrovasc Dis* 2013;35:7–22.
39. Saunders DH, Sanderson M, Brazzelli M, *et al*. Physical fitness training for stroke patients. *Cochrane Database Syst Rev* 2013;10:CD003316.
40. Yang X, Wang P, Liu C, *et al*. The effect of whole body vibration on balance, gait performance and mobility in people with stroke: a systematic review and meta-analysis. *Clin Rehabil* 2015;29:627–38.
41. Lu J, Xu G, Wang Y. Effects of whole body vibration training on people with chronic stroke: a systematic review and meta-analysis. *Top Stroke Rehabil* 2015;22:161–8.
42. Veerbeek JM, van Wegen E, van Peppen R, *et al*. What is the evidence for physical therapy poststroke? A systematic review and meta-analysis. *PLoS ONE* 2014;9:e87987.
43. Pollock A, Baer G, Campbell P, *et al*. Physical rehabilitation approaches for the recovery of function and mobility following stroke. *Cochrane Database Syst Rev* 2014;4:CD001920.
44. Bowen A, Hazelton C, Pollock A, *et al*. Cognitive rehabilitation for spatial neglect following stroke. *Cochrane* Database Syst Rev 2013;7:CD003586.
45. Rossetti Y, Jacquin-Courtois S, Calabria M, *et al*. Testing cognition and rehabilitation in unilateral neglect by wedge prism adaptation: multiple interplays between sensorimotor adaptation and spatial cognition. In: Kansaku K, Cohen LG, Birbaumer N, eds. *Systems neuroscience: from laboratory to clinical practice*. Japan: Springer, 2015:359–81.
46. Rode G, Lacour S, Jacquin-Courtois S, *et al*. Long-term sensorimotor and therapeutical effects of a mild regime of prism

adaptation in spatial neglect. A double-blind RCT essay. *Ann Phys Rehabil Med* 2015;58:40–53.
47. Higgins JPT, Green S, eds. Cochrane handbook for systematic reviews of interventions version 5.1.0 [updated March 2011]. The Cochrane Collaboration, 2011. http://www.cochrane-handbook.org
48. Moher D, Liberati A, Tetzlaff J, *et al*. Preferred reporting items for systematic reviews and meta-analyses: the PRISMA statement. *BMJ* 2009;339:b2535.
49. Easton JD, Saver JL, Albers GW, *et al*. Definition and evaluation of transient ischemic attack: a scientific statement for healthcare professionals from the American Heart Association/American Stroke Association Stroke Council; Council on Cardiovascular Surgery and Anesthesia; Council on Cardiovascular Radiology and Intervention; Council on Cardiovascular Nursing; and the Interdisciplinary Council on Peripheral Vascular Disease. The American Academy of Neurology affirms the value of this statement as an educational tool for neurologists. *Stroke* 2009;40:2276–93.
50. Tyson SF, DeSouza LH. Reliability and validity of functional balance tests post stroke. *Clin Rehabil* 2004;18:916–23.
51. Pérennou D, Decavel P, Manckoundia P, *et al*. Evaluation de l'équilibre en pathologie neurologique et gériatrique. *Ann Réadaptation Médecine Phys* 2005;48:317–35.
52. Yelnik A, Bonan I. Clinical tools for assessing balance disorders. *Neurophysiol Clin Neurophysiol* 2008;38:439–445.
53. Blum L, Korner-Bitensky N. Usefulness of the berg balance scale in stroke rehabilitation: a systematic review. *Phys Ther* 2008;88:559–66.
54. Tyson SF, Connell LA. How to measure balance in clinical practice. A systematic review of the psychometrics and clinical utility of measures of balance activity for neurological conditions. *Clin Rehabil* 2009;23:824–40.
55. Benaim C, Pérennou DA, Villy J, *et al*. Validation of a standardized assessment of postural control in stroke patients: the Postural Assessment Scale for Stroke Patients (PASS). *Stroke* 1999;30:1862–8.
56. Mao HF, Hsueh IP, Tang PF, *et al*. Analysis and comparison of the psychometric properties of three balance measures for stroke patients. *Stroke* 2002;33:1022–7.
57. Stinear C, Ackerley S, Byblow W. Rehabilitation is initiated early after stroke, but most motor rehabilitation trials are not: a systematic review. *Stroke* 2013;44:2039–45.
58. Pérennou D, Benaim C, Rouget E, *et al*. Postural balance following stroke: towards a disadvantage of right brain-damaged hemisphere. *Rev Neuro* 1999;155:281–90.
